# Secular trends in physical fitness of Slovenian boys and girls aged 7 to 15 years from 1989 to 2019: a population-based study

**DOI:** 10.1038/s41598-022-14813-7

**Published:** 2022-06-21

**Authors:** Ana Radulović, Gregor Jurak, Bojan Leskošek, Gregor Starc, Rok Blagus

**Affiliations:** 1 Faculty of Medicine, Institute for Biostatistics and Medical Informatics, University of Ljubljana, Vrazov trg 2, 1000 Ljubljana, Slovenia; 2 Faculty of Sports, University of Ljubljana, Gortanova ulica 22, 1000 Ljubljana, Slovenia; 3 Faculty of Mathematics, Natural Sciences and Information Technologies, University of Primorska, Glagoljaška ulica 8, 6000 Koper, Slovenia; 4 Center for Control and Prevention of Infectious Diseases, Institute of Public Health of Montenegro, Džona Džeksona bb, 81000 Podgorica, Montenegro

**Keywords:** Health care, Medical research

## Abstract

Using the population-based data we aim to estimate the general population trends of multiple components of physical fitness of children, identify critical structural changes in these trends, and evaluate the potential changes in differences in the test scores between the children. During the entire study period, 1989–2019, median body mass index and triceps skinfold increased in both genders and all age groups. Muscular fitness, in general, showed negative trends, with some exceptions: during the post-2010 period, children were mostly experiencing the improvement of isometric strength of the upper body. The neuromuscular components of physical fitness showed positive trends, especially in girls. Cardiorespiratory fitness has been declining in all age groups until the last decade, with the largest decreases occurring before 2000. In the last decade, the trends reversed. The flexibility indicator revealed the largest differences between boys and girls, with boys mainly experiencing negative and girls mostly positive trends. The variability of the test scores mostly increased during the study period. This increasing variance suggests that—despite generally favourable trends in the last decade—children in Slovenia have been facing increasing inequalities in their development, which can potentially lead to future inequalities in health.

## Introduction

There is strong evidence that physical fitness (PF) is a powerful marker of health in children^1,2^. Cardiorespiratory fitness (CRF) and muscular fitness (MF) were shown to be particularly important in this regard^3,4^, therefore the American Heart Association highlighted their clinical value in youth and recommended their assessment^5^. For example, data from the Swedish registries have added compelling evidence linking both components of PF at late adolescence with mid-adulthood all-cause mortality, as well as cardiovascular and cancer disease mortality^6–9^. Both PF components have also been shown to predict severe, chronic and irreversible all-cause disease as indicated by granted disability pensions, with a low PF level predicting future cardiovascular, musculoskeletal, neurological and psychiatric diseases^10–13^. Therefore, it is imperative to assess PF in youth to obtain relevant information about the health status of the youth population.

Monitoring the population PF levels for the early identification of unfavourable trends is therefore crucial so that appropriate and early interventions aimed at reversing the unfavourable trends can be implemented^14,15^. The assessments of the secular trends of PF of children have been somewhat understudied and have been mostly derived from temporal studies, relying mostly on cross-sectional designs, usually comparing only individual or joint age-groups of children at two or three time-points, predominantly comparing data on a relatively small number of samples of one or several age-groups, and using a variety of different test batteries^16–29^. The majority of secular trends analyses have been focusing only on one component of PF, predominantly on CRF^30–32^, or MF^33–37^ but there is a lack of secular analyses researching other components of PF. Existing secular analyses are also rarely based on population data^38^ and cover a narrow age span of children. The existing evidence, however, suggests that negative changes in secular trends of PF of children have been identified in the previous four decades^23,26,39–46^ with rare exceptions^38^. This decline could be attributed to growing sedentary behaviour, lack of habitual physical activity, and the easy availability of energy-rich food^38^.

Slovenia as Central European country has been facing the challanges of high income countries combating lifestyle changes of population. The annual consumption of chocolate, cocoa, cookies and biscuits that remained below 5 kg per person per year in the 1980s and 1990s, doubled by 2004^47^. This went along with changes in sedentariness due to proliferation of screen technologies in the households. The share of households with cable or Internet Protocol television increased from 11 in the late 1980s to 90% by 2008, while the share of households with internet access increased from 1% in 1995, to 50% in 2010 and exceeded 70% by 2011^48,49^. In the same period, the household ownership of bicycles fell from 68.5 in 1990 to 60.4% in 2000^47^. Consistent with this unfavourable trends, overweight and obesity increased in Slovene children^50^. This growing problem has not been adequately addressed until late 2000s, when several nation-wide initiatives targeting both physical activity and nutrition were introduced within the educational system. Following these interventions a decline in overweight and obesity in Slovene children was observed^50^.

In this paper, we study the secular changes in different components of somatic characteristics, CRF, MF, neuromuscular fitness (NMF), and flexibility for children aged 7–15 for the period 1989–2019 using the population-based data from the SLOfit, the Slovenian national surveillance system for physical fitness (PF) development of school children, which has been used to continuously monitor the PF of Slovene children over the last 3 decades^51^. The goal of the study is threefold: for each test from the SLOfit battery, we aim to (1) estimate the general population trends of multiple components of PF of children during the entire studied period, (2) identify structural changes in these trends evaluating the times at which these changes have occurred as well as their magnitude and finally (3) assess the potential changes in differences (variability) of the test scores between the children.

## Methods

### Study design, subjects and measurements

This is a population-based study using a large data set collected through the SLOfit. The SLOfit test battery incorporates the following anthropometric measurements and fitness tests (see^51^ for more details): body mass index (BMI), triceps skinfold (TSF), 600-m run (R600), 60-s sit-ups (SU60), bent-arm hangs (BAH), stand-and-reach (SAR), standing broad jump (SBJ), backwards obstacle course (BOC), 60-m dash (D60), and 20-s arm plate tapping (APT). The monitoring was implemented in 1982 and after a 6-year testing period became compulsory for all Slovenian schools in the school year 1987/1988. Every April the measurements are conducted by physical education teachers in all Slovenian schools according to the uniform official protocol^51^. During the course of their graduate education, physical education teachers are thoroughly educated in anthropometry with the level of detail that exceeds the demands of the SLOfit system. All the schools in Slovenia are equipped with the required measurement instruments, including medical scales with stadiometers.

After the school-based measurements, the results are sent to the Laboratory for Diagnostics of Somatic and Motor Development at the Faculty of Sport, University of Ljubljana. The main SLOfit administrator uses specially designed software to check the data for logical (univariate) errors, communicates the eventual needs for corrections to teachers, but does not remove the multivariate outliers; the multivariate outliers are removed as described in Supplementary Material.

This study is based on the data from 1989 to 2019. The target population were students from all Slovenian primary schools, aged 7 to 15 years at the time of measurement. Children younger than 7 years old were not considered in the analysis since in the 2002/2003 school year the school system changed, with children enrolling in the primary school 1 year earlier, at the age of six, as opposed to age of seven as in the previous school years. The studied sample did not include children and youth with special needs.

The number of participants over this interval averaged 137,320 individuals per year, leading to a total of 4,256,930 data points during the 31 years (the exact number of participants across the study period by the three age groups—children (7–9-year-olds), early adolescents (10–12-year-olds) and adolescents (13–15-year-olds)—are shown in Supplementary Material—Supplementary Table 1.1). The study included about 95% of the target population.

### Statistical analysis

Each test score was converted to a centile as follows. Smoothed centile curves for the entire period 1989–2019 for all children were obtained using Generalized Additive Models for Location, Scale and Shape (GAMLSS)^52^, separately for boys and girls. Several continuous (Box-Cox Cole and Green (BCCG), Box-Cox power exponential—BCPE and Box-Cox-*t*—BCT distributions were fitted to the data, optimizing the degrees of freedom (DF) for P-splines fit for all parameters of the respective distributions using Schwarz Bayesian criterion (SBC); appropriate link functions were used for the parameters. In all the models 1/2 was used for the power transformation of *age*. The final model for each test and gender was determined by using SBC. The final model based on the best fitting distribution of each test is presented in Supplementary Material. The results of the final models were then used to calculate the centile for each test score by using the estimated cumulative distribution function based on subject’s age and gender. By doing this we obtain age and gender-adjusted percentile ranking of every child in the 1989–2019 period, which enables direct comparison between different tests, genders, and age groups. Note that the value below 1/2 implies that the test score for a given child is worse (APT, SBJ, SU60, BAH, SAR) or better (BOC, D60, R600) than the age and gender-adjusted test scores averaged over the entire study period.

Quantile regression^53^ was used to estimate the changes of PF in time, considering the centile for each test score as a dependent variable and year (either as a categorical covariate, using 1989 as a reference, or as a cubic spline with 5 DF), age (considering 3 age groups: 7–9 years, 10–12 years and 13–15 years), and region as covariates including in the model also the year and age interaction. The following quantiles were considered: 1st decile, 1st quartile, median, 3rd quartile, and 9th decile. The trends observed for the difference of the 3rd and the 1st quartiles (interquartile range—IQR) and for the difference of the 9th and the 1st decile (interdecile range—IDR) were similar, hence we only show the results for the latter. Separate models were fitted for boys and girls. The same analysis was performed also using raw test results as a dependent variable, observing similar trends as when using the centiles (results not shown). Sensitivity analysis using beta regression model^54^ was performed, obtaining very similar results (results not shown).

Segmented regression was used to detect structural changes in secular trends of PF. A segmented (or broken-line) relationship is defined by the slope parameters and the joinpoints where the linear relation changes. We fix the number of joinpoints to 0, 1, 2, and 3 and then use Schwarz information criterion (BIC) to select the optimal number of joinpoints. For each pre-specified number of joinpoints we used the method proposed by Muggeo^55^ to estimate the joinpoint location. Thus, through applying segmented regression analysis, we identified the moment when the change has occurred in the trend, as well as the magnitude of the increase or decrease observed in the interval by estimating annual percent change (APC), defined as $\left(\mathrm{exp}\left({b}_{j}\right)-1\right)\times 100$, where ${b}_{j}$ is the slope in segment *j*. The average APC (AAPC) was calculated as proposed by Clegg et al.^56^. To estimate the joinpoints, a quantile regression model was fitted, modelling year as a continuous independent variable adjusting for region, stratifying the analysis by gender and age group, considering the median. As before we fitted the models using centiles and raw data results as dependent variables, obtaining similar results (results for the latter not shown).

The analysis was performed using R language for statistical computing (R version 3.6.3)^57^; GAMLSS were fitted using R package GAMLSS^58^. Quantile regression models were fitted using the R package QUANTREG^59^; standard errors were obtained using a kernel estimate of the sandwich estimator as proposed by Powell^60^; B-spline basis matrix for the cubic spline was obtained with the R package SPLINES^61^. Beta regression models were fitted using the R package BETAREG^62^. R package SEGMENTED^63^ was used for fitting the segmented regression models and for calculating APC and AAPC. Due to vast amount of data and consequently high computing demands the analysis was performed on clusters of CentOS based containers. These sped up the analysis by a factor of 200; a rough estimate is that the analysis on an ordinary desktop computer would have taken about a year.

### Ethics statement

The studies involving human participants were reviewed and approved by National Medical Ethics Committee of the Republic of Slovenia (ID 102/03/15). Written informed consent to participate in this study was provided by the participants' legal guardian/next of kin. All methods were performed in accordance with the relevant guidelines and regulations.

## Results

**Figure 1:**
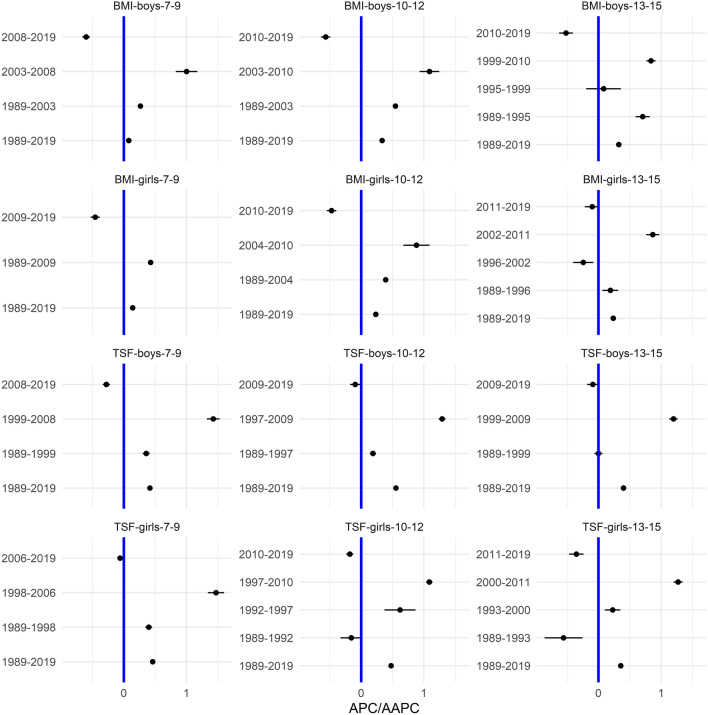
The estimated APC and AAPC (x-axis) with their respective 95% confidence intervals (CI) for each segment as estimated by minimizing the BIC (y-axis) for BMI and TSF by age groups (7–9-year-olds, 10–12-year-olds, and 13–15-year-olds) and gender. Positive (negative) values of APC (AAPC) imply that BMI was increasing (decreasing) during the estimated segment, when the CIs cross the vertical blue line (set at APC (AAPC) equal to zero) this implies that the results did not change during the estimated segment. See Supplementary Information for more results including the uncertainty in the estimated segments.

In the Supplementary Material, we show the smoothed centile curves for the entire studied period which were used to calculate the centiles for each test and gender. There we also show detailed results of the quantile regression models, reporting the estimated coefficients and their respective standard errors for each test (converted to centile ranks) and gender. We also show the estimated position of the joinpoints, APC and AAPC with corresponding 95% confidence intervals (CI) for all tests by gender and age group. In what follows we show inFigs. 2, 3, 5 and 7 the estimated median and the estimated IDR adjusted for the region for the three age groups by year and gender; we show the results when considering year as a categorical predictor—points, circles, and triangles for 7–9-year-olds, 10–12-year-olds and 13–15 year-olds, respectively, and when using cubic splines—solid red lines, solid green lines and solid blue lines for 7–9-year-olds, 10–12-year-olds and 13–15 year-olds, respectively. In Figs. 1, 4, 6 and 8 we show the estimated APC and AAPC (x-axis) with their respective 95% confidence intervals for each segment as estimated by minimizing the BIC (y-axis) for each test by age group and gender, see Supplementary Material for exact results about APC and AAPC. We briefly describe these results for somatic characteristics, MF, NMF, flexibility, and CRF in the following subsections.

### Somatic characteristics

During the entire study period 1989–2019, median BMI and TSF increased in all genders and age groups (Fig. 1). The increase for BMI was the largest in the period 2003–2008, 2003–2010 and 1999–2010 for 7–9 years old, 10–12 years old and 13–15 years old boys and in the period 1989–2009, 2004–2010 and 2002–2011 for 7–9 years old, 10–12 years old and 13–15 years old girls. For TSF the increase was the largest in the period 1999–2008, 1997–2009 and 1999–2009 for 7–9 years old, 10–12 years old and 13–15 years old boys and in the period 1998–2006, 1997–2010 and 2000–2011 for 7–9 years old, 10–12 years old and 13–15 years old girls. In the final period, median BMI and TSF decreased.

**Figure 2:**
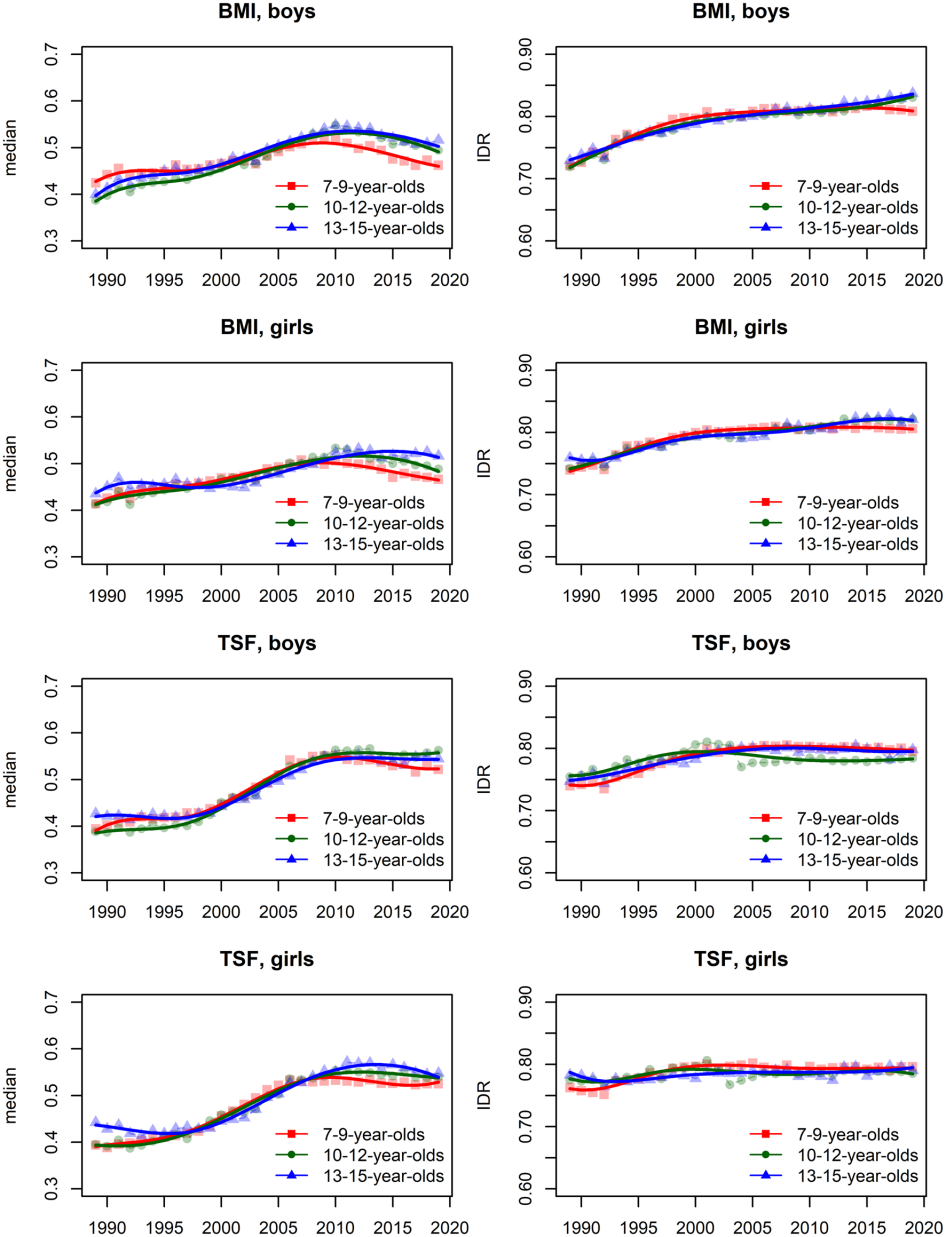
Time trends (medians and IDR) in the BMI and TSF between 1989 and 2019 by gender, across three age groups: 7–9 years, 10–12 years, and 13–15 years. Points and solid lines are the estimated values and smoothed estimated values, respectively, adjusted by region (the results are shown fixing the region to its mode). In the plots showing the estimated medians, the estimates above (below) 1/2 imply that the results were larger (smaller) than in the period 1989–2019 when comparing children of the same gender and age. See Supplementary Information for more results including the uncertainty in the point estimates.

The variability of BMI, as measured by IDR, was increasing during the entire study period for both genders and all age groups. For TSF this was observed for boys up to about 2005 for all age groups, while afterward the variability did not change much. For girls, the variability of TSF did not change substantially (Fig. 2; see Supplementary Material for more details including the results about the other estimated quantiles).

### Muscular fitness

**Figure 3:**
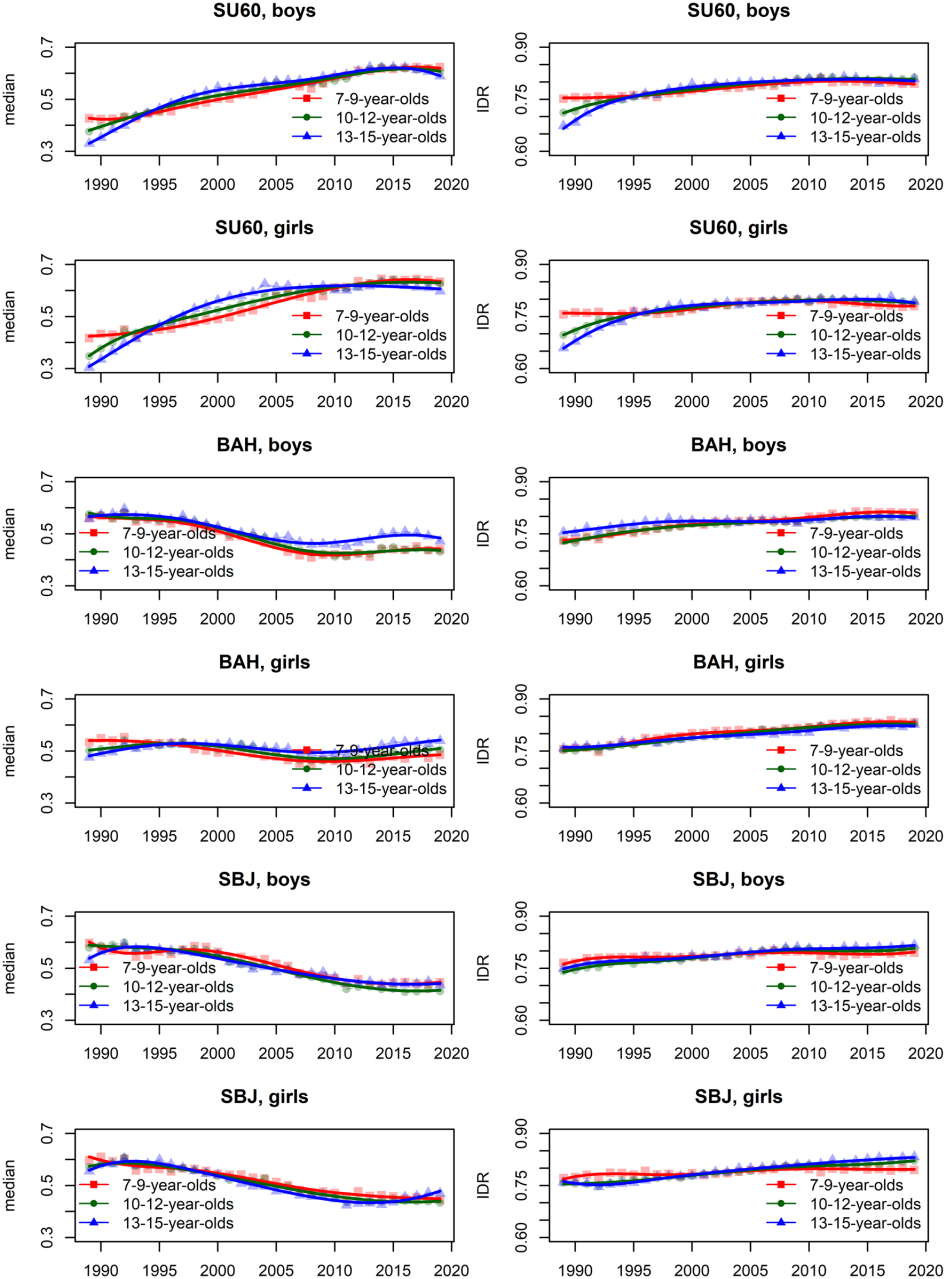
Time trends (medians and IDR) in the SU60, BAH, and SBJ between 1989 and 2019 by gender, across three age groups: 7–9 years, 10–12 years, and 13–15 years. Points and solid lines are the estimated values and smoothed estimated values, respectively, adjusted by region (the results are shown fixing the region to its mode). In the plots showing the estimated medians, the estimates above (below) 1/2 imply that the results were better (worse) than in the period 1989–2019 when comparing children of the same gender and age. See Supplementary Information for more results including the uncertainty in the point estimates.

**Figure 4:**
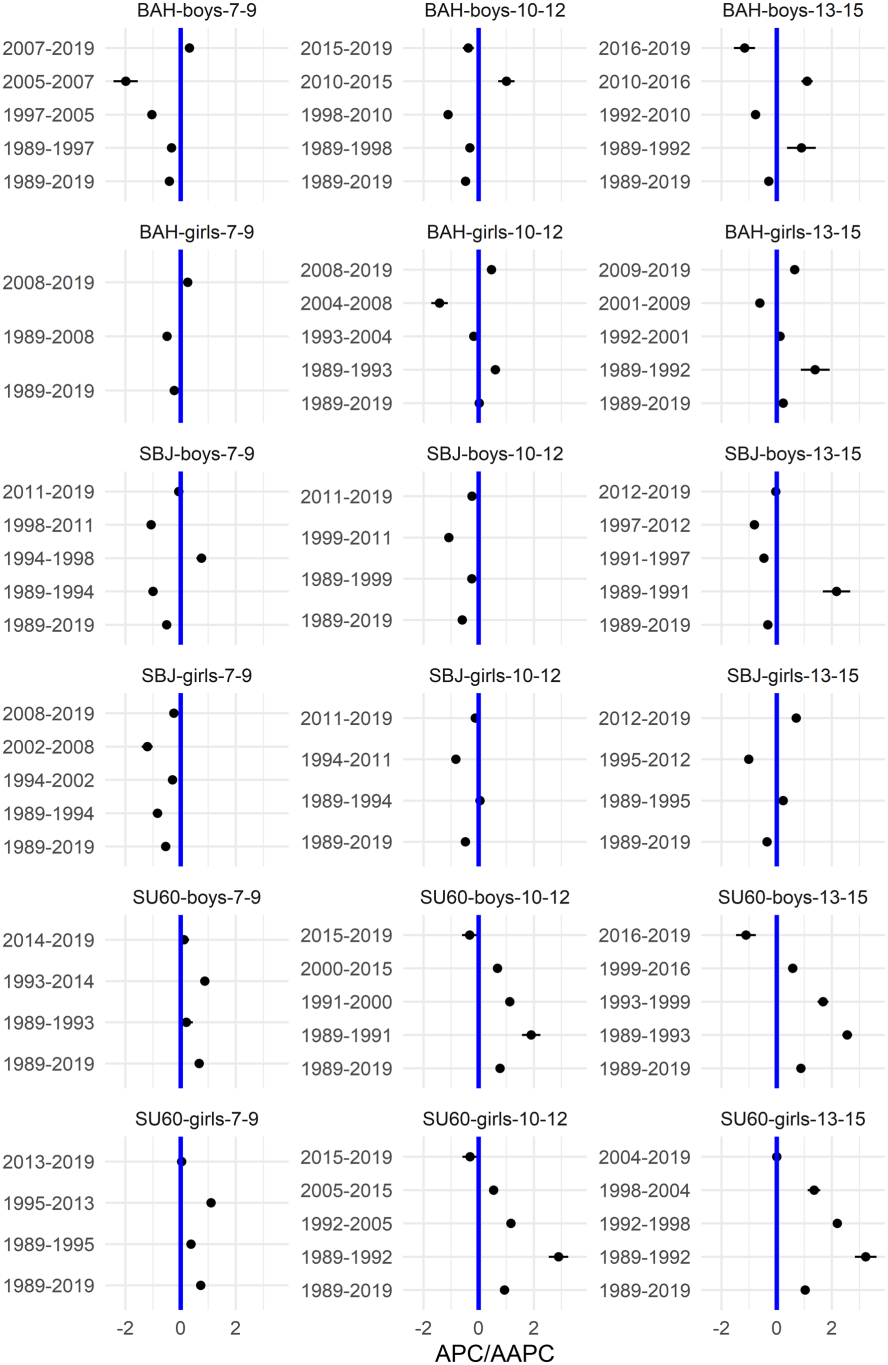
The estimated APC and AAPC (x-axis) with their respective 95% confidence intervals (CI) for each segment as estimated by minimizing the BIC (y-axis) for BAH, SBJ, and SU60 by age groups (7–9-year-olds, 10–12-year-olds, and 13–15-year-olds) and gender. Positive (negative) values of APC (AAPC) imply that the children were improving (worsened) during the estimated segment, when the CIs cross the vertical blue line (set at APC (AAPC) equal to zero) this implies that the results did not change during the estimated segment. See Supplementary Information for more results including the uncertainty in the estimated segments.

During the period 1989–2019 the results of SU60 improved, while on the other hand, the results of BAH and SBJ worsened in both genders and all age groups (Figs. 3, 4). Two exceptions to this overall trend were observed for BAH for girls: in the oldest age group the results improved and they did not change substantially for the 10–12-year-olds.

For SU60 the largest improvement for boys was observed in the period 1993–2004, 1989–1991 and 1989–1993, for 7–9, 10–12 and 13–15 year-olds, respectively, while for girls this was observed in 1995–2013 for 7–9-year-olds and in 1989–1992 for the other two age groups (Fig. 4). This positive trend changed however in the latest period, where the results for 10–12 and 13–15 years old boys and for 10–12 years old girls worsened or did not change substantially for the other groups of children.

The results for BAH worsened for the youngest boys (especially in the period 2005–2007), except in the final period, where a slight improvement was observed. In boys aged 10–12 years, the results worsened (especially in the period 1998–2010), with the exception of the period 2010–2015, where a substantial improvement was observed. For the oldest boys two periods where the results improved were identified (1989–1992 and 2010–2016), but at the same time there were two periods where the results worsened (especially in the latest period from 2016 to 2019. For the youngest girls, the results worsened in the period 1989–2008 but improved in the period 2008–2019. In the two older age groups the results generally improved (especially for girls aged 13–15 in the period 1989–1992), except in the period 2004–2008 for 10–12 year-olds and in the period 2001–2009 for the 13–15 year-olds where the results worsened.

The results for SBJ either worsened in all the identified periods or did not change much (especially in the latest periods) with some exceptions where the results improved: 7–9 years old boys in the period 1994–1998, 13–15 years old boys in the period 1998–1991 and the oldest girls in the period 2012–2019 (Fig. 4).

The variability of all tests increased during the period 1989–2019 for both genders and all age groups. For SU60 in the two older age groups, a large increase was observed up to about 1995 for both genders, afterward the increase in variability was smaller. For the other tests/age groups, the increase in variability was fairly constant across the entire studied period (Fig. 3).

### Neuromuscular fitness

**Figure 5:**
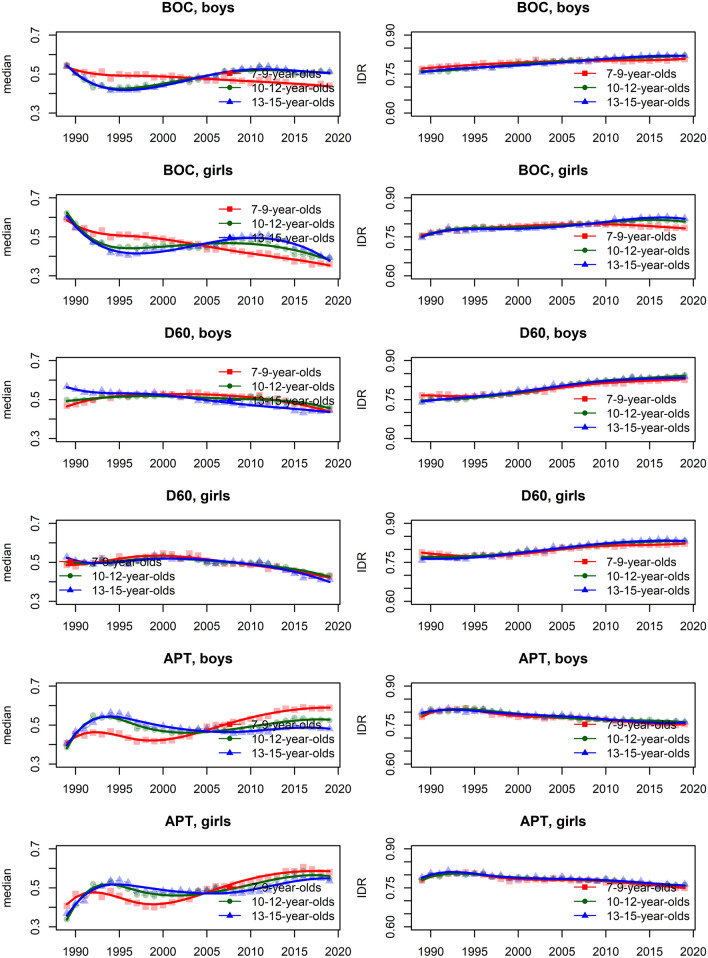
Time trends (medians and IDR) in the BOC, D60, and APT between 1989 and 2019 by gender, across three age groups: 7–9 years, 10–12 years, and 13–15 years. Points and solid lines are the estimated values and smoothed estimated values, respectively, adjusted by region (the results are shown fixing the region to its mode). In the plots for BOC and D60 showing the estimated medians, the estimates below (above) 1/2 imply that the results were better (worse) than in the period 1989–2019 when comparing children of the same gender and age; for APT the opposite holds. See Supplementary Information for more results including the uncertainty in the point estimates.

**Figure 6:**
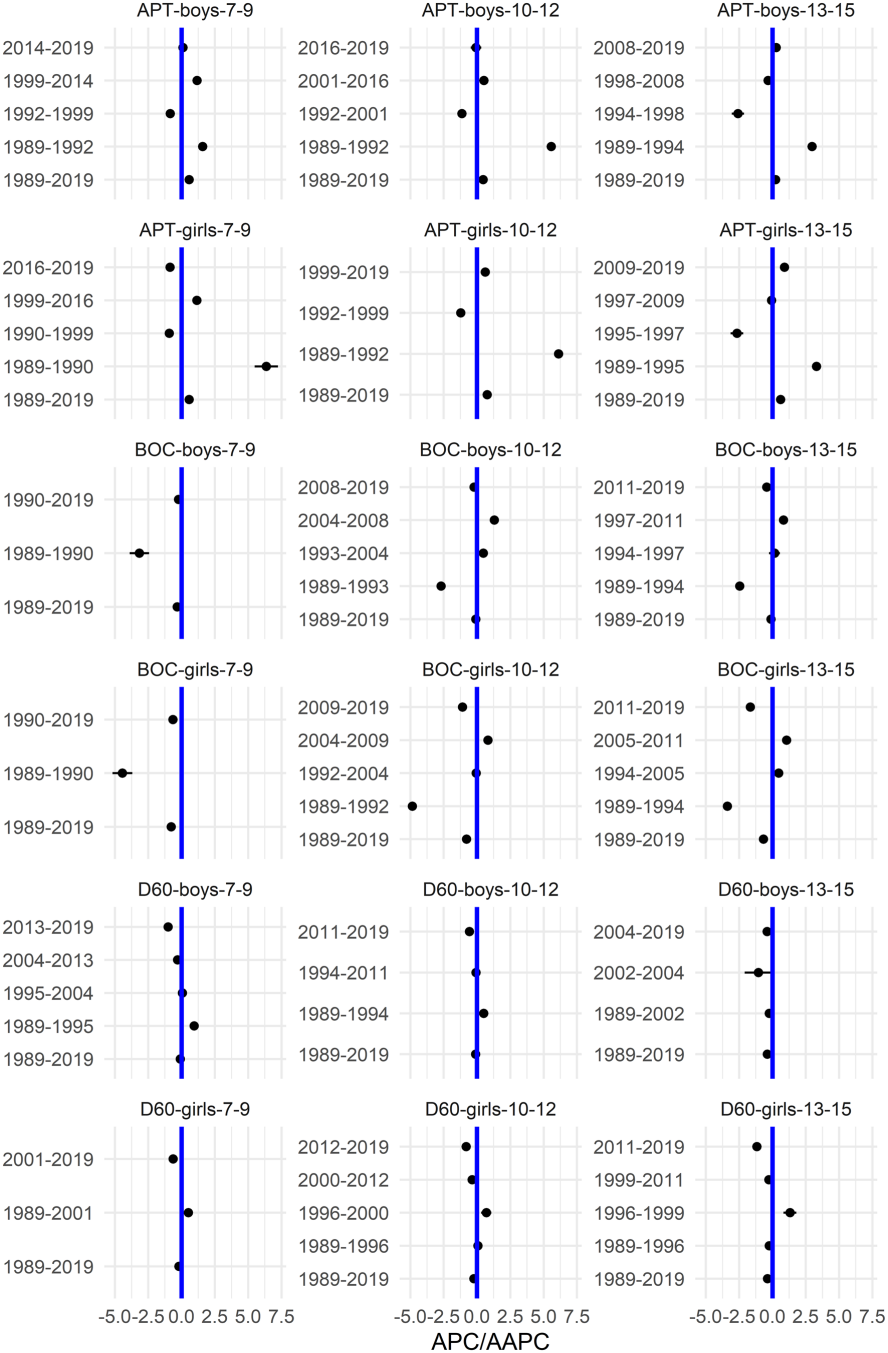
The estimated APC and AAPC (x-axis) with their respective 95% confidence intervals (CI) for each segment as estimated by minimizing the BIC (y-axis) for APT, BOC, and D60 by age groups (7–9-year-olds, 10–12-year-olds, and 13–15-year-olds) and gender. Negative (positive) values of APC (AAPC) for BOC and D60 imply that the children were improving (worsened) during the estimated segment (reverse holds for APT), when the CIs cross the vertical blue line (set at APC (AAPC) equal to zero) this implies that results did not change during the estimated segment. See Supplementary Information for more results including the uncertainty in the estimated segments.

Averaged over the entire study period the results for BOC, D60, and APT slightly improved, more so for girls than for boys (Figs. 5, 6).

For BOC the improvement was large in the early period (1989–1990, 1989–1993, and 1989–1994 for boys aged 7–9, 10–12, and 13–15, respectively; 1989–1990, 1989–1992, and 1989–1994 for girls aged 7–9, 10–12, and 13–15, respectively), whereas in the later period this improvement was much smaller or the results even worsened for the two older age groups (especially in the period 2004–2008 and 1997–2011 for 10–12 and 13–15 years old boys, respectively and in the period 2004–2009 and 2005–2011 for 10–12 and 13–15 years old girls; Fig. 6).

The results for D60 in general improved in most of the identified periods (especially in the period 2013–2019, 2011–2019 and 2002–2004 for 7–9, 10–12 and 13–15 years old boys and in the period 2001–2019, 2012–2019 and 2011–2019 for 7–9, 10–12 and 13–15 years old girls) but there were some exceptions where the results worsened, especially for girls aged 10–12 and 13–15 in the period 1996–2000 and 1996–1999, respectively (Fig. 6).

The results for APT improved in most of the identified periods, especially during the early period (1989–1992, 1989–1992 and 1989–1994 for 7–9, 10–12 and 13–15 years old boys, respectively and 1989–1990, 1989–1992 and 1989–1995 for 7–9, 10–12 and 13–15 years old girls, respectively) but there were also periods where the results did not change much (2014–2019, 2016–2019, 1998–2019 and 1997–2009 for 7–9, 10–12 and 13–15 years old boys and for 13–15 years old girls, respectively) or even worsened (especially during 1992–1999, 1992–2001, 1994–1998, 1990–1999, 1992–1999 and 1995–1997 for 7–9, 10–12 and 13–15 years old boys and for 7–9, 10–12 and 13–15 years old girls, respectively; Fig. 6).

The variability of BOC and D60 was increasing during the study period in both genders and all age groups. For BOC this trend changed for the youngest boys and girls where after about 2005 the variability either stabilized (boys) or even started to decrease (girls). For APT the opposite was observed with the variability decreasing slightly during the entire study period (Fig. 5).

### Cardiorespiratory fitness

**Figure 7:**
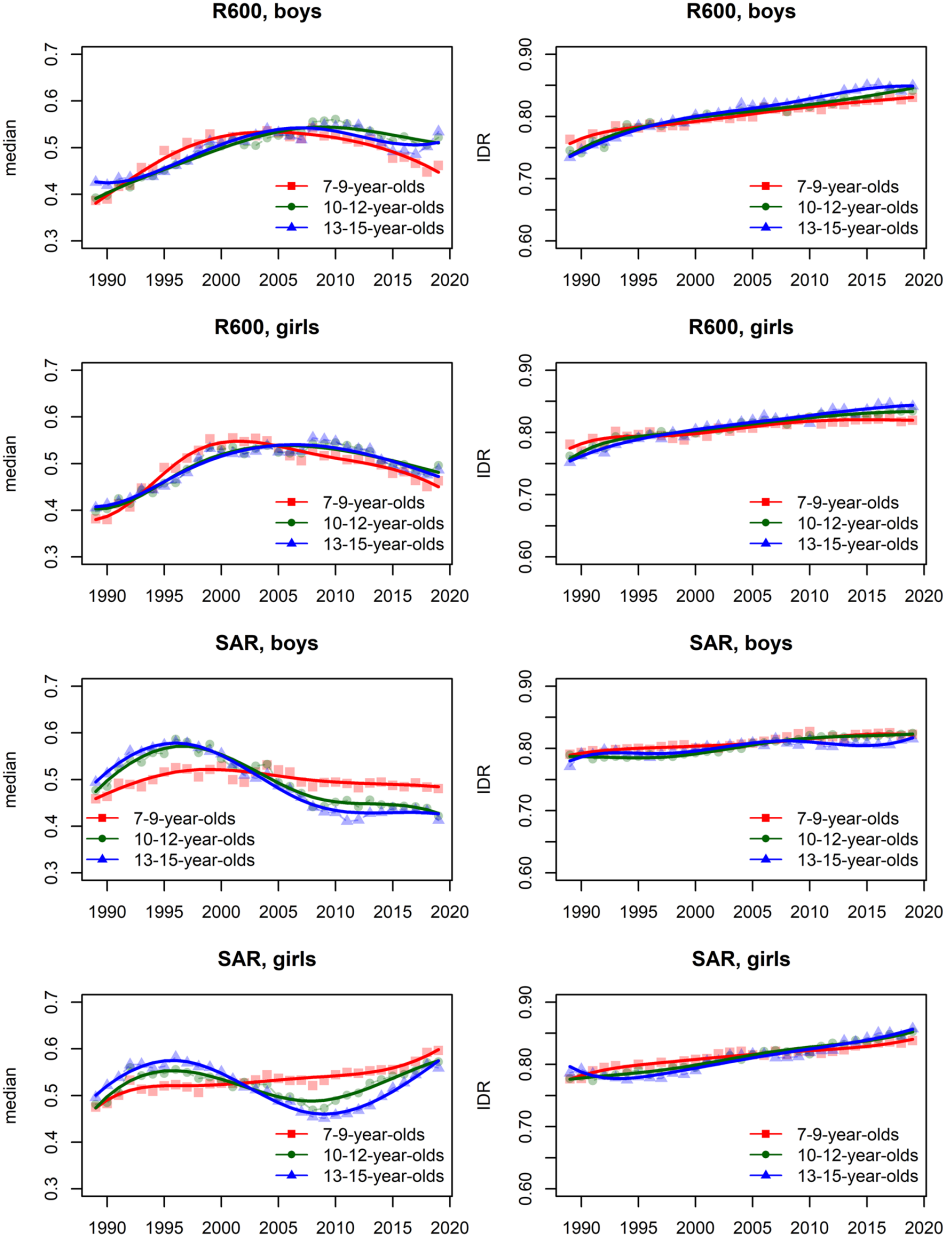
Time trends (medians and IDR) in the R600 and SAR between 1989 and 2019 by gender, across three age groups: 7–9 years, 10–12 years, and 13–15 years. Points and solid lines are the estimated values and smoothed estimated values, respectively, adjusted by region (the results are shown fixing the region to its mode). In the plots for R600 showing the estimated medians, the estimates below (above) 1/2 imply that the results were better (worse) than in the period 1989–2019 when comparing children of the same gender and age; for SAR the opposite holds. See Supplementary Information for more results including the uncertainty in the point estimates.

**Figure 8:**
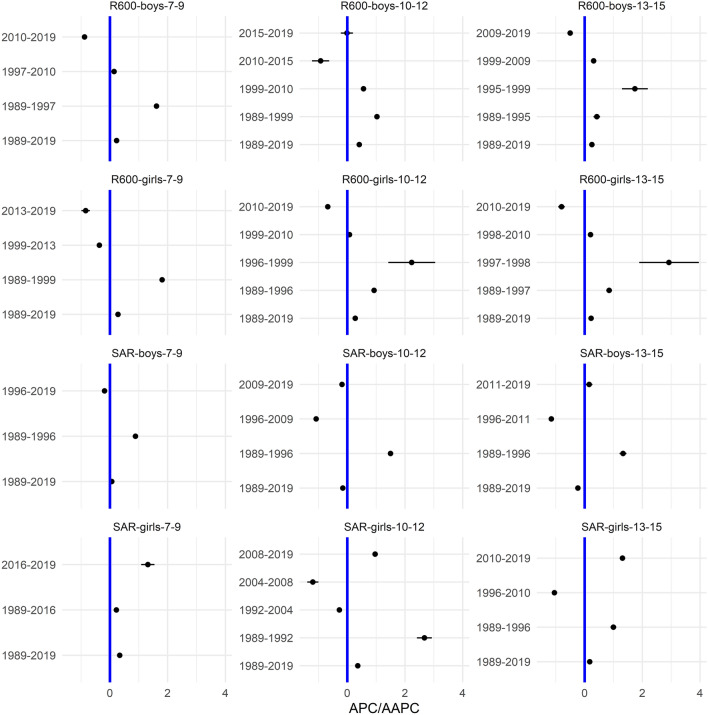
The estimated APC and AAPC (x-axis) with their respective 95% confidence intervals (CI) for each segment as estimated by minimizing the BIC (y-axis) for R600 and SAR by age groups (7–9-year-olds, 10–12-year-olds, and 13–15-year-olds) and gender. Negative (positive) values of APC (AAPC) for R600 imply that the children were improving (worsened) during the estimated segment (for SAR the reverse holds), when the CIs cross the vertical blue line (set at APC (AAPC) equal to zero) this implies that the results did not change during the estimated segment. See Supplementary Information for more results including the uncertainty in the estimated segments.

Averaged over the entire study period the results for R600 worsened for both genders and all age groups (Figs. 7, 8).

The results for boys worsened in all the identified periods, the exceptions were periods 2010–2019 for the youngest boys, 2010–2015 for 10–12-year-olds and 2009–2019 for 13–15-year-olds where the results improved and 2015–2019 for 10–12-year-olds where the results did not change (Fig. 8). For girls, the results worsened in most periods (especially during 1989–1999, 1996–1999, and 1997–1998 for the 7–9, 10–12 and 13–15-year-olds). The exceptions were during 1999–2013 and 2013–2019 for the 7–9-year-olds, 2010–2019 for the 10–12 and 13–15-year-olds where the results worsened.

The variability was increasing during the entire study period for both genders, more so for the two older age groups (Fig. 7). For the youngest girls, the variability plateaued after about 2015.

### Flexibility

The results for SAR for boys either worsened (two older age groups) or did not change substantially (the youngest group of children). For girls, the results improved slightly for all age groups (Figs. 7, 8).

For 7–9 and 10–12 years old boys, SAR improved in the initial period (1989–1996) but then the results worsened in the later periods, especially for 10–12-year-olds during 1996–2009. A similar trend as for 10–12-year-olds was observed also for the 13–15-year-olds with the exception that here the results improved slightly in the latest identified period from 2011 to 2019. For the youngest girls, the results improved in both periods, more so in the later period, from 2016 to 2019. For the other two age groups, the improvement in the earlier period (1989–1992 and 1989–1996 for 10–12 and 13–15-year-olds, respectively) was followed by the worsened results in the following periods (for 10–12-year-olds especially during 2004–2008 and for the 13–15-year-olds during 1996–2010) and finally improved results again the latest period (2008–2019 for 10–12-year-olds, 2010–2019 for 13–15-year-olds; Fig. 8).

The variability, in general, increased during the entire study period, more so for girls than for boys.

## Discussion

In this work we aimed to estimate the general secular trends of multiple PF components during the last three decades, to identify structural changes in these trends, and to establish whether the differences within population groups have been increasing.

Our results suggest that from the mid-1990s until 2010 the level of CRF, MF, NMF, and flexibility was declining, but that in the following period until 2019 the trends generally reversed. However, the differences among the fittest and the least fit children continued to increase also in the periods when physical fitness was generally improving.

The largest increases of BMI and TSF in boys and girls of all age groups occurred in the period between 2000 and 2010—following less pronounced increases in previous periods—but the overall increase of BMI in this period was lower than the increase of TSF. In the last decade, however, the trends of BMI and TSF mostly reversed, but BMI decreased more than TSF. In this period the difference between both extreme deciles in BMI increased while in TSF it remained stable. The existing evidence on the Slovenian population suggests^50^, that after 2010 the prevalence of childhood obesity started decreasing, and our findings are providing additional evidence that this observed decline in the last decade—based on decreased BMI—might be attributed more to loss of lean rather than fat mass.

Although the latest evidence on the trends of general PF among Slovenian children in the last decade^38^ is positive, the trends in different PF components are not uniform but are, nevertheless, in agreement with our observed trends in BMI and TSF.

Namely, the suggested larger loss of lean body mass is especially evident in explosive power and isometric strength tasks where maximum muscle exertion is required, in our case in BAH and SBJ, both serving as indicators of MF. We were unable to identify any published evidence on the secular trends of BAH in children aged 7–9, but the latest systematic reviews^64–66^ show that in children aged 10 and above, predominantly negative trends have been observed in the last decades. Our evidence agrees with these findings regarding the pre-2010 periods but not with the post-2010 period in which children in Slovenia were mostly experiencing the improvement of isometric upper limb strength, with exception of the oldest group of boys who experienced a considerable decline also between 2016 and 2019. The variability of BAH results, nevertheless, was increasing the entire observed period 1989–2019.

In SBJ the most pronounced negative trends in Slovenian children occurred in the decade before 2012 while afterward the results stagnated across all age groups of boys and girls with exception of girls aged between 13 and 15 who experienced positive trends in this period. Our findings agree with the large majority of studies on secular trends of explosive strength of lower limbs and legs which reported the negative trends^64,65^ but it agrees also with a few studies who identified positive trends in certain age groups and periods^42,67,68^. Namely, also in Slovenian children periods with positive trends in boys (1994–1998 in 7–9 year-olds and 1989–1991 in 13–15 year-olds) and girls (1989–1994 in 10–12 year-olds, or 1989–1995 and 2012–2019 in 13–15 year-olds) were identified, although the overall secular trend between 1989 and 2019 was negative. The variability of results in boys and girls aged 10 or more was increasing the entire observed period, while the youngest 7–9 year-olds experienced stagnation after 2010.

Although the SU60 results have been generally increasing from 1989 onwards, the trend was decelerating towards the latest periods in which the trends either stagnated in the youngest age group or declined in the older ones. These results are contrasting the trends in majority of other studies of secular trends in core strength which identified exclusively negative trends^36,67,69–72^ In a few studies, however, similar positive trends have been identified in some periods—typically after the mid 1990s—in Portugal, Greek, Chinese, Finland, and New Zealand children^25,29,42,73,74^ Variability in core strength was increasing until 2010 but started stagnating or decreasing afterward.

Overall, the components of NMF showed positive trends. The results in BOC were improving from 1989 to 2019, although the improvement was more expressed in girls than in boys. The most pronounced improvements in the oldest two age groups occurred before 1995 and were afterwards followed by a declining trend until 2010 when the trends started improving again. In the youngest age group coordination was mildly improving throughout the entire 1989–2019 period but was more pronounced in girls. In 7–9 year-old girls, variability in BOC results started declining after 2010 while in older age groups of girls variability declined after 2015. In boys, variability was increasing throughout the entire observed period and was less expressed in the youngest age group. We were unable to identify any published evidence on secular trends of BOC but the only comparable existing research of secular trends in coordination based on balance performance showed the improvement in the 1992–2012 period among Lithuanian 11–18 year-olds^26^.

The D60 results were moderately improving in almost all periods and all age groups, except in the pre-1995 period in the youngest age-groups of boys, between 1989 and 2001 in the youngest age-group of girls and from the mid-1990s to the break of millennium in the older age groups of girls. The largest increases occurred after 2010 except in the oldest group of boys who experienced the largest improvement between 2002 and 2004. The variability in sprinting speed in all age groups of boys and girls was increasing throughout the 1989–2019 period. Fühner et al.^66^ similarly showed that speed in children has been rising since 2002 when it bottomed out after the decreasing trend throughout the 1990s and also identified the inflection point around the year 2000. Also, other systematic reviews report mostly positive trends in speed but do point out a few studies with negative trends as well^64,65^.

In APT the largest increases occurred before 1995 but the speed of alternate hand moves has been generally modestly improving throughout the 1989–2019 period. In the last decade, boys experienced very small improvements in the oldest and the youngest age-group but improvement of girls’ results in the oldest two age-groups was more pronounced. In the youngest age-groups of boys and girls the results bottomed-up around the year 2000 while in the older two age-groups this occurred around five years later. Speed of alternate hand moves was the only motor ability with decreasing variability almost throughout the entire 1989–2019 period. We were only able to identify one study of secular trends in APT of Flemish adolescents in the period 1969–2005^41^ which revealed the opposite, worsening trend of the speed of alternate hand moves.

The R600 results have been declining in all age groups until the last decade, with the largest decreases occurring before the year 2000. In the last decade, however, the trends in cardiorespiratory endurance reversed, although the overall trend in the period 1989–2019 remained negative. The youngest group of girls experienced somewhat different trend pattern since their R600 results bottomed up around the year 2000, which was around a decade earlier than in other age groups. Secular trends in cardiorespiratory fitness are the most studied and although two systematic reviews identified mostly declining trends^64,65^, Fühner et al.^66^ identified stabilisation and possible improvement after 2010 which was confirmed in our case. The variability of results in Slovenian children continued to increase also after 2010 with exception of the youngest group of girls who experienced plateauing in this period.

The trends in SAR revealed the largest differences between boys and girls with boys experiencing mostly negative and girls mostly positive trends in the 1989–2019 period. The largest declines in flexibility in boys and girls occurred between 1996 and 2011 in the oldest two age groups, while in the youngest age groups the exclusively improving trends were evident in girls in all periods as well as in boys before 1996. Afterward, boys results steadily deteriorated and reached the overall lowest point in 2019 when—in contrast—girls experienced the highest observed flexibility in the entire 1989–2019 period. In girls, the variability of results in SAR was increasing at a more pronounced pace than in boys. In most published studies on secular trends in flexibility, mostly negative trends have been observed^25,26,41,69,75^, although in some cases, positive trends in boys were identified before 2007 and in girls also in later periods^24,28,42,74^.

In comparison to existing evidence, our research did not provide only the report on secular trends of PF performance but also on the secular trends of the variability of performance which brings an important insight into the actual inequality trends in the population. No other published research was able to use the three decades of annually gathered population data on multiple PF components so far, and our results thus give room to possible future analyses of various environmental changes on PF trends. Due to a large volume of data and consequent computing demands, a supercomputer was used to run the complex analyses which gives additional strength to the results of the study.

Our results are consistent with a number of existing studies but due to the recency of our data supplement the existing evidence. They provide exciting new possibilities for future research since they provide the context required to determine how the changes in children’s development have been related to the wider lifestyle changes in this population and its subgroups in certain periods and, therefore, enables the prediction of future outcomes. Our study shows that despite the unfavorable lifestyles of contemporary children it is still possible to ensure the maintenance or improvement of multiple components of their PF. It also reinforces the notion that trends of childrens’ development should not be looked only at the mean or median level: looking at the differences between the children is as if not even more important. The increasing variance in the majority of PF indicators throughout the 1989–2019 period suggests that—despite the favorable trends in the last decade—children in Slovenia have been facing increasing inequalities in their development which can potentially lead also to future inequalities in health.

## Supplementary Information

Supplementary Information.

## Data Availability

The datasets generated during and/or analysed during the current study are not publicly available due to violating confidentiality but are available from the corresponding author on reasonable request.
