# Supplementary material for: Secular trends in physical fitness of Slovenian boys and girls aged 7 to 15 years from 1989 to 2019: a population-based study
Source: Sci Rep. 2022 Jun 21;12:10495. doi: 10.1038/s41598-022-14813-7 (PMC9213534; doi:10.1038/s41598-022-14813-7)
Supplement: Supplementary file 1 — Supplementary Information. [file 41598_2022_14813_MOESM1_ESM.docx]

Secular trends in physical fitness of Slovenian boys and girls aged 7 to 15 years from 1989 to 2019: A population-based study

Supplementary Material

Ana Radulović^4^, Gregor Jurak^2^, Bojan Leskošek^2^, Gregor Starc^2^, Rok Blagus*^1,2,3^

*corresponding author

^1^ Institute for Biostatistics and Medical Informatics, Faculty of Medicine, University of Ljubljana, Vrazov trg 2, 1000 Ljubljana, Slovenia; [rok.blagus@mf.uni-lj.si](mailto:rok.blagus@mf.uni-lj.si)

^2^ Faculty of Sports, University of Ljubljana, Gortanova ulica 22, 1000 Ljubljana, Slovenia

^3^ Faculty of Mathematics, Natural Sciences and Information Technologies, University of Primorska, Glagoljaška ulica 8, 6000 Koper, Slovenia

^4^ Institute of Public Health of Montenegro, Center for Control and Prevention of Infectious Diseases, Džona Džeksona bb, 81000 Podgorica, Montenegro

Contents

[1. Details about the data cleaning procedure 2](#_Toc89948604)

[2. Optimal GAMLSS models and centile curves 3](#_Toc89948605)

[3.Results of quantile regression models 8](#_Toc89948606)

[4. Results of joinpoint (segmented) regression analysis 66](#_Toc89948607)

# 1. Details about the data cleaning procedure and sample size by year

#
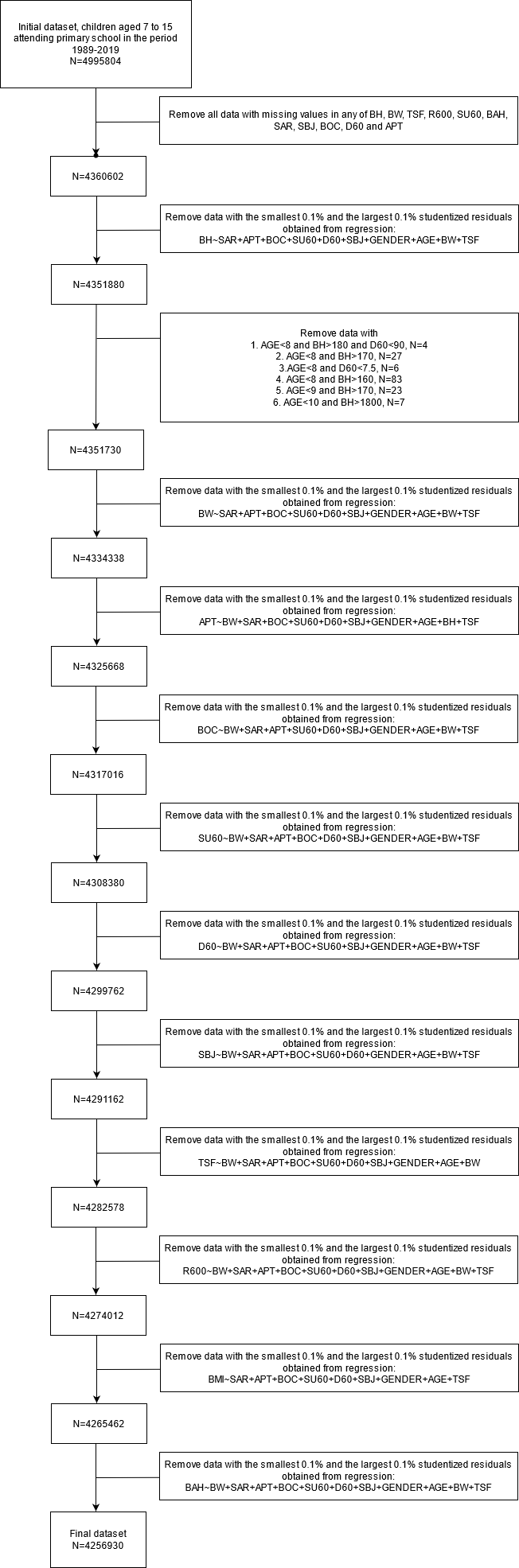


*Supplementary Table 1.1: Number of participants across study years.*

|  | 7-9-year-olds | | 10-12-year-olds | | 13-15-year-olds | |  |
| --- | --- | --- | --- | --- | --- | --- | --- |
| Year | Boys | Girls | Boys | Girls | Boys | Girls | Total |
| 1989 | 27673 | 26086 | 24272 | 22910 | 13925 | 12809 | 127675 |
| 1990 | 28293 | 26791 | 33933 | 31115 | 21234 | 19861 | 161227 |
| 1991 | 27585 | 26249 | 33794 | 31480 | 21582 | 20148 | 160838 |
| 1992 | 27545 | 26610 | 33740 | 31922 | 21949 | 20435 | 162201 |
| 1993 | 29070 | 27976 | 34634 | 32890 | 23301 | 21827 | 169698 |
| 1994 | 29159 | 28070 | 34567 | 32681 | 23603 | 22186 | 170266 |
| 1995 | 28747 | 27679 | 33619 | 31796 | 23075 | 21745 | 166661 |
| 1996 | 29048 | 27852 | 33285 | 31757 | 22207 | 20996 | 165145 |
| 1997 | 27953 | 25848 | 31138 | 29167 | 20448 | 18914 | 153468 |
| 1998 | 26822 | 25086 | 31115 | 29274 | 20080 | 18714 | 151091 |
| 1999 | 25979 | 23892 | 30984 | 29035 | 19578 | 18173 | 147641 |
| 2000 | 25103 | 23364 | 30744 | 28495 | 18966 | 17682 | 144354 |
| 2001 | 23835 | 22552 | 29189 | 27391 | 19150 | 18337 | 140454 |
| 2002 | 23948 | 22349 | 28059 | 26317 | 19420 | 18039 | 138132 |
| 2003 | 24253 | 22682 | 27187 | 25393 | 18560 | 16980 | 135055 |
| 2004 | 24141 | 22434 | 25385 | 23735 | 17420 | 15884 | 128999 |
| 2005 | 24020 | 22576 | 25238 | 23584 | 16941 | 15724 | 128083 |
| 2006 | 23109 | 21447 | 23955 | 22506 | 15829 | 14573 | 121419 |
| 2007 | 22681 | 21027 | 23260 | 21876 | 15233 | 14104 | 118181 |
| 2008 | 22786 | 21122 | 23021 | 21297 | 14953 | 13867 | 117046 |
| 2009 | 23422 | 21544 | 23274 | 21763 | 14893 | 13703 | 118599 |
| 2010 | 22826 | 21393 | 22921 | 21115 | 14693 | 13440 | 116388 |
| 2011 | 22892 | 21591 | 23184 | 21606 | 14520 | 13661 | 117454 |
| 2012 | 23180 | 21683 | 23196 | 21585 | 14381 | 13146 | 117171 |
| 2013 | 23600 | 22178 | 22819 | 21501 | 14099 | 12764 | 116961 |
| 2014 | 24772 | 23242 | 23012 | 21625 | 14554 | 13392 | 120597 |
| 2015 | 25532 | 24067 | 22922 | 21706 | 14373 | 13210 | 121810 |
| 2016 | 27282 | 25968 | 23532 | 22009 | 14466 | 13027 | 126284 |
| 2017 | 28422 | 26979 | 24305 | 22953 | 14202 | 13037 | 129898 |
| 2018 | 28875 | 27238 | 25017 | 23426 | 14262 | 13110 | 131928 |
| 2019 | 28409 | 26606 | 25930 | 24459 | 14206 | 12596 | 132206 |
| Total | 800962 | 754181 | 851231 | 798369 | 546103 | 506084 | 4256930 |

# 2. Optimal GAMLSS models and centile curves

Supplementary Table 2.1: Chosen distribution for each anthropometric and physical fitness measure with the optimal DFs for the P-splines fit for all parameters of the respective distributions and Schwarz Bayesian criterion (SBC) by gender.

| Test | Gender | Distribution | $\mu$ (log link) | $\sigma$ (log link) | $\tau$ (log link) | $\nu$ (identity link | SBC |
| --- | --- | --- | --- | --- | --- | --- | --- |
| BMI | boys | BCPE | 19.73 | 6.73 | 4.59 | 5.66 | 10562105 |
|  | girls | BCPE | 8.08 | 9.99 | 4.80 | 5.57 | 9917224 |
| TSF | boys | BCPE | 10.46 | 23.00 | 4.50 | 5.73 | 13107721 |
|  | girls | BCT | 12.48 | 7.43 | 5.49 | 5.69 | 12331624 |
| BOC | boys | BCPE | 19.25 | 5.70 | 5.14 | 4.12 | 21633174 |
|  | girls | BCPE | 22.53 | 5.37 | 3.84 | 3.13 | 20595707 |
| APT | boys | BCT | 20.09 | 16.72 | 3.98 | 2.05 | 12974617 |
|  | girls | BCT | 20.38 | 17.29 | 3.93 | 2.04 | 12038916 |
| SBJ | boys | BCPE | 22.29 | 9.02 | 6.80 | 2.61 | 19580893 |
|  | girls | BCT | 22.20 | 4.05 | 2.09 | 2.00 | 18030366 |
| SU60 | boys | BCT | 19.74 | 6.22 | 4.24 | 8.51 | 15733293 |
|  | girls | BCT | 22.63 | 6.14 | 4.95 | 5.32 | 14537544 |
| BAH | boys | BCPE | 16.83 | 8.22 | 6.41 | 3.82 | 19701075 |
|  | girls | BCT | 15.16 | 7.96 | 4.90 | 3.49 | 17590409 |
| SAR | boys | BCPE | 17.31 | 6.43 | 5.25 | 3.13 | 14679469 |
|  | girls | BCPE | 17.62 | 5.58 | 3.93 | 2.49 | 13533087 |
| D60 | boys | BCPE | 21.80 | 9.32 | 7.33 | 4.51 | 16155917 |
|  | girls | BCT | 19.34 | 4.71 | 2.04 | 2.08 | 15025723 |
| R600 | boys | BCPE | 22.23 | 6.43 | 4.92 | 2.49 | 20397910 |
|  | girls | BCT | 21.78 | 5.34 | 4.46 | 2.00 | 19256957 |

Supplementary Figure 2.1. Smoothed reference curves for the 1^st^, 3^rd^, 10^th^, 25^th^, 50^th^, 75^th^, 90^th^, 97^th,^ and 99^th^ centiles of BMI and TSF in 7- to 15-year-old Slovenian boys and girls for the period 1989-2019.


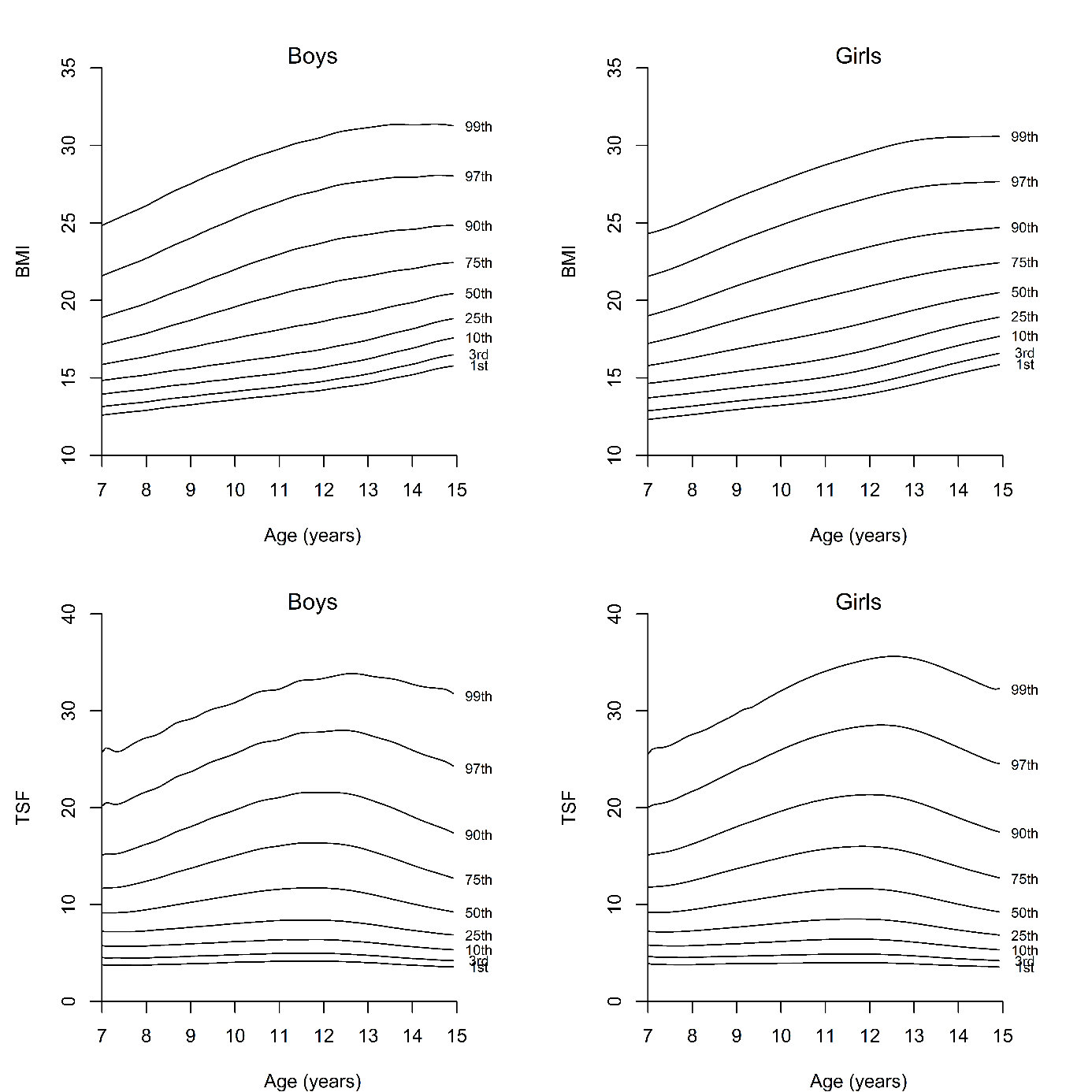


Supplementary Figure 2.2. Smoothed reference curves for the 1^st^, 3^rd^, 10^th^, 25^th^, 50^th^, 75^th^, 90^th^, 97^th,^ and 99^th^ centiles of SBJ, SU60 and BAH in 7- to 15 -year-old Slovenian boys and girls for the period 1989-2019.


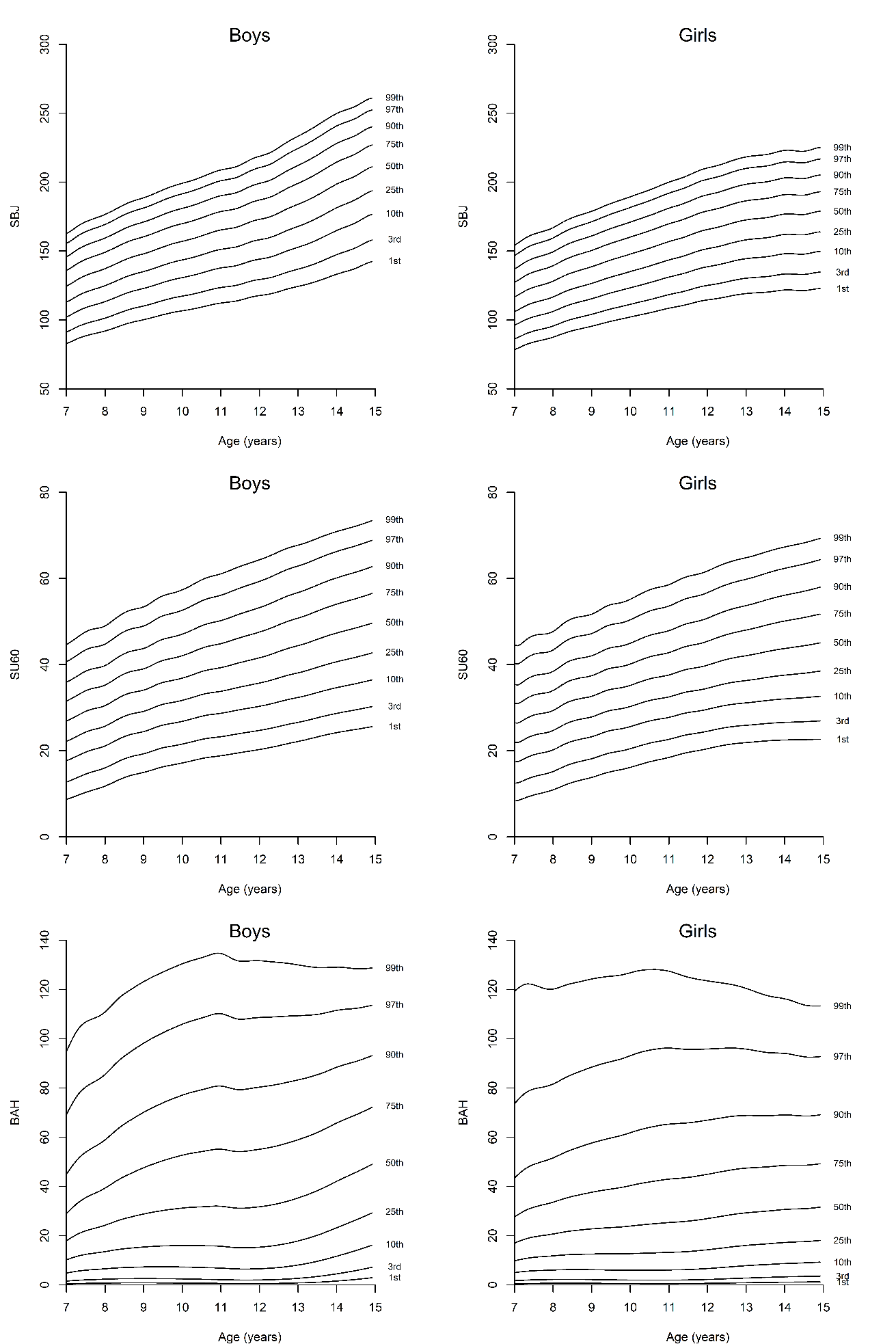


Supplementary Figure 2.3. Smoothed reference curves for the 1^st^, 3^rd^, 10^th^, 25^th^, 50^th^, 75^th^, 90^th^, 97^th,^ and 99^th^ centiles of D60, BOC and APT in 7- to 15 -year-old Slovenian boys and girls for the period 1989-2019.


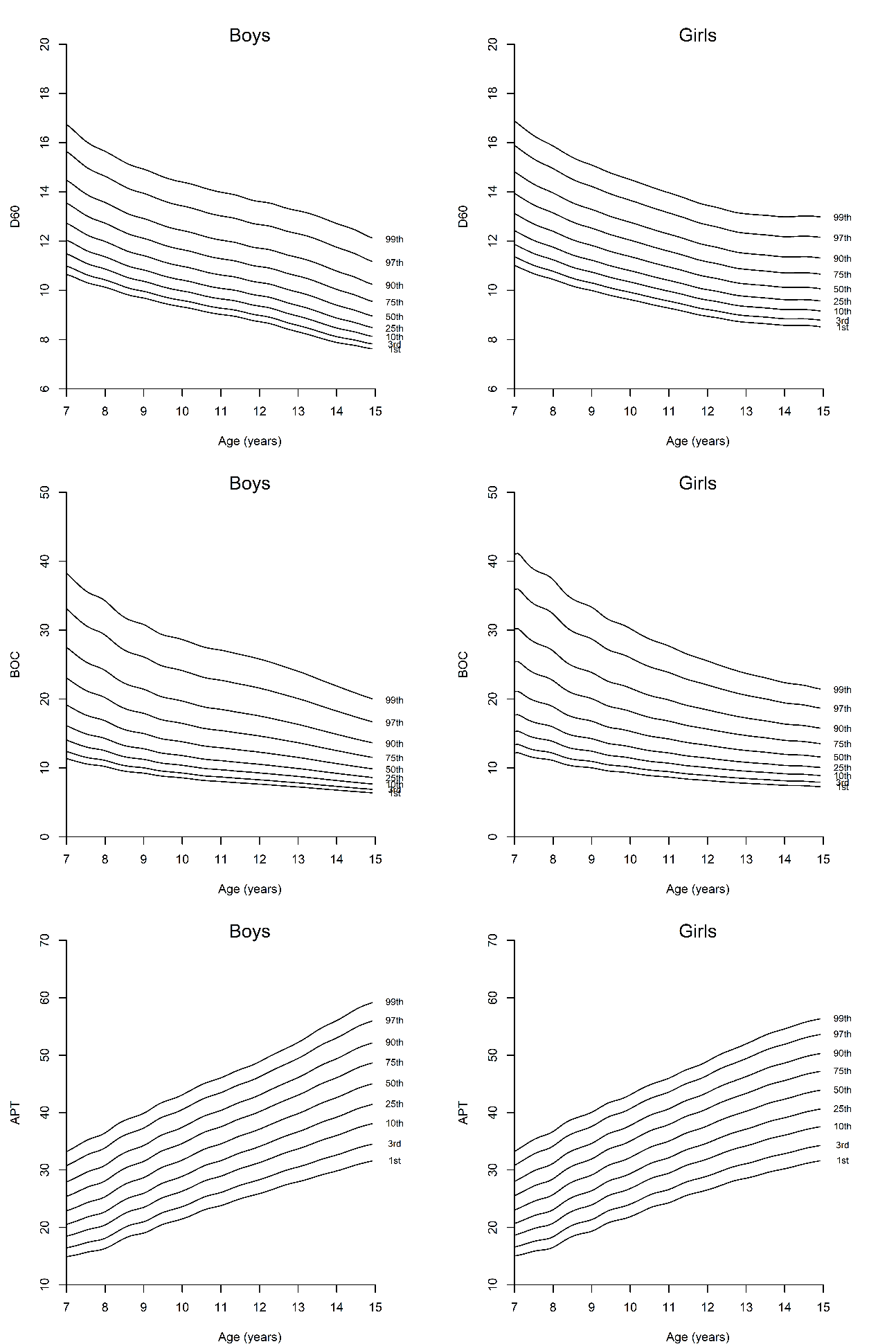


Supplementary Figure 2.4. Smoothed reference curves for the 1^st^, 3^rd^, 10^th^, 25^th^, 50^th^, 75^th^, 90^th^, 97^th,^ and 99^th^ centiles of R600 and SAR in 7- to 15-year-old Slovenian boys and girls for the period 1989-2019.


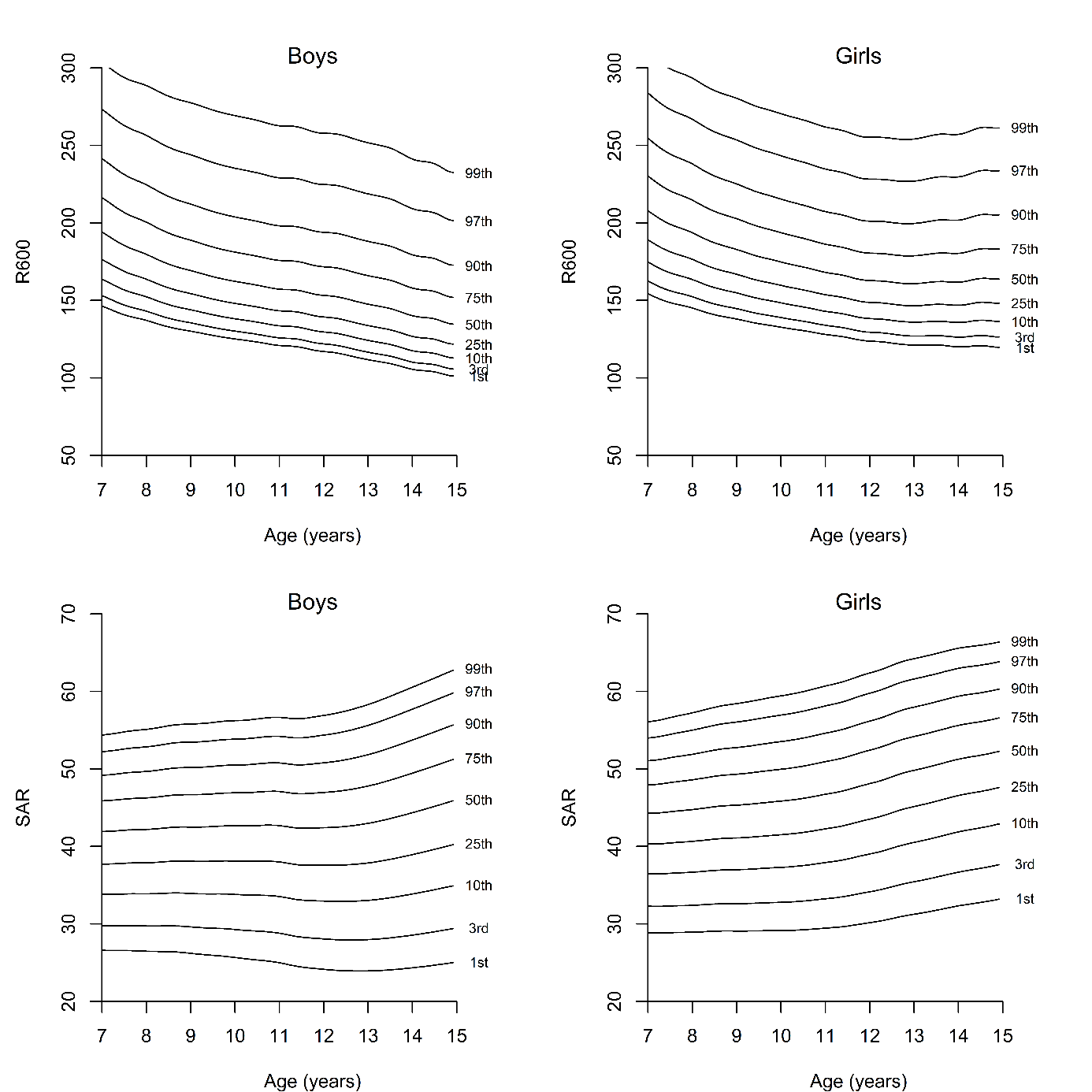


# 3.Results of quantile regression models

Supplementary Table 3.1: Coefficient estimates (standard errors) for different quantiles (tau) for BMI, boys; **p<*0.05, ***p<*0.01,****p<*0.001; age2=10-12 years, age3=13-15 years.

| Covariate | tau=0.1 | tau=0.25 | tau=0.5 | tau=0.75 | tau=0.9 |
| --- | --- | --- | --- | --- | --- |
| (Intercept) | 0.104(0.002)*** | 0.226(0.002)*** | 0.433(0.003)*** | 0.676(0.003)*** | 0.856(0.003)*** |
| year1990 | 0.009(0.002)*** | 0.013(0.003)*** | 0.018(0.003)*** | 0.026(0.004)*** | 0.014(0.003)*** |
| year1991 | 0.007(0.003)** | 0.017(0.003)** | 0.031(0.004)** | 0.039(0.004)** | 0.029(0.003)** |
| year1992 | 0.001(0.002) | 0.008(0.003) | 0.017(0.003) | 0.024(0.004) | 0.017(0.004) |
| year1993 | -0.004(0.002) | 0.005(0.003) | 0.021(0.004) | 0.041(0.004) | 0.038(0.003) |
| year1994 | -0.007(0.002)** | 0.003(0.003)** | 0.022(0.004)** | 0.047(0.004)** | 0.043(0.003)** |
| year1995 | 0(0.002) | 0.005(0.003) | 0.024(0.004) | 0.053(0.004) | 0.046(0.003) |
| year1996 | 0.002(0.003) | 0.012(0.003) | 0.038(0.004) | 0.066(0.004) | 0.056(0.003) |
| year1997 | -0.006(0.002)* | 0.006(0.003)* | 0.029(0.004)* | 0.065(0.004)* | 0.056(0.003)* |
| year1998 | -0.013(0.002)*** | -0.004(0.003)*** | 0.03(0.004)*** | 0.071(0.004)*** | 0.061(0.003)*** |
| year1999 | -0.01(0.002)*** | 0.001(0.003)*** | 0.038(0.004)*** | 0.084(0.004)*** | 0.067(0.003)*** |
| year2000 | -0.008(0.003)** | 0.004(0.003)** | 0.042(0.004)** | 0.087(0.004)** | 0.068(0.003)** |
| year2001 | -0.009(0.003)*** | 0.005(0.003)*** | 0.048(0.004)*** | 0.097(0.004)*** | 0.077(0.003)*** |
| year2002 | -0.004(0.003) | 0.011(0.003) | 0.047(0.004) | 0.089(0.004) | 0.074(0.003) |
| year2003 | -0.01(0.003)*** | 0.002(0.003)*** | 0.04(0.004)*** | 0.093(0.004)*** | 0.074(0.003)*** |
| year2004 | -0.002(0.003) | 0.012(0.003) | 0.057(0.004) | 0.112(0.004) | 0.077(0.003) |
| year2005 | -0.005(0.003) | 0.014(0.003) | 0.067(0.004) | 0.119(0.004) | 0.082(0.003) |
| year2006 | -0.002(0.003) | 0.017(0.003) | 0.077(0.004) | 0.13(0.004) | 0.089(0.003) |
| year2007 | -0.001(0.003) | 0.023(0.003) | 0.084(0.004) | 0.135(0.004) | 0.091(0.003) |
| year2008 | 0.008(0.003)** | 0.034(0.003)** | 0.095(0.004)** | 0.144(0.004)** | 0.095(0.003)** |
| year2009 | 0.005(0.003) | 0.032(0.004) | 0.093(0.004) | 0.136(0.004) | 0.093(0.003) |
| year2010 | 0.005(0.003) | 0.033(0.004) | 0.093(0.004) | 0.141(0.004) | 0.095(0.003) |
| year2011 | 0.003(0.003) | 0.024(0.004) | 0.085(0.004) | 0.133(0.004) | 0.093(0.003) |
| year2012 | 0.002(0.003) | 0.017(0.004) | 0.074(0.004) | 0.127(0.004) | 0.089(0.003) |
| year2013 | -0.002(0.003) | 0.022(0.003) | 0.079(0.004) | 0.128(0.004) | 0.091(0.003) |
| year2014 | -0.004(0.003) | 0.015(0.003) | 0.069(0.004) | 0.125(0.004) | 0.088(0.003) |
| year2015 | -0.011(0.003)*** | 0(0.003)*** | 0.051(0.004)*** | 0.111(0.004)*** | 0.084(0.003)*** |
| year2016 | -0.011(0.002)*** | 0.006(0.003)*** | 0.047(0.004)*** | 0.106(0.004)*** | 0.086(0.003)*** |
| year2017 | -0.01(0.002)*** | -0.001(0.003)*** | 0.037(0.004)*** | 0.098(0.004)*** | 0.08(0.003)*** |
| year2018 | -0.008(0.002)** | 0.006(0.003)** | 0.049(0.004)** | 0.105(0.004)** | 0.082(0.003)** |
| year2019 | -0.009(0.002)*** | -0.001(0.003)*** | 0.038(0.004)*** | 0.096(0.004)*** | 0.079(0.003)*** |
| age2 | -0.017(0.002)*** | -0.027(0.003)*** | -0.038(0.003)*** | -0.035(0.004)*** | -0.02(0.004)*** |
| age3 | -0.022(0.003)*** | -0.026(0.003)*** | -0.025(0.004)*** | -0.021(0.005)*** | -0.014(0.004)*** |
| region1 | 0.006(0.001)*** | 0.007(0.001)*** | -0.001(0.002)*** | -0.013(0.002)*** | -0.01(0.001)*** |
| region2 | 0.001(0.001) | 0.002(0.002) | -0.007(0.002) | -0.01(0.002) | -0.007(0.001) |
| region3 | 0.014(0.001)*** | 0.02(0.001)*** | 0.008(0.002)*** | -0.014(0.002)*** | -0.012(0.001)*** |
| region4 | 0.008(0.002)*** | 0.017(0.002)*** | 0.025(0.003)*** | 0.02(0.002)*** | 0.002(0.001)*** |
| region5 | 0.03(0.001)*** | 0.044(0.002)*** | 0.037(0.002)*** | 0.015(0.002)*** | 0.003(0.001)*** |
| region6 | 0.02(0.001)*** | 0.028(0.001)*** | 0.016(0.002)*** | -0.006(0.002)*** | -0.008(0.001)*** |
| region7 | 0.009(0.001)*** | 0.008(0.001)*** | -0.008(0.002)*** | -0.029(0.001)*** | -0.023(0.001)*** |
| region8 | 0.006(0.001)*** | -0.003(0.001)*** | -0.03(0.002)*** | -0.051(0.002)*** | -0.031(0.001)*** |
| region9 | 0.021(0.002)*** | 0.029(0.002)*** | 0.02(0.003)*** | 0.002(0.002)*** | -0.003(0.001)*** |
| Covariate | tau=0.1 | tau=0.25 | tau=0.5 | tau=0.75 | tau=0.9 |
| region10 | 0.025(0.001)*** | 0.03(0.002)*** | 0.017(0.002)*** | -0.006(0.002)*** | -0.008(0.001)*** |
| region11 | 0.028(0.001)*** | 0.043(0.002)*** | 0.044(0.002)*** | 0.028(0.002)*** | 0.007(0.001)*** |
| year1990age2 | -0.005(0.003) | -0.005(0.004) | -0.007(0.005) | -0.013(0.006) | -0.001(0.005) |
| year1991age2 | 0.004(0.003) | 0.003(0.004) | -0.001(0.005) | -0.002(0.006) | 0.002(0.005) |
| year1992age2 | 0.006(0.003) | 0.003(0.004) | 0.002(0.005) | -0.007(0.005) | 0.001(0.005) |
| year1993age2 | 0.008(0.003)* | 0.011(0.004)* | 0.013(0.005)* | 0.007(0.006)* | 0.005(0.005)* |
| year1994age2 | 0.01(0.003)** | 0.014(0.004)** | 0.017(0.005)** | 0.015(0.006)** | 0.01(0.005)** |
| year1995age2 | 0.005(0.003) | 0.012(0.004) | 0.016(0.005) | 0.013(0.006) | 0.008(0.005) |
| year1996age2 | 0.007(0.003)* | 0.014(0.004)* | 0.01(0.005)* | 0.012(0.006)* | 0.008(0.005)* |
| year1997age2 | 0.013(0.003)*** | 0.014(0.004)*** | 0.016(0.005)*** | 0.004(0.006)*** | 0.004(0.005)*** |
| year1998age2 | 0.021(0.003)*** | 0.03(0.004)*** | 0.026(0.005)*** | 0.012(0.006)*** | 0.006(0.005)*** |
| year1999age2 | 0.017(0.003)*** | 0.026(0.004)*** | 0.023(0.005)*** | 0.01(0.006)*** | 0.009(0.005)*** |
| year2000age2 | 0.019(0.004)*** | 0.029(0.004)*** | 0.028(0.005)*** | 0.026(0.006)*** | 0.016(0.004)*** |
| year2001age2 | 0.018(0.004)*** | 0.028(0.004)*** | 0.034(0.006)*** | 0.028(0.006)*** | 0.012(0.004)*** |
| year2002age2 | 0.011(0.004)** | 0.019(0.004)** | 0.029(0.005)** | 0.031(0.006)** | 0.012(0.004)** |
| year2003age2 | 0.016(0.004)*** | 0.026(0.004)*** | 0.039(0.006)*** | 0.024(0.006)*** | 0.01(0.005)*** |
| year2004age2 | 0.015(0.004)*** | 0.029(0.005)*** | 0.037(0.006)*** | 0.026(0.006)*** | 0.015(0.004)*** |
| year2005age2 | 0.021(0.004)*** | 0.037(0.005)*** | 0.043(0.006)*** | 0.033(0.006)*** | 0.02(0.004)*** |
| year2006age2 | 0.023(0.004)*** | 0.039(0.005)*** | 0.043(0.006)*** | 0.032(0.006)*** | 0.014(0.004)*** |
| year2007age2 | 0.024(0.004)*** | 0.038(0.005)*** | 0.043(0.006)*** | 0.039(0.006)*** | 0.017(0.004)*** |
| year2008age2 | 0.013(0.004)*** | 0.03(0.005)*** | 0.048(0.006)*** | 0.036(0.006)*** | 0.016(0.004)*** |
| year2009age2 | 0.025(0.004)*** | 0.042(0.005)*** | 0.055(0.006)*** | 0.05(0.006)*** | 0.021(0.004)*** |
| year2010age2 | 0.024(0.004)*** | 0.048(0.005)*** | 0.069(0.006)*** | 0.057(0.006)*** | 0.022(0.004)*** |
| year2011age2 | 0.027(0.004)*** | 0.054(0.005)*** | 0.067(0.006)*** | 0.062(0.006)*** | 0.025(0.004)*** |
| year2012age2 | 0.022(0.004)*** | 0.055(0.005)*** | 0.073(0.006)*** | 0.06(0.006)*** | 0.027(0.004)*** |
| year2013age2 | 0.028(0.004)*** | 0.049(0.005)*** | 0.067(0.006)*** | 0.058(0.006)*** | 0.025(0.004)*** |
| year2014age2 | 0.02(0.004)*** | 0.044(0.005)*** | 0.064(0.006)*** | 0.054(0.006)*** | 0.025(0.004)*** |
| year2015age2 | 0.022(0.004)*** | 0.053(0.005)*** | 0.07(0.006)*** | 0.063(0.006)*** | 0.027(0.004)*** |
| year2016age2 | 0.025(0.004)*** | 0.047(0.005)*** | 0.078(0.006)*** | 0.076(0.006)*** | 0.027(0.004)*** |
| year2017age2 | 0.016(0.004)*** | 0.044(0.005)*** | 0.08(0.006)*** | 0.077(0.006)*** | 0.033(0.004)*** |
| year2018age2 | 0.015(0.004)*** | 0.04(0.005)*** | 0.07(0.006)*** | 0.072(0.006)*** | 0.033(0.004)*** |
| year2019age2 | 0.01(0.003)** | 0.039(0.004)** | 0.07(0.006)** | 0.075(0.006)** | 0.034(0.004)** |
| year1990age3 | -0.008(0.004)* | -0.005(0.005)* | -0.004(0.006)* | -0.012(0.007)* | -0.004(0.006)* |
| year1991age3 | 0(0.004) | 0.001(0.005) | 0.003(0.006) | -0.005(0.007) | -0.003(0.006) |
| year1992age3 | 0.004(0.004) | 0.004(0.005) | 0.002(0.006) | -0.01(0.006) | -0.008(0.006) |
| year1993age3 | 0.009(0.004)* | 0.015(0.005)* | 0.015(0.006)* | -0.003(0.006)* | -0.008(0.006)* |
| year1994age3 | 0.015(0.004)*** | 0.02(0.005)*** | 0.019(0.006)*** | 0.006(0.007)*** | 0.001(0.006)*** |
| year1995age3 | 0.011(0.004)** | 0.024(0.005)** | 0.026(0.006)** | 0.004(0.006)** | 0.004(0.005)** |
| year1996age3 | 0.009(0.004)* | 0.018(0.005)* | 0.019(0.006)* | 0.001(0.006)* | 0.001(0.005)* |
| year1997age3 | 0.013(0.004)*** | 0.018(0.005)*** | 0.019(0.006)*** | -0.006(0.007)*** | -0.006(0.005)*** |
| year1998age3 | 0.019(0.004)*** | 0.028(0.005)*** | 0.022(0.006)*** | -0.003(0.007)*** | -0.005(0.005)*** |
| year1999age3 | 0.018(0.004)*** | 0.026(0.005)*** | 0.02(0.006)*** | -0.01(0.007)*** | -0.004(0.005)*** |
| year2000age3 | 0.022(0.004)*** | 0.03(0.005)*** | 0.023(0.006)*** | 0.001(0.007)*** | 0.004(0.005)*** |
| year2001age3 | 0.022(0.004)*** | 0.037(0.005)*** | 0.031(0.006)*** | 0.001(0.007)*** | 0.004(0.005)*** |
| year2002age3 | 0.019(0.004)*** | 0.03(0.005)*** | 0.031(0.006)*** | 0.012(0.007)*** | 0.005(0.005)*** |
| Covariate | tau=0.1 | tau=0.25 | tau=0.5 | tau=0.75 | tau=0.9 |
| year2003age3 | 0.02(0.004)*** | 0.028(0.005)*** | 0.03(0.006)*** | 0.007(0.007)*** | 0.008(0.005)*** |
| year2004age3 | 0.022(0.004)*** | 0.037(0.005)*** | 0.039(0.007)*** | 0.011(0.007)*** | 0.011(0.005)*** |
| year2005age3 | 0.027(0.004)*** | 0.04(0.005)*** | 0.038(0.007)*** | 0.012(0.007)*** | 0.011(0.005)*** |
| year2006age3 | 0.027(0.004)*** | 0.046(0.006)*** | 0.036(0.007)*** | 0.011(0.007)*** | 0.01(0.005)*** |
| year2007age3 | 0.024(0.004)*** | 0.042(0.006)*** | 0.038(0.007)*** | 0.016(0.007)*** | 0.015(0.005)*** |
| year2008age3 | 0.019(0.004)*** | 0.035(0.006)*** | 0.033(0.007)*** | 0.02(0.007)*** | 0.016(0.005)*** |
| year2009age3 | 0.028(0.005)*** | 0.046(0.006)*** | 0.048(0.007)*** | 0.033(0.007)*** | 0.017(0.005)*** |
| year2010age3 | 0.025(0.005)*** | 0.049(0.006)*** | 0.056(0.007)*** | 0.033(0.007)*** | 0.018(0.005)*** |
| year2011age3 | 0.032(0.005)*** | 0.058(0.006)*** | 0.061(0.007)*** | 0.043(0.007)*** | 0.023(0.005)*** |
| year2012age3 | 0.03(0.005)*** | 0.06(0.006)*** | 0.068(0.007)*** | 0.051(0.007)*** | 0.026(0.005)*** |
| year2013age3 | 0.023(0.005)*** | 0.049(0.006)*** | 0.061(0.007)*** | 0.048(0.007)*** | 0.025(0.005)*** |
| year2014age3 | 0.026(0.004)*** | 0.046(0.006)*** | 0.057(0.007)*** | 0.039(0.007)*** | 0.026(0.005)*** |
| year2015age3 | 0.027(0.004)*** | 0.055(0.006)*** | 0.07(0.007)*** | 0.05(0.007)*** | 0.027(0.005)*** |
| year2016age3 | 0.026(0.004)*** | 0.043(0.006)*** | 0.066(0.007)*** | 0.057(0.007)*** | 0.025(0.005)*** |
| year2017age3 | 0.018(0.004)*** | 0.044(0.006)*** | 0.07(0.007)*** | 0.063(0.007)*** | 0.031(0.005)*** |
| year2018age3 | 0.017(0.004)*** | 0.038(0.006)*** | 0.06(0.007)*** | 0.053(0.007)*** | 0.027(0.005)*** |
| year2019age3 | 0.014(0.004)*** | 0.042(0.005)*** | 0.079(0.007)*** | 0.068(0.007)*** | 0.035(0.005)*** |

Supplementary Table 3.2: Coefficient estimates (standard errors) for different quantiles (tau) for BMI, girls; **p<*0.05, ***p<*0.01,****p<*0.001, age2=10-12 years, age3=13-15 years.

| Covariate | tau=0.1 | tau=0.25 | tau=0.5 | tau=0.75 | tau=0.9 |
| --- | --- | --- | --- | --- | --- |
| (Intercept) | 0.095(0.002)*** | 0.216(0.002)*** | 0.431(0.003)*** | 0.684(0.003)*** | 0.868(0.003)*** |
| year1990 | 0.006(0.002)* | 0.01(0.003)* | 0.012(0.004)* | 0.019(0.004)* | 0.01(0.003)* |
| year1991 | 0.01(0.002)*** | 0.021(0.003)*** | 0.026(0.004)*** | 0.037(0.004)*** | 0.018(0.003)*** |
| year1992 | 0.004(0.002) | 0.006(0.003) | 0.012(0.004) | 0.015(0.004) | 0.005(0.004) |
| year1993 | 0.004(0.002) | 0.014(0.003) | 0.029(0.004) | 0.042(0.004) | 0.028(0.003) |
| year1994 | -0.001(0.002) | 0.008(0.003) | 0.029(0.004) | 0.052(0.004) | 0.038(0.003) |
| year1995 | 0.003(0.002) | 0.016(0.003) | 0.037(0.004) | 0.056(0.004) | 0.042(0.003) |
| year1996 | 0.003(0.002) | 0.016(0.003) | 0.042(0.004) | 0.066(0.004) | 0.047(0.003) |
| year1997 | 0.003(0.002) | 0.017(0.003) | 0.039(0.004) | 0.06(0.004) | 0.045(0.003) |
| year1998 | -0.003(0.002) | 0.008(0.003) | 0.044(0.004) | 0.077(0.004) | 0.049(0.003) |
| year1999 | 0(0.003) | 0.016(0.003) | 0.049(0.004) | 0.082(0.004) | 0.055(0.003) |
| year2000 | 0(0.003) | 0.023(0.003) | 0.055(0.004) | 0.089(0.004) | 0.06(0.003) |
| year2001 | -0.001(0.003) | 0.015(0.003) | 0.056(0.004) | 0.094(0.004) | 0.063(0.003) |
| year2002 | 0(0.003) | 0.019(0.003) | 0.059(0.004) | 0.095(0.004) | 0.062(0.003) |
| year2003 | -0.002(0.003) | 0.016(0.003) | 0.055(0.004) | 0.091(0.004) | 0.06(0.003) |
| year2004 | 0.006(0.003)* | 0.029(0.004)* | 0.075(0.004)* | 0.102(0.004)* | 0.07(0.003)* |
| year2005 | 0.006(0.003)* | 0.026(0.004)* | 0.079(0.004)* | 0.115(0.004)* | 0.073(0.003)* |
| year2006 | 0.007(0.003)** | 0.027(0.004)** | 0.084(0.004)** | 0.122(0.004)** | 0.075(0.003)** |
| year2007 | 0.01(0.003)*** | 0.039(0.004)*** | 0.094(0.004)*** | 0.129(0.004)*** | 0.08(0.003)*** |
| year2008 | 0.011(0.003)*** | 0.037(0.004)*** | 0.089(0.004)*** | 0.13(0.004)*** | 0.079(0.003)*** |
| year2009 | 0.012(0.003)*** | 0.036(0.003)*** | 0.085(0.004)*** | 0.124(0.004)*** | 0.077(0.003)*** |
| year2010 | 0.01(0.003)*** | 0.041(0.003)*** | 0.092(0.005)*** | 0.129(0.004)*** | 0.079(0.003)*** |
| year2011 | 0.009(0.003)*** | 0.036(0.004)*** | 0.087(0.004)*** | 0.123(0.004)*** | 0.078(0.003)*** |
| year2012 | 0.011(0.003)*** | 0.035(0.004)*** | 0.085(0.004)*** | 0.119(0.004)*** | 0.076(0.003)*** |
| year2013 | 0.007(0.002)** | 0.03(0.004)** | 0.083(0.004)** | 0.126(0.004)** | 0.077(0.003)** |
| year2014 | 0.006(0.003)* | 0.027(0.003)* | 0.074(0.004)* | 0.114(0.004)* | 0.073(0.003)* |
| year2015 | -0.001(0.002) | 0.016(0.003) | 0.057(0.004) | 0.105(0.004) | 0.07(0.003) |
| year2016 | 0.004(0.002) | 0.019(0.003) | 0.065(0.004) | 0.108(0.004) | 0.072(0.003) |
| year2017 | 0.005(0.002)* | 0.02(0.003)* | 0.059(0.004)* | 0.104(0.004)* | 0.071(0.003)* |
| year2018 | 0.001(0.002) | 0.016(0.003) | 0.057(0.004) | 0.102(0.004) | 0.067(0.003) |
| year2019 | 0(0.002) | 0.014(0.003) | 0.052(0.004) | 0.091(0.004) | 0.066(0.003) |
| age2 | -0.006(0.002)** | -0.005(0.003)** | -0.001(0.004)** | 0.003(0.004)** | -0.003(0.004)** |
| age3 | -0.009(0.003)** | 0.004(0.004)** | 0.021(0.005)** | 0.028(0.005)** | 0.01(0.004)** |
| region1 | 0.003(0.001)*** | 0.006(0.001)*** | -0.006(0.002)*** | -0.02(0.002)*** | -0.014(0.001)*** |
| region2 | 0.007(0.001)*** | 0.007(0.002)*** | -0.002(0.002)*** | -0.02(0.002)*** | -0.016(0.001)*** |
| region3 | 0.011(0.001)*** | 0.017(0.001)*** | 0.004(0.002)*** | -0.015(0.002)*** | -0.015(0.001)*** |
| region4 | 0.018(0.002)*** | 0.033(0.002)*** | 0.031(0.003)*** | 0.012(0.003)*** | 0.001(0.001)*** |
| region5 | 0.032(0.001)*** | 0.045(0.002)*** | 0.04(0.002)*** | 0.015(0.002)*** | 0.002(0.001)*** |
| region6 | 0.02(0.001)*** | 0.027(0.002)*** | 0.015(0.002)*** | -0.01(0.002)*** | -0.009(0.001)*** |
| region7 | 0.006(0.001)*** | 0.003(0.001)*** | -0.017(0.002)*** | -0.038(0.002)*** | -0.028(0.001)*** |
| region8 | 0.005(0.001)*** | 0(0.001)*** | -0.026(0.002)*** | -0.051(0.002)*** | -0.035(0.001)*** |
| Covariate | tau=0.1 | tau=0.25 | tau=0.5 | tau=0.75 | tau=0.9 |
| region9 | 0.023(0.002)*** | 0.034(0.002)*** | 0.026(0.003)*** | 0.002(0.002)*** | -0.007(0.001)*** |
| region10 | 0.023(0.001)*** | 0.032(0.002)*** | 0.017(0.002)*** | -0.01(0.002)*** | -0.014(0.001)*** |
| region11 | 0.029(0.001)*** | 0.044(0.002)*** | 0.043(0.002)*** | 0.019(0.002)*** | 0.003(0.001)*** |
| year1990age2 | -0.004(0.003) | -0.007(0.004) | -0.007(0.005) | -0.015(0.006) | -0.005(0.005) |
| year1991age2 | -0.004(0.003) | -0.003(0.004) | 0.001(0.005) | -0.009(0.006) | 0.001(0.005) |
| year1992age2 | -0.004(0.003) | -0.003(0.004) | -0.013(0.005) | -0.017(0.006) | -0.003(0.005) |
| year1993age2 | -0.002(0.003) | -0.005(0.004) | -0.008(0.005) | -0.015(0.006) | -0.006(0.005) |
| year1994age2 | -0.001(0.003) | 0.001(0.004) | -0.005(0.005) | -0.016(0.006) | -0.007(0.005) |
| year1995age2 | 0(0.003) | -0.002(0.004) | -0.007(0.005) | -0.015(0.006) | -0.009(0.005) |
| year1996age2 | 0.001(0.003) | 0.003(0.004) | -0.003(0.005) | -0.009(0.006) | -0.005(0.005) |
| year1997age2 | 0.001(0.003) | -0.002(0.005) | -0.005(0.006) | -0.012(0.006) | -0.008(0.005) |
| year1998age2 | 0.005(0.003) | 0.008(0.005) | -0.004(0.006) | -0.017(0.006) | -0.004(0.005) |
| year1999age2 | 0.001(0.004) | 0(0.005) | -0.007(0.006) | -0.019(0.006) | -0.005(0.005) |
| year2000age2 | 0.003(0.004) | -0.001(0.005) | -0.001(0.006) | -0.009(0.006) | -0.004(0.005) |
| year2001age2 | 0.006(0.004) | 0.011(0.005) | 0.001(0.006) | -0.011(0.006) | -0.003(0.005) |
| year2002age2 | 0.002(0.004) | 0.001(0.005) | -0.009(0.006) | -0.019(0.006) | -0.006(0.005) |
| year2003age2 | 0.006(0.004) | 0.003(0.005) | -0.006(0.006) | -0.018(0.006) | -0.007(0.005) |
| year2004age2 | 0.006(0.004) | 0.006(0.005) | -0.01(0.006) | -0.014(0.006) | -0.008(0.005) |
| year2005age2 | 0.004(0.004) | 0.006(0.005) | -0.011(0.006) | -0.017(0.006) | -0.004(0.004) |
| year2006age2 | 0.011(0.004)** | 0.02(0.005)** | 0.004(0.006)** | -0.011(0.006)** | 0(0.004)** |
| year2007age2 | 0.004(0.004) | 0.008(0.005) | -0.006(0.006) | -0.014(0.006) | -0.001(0.004) |
| year2008age2 | 0.003(0.004) | 0.009(0.005) | 0.012(0.006) | -0.005(0.006) | 0.002(0.004) |
| year2009age2 | 0.009(0.004)* | 0.014(0.005)* | 0.016(0.006)* | 0.001(0.006)* | 0.006(0.004)* |
| year2010age2 | 0.017(0.004)*** | 0.027(0.005)*** | 0.029(0.006)*** | 0.012(0.006)*** | 0.009(0.004)*** |
| year2011age2 | 0.013(0.004)** | 0.023(0.005)** | 0.031(0.006)** | 0.015(0.006)** | 0.01(0.004)** |
| year2012age2 | 0.006(0.004) | 0.015(0.005) | 0.024(0.006) | 0.014(0.006) | 0.009(0.004) |
| year2013age2 | 0(0.004) | 0.014(0.005) | 0.015(0.006) | 0.003(0.006) | 0.009(0.004) |
| year2014age2 | -0.001(0.004) | 0.01(0.005) | 0.021(0.006) | 0.012(0.006) | 0.008(0.004) |
| year2015age2 | 0.004(0.004) | 0.017(0.005) | 0.03(0.006) | 0.016(0.006) | 0.012(0.004) |
| year2016age2 | 0.002(0.004) | 0.014(0.005) | 0.027(0.006) | 0.021(0.006) | 0.013(0.004) |
| year2017age2 | 0.001(0.004) | 0.01(0.005) | 0.025(0.006) | 0.015(0.006) | 0.011(0.004) |
| year2018age2 | 0.003(0.004) | 0.013(0.005) | 0.025(0.006) | 0.016(0.006) | 0.014(0.004) |
| year2019age2 | 0.001(0.004) | 0.013(0.005) | 0.023(0.006) | 0.024(0.006) | 0.014(0.004) |
| year1990age3 | 0.004(0.004) | 0.003(0.005) | 0.003(0.006) | 0(0.006) | -0.005(0.005) |
| year1991age3 | 0.009(0.004)* | 0.003(0.005)* | 0.006(0.006)* | -0.008(0.006)* | -0.001(0.005)* |
| year1992age3 | 0.008(0.004) | 0.004(0.005) | -0.001(0.006) | -0.013(0.006) | -0.004(0.005) |
| year1993age3 | 0.008(0.004) | 0(0.005) | -0.01(0.006) | -0.025(0.006) | -0.017(0.005) |
| year1994age3 | 0.006(0.004) | 0.004(0.005) | -0.012(0.006) | -0.033(0.006) | -0.022(0.005) |
| year1995age3 | 0.001(0.004) | -0.005(0.005) | -0.016(0.006) | -0.033(0.006) | -0.024(0.005) |
| year1996age3 | 0.006(0.004) | 0.001(0.005) | -0.011(0.006) | -0.03(0.006) | -0.02(0.005) |
| year1997age3 | 0.004(0.004) | -0.009(0.005) | -0.018(0.006) | -0.027(0.006) | -0.021(0.005) |
| year1998age3 | 0.006(0.004) | 0(0.005) | -0.022(0.007) | -0.042(0.006) | -0.02(0.005) |
| year1999age3 | -0.004(0.004) | -0.016(0.005) | -0.038(0.007) | -0.05(0.007) | -0.026(0.005) |
| year2000age3 | -0.002(0.004) | -0.026(0.006) | -0.037(0.007) | -0.054(0.007) | -0.029(0.005) |
| year2001age3 | -0.003(0.004) | -0.022(0.006) | -0.041(0.007) | -0.056(0.007) | -0.028(0.005) |
| year2002age3 | 0(0.004) | -0.019(0.006) | -0.046(0.007) | -0.059(0.007) | -0.023(0.005) |
| Covariate | tau=0.1 | tau=0.25 | tau=0.5 | tau=0.75 | tau=0.9 |
| year2003age3 | 0.001(0.004) | -0.014(0.006) | -0.032(0.007) | -0.049(0.007) | -0.025(0.005) |
| year2004age3 | 0.001(0.004) | -0.013(0.006) | -0.035(0.007) | -0.052(0.007) | -0.032(0.005) |
| year2005age3 | 0.003(0.004) | -0.007(0.006) | -0.037(0.007) | -0.058(0.007) | -0.031(0.005) |
| year2006age3 | 0.007(0.004) | 0.002(0.006) | -0.03(0.007) | -0.06(0.007) | -0.027(0.005) |
| year2007age3 | 0.006(0.005) | -0.012(0.006) | -0.035(0.007) | -0.052(0.007) | -0.019(0.005) |
| year2008age3 | -0.002(0.005) | -0.012(0.006) | -0.026(0.007) | -0.049(0.007) | -0.016(0.005) |
| year2009age3 | 0.002(0.005) | 0.009(0.006) | -0.013(0.007) | -0.034(0.007) | -0.012(0.005) |
| year2010age3 | 0.015(0.005)** | 0.012(0.006)** | -0.003(0.008)** | -0.029(0.007)** | -0.01(0.005)** |
| year2011age3 | 0.017(0.005)*** | 0.015(0.006)*** | 0.006(0.007)*** | -0.013(0.007)*** | -0.005(0.005)*** |
| year2012age3 | 0.016(0.005)*** | 0.015(0.006)*** | 0.011(0.008)*** | -0.008(0.007)*** | -0.003(0.005)*** |
| year2013age3 | 0.014(0.005)** | 0.02(0.006)** | 0.01(0.008)** | -0.012(0.007)** | -0.004(0.005)** |
| year2014age3 | 0.004(0.004) | 0.004(0.006) | 0.003(0.007) | -0.012(0.007) | 0.001(0.005) |
| year2015age3 | 0.01(0.004)* | 0.019(0.006)* | 0.026(0.007)* | -0.005(0.007)* | 0.003(0.005)* |
| year2016age3 | 0.003(0.004) | 0.015(0.006) | 0.015(0.007) | -0.001(0.007) | -0.001(0.005) |
| year2017age3 | 0(0.004) | 0.013(0.006) | 0.027(0.007) | 0.004(0.007) | 0.002(0.005) |
| year2018age3 | 0.014(0.004)** | 0.028(0.006)** | 0.033(0.007)** | 0.008(0.007)** | 0.005(0.005)** |
| year2019age3 | 0.012(0.005)** | 0.021(0.006)** | 0.029(0.008)** | 0.013(0.007)** | 0.008(0.005)** |

Supplementary Table 3.3: Coefficient estimates (standard errors) for different quantiles (tau) for TSF, boys; **p<*0.05, ***p<*0.01,****p<*0.001, age2=10-12 years, age3=13-15 years.

| Covariate | tau=0.1 | tau=0.25 | tau=0.5 | tau=0.75 | tau=0.9 |
| --- | --- | --- | --- | --- | --- |
| (Intercept) | 0.103(0.001)*** | 0.212(0.002)*** | 0.411(0.004)*** | 0.668(0.003)*** | 0.862(0.003)*** |
| year1990 | -0.002(0.001) | 0(0.002) | 0.008(0.005) | -0.002(0.005) | 0(0.004) |
| year1991 | -0.001(0.001) | 0.004(0.002) | 0.015(0.005) | 0.008(0.004) | 0.005(0.004) |
| year1992 | 0.001(0.001) | 0.003(0.002) | 0.015(0.005) | 0.001(0.005) | -0.004(0.004) |
| year1993 | -0.001(0.001) | 0.003(0.002) | 0.025(0.005) | 0.02(0.004) | 0.013(0.004) |
| year1994 | 0(0.001) | 0.003(0.002) | 0.021(0.005) | 0.021(0.004) | 0.019(0.004) |
| year1995 | -0.001(0.001) | 0.003(0.002) | 0.024(0.005) | 0.025(0.004) | 0.019(0.004) |
| year1996 | -0.004(0.001)** | 0(0.002)** | 0.025(0.005)** | 0.033(0.004)** | 0.027(0.004)** |
| year1997 | -0.003(0.001)* | 0.003(0.002)* | 0.034(0.004)* | 0.04(0.004)* | 0.033(0.004)* |
| year1998 | -0.002(0.001) | 0.003(0.002) | 0.034(0.005) | 0.051(0.005) | 0.042(0.003) |
| year1999 | -0.005(0.002)** | 0.003(0.002)** | 0.043(0.004)** | 0.06(0.005)** | 0.045(0.003)** |
| year2000 | -0.001(0.001) | 0.013(0.002) | 0.053(0.004) | 0.072(0.005) | 0.05(0.003) |
| year2001 | 0(0.001) | 0.016(0.002) | 0.065(0.004) | 0.092(0.005) | 0.059(0.003) |
| year2002 | 0.006(0.001)*** | 0.022(0.003)*** | 0.07(0.004)*** | 0.093(0.004)*** | 0.06(0.003)*** |
| year2003 | 0.011(0.001)*** | 0.049(0.004)*** | 0.089(0.004)*** | 0.11(0.004)*** | 0.068(0.003)*** |
| year2004 | 0.015(0.001)*** | 0.058(0.004)*** | 0.099(0.005)*** | 0.124(0.004)*** | 0.073(0.003)*** |
| year2005 | 0.017(0.001)*** | 0.065(0.003)*** | 0.116(0.005)*** | 0.132(0.004)*** | 0.078(0.003)*** |
| year2006 | 0.021(0.002)*** | 0.085(0.004)*** | 0.148(0.005)*** | 0.153(0.004)*** | 0.086(0.003)*** |
| year2007 | 0.02(0.001)*** | 0.078(0.004)*** | 0.141(0.005)*** | 0.15(0.004)*** | 0.084(0.003)*** |
| year2008 | 0.022(0.001)*** | 0.085(0.004)*** | 0.155(0.005)*** | 0.158(0.004)*** | 0.087(0.003)*** |
| year2009 | 0.022(0.002)*** | 0.089(0.004)*** | 0.156(0.005)*** | 0.156(0.004)*** | 0.085(0.003)*** |
| year2010 | 0.022(0.002)*** | 0.093(0.004)*** | 0.158(0.005)*** | 0.156(0.004)*** | 0.085(0.003)*** |
| year2011 | 0.022(0.002)*** | 0.089(0.004)*** | 0.149(0.005)*** | 0.152(0.004)*** | 0.084(0.003)*** |
| year2012 | 0.023(0.002)*** | 0.088(0.004)*** | 0.147(0.005)*** | 0.146(0.004)*** | 0.083(0.003)*** |
| year2013 | 0.022(0.002)*** | 0.093(0.004)*** | 0.148(0.005)*** | 0.152(0.004)*** | 0.085(0.003)*** |
| year2014 | 0.022(0.001)*** | 0.085(0.004)*** | 0.143(0.005)*** | 0.153(0.004)*** | 0.084(0.003)*** |
| year2015 | 0.021(0.001)*** | 0.074(0.004)*** | 0.136(0.005)*** | 0.146(0.004)*** | 0.081(0.003)*** |
| year2016 | 0.022(0.001)*** | 0.086(0.004)*** | 0.138(0.005)*** | 0.148(0.004)*** | 0.084(0.003)*** |
| year2017 | 0.02(0.001)*** | 0.068(0.004)*** | 0.122(0.005)*** | 0.13(0.004)*** | 0.078(0.003)*** |
| year2018 | 0.022(0.001)*** | 0.087(0.003)*** | 0.139(0.005)*** | 0.147(0.004)*** | 0.083(0.003)*** |
| year2019 | 0.022(0.001)*** | 0.082(0.004)*** | 0.127(0.005)*** | 0.135(0.004)*** | 0.077(0.003)*** |
| age2 | -0.02(0.001)*** | 0.006(0.002)*** | -0.006(0.004)*** | -0.015(0.005)*** | -0.006(0.004)*** |
| age3 | 0.004(0.002)* | 0.017(0.004)* | 0.033(0.005)* | 0.023(0.006)* | 0.011(0.004)* |
| region1 | -0.005(0.001)*** | -0.006(0.001)*** | -0.016(0.002)*** | -0.02(0.002)*** | -0.015(0.001)*** |
| region2 | 0.011(0.001)*** | 0.013(0.001)*** | 0.007(0.002)*** | -0.008(0.002)*** | -0.01(0.001)*** |
| region3 | -0.006(0.001)*** | -0.011(0.001)*** | -0.024(0.002)*** | -0.027(0.002)*** | -0.016(0.001)*** |
| region4 | 0.021(0.002)*** | 0.043(0.003)*** | 0.059(0.003)*** | 0.058(0.002)*** | 0.021(0.001)*** |
| region5 | 0.004(0.001)*** | 0.007(0.001)*** | 0.003(0.002)*** | 0.001(0.002)*** | 0(0.001)*** |
| region6 | 0(0.001) | 0.002(0.001) | 0.003(0.002) | -0.003(0.002) | -0.007(0.001) |
| region7 | 0(0.001) | -0.002(0.001) | -0.017(0.002) | -0.025(0.001) | -0.018(0.001) |
| region8 | -0.002(0.001)** | -0.006(0.001)** | -0.027(0.002)** | -0.037(0.002)** | -0.022(0.001)** |
| region9 | 0.004(0.001)*** | 0.01(0.002)*** | 0.014(0.003)*** | 0.006(0.002)*** | 0.001(0.001)*** |
| Covariate | tau=0.1 | tau=0.25 | tau=0.5 | tau=0.75 | tau=0.9 |
| region10 | 0.004(0.001)*** | 0.007(0.001)*** | -0.001(0.002)*** | -0.007(0.002)*** | -0.007(0.001)*** |
| region11 | 0.006(0.001)*** | 0.013(0.001)*** | 0.033(0.002)*** | 0.034(0.002)*** | 0.012(0.001)*** |
| year1990age2 | 0.002(0.002) | 0.001(0.003) | -0.009(0.005) | 0.004(0.006) | 0.003(0.005) |
| year1991age2 | 0.001(0.002) | 0(0.003) | -0.006(0.005) | 0.01(0.006) | 0.008(0.005) |
| year1992age2 | 0(0.002) | 0(0.003) | -0.016(0.005) | 0.004(0.006) | 0.006(0.005) |
| year1993age2 | 0.002(0.002) | 0.001(0.003) | -0.019(0.005) | 0.004(0.006) | 0.005(0.005) |
| year1994age2 | 0.001(0.002) | 0.002(0.003) | -0.005(0.006) | 0.019(0.006) | 0.01(0.005) |
| year1995age2 | 0.001(0.002) | 0(0.003) | -0.016(0.005) | 0.006(0.006) | 0.003(0.005) |
| year1996age2 | 0.004(0.002)* | 0.003(0.003)* | -0.012(0.005)* | 0.012(0.006)* | 0.002(0.005)* |
| year1997age2 | 0.003(0.002) | 0(0.003) | -0.021(0.005) | 0.002(0.006) | -0.007(0.005) |
| year1998age2 | 0.003(0.002) | 0.002(0.003) | -0.012(0.006) | 0.004(0.006) | -0.009(0.005) |
| year1999age2 | 0.005(0.002)** | 0.002(0.003)** | -0.007(0.006)** | 0.005(0.006)** | -0.005(0.005)** |
| year2000age2 | 0.004(0.002)* | -0.001(0.003)* | 0.01(0.005)* | 0.019(0.007)* | 0.005(0.005)* |
| year2001age2 | 0.003(0.002) | -0.003(0.003) | 0.009(0.005) | 0.016(0.007) | 0(0.005) |
| year2002age2 | -0.001(0.002) | -0.008(0.003) | 0.002(0.005) | 0.014(0.006) | -0.002(0.005) |
| year2003age2 | -0.003(0.002) | -0.029(0.004) | -0.011(0.005) | -0.002(0.006) | -0.009(0.005) |
| year2004age2 | 0.04(0.002)*** | -0.03(0.004)*** | 0.008(0.007)*** | 0.007(0.006)*** | -0.002(0.005)*** |
| year2005age2 | 0.039(0.002)*** | 0.004(0.006)*** | 0.007(0.006)*** | 0.013(0.006)*** | 0(0.005)*** |
| year2006age2 | 0.038(0.002)*** | -0.002(0.004)*** | -0.007(0.006)*** | 0.007(0.006)*** | -0.003(0.004)*** |
| year2007age2 | 0.04(0.002)*** | 0.006(0.005)*** | 0.002(0.006)*** | 0.013(0.006)*** | 0(0.004)*** |
| year2008age2 | 0.041(0.002)*** | 0.001(0.005)*** | -0.004(0.007)*** | 0.009(0.006)*** | 0.003(0.004)*** |
| year2009age2 | 0.041(0.002)*** | 0.003(0.004)*** | 0.008(0.007)*** | 0.013(0.006)*** | 0.004(0.004)*** |
| year2010age2 | 0.039(0.002)*** | -0.002(0.005)*** | 0.018(0.007)*** | 0.031(0.006)*** | 0.006(0.004)*** |
| year2011age2 | 0.041(0.002)*** | 0.003(0.005)*** | 0.024(0.007)*** | 0.031(0.006)*** | 0.008(0.004)*** |
| year2012age2 | 0.042(0.002)*** | 0.004(0.004)*** | 0.029(0.007)*** | 0.035(0.006)*** | 0.007(0.004)*** |
| year2013age2 | 0.043(0.002)*** | 0.003(0.004)*** | 0.03(0.007)*** | 0.031(0.006)*** | 0.005(0.004)*** |
| year2014age2 | 0.041(0.002)*** | 0.004(0.004)*** | 0.02(0.007)*** | 0.025(0.006)*** | 0.006(0.004)*** |
| year2015age2 | 0.043(0.002)*** | 0.016(0.004)*** | 0.023(0.007)*** | 0.027(0.006)*** | 0.007(0.004)*** |
| year2016age2 | 0.043(0.002)*** | 0.006(0.004)*** | 0.027(0.007)*** | 0.035(0.006)*** | 0.008(0.004)*** |
| year2017age2 | 0.045(0.002)*** | 0.021(0.004)*** | 0.037(0.007)*** | 0.049(0.006)*** | 0.014(0.004)*** |
| year2018age2 | 0.043(0.002)*** | 0.004(0.004)*** | 0.031(0.007)*** | 0.037(0.006)*** | 0.011(0.004)*** |
| year2019age2 | 0.044(0.002)*** | 0.011(0.004)*** | 0.047(0.007)*** | 0.051(0.006)*** | 0.018(0.004)*** |
| year1990age3 | -0.002(0.003) | -0.003(0.005) | -0.014(0.006) | 0.001(0.007) | 0.002(0.006) |
| year1991age3 | -0.003(0.003) | -0.001(0.005) | -0.019(0.006) | -0.01(0.007) | 0.004(0.006) |
| year1992age3 | -0.005(0.003) | -0.008(0.005) | -0.028(0.006) | -0.027(0.007) | -0.005(0.006) |
| year1993age3 | -0.003(0.003) | -0.003(0.005) | -0.032(0.006) | -0.026(0.007) | -0.005(0.006) |
| year1994age3 | -0.006(0.003)* | -0.004(0.005)* | -0.023(0.006)* | -0.009(0.007)* | -0.002(0.005)* |
| year1995age3 | -0.006(0.003)* | -0.011(0.005)* | -0.029(0.006)* | -0.018(0.007)* | -0.006(0.005)* |
| year1996age3 | -0.007(0.003)* | -0.014(0.005)* | -0.033(0.006)* | -0.024(0.007)* | -0.013(0.005)* |
| year1997age3 | -0.014(0.004)*** | -0.017(0.005)*** | -0.042(0.006)*** | -0.027(0.007)*** | -0.017(0.005)*** |
| year1998age3 | -0.009(0.003)** | -0.013(0.005)** | -0.035(0.006)** | -0.04(0.007)** | -0.022(0.005)** |
| year1999age3 | -0.004(0.003) | -0.008(0.005) | -0.042(0.006) | -0.041(0.007) | -0.026(0.005) |
| year2000age3 | -0.003(0.003) | -0.004(0.005) | -0.038(0.006) | -0.033(0.008) | -0.018(0.005) |
| year2001age3 | -0.001(0.003) | -0.003(0.005) | -0.035(0.006) | -0.034(0.008) | -0.015(0.005) |
| year2002age3 | -0.01(0.003)** | -0.01(0.005)** | -0.04(0.006)** | -0.036(0.007)** | -0.015(0.005)** |
| Covariate | tau=0.1 | tau=0.25 | tau=0.5 | tau=0.75 | tau=0.9 |
| year2003age3 | -0.014(0.003)*** | -0.032(0.006)*** | -0.051(0.006)*** | -0.041(0.007)*** | -0.021(0.005)*** |
| year2004age3 | -0.012(0.003)*** | -0.033(0.006)*** | -0.035(0.007)*** | -0.037(0.007)*** | -0.016(0.005)*** |
| year2005age3 | -0.007(0.003)* | -0.034(0.006)* | -0.046(0.007)* | -0.035(0.007)* | -0.015(0.005)* |
| year2006age3 | -0.006(0.003) | -0.047(0.006) | -0.067(0.008) | -0.044(0.007) | -0.018(0.005) |
| year2007age3 | 0.001(0.003) | -0.033(0.007) | -0.047(0.007) | -0.031(0.007) | -0.013(0.005) |
| year2008age3 | 0.001(0.003) | -0.022(0.007) | -0.044(0.007) | -0.022(0.007) | -0.011(0.005) |
| year2009age3 | 0.009(0.004)* | -0.009(0.007)* | -0.035(0.007)* | -0.017(0.007)* | -0.008(0.005)* |
| year2010age3 | 0.002(0.003) | -0.024(0.008) | -0.033(0.007) | -0.013(0.007) | -0.01(0.005) |
| year2011age3 | 0.007(0.003)* | -0.017(0.007)* | -0.028(0.008)* | -0.01(0.007)* | -0.005(0.005)* |
| year2012age3 | 0.007(0.003)* | -0.013(0.007)* | -0.021(0.007)* | -0.006(0.007)* | -0.005(0.005)* |
| year2013age3 | 0.008(0.003)* | -0.013(0.007)* | -0.026(0.007)* | -0.001(0.007)* | -0.004(0.005)* |
| year2014age3 | 0.002(0.003) | -0.027(0.007) | -0.03(0.008) | -0.013(0.007) | -0.003(0.005) |
| year2015age3 | 0.005(0.003) | -0.018(0.007) | -0.025(0.007) | -0.012(0.007) | -0.002(0.005) |
| year2016age3 | 0.005(0.003) | -0.027(0.007) | -0.034(0.007) | -0.011(0.007) | -0.006(0.005) |
| year2017age3 | 0.022(0.003)*** | 0.008(0.007)*** | 0(0.007)*** | 0.005(0.007)*** | -0.001(0.005)*** |
| year2018age3 | 0.007(0.003)* | -0.012(0.007)* | -0.016(0.007)* | -0.01(0.007)* | -0.006(0.005)* |
| year2019age3 | 0.009(0.003)* | -0.007(0.007)* | -0.01(0.007)* | 0.009(0.007)* | 0.004(0.005)* |

Supplementary Table 3.4: Coefficient estimates (standard errors) for different quantiles (tau) for TSF, girls; **p<*0.05, ***p<*0.01,****p<*0.001, age2=10-12 years, age3=13-15 years.

| Covariate | tau=0.1 | tau=0.25 | tau=0.5 | tau=0.75 | tau=0.9 |
| --- | --- | --- | --- | --- | --- |
| (Intercept) | 0.09(0.002)*** | 0.209(0.002)*** | 0.422(0.003)*** | 0.681(0.004)*** | 0.87(0.003)*** |
| year1990 | -0.003(0.002) | -0.006(0.002) | -0.005(0.003) | -0.015(0.005) | -0.008(0.004) |
| year1991 | 0.002(0.002) | 0(0.002) | 0.002(0.003) | -0.002(0.005) | -0.006(0.004) |
| year1992 | 0.003(0.002) | 0.002(0.002) | 0.004(0.003) | -0.009(0.005) | -0.008(0.004) |
| year1993 | -0.003(0.002) | 0.002(0.002) | 0.01(0.003) | 0.017(0.005) | 0.008(0.004) |
| year1994 | 0.003(0.002) | 0.007(0.002) | 0.017(0.003) | 0.024(0.005) | 0.016(0.004) |
| year1995 | 0.003(0.002) | 0.004(0.002) | 0.017(0.003) | 0.03(0.005) | 0.018(0.003) |
| year1996 | 0.001(0.002) | 0.006(0.002) | 0.02(0.003) | 0.038(0.005) | 0.025(0.003) |
| year1997 | 0.003(0.002) | 0.01(0.002) | 0.026(0.003) | 0.04(0.005) | 0.022(0.004) |
| year1998 | 0.003(0.002) | 0.011(0.002) | 0.03(0.004) | 0.047(0.005) | 0.034(0.003) |
| year1999 | 0.005(0.002)* | 0.012(0.002)* | 0.041(0.005)* | 0.056(0.005)* | 0.038(0.003)* |
| year2000 | 0.007(0.002)** | 0.022(0.003)** | 0.062(0.005)** | 0.07(0.005)** | 0.041(0.003)** |
| year2001 | 0.011(0.002)*** | 0.026(0.003)*** | 0.073(0.004)*** | 0.083(0.005)*** | 0.049(0.003)*** |
| year2002 | 0.02(0.002)*** | 0.047(0.005)*** | 0.087(0.004)*** | 0.093(0.005)*** | 0.051(0.003)*** |
| year2003 | 0.026(0.002)*** | 0.068(0.004)*** | 0.101(0.004)*** | 0.106(0.005)*** | 0.06(0.003)*** |
| year2004 | 0.03(0.002)*** | 0.082(0.003)*** | 0.119(0.004)*** | 0.124(0.005)*** | 0.066(0.003)*** |
| year2005 | 0.03(0.003)*** | 0.086(0.003)*** | 0.128(0.004)*** | 0.127(0.005)*** | 0.07(0.003)*** |
| year2006 | 0.041(0.003)*** | 0.097(0.002)*** | 0.139(0.004)*** | 0.142(0.005)*** | 0.074(0.003)*** |
| year2007 | 0.042(0.003)*** | 0.095(0.003)*** | 0.137(0.004)*** | 0.134(0.005)*** | 0.072(0.003)*** |
| year2008 | 0.038(0.003)*** | 0.095(0.002)*** | 0.136(0.004)*** | 0.138(0.005)*** | 0.074(0.003)*** |
| year2009 | 0.046(0.003)*** | 0.099(0.002)*** | 0.137(0.004)*** | 0.136(0.005)*** | 0.071(0.003)*** |
| year2010 | 0.04(0.003)*** | 0.095(0.003)*** | 0.139(0.004)*** | 0.143(0.005)*** | 0.074(0.003)*** |
| year2011 | 0.041(0.003)*** | 0.095(0.003)*** | 0.137(0.004)*** | 0.137(0.005)*** | 0.071(0.003)*** |
| year2012 | 0.041(0.003)*** | 0.096(0.002)*** | 0.135(0.004)*** | 0.135(0.005)*** | 0.073(0.003)*** |
| year2013 | 0.042(0.003)*** | 0.098(0.002)*** | 0.14(0.004)*** | 0.143(0.005)*** | 0.075(0.003)*** |
| year2014 | 0.038(0.002)*** | 0.092(0.002)*** | 0.133(0.004)*** | 0.133(0.005)*** | 0.072(0.003)*** |
| year2015 | 0.041(0.003)*** | 0.093(0.002)*** | 0.132(0.004)*** | 0.128(0.004)*** | 0.071(0.003)*** |
| year2016 | 0.04(0.003)*** | 0.093(0.002)*** | 0.135(0.004)*** | 0.135(0.005)*** | 0.073(0.003)*** |
| year2017 | 0.042(0.003)*** | 0.091(0.002)*** | 0.129(0.004)*** | 0.128(0.004)*** | 0.07(0.003)*** |
| year2018 | 0.038(0.002)*** | 0.091(0.002)*** | 0.132(0.003)*** | 0.131(0.005)*** | 0.072(0.003)*** |
| year2019 | 0.036(0.002)*** | 0.091(0.002)*** | 0.132(0.004)*** | 0.128(0.004)*** | 0.07(0.003)*** |
| age2 | -0.003(0.002) | 0.01(0.002) | 0.001(0.003) | 0.022(0.005) | 0.009(0.004) |
| age3 | 0.003(0.004) | 0.018(0.005) | 0.048(0.004) | 0.045(0.006) | 0.023(0.004) |
| region1 | -0.012(0.001)*** | -0.014(0.001)*** | -0.033(0.002)*** | -0.034(0.002)*** | -0.02(0.001)*** |
| region2 | 0.011(0.001)*** | 0.007(0.001)*** | 0.004(0.003)*** | -0.018(0.002)*** | -0.017(0.001)*** |
| region3 | -0.012(0.001)*** | -0.015(0.001)*** | -0.034(0.002)*** | -0.033(0.002)*** | -0.021(0.001)*** |
| region4 | 0.046(0.002)*** | 0.068(0.002)*** | 0.073(0.003)*** | 0.052(0.002)*** | 0.017(0.001)*** |
| region5 | 0.011(0.001)*** | 0.014(0.002)*** | 0.022(0.003)*** | 0.011(0.002)*** | 0.001(0.001)*** |
| region6 | 0.002(0.001)* | 0.003(0.001)* | 0.007(0.002)* | -0.001(0.002)* | -0.005(0.001)* |
| region7 | -0.004(0.001)*** | -0.008(0.001)*** | -0.028(0.002)*** | -0.033(0.002)*** | -0.021(0.001)*** |
| region8 | -0.001(0.001) | -0.007(0.001) | -0.029(0.002) | -0.038(0.002) | -0.026(0.001) |
| Covariate | tau=0.1 | tau=0.25 | tau=0.5 | tau=0.75 | tau=0.9 |
| region9 | 0.024(0.002)*** | 0.025(0.003)*** | 0.033(0.003)*** | 0.014(0.002)*** | 0(0.001)*** |
| region10 | 0.001(0.001) | -0.001(0.001) | -0.018(0.002) | -0.019(0.002) | -0.011(0.001) |
| region11 | 0.011(0.001)*** | 0.011(0.001)*** | 0.031(0.002)*** | 0.024(0.002)*** | 0.007(0.001)*** |
| year1990age2 | 0.002(0.003) | 0.001(0.003) | 0.002(0.004) | 0(0.007) | 0.005(0.005) |
| year1991age2 | -0.002(0.003) | -0.002(0.003) | 0.008(0.005) | 0.009(0.006) | 0.006(0.005) |
| year1992age2 | -0.006(0.002)* | -0.008(0.003)* | -0.012(0.004)* | -0.031(0.006)* | -0.004(0.005)* |
| year1993age2 | 0.003(0.003) | -0.005(0.003) | -0.012(0.004) | -0.018(0.006) | -0.008(0.005) |
| year1994age2 | -0.001(0.002) | -0.007(0.003) | -0.01(0.005) | -0.018(0.006) | -0.009(0.005) |
| year1995age2 | -0.001(0.003) | -0.005(0.003) | 0.003(0.006) | -0.023(0.006) | -0.011(0.005) |
| year1996age2 | -0.002(0.003) | -0.007(0.003) | 0.002(0.007) | -0.026(0.006) | -0.009(0.004) |
| year1997age2 | -0.004(0.003) | -0.011(0.003) | -0.014(0.006) | -0.031(0.006) | -0.021(0.005) |
| year1998age2 | -0.003(0.003) | -0.011(0.003) | 0.004(0.006) | -0.032(0.006) | -0.015(0.005) |
| year1999age2 | -0.002(0.003) | -0.009(0.003) | 0.01(0.006) | -0.028(0.007) | -0.016(0.004) |
| year2000age2 | 0(0.003) | -0.012(0.003) | 0.002(0.006) | -0.011(0.006) | -0.012(0.004) |
| year2001age2 | -0.002(0.003) | -0.01(0.004) | -0.002(0.005) | -0.021(0.006) | -0.009(0.004) |
| year2002age2 | -0.009(0.003)** | -0.03(0.005)** | -0.018(0.005)** | -0.036(0.006)** | -0.021(0.004)** |
| year2003age2 | 0.018(0.003)*** | -0.009(0.005)*** | -0.025(0.005)*** | -0.039(0.006)*** | -0.024(0.004)*** |
| year2004age2 | 0.017(0.003)*** | -0.012(0.004)*** | -0.017(0.007)*** | -0.046(0.006)*** | -0.02(0.004)*** |
| year2005age2 | 0.017(0.003)*** | -0.015(0.003)*** | -0.021(0.007)*** | -0.025(0.006)*** | -0.018(0.004)*** |
| year2006age2 | 0.01(0.003)** | -0.018(0.003)** | 0.001(0.005)** | -0.025(0.006)** | -0.012(0.004)** |
| year2007age2 | 0.01(0.004)** | -0.016(0.003)** | 0.002(0.005)** | -0.021(0.006)** | -0.01(0.004)** |
| year2008age2 | 0.014(0.003)*** | -0.017(0.003)*** | 0.006(0.005)*** | -0.02(0.006)*** | -0.008(0.004)*** |
| year2009age2 | 0.01(0.004)** | -0.018(0.003)** | 0.009(0.005)** | -0.017(0.006)** | -0.007(0.004)** |
| year2010age2 | 0.018(0.003)*** | -0.01(0.003)*** | 0.02(0.005)*** | -0.004(0.006)*** | -0.003(0.004)*** |
| year2011age2 | 0.017(0.003)*** | -0.008(0.003)*** | 0.022(0.005)*** | 0.004(0.006)*** | -0.001(0.004)*** |
| year2012age2 | 0.019(0.003)*** | -0.011(0.003)*** | 0.021(0.005)*** | -0.01(0.006)*** | -0.006(0.004)*** |
| year2013age2 | 0.013(0.003)*** | -0.015(0.003)*** | 0.014(0.005)*** | -0.012(0.007)*** | -0.005(0.004)*** |
| year2014age2 | 0.015(0.003)*** | -0.012(0.003)*** | 0.017(0.005)*** | -0.01(0.006)*** | -0.004(0.004)*** |
| year2015age2 | 0.012(0.003)*** | -0.014(0.003)*** | 0.015(0.005)*** | -0.004(0.006)*** | -0.003(0.004)*** |
| year2016age2 | 0.017(0.003)*** | -0.01(0.003)*** | 0.02(0.005)*** | 0.001(0.006)*** | -0.003(0.004)*** |
| year2017age2 | 0.012(0.003)*** | -0.012(0.003)*** | 0.015(0.005)*** | -0.008(0.006)*** | -0.002(0.004)*** |
| year2018age2 | 0.015(0.003)*** | -0.012(0.003)*** | 0.011(0.005)*** | -0.01(0.006)*** | -0.005(0.004)*** |
| year2019age2 | 0.019(0.003)*** | -0.011(0.003)*** | 0.013(0.005)*** | -0.004(0.006)*** | -0.004(0.004)*** |
| year1990age3 | -0.003(0.004) | -0.005(0.006) | -0.009(0.006) | 0.004(0.007) | 0.002(0.006) |
| year1991age3 | 0(0.004) | -0.006(0.006) | -0.008(0.006) | -0.004(0.008) | 0.002(0.005) |
| year1992age3 | -0.009(0.004)* | -0.014(0.006)* | -0.028(0.006)* | -0.022(0.007)* | -0.017(0.006)* |
| year1993age3 | -0.004(0.004) | -0.019(0.006) | -0.038(0.006) | -0.041(0.007) | -0.021(0.005) |
| year1994age3 | -0.006(0.004) | -0.016(0.006) | -0.035(0.006) | -0.044(0.007) | -0.029(0.005) |
| year1995age3 | -0.007(0.004) | -0.018(0.006) | -0.041(0.006) | -0.05(0.007) | -0.03(0.005) |
| year1996age3 | -0.004(0.004) | -0.013(0.006) | -0.034(0.006) | -0.055(0.007) | -0.035(0.005) |
| year1997age3 | -0.008(0.004) | -0.016(0.006) | -0.036(0.006) | -0.054(0.007) | -0.031(0.005) |
| year1998age3 | -0.01(0.004)* | -0.022(0.006)* | -0.045(0.006)* | -0.058(0.007)* | -0.039(0.005)* |
| year1999age3 | -0.011(0.004)* | -0.023(0.006)* | -0.053(0.007)* | -0.062(0.007)* | -0.045(0.005)* |
| year2000age3 | -0.002(0.004) | -0.023(0.006) | -0.059(0.007) | -0.066(0.008) | -0.042(0.005) |
| year2001age3 | -0.011(0.004)* | -0.021(0.006)* | -0.066(0.006)* | -0.068(0.008)* | -0.044(0.005)* |
| Covariate | tau=0.1 | tau=0.25 | tau=0.5 | tau=0.75 | tau=0.9 |
| year2002age3 | -0.015(0.004)*** | -0.036(0.007)*** | -0.077(0.006)*** | -0.074(0.008)*** | -0.042(0.005)*** |
| year2003age3 | -0.014(0.005)** | -0.042(0.007)** | -0.073(0.007)** | -0.072(0.007)** | -0.046(0.005)** |
| year2004age3 | -0.008(0.005) | -0.041(0.007) | -0.065(0.007) | -0.075(0.007) | -0.041(0.005) |
| year2005age3 | -0.001(0.005) | -0.037(0.006) | -0.067(0.007) | -0.062(0.007) | -0.034(0.005) |
| year2006age3 | -0.004(0.005) | -0.035(0.006) | -0.053(0.006) | -0.062(0.007) | -0.033(0.005) |
| year2007age3 | -0.006(0.005) | -0.034(0.006) | -0.051(0.006) | -0.05(0.007) | -0.028(0.005) |
| year2008age3 | -0.001(0.005) | -0.027(0.006) | -0.037(0.007) | -0.047(0.007) | -0.028(0.005) |
| year2009age3 | 0.004(0.005) | -0.019(0.007) | -0.027(0.007) | -0.039(0.007) | -0.024(0.005) |
| year2010age3 | 0.011(0.005)* | -0.017(0.007)* | -0.031(0.007)* | -0.041(0.007)* | -0.021(0.005)* |
| year2011age3 | 0.017(0.006)** | 0.003(0.008)** | -0.008(0.007)** | -0.023(0.007)** | -0.015(0.005)** |
| year2012age3 | 0.024(0.006)*** | 0.003(0.007)*** | -0.008(0.007)*** | -0.024(0.007)*** | -0.017(0.005)*** |
| year2013age3 | 0.003(0.005) | -0.007(0.008) | -0.013(0.007) | -0.027(0.007) | -0.017(0.005) |
| year2014age3 | 0.007(0.005) | -0.021(0.007) | -0.018(0.007) | -0.026(0.007) | -0.014(0.005) |
| year2015age3 | 0.015(0.005)** | -0.009(0.007)** | -0.008(0.007)** | -0.02(0.007)** | -0.017(0.005)** |
| year2016age3 | 0.01(0.005)* | -0.012(0.007)* | -0.017(0.007)* | -0.029(0.007)* | -0.016(0.005)* |
| year2017age3 | 0.001(0.005) | -0.02(0.006) | -0.022(0.007) | -0.019(0.007) | -0.012(0.005) |
| year2018age3 | 0.004(0.005) | -0.02(0.007) | -0.029(0.007) | -0.023(0.007) | -0.016(0.005) |
| year2019age3 | 0.011(0.005)* | -0.02(0.006)* | -0.026(0.007)* | -0.025(0.007)* | -0.015(0.005)* |

Supplementary Table 3.5: Coefficient estimates (standard errors) for different quantiles (tau) for APT, boys; **p<*0.05, ***p<*0.01,****p<*0.001, age2=10-12 years, age3=13-15 years.

| Covariate | tau=0.1 | tau=0.25 | tau=0.5 | tau=0.75 | tau=0.9 |
| --- | --- | --- | --- | --- | --- |
| (Intercept) | 0.058(0.002)*** | 0.166(0.002)*** | 0.39(0.003)*** | 0.665(0.003)*** | 0.856(0.003)*** |
| year1990 | 0.014(0.002)*** | 0.031(0.003)*** | 0.048(0.004)*** | 0.043(0.004)*** | 0.035(0.003)*** |
| year1991 | 0.01(0.002)*** | 0.024(0.003)*** | 0.04(0.004)*** | 0.044(0.004)*** | 0.032(0.003)*** |
| year1992 | 0.014(0.002)*** | 0.032(0.003)*** | 0.055(0.004)*** | 0.064(0.004)*** | 0.04(0.003)*** |
| year1993 | 0.008(0.002)*** | 0.02(0.003)*** | 0.047(0.004)*** | 0.053(0.004)*** | 0.037(0.003)*** |
| year1994 | 0.013(0.002)*** | 0.032(0.003)*** | 0.062(0.004)*** | 0.063(0.004)*** | 0.045(0.003)*** |
| year1995 | 0.016(0.002)*** | 0.032(0.003)*** | 0.051(0.004)*** | 0.059(0.004)*** | 0.042(0.003)*** |
| year1996 | 0.008(0.002)*** | 0.015(0.003)*** | 0.028(0.004)*** | 0.036(0.004)*** | 0.03(0.003)*** |
| year1997 | 0.006(0.002)** | 0.006(0.003)** | 0.009(0.004)** | 0.014(0.004)** | 0.014(0.003)** |
| year1998 | 0.005(0.002)* | 0.009(0.003)* | 0.018(0.004)* | 0.014(0.004)* | 0.014(0.003)* |
| year1999 | 0.006(0.002)** | 0.008(0.003)** | 0.011(0.004)** | 0.008(0.004)** | 0.012(0.003)** |
| year2000 | 0.009(0.002)*** | 0.009(0.003)*** | 0.013(0.004)*** | 0.008(0.005)*** | 0.008(0.003)*** |
| year2001 | 0.009(0.002)*** | 0.015(0.003)*** | 0.016(0.004)*** | 0.014(0.004)*** | 0.012(0.003)*** |
| year2002 | 0.017(0.002)*** | 0.032(0.003)*** | 0.037(0.004)*** | 0.031(0.004)*** | 0.016(0.003)*** |
| year2003 | 0.017(0.002)*** | 0.028(0.003)*** | 0.036(0.004)*** | 0.027(0.004)*** | 0.011(0.003)*** |
| year2004 | 0.026(0.002)*** | 0.048(0.003)*** | 0.062(0.004)*** | 0.044(0.004)*** | 0.023(0.003)*** |
| year2005 | 0.036(0.002)*** | 0.068(0.004)*** | 0.085(0.004)*** | 0.069(0.004)*** | 0.035(0.003)*** |
| year2006 | 0.04(0.002)*** | 0.079(0.003)*** | 0.094(0.004)*** | 0.074(0.004)*** | 0.036(0.003)*** |
| year2007 | 0.041(0.002)*** | 0.077(0.004)*** | 0.094(0.004)*** | 0.073(0.004)*** | 0.036(0.003)*** |
| year2008 | 0.045(0.002)*** | 0.085(0.004)*** | 0.11(0.004)*** | 0.082(0.004)*** | 0.036(0.003)*** |
| year2009 | 0.049(0.002)*** | 0.089(0.004)*** | 0.111(0.004)*** | 0.085(0.004)*** | 0.04(0.003)*** |
| year2010 | 0.057(0.003)*** | 0.101(0.004)*** | 0.126(0.004)*** | 0.094(0.004)*** | 0.046(0.003)*** |
| year2011 | 0.063(0.003)*** | 0.109(0.004)*** | 0.131(0.004)*** | 0.095(0.004)*** | 0.046(0.003)*** |
| year2012 | 0.067(0.003)*** | 0.118(0.004)*** | 0.142(0.004)*** | 0.097(0.004)*** | 0.047(0.003)*** |
| year2013 | 0.078(0.003)*** | 0.135(0.004)*** | 0.165(0.004)*** | 0.119(0.004)*** | 0.056(0.003)*** |
| year2014 | 0.087(0.003)*** | 0.151(0.004)*** | 0.177(0.004)*** | 0.128(0.004)*** | 0.061(0.003)*** |
| year2015 | 0.08(0.003)*** | 0.144(0.004)*** | 0.173(0.004)*** | 0.122(0.004)*** | 0.055(0.003)*** |
| year2016 | 0.09(0.003)*** | 0.154(0.004)*** | 0.185(0.004)*** | 0.131(0.004)*** | 0.062(0.003)*** |
| year2017 | 0.087(0.003)*** | 0.152(0.004)*** | 0.18(0.004)*** | 0.126(0.004)*** | 0.06(0.003)*** |
| year2018 | 0.087(0.003)*** | 0.154(0.004)*** | 0.179(0.004)*** | 0.121(0.004)*** | 0.054(0.003)*** |
| year2019 | 0.086(0.003)*** | 0.151(0.004)*** | 0.183(0.004)*** | 0.125(0.004)*** | 0.057(0.003)*** |
| age2 | -0.012(0.002)*** | -0.019(0.003)*** | -0.02(0.004)*** | -0.009(0.004)*** | -0.005(0.004)*** |
| age3 | -0.004(0.002) | -0.002(0.004) | 0.001(0.005) | 0.018(0.005) | 0.013(0.004) |
| region1 | 0.009(0.001)*** | 0.018(0.001)*** | 0.027(0.002)*** | 0.023(0.001)*** | 0.016(0.001)*** |
| region2 | 0.023(0.001)*** | 0.037(0.002)*** | 0.047(0.002)*** | 0.042(0.002)*** | 0.028(0.001)*** |
| region3 | 0.018(0.001)*** | 0.036(0.001)*** | 0.045(0.002)*** | 0.034(0.001)*** | 0.02(0.001)*** |
| region4 | 0.005(0.001)*** | -0.008(0.002)*** | -0.031(0.003)*** | -0.049(0.002)*** | -0.042(0.002)*** |
| region5 | 0.038(0.001)*** | 0.061(0.002)*** | 0.069(0.002)*** | 0.047(0.002)*** | 0.021(0.001)*** |
| region6 | 0.016(0.001)*** | 0.027(0.002)*** | 0.028(0.002)*** | 0.018(0.002)*** | 0.006(0.001)*** |
| region7 | 0.015(0.001)*** | 0.019(0.001)*** | 0.016(0.002)*** | 0.004(0.001)*** | -0.001(0.001)*** |
| region8 | 0.01(0.001)*** | 0.014(0.001)*** | 0.01(0.002)*** | 0.003(0.002)*** | -0.001(0.001)*** |
| region9 | 0.022(0.001)*** | 0.038(0.002)*** | 0.04(0.002)*** | 0.021(0.002)*** | 0.009(0.001)*** |
| Covariate | tau=0.1 | tau=0.25 | tau=0.5 | tau=0.75 | tau=0.9 |
| region10 | 0.013(0.001)*** | 0.019(0.002)*** | 0.015(0.002)*** | 0(0.002)*** | -0.006(0.001)*** |
| region11 | 0.005(0.001)*** | 0.005(0.002)*** | -0.003(0.002)*** | -0.011(0.002)*** | -0.01(0.001)*** |
| year1990age2 | 0(0.003) | 0.004(0.004) | 0.013(0.006) | 0.009(0.006) | 0.001(0.004) |
| year1991age2 | 0.026(0.003)*** | 0.048(0.005)*** | 0.069(0.006)*** | 0.045(0.006)*** | 0.016(0.004)*** |
| year1992age2 | 0.042(0.003)*** | 0.088(0.005)*** | 0.109(0.006)*** | 0.062(0.006)*** | 0.029(0.004)*** |
| year1993age2 | 0.04(0.003)*** | 0.085(0.005)*** | 0.105(0.006)*** | 0.064(0.006)*** | 0.027(0.004)*** |
| year1994age2 | 0.035(0.003)*** | 0.075(0.005)*** | 0.095(0.006)*** | 0.064(0.006)*** | 0.029(0.004)*** |
| year1995age2 | 0.026(0.003)*** | 0.06(0.005)*** | 0.092(0.006)*** | 0.057(0.006)*** | 0.026(0.004)*** |
| year1996age2 | 0.031(0.003)*** | 0.07(0.005)*** | 0.106(0.006)*** | 0.076(0.006)*** | 0.033(0.004)*** |
| year1997age2 | 0.024(0.003)*** | 0.056(0.004)*** | 0.087(0.006)*** | 0.063(0.006)*** | 0.031(0.005)*** |
| year1998age2 | 0.029(0.003)*** | 0.059(0.004)*** | 0.078(0.006)*** | 0.061(0.006)*** | 0.028(0.005)*** |
| year1999age2 | 0.031(0.003)*** | 0.065(0.004)*** | 0.079(0.006)*** | 0.063(0.006)*** | 0.029(0.005)*** |
| year2000age2 | 0.025(0.003)*** | 0.056(0.004)*** | 0.071(0.006)*** | 0.057(0.006)*** | 0.028(0.005)*** |
| year2001age2 | 0.02(0.003)*** | 0.039(0.004)*** | 0.056(0.006)*** | 0.037(0.006)*** | 0.014(0.005)*** |
| year2002age2 | 0.015(0.003)*** | 0.025(0.005)*** | 0.04(0.006)*** | 0.022(0.006)*** | 0.011(0.005)*** |
| year2003age2 | 0.016(0.003)*** | 0.03(0.005)*** | 0.038(0.006)*** | 0.025(0.006)*** | 0.016(0.005)*** |
| year2004age2 | 0.01(0.003)** | 0.019(0.005)** | 0.028(0.006)** | 0.017(0.006)** | 0.006(0.005)** |
| year2005age2 | -0.001(0.003) | -0.002(0.005) | 0.001(0.006) | -0.006(0.006) | -0.004(0.005) |
| year2006age2 | 0.002(0.003) | -0.002(0.005) | 0.005(0.006) | -0.008(0.006) | -0.004(0.005) |
| year2007age2 | 0.002(0.003) | -0.005(0.005) | -0.01(0.006) | -0.021(0.006) | -0.015(0.005) |
| year2008age2 | -0.002(0.003) | -0.012(0.005) | -0.025(0.006) | -0.024(0.006) | -0.01(0.005) |
| year2009age2 | -0.006(0.003) | -0.013(0.005) | -0.017(0.006) | -0.025(0.006) | -0.014(0.005) |
| year2010age2 | -0.01(0.004)** | -0.014(0.005)** | -0.022(0.006)** | -0.028(0.006)** | -0.018(0.004)** |
| year2011age2 | -0.016(0.004)*** | -0.024(0.005)*** | -0.03(0.006)*** | -0.03(0.006)*** | -0.02(0.004)*** |
| year2012age2 | -0.017(0.004)*** | -0.03(0.005)*** | -0.037(0.006)*** | -0.035(0.006)*** | -0.018(0.004)*** |
| year2013age2 | -0.016(0.004)*** | -0.025(0.005)*** | -0.037(0.006)*** | -0.029(0.006)*** | -0.014(0.004)*** |
| year2014age2 | -0.022(0.004)*** | -0.041(0.005)*** | -0.04(0.006)*** | -0.037(0.005)*** | -0.019(0.004)*** |
| year2015age2 | -0.014(0.004)*** | -0.026(0.005)*** | -0.022(0.006)*** | -0.021(0.005)*** | -0.008(0.004)*** |
| year2016age2 | -0.02(0.004)*** | -0.025(0.005)*** | -0.037(0.006)*** | -0.035(0.005)*** | -0.018(0.004)*** |
| year2017age2 | -0.021(0.004)*** | -0.034(0.005)*** | -0.041(0.006)*** | -0.031(0.005)*** | -0.02(0.004)*** |
| year2018age2 | -0.022(0.004)*** | -0.037(0.005)*** | -0.037(0.006)*** | -0.029(0.005)*** | -0.013(0.004)*** |
| year2019age2 | -0.017(0.004)*** | -0.033(0.005)*** | -0.045(0.006)*** | -0.033(0.005)*** | -0.017(0.004)*** |
| year1990age3 | 0.006(0.003) | 0.013(0.005) | 0.012(0.007) | -0.003(0.007) | -0.012(0.005) |
| year1991age3 | 0.014(0.003)*** | 0.023(0.005)*** | 0.029(0.007)*** | 0.007(0.007)*** | -0.002(0.005)*** |
| year1992age3 | 0.022(0.004)*** | 0.044(0.006)*** | 0.05(0.007)*** | 0.024(0.007)*** | 0.01(0.005)*** |
| year1993age3 | 0.042(0.004)*** | 0.088(0.005)*** | 0.093(0.007)*** | 0.054(0.007)*** | 0.018(0.005)*** |
| year1994age3 | 0.045(0.004)*** | 0.084(0.005)*** | 0.095(0.007)*** | 0.051(0.007)*** | 0.011(0.005)*** |
| year1995age3 | 0.041(0.004)*** | 0.081(0.005)*** | 0.102(0.007)*** | 0.056(0.007)*** | 0.017(0.005)*** |
| year1996age3 | 0.039(0.004)*** | 0.086(0.005)*** | 0.113(0.007)*** | 0.07(0.007)*** | 0.026(0.005)*** |
| year1997age3 | 0.029(0.003)*** | 0.074(0.005)*** | 0.095(0.007)*** | 0.057(0.007)*** | 0.021(0.005)*** |
| year1998age3 | 0.029(0.004)*** | 0.053(0.005)*** | 0.075(0.007)*** | 0.053(0.007)*** | 0.02(0.005)*** |
| year1999age3 | 0.029(0.003)*** | 0.053(0.005)*** | 0.07(0.007)*** | 0.048(0.007)*** | 0.014(0.005)*** |
| year2000age3 | 0.025(0.004)*** | 0.056(0.005)*** | 0.076(0.007)*** | 0.048(0.007)*** | 0.018(0.005)*** |
| year2001age3 | 0.024(0.004)*** | 0.047(0.005)*** | 0.059(0.007)*** | 0.027(0.007)*** | 0.006(0.005)*** |
| year2002age3 | 0.015(0.004)*** | 0.03(0.005)*** | 0.038(0.007)*** | 0.01(0.007)*** | 0.003(0.005)*** |
| Covariate | tau=0.1 | tau=0.25 | tau=0.5 | tau=0.75 | tau=0.9 |
| year2003age3 | 0.016(0.004)*** | 0.023(0.005)*** | 0.03(0.007)*** | 0.005(0.007)*** | 0(0.005)*** |
| Covariate | tau=0.1 | tau=0.25 | tau=0.5 | tau=0.75 | tau=0.9 |
| year2004age3 | 0.001(0.004) | 0.004(0.006) | 0.01(0.007) | -0.008(0.007) | -0.01(0.005) |
| year2005age3 | -0.007(0.004) | -0.014(0.006) | -0.011(0.007) | -0.028(0.007) | -0.018(0.005) |
| year2006age3 | -0.006(0.004) | -0.021(0.006) | -0.02(0.007) | -0.036(0.007) | -0.016(0.005) |
| year2007age3 | -0.01(0.004)* | -0.024(0.006)* | -0.035(0.007)* | -0.048(0.007)* | -0.024(0.005)* |
| year2008age3 | -0.017(0.004)*** | -0.039(0.006)*** | -0.061(0.007)*** | -0.057(0.007)*** | -0.029(0.005)*** |
| year2009age3 | -0.02(0.004)*** | -0.041(0.006)*** | -0.057(0.007)*** | -0.063(0.007)*** | -0.039(0.005)*** |
| year2010age3 | -0.028(0.004)*** | -0.049(0.006)*** | -0.071(0.007)*** | -0.079(0.007)*** | -0.045(0.005)*** |
| year2011age3 | -0.032(0.004)*** | -0.056(0.006)*** | -0.071(0.007)*** | -0.072(0.007)*** | -0.046(0.005)*** |
| year2012age3 | -0.033(0.004)*** | -0.065(0.006)*** | -0.084(0.007)*** | -0.074(0.007)*** | -0.044(0.005)*** |
| year2013age3 | -0.035(0.004)*** | -0.066(0.006)*** | -0.088(0.007)*** | -0.084(0.007)*** | -0.048(0.005)*** |
| year2014age3 | -0.045(0.004)*** | -0.082(0.006)*** | -0.099(0.007)*** | -0.098(0.007)*** | -0.056(0.005)*** |
| year2015age3 | -0.031(0.004)*** | -0.063(0.006)*** | -0.082(0.007)*** | -0.081(0.007)*** | -0.047(0.005)*** |
| year2016age3 | -0.042(0.004)*** | -0.078(0.006)*** | -0.101(0.007)*** | -0.095(0.007)*** | -0.057(0.005)*** |
| year2017age3 | -0.043(0.004)*** | -0.076(0.006)*** | -0.101(0.007)*** | -0.097(0.007)*** | -0.062(0.005)*** |
| year2018age3 | -0.047(0.004)*** | -0.078(0.006)*** | -0.104(0.007)*** | -0.097(0.007)*** | -0.054(0.005)*** |
| year2019age3 | -0.047(0.004)*** | -0.087(0.006)*** | -0.109(0.007)*** | -0.098(0.007)*** | -0.057(0.005)*** |

Supplementary Table 3.6: Coefficient estimates (standard errors) for different quantiles (tau) for APT, girls; **p<*0.05, ***p<*0.01,****p<*0.001, age2=10-12 years, age3=13-15 years.

| Covariates | tau=0.1 | tau=0.25 | tau=0.5 | tau=0.75 | tau=0.9 |
| --- | --- | --- | --- | --- | --- |
| (Intercept) | 0.052(0.002)*** | 0.151(0.002)*** | 0.371(0.003)*** | 0.646(0.003)*** | 0.844(0.003)*** |
| year1990 | 0.018(0.002)*** | 0.04(0.003)*** | 0.061(0.004)*** | 0.057(0.004)*** | 0.037(0.003)*** |
| year1991 | 0.013(0.002)*** | 0.031(0.003)*** | 0.051(0.004)*** | 0.046(0.004)*** | 0.033(0.003)*** |
| year1992 | 0.019(0.002)*** | 0.043(0.003)*** | 0.076(0.004)*** | 0.066(0.004)*** | 0.045(0.003)*** |
| year1993 | 0.01(0.002)*** | 0.025(0.003)*** | 0.045(0.004)*** | 0.048(0.004)*** | 0.038(0.003)*** |
| year1994 | 0.014(0.002)*** | 0.042(0.003)*** | 0.076(0.004)*** | 0.081(0.004)*** | 0.046(0.003)*** |
| year1995 | 0.012(0.002)*** | 0.029(0.003)*** | 0.052(0.004)*** | 0.055(0.004)*** | 0.039(0.003)*** |
| year1996 | 0.001(0.002) | 0.011(0.003) | 0.022(0.004) | 0.029(0.004) | 0.023(0.003) |
| year1997 | 0.002(0.002) | 0.004(0.003) | 0.014(0.004) | 0.02(0.004) | 0.017(0.003) |
| year1998 | -0.001(0.002) | -0.002(0.003) | -0.004(0.004) | 0.004(0.004) | 0.005(0.004) |
| year1999 | 0(0.002) | -0.003(0.003) | -0.007(0.004) | -0.003(0.005) | -0.003(0.004) |
| year2000 | 0.001(0.002) | 0.001(0.003) | 0.003(0.004) | 0.002(0.004) | 0.002(0.004) |
| year2001 | 0.006(0.002)** | 0.013(0.003)** | 0.018(0.004)** | 0.02(0.004)** | 0.009(0.003)** |
| year2002 | 0.015(0.002)*** | 0.028(0.003)*** | 0.042(0.004)*** | 0.036(0.004)*** | 0.02(0.003)*** |
| year2003 | 0.013(0.002)*** | 0.024(0.003)*** | 0.035(0.004)*** | 0.024(0.004)*** | 0.014(0.003)*** |
| year2004 | 0.02(0.002)*** | 0.045(0.004)*** | 0.072(0.004)*** | 0.054(0.004)*** | 0.029(0.003)*** |
| year2005 | 0.029(0.002)*** | 0.062(0.004)*** | 0.086(0.004)*** | 0.071(0.004)*** | 0.037(0.003)*** |
| year2006 | 0.039(0.002)*** | 0.077(0.004)*** | 0.1(0.004)*** | 0.075(0.004)*** | 0.04(0.003)*** |
| year2007 | 0.036(0.002)*** | 0.075(0.004)*** | 0.1(0.004)*** | 0.075(0.004)*** | 0.036(0.003)*** |
| year2008 | 0.039(0.003)*** | 0.084(0.004)*** | 0.109(0.004)*** | 0.083(0.004)*** | 0.041(0.003)*** |
| year2009 | 0.04(0.002)*** | 0.082(0.004)*** | 0.104(0.004)*** | 0.083(0.004)*** | 0.039(0.003)*** |
| year2010 | 0.053(0.003)*** | 0.099(0.004)*** | 0.121(0.004)*** | 0.093(0.004)*** | 0.045(0.003)*** |
| year2011 | 0.056(0.003)*** | 0.111(0.004)*** | 0.142(0.004)*** | 0.1(0.004)*** | 0.051(0.003)*** |
| year2012 | 0.064(0.003)*** | 0.124(0.004)*** | 0.149(0.004)*** | 0.107(0.004)*** | 0.05(0.003)*** |
| year2013 | 0.07(0.003)*** | 0.125(0.004)*** | 0.155(0.004)*** | 0.116(0.004)*** | 0.057(0.003)*** |
| year2014 | 0.078(0.003)*** | 0.145(0.004)*** | 0.178(0.004)*** | 0.13(0.004)*** | 0.066(0.003)*** |
| year2015 | 0.077(0.003)*** | 0.142(0.004)*** | 0.177(0.004)*** | 0.129(0.004)*** | 0.062(0.003)*** |
| year2016 | 0.088(0.003)*** | 0.166(0.004)*** | 0.198(0.004)*** | 0.141(0.004)*** | 0.07(0.003)*** |
| year2017 | 0.087(0.003)*** | 0.161(0.004)*** | 0.187(0.004)*** | 0.135(0.004)*** | 0.064(0.003)*** |
| year2018 | 0.084(0.003)*** | 0.149(0.004)*** | 0.172(0.004)*** | 0.122(0.004)*** | 0.055(0.003)*** |
| year2019 | 0.082(0.003)*** | 0.148(0.004)*** | 0.173(0.004)*** | 0.119(0.004)*** | 0.053(0.003)*** |
| age2 | -0.027(0.002)*** | -0.052(0.003)*** | -0.069(0.004)*** | -0.053(0.005)*** | -0.026(0.004)*** |
| age3 | -0.017(0.002)*** | -0.026(0.004)*** | -0.038(0.005)*** | -0.034(0.006)*** | -0.006(0.005)*** |
| region1 | 0.01(0.001)*** | 0.018(0.001)*** | 0.022(0.002)*** | 0.023(0.002)*** | 0.017(0.001)*** |
| region2 | 0.032(0.001)*** | 0.054(0.002)*** | 0.067(0.002)*** | 0.06(0.002)*** | 0.039(0.001)*** |
| region3 | 0.03(0.001)*** | 0.054(0.001)*** | 0.066(0.002)*** | 0.051(0.002)*** | 0.029(0.001)*** |
| region4 | 0.019(0.001)*** | 0.017(0.002)*** | -0.001(0.003)*** | -0.023(0.002)*** | -0.028(0.002)*** |
| region5 | 0.054(0.001)*** | 0.092(0.002)*** | 0.105(0.002)*** | 0.077(0.002)*** | 0.036(0.001)*** |
| region6 | 0.026(0.001)*** | 0.045(0.002)*** | 0.048(0.002)*** | 0.033(0.002)*** | 0.013(0.001)*** |
| region7 | 0.025(0.001)*** | 0.038(0.001)*** | 0.037(0.002)*** | 0.025(0.001)*** | 0.01(0.001)*** |
| region8 | 0.021(0.001)*** | 0.036(0.001)*** | 0.037(0.002)*** | 0.025(0.002)*** | 0.01(0.001)*** |
| region9 | 0.048(0.002)*** | 0.083(0.002)*** | 0.094(0.003)*** | 0.063(0.002)*** | 0.029(0.001)*** |
| region10 | 0.035(0.001)*** | 0.059(0.002)*** | 0.064(0.002)*** | 0.042(0.002)*** | 0.016(0.001)*** |
| Covariate | tau=0.1 | tau=0.25 | tau=0.5 | tau=0.75 | tau=0.9 |
| region11 | 0.007(0.001)*** | 0.007(0.002)*** | 0(0.002)*** | -0.001(0.002)*** | -0.001(0.001)*** |
| year1990age2 | 0.001(0.003) | 0.007(0.004) | 0.012(0.006) | 0.003(0.006) | 0.001(0.005) |
| year1991age2 | 0.028(0.003)*** | 0.054(0.004)*** | 0.073(0.006)*** | 0.055(0.006)*** | 0.021(0.005)*** |
| year1992age2 | 0.04(0.003)*** | 0.084(0.005)*** | 0.106(0.006)*** | 0.085(0.006)*** | 0.037(0.005)*** |
| year1993age2 | 0.045(0.003)*** | 0.095(0.004)*** | 0.127(0.006)*** | 0.097(0.006)*** | 0.041(0.005)*** |
| year1994age2 | 0.051(0.003)*** | 0.094(0.005)*** | 0.118(0.006)*** | 0.077(0.006)*** | 0.04(0.005)*** |
| year1995age2 | 0.042(0.003)*** | 0.088(0.005)*** | 0.118(0.006)*** | 0.091(0.006)*** | 0.041(0.005)*** |
| year1996age2 | 0.048(0.003)*** | 0.093(0.004)*** | 0.132(0.006)*** | 0.102(0.006)*** | 0.05(0.005)*** |
| year1997age2 | 0.038(0.003)*** | 0.081(0.004)*** | 0.113(0.006)*** | 0.081(0.006)*** | 0.035(0.005)*** |
| year1998age2 | 0.041(0.003)*** | 0.088(0.004)*** | 0.128(0.006)*** | 0.097(0.007)*** | 0.049(0.005)*** |
| year1999age2 | 0.043(0.003)*** | 0.09(0.004)*** | 0.126(0.006)*** | 0.099(0.007)*** | 0.054(0.005)*** |
| year2000age2 | 0.042(0.003)*** | 0.09(0.005)*** | 0.124(0.006)*** | 0.1(0.007)*** | 0.054(0.005)*** |
| year2001age2 | 0.036(0.003)*** | 0.073(0.005)*** | 0.108(0.006)*** | 0.076(0.007)*** | 0.042(0.005)*** |
| year2002age2 | 0.033(0.003)*** | 0.073(0.005)*** | 0.095(0.006)*** | 0.069(0.007)*** | 0.033(0.005)*** |
| year2003age2 | 0.033(0.003)*** | 0.075(0.005)*** | 0.102(0.006)*** | 0.083(0.007)*** | 0.037(0.005)*** |
| year2004age2 | 0.033(0.003)*** | 0.063(0.005)*** | 0.077(0.006)*** | 0.059(0.007)*** | 0.03(0.005)*** |
| year2005age2 | 0.024(0.003)*** | 0.046(0.005)*** | 0.062(0.006)*** | 0.043(0.007)*** | 0.02(0.005)*** |
| year2006age2 | 0.021(0.003)*** | 0.042(0.005)*** | 0.065(0.006)*** | 0.054(0.006)*** | 0.024(0.005)*** |
| year2007age2 | 0.016(0.003)*** | 0.032(0.005)*** | 0.047(0.006)*** | 0.038(0.006)*** | 0.019(0.005)*** |
| year2008age2 | 0.017(0.003)*** | 0.032(0.005)*** | 0.045(0.006)*** | 0.03(0.007)*** | 0.014(0.005)*** |
| year2009age2 | 0.016(0.003)*** | 0.029(0.005)*** | 0.043(0.006)*** | 0.033(0.006)*** | 0.017(0.005)*** |
| year2010age2 | 0.005(0.004) | 0.017(0.005) | 0.039(0.006) | 0.029(0.006) | 0.017(0.005) |
| year2011age2 | 0.006(0.004) | 0.018(0.005) | 0.028(0.006) | 0.024(0.006) | 0.01(0.005) |
| year2012age2 | 0.001(0.004) | 0.01(0.005) | 0.025(0.006) | 0.024(0.006) | 0.011(0.005) |
| year2013age2 | 0.011(0.004)** | 0.032(0.005)** | 0.048(0.006)** | 0.035(0.006)** | 0.014(0.005)** |
| year2014age2 | 0.017(0.004)*** | 0.035(0.006)*** | 0.047(0.006)*** | 0.036(0.006)*** | 0.012(0.005)*** |
| year2015age2 | 0.026(0.004)*** | 0.049(0.005)*** | 0.064(0.006)*** | 0.047(0.006)*** | 0.027(0.005)*** |
| year2016age2 | 0.023(0.004)*** | 0.039(0.005)*** | 0.05(0.006)*** | 0.042(0.006)*** | 0.018(0.005)*** |
| year2017age2 | 0.013(0.004)*** | 0.032(0.005)*** | 0.05(0.006)*** | 0.037(0.006)*** | 0.02(0.005)*** |
| year2018age2 | 0.012(0.004)** | 0.026(0.005)** | 0.048(0.006)** | 0.036(0.006)** | 0.02(0.005)** |
| year2019age2 | 0.01(0.004)** | 0.026(0.005)** | 0.037(0.006)** | 0.034(0.006)** | 0.021(0.005)** |
| year1990age3 | -0.001(0.003) | -0.004(0.005) | -0.011(0.007) | -0.002(0.007) | -0.004(0.006) |
| year1991age3 | 0.01(0.003)** | 0.01(0.005)** | 0.011(0.007)** | 0.021(0.007)** | 0.003(0.006)** |
| year1992age3 | 0.015(0.003)*** | 0.036(0.006)*** | 0.043(0.007)*** | 0.048(0.007)*** | 0.015(0.006)*** |
| year1993age3 | 0.04(0.003)*** | 0.074(0.005)*** | 0.095(0.007)*** | 0.084(0.007)*** | 0.027(0.006)*** |
| year1994age3 | 0.045(0.003)*** | 0.075(0.005)*** | 0.091(0.007)*** | 0.068(0.007)*** | 0.026(0.006)*** |
| year1995age3 | 0.045(0.003)*** | 0.086(0.005)*** | 0.118(0.007)*** | 0.093(0.007)*** | 0.032(0.006)*** |
| year1996age3 | 0.053(0.003)*** | 0.096(0.005)*** | 0.135(0.007)*** | 0.113(0.007)*** | 0.049(0.006)*** |
| year1997age3 | 0.038(0.003)*** | 0.08(0.005)*** | 0.102(0.007)*** | 0.082(0.007)*** | 0.029(0.006)*** |
| year1998age3 | 0.039(0.003)*** | 0.08(0.005)*** | 0.118(0.007)*** | 0.101(0.007)*** | 0.04(0.006)*** |
| year1999age3 | 0.041(0.003)*** | 0.079(0.005)*** | 0.117(0.007)*** | 0.097(0.008)*** | 0.039(0.006)*** |
| year2000age3 | 0.043(0.003)*** | 0.079(0.005)*** | 0.111(0.007)*** | 0.092(0.007)*** | 0.036(0.006)*** |
| year2001age3 | 0.036(0.003)*** | 0.068(0.005)*** | 0.093(0.007)*** | 0.078(0.007)*** | 0.03(0.006)*** |
| year2002age3 | 0.026(0.004)*** | 0.054(0.006)*** | 0.076(0.007)*** | 0.068(0.007)*** | 0.024(0.006)*** |
| year2003age3 | 0.029(0.004)*** | 0.058(0.006)*** | 0.076(0.007)*** | 0.068(0.007)*** | 0.021(0.006)*** |
| Covariate | tau=0.1 | tau=0.25 | tau=0.5 | tau=0.75 | tau=0.9 |
| year2004age3 | 0.025(0.004)*** | 0.038(0.006)*** | 0.039(0.007)*** | 0.041(0.007)*** | 0.009(0.006)*** |
| year2005age3 | 0.01(0.004)** | 0.019(0.006)** | 0.029(0.007)** | 0.024(0.007)** | 0.005(0.006)** |
| year2006age3 | 0.01(0.004)* | 0.012(0.006)* | 0.025(0.007)* | 0.028(0.008)* | 0.004(0.006)* |
| year2007age3 | 0.009(0.004)* | 0.007(0.006)* | 0.008(0.007)* | 0.014(0.007)* | -0.001(0.006)* |
| year2008age3 | 0.002(0.004) | -0.013(0.006) | -0.009(0.007) | -0.006(0.007) | -0.01(0.006) |
| year2009age3 | 0.001(0.004) | -0.003(0.006) | -0.002(0.007) | 0.001(0.007) | -0.012(0.006) |
| year2010age3 | -0.012(0.004)** | -0.019(0.006)** | -0.012(0.007)** | -0.01(0.007)** | -0.012(0.006)** |
| year2011age3 | -0.014(0.004)*** | -0.022(0.006)*** | -0.031(0.007)*** | -0.012(0.007)*** | -0.023(0.006)*** |
| year2012age3 | -0.018(0.004)*** | -0.034(0.006)*** | -0.03(0.007)*** | -0.011(0.007)*** | -0.015(0.006)*** |
| year2013age3 | -0.012(0.004)** | -0.011(0.006)** | -0.012(0.007)** | -0.006(0.007)** | -0.018(0.006)** |
| year2014age3 | -0.018(0.004)*** | -0.019(0.006)*** | -0.019(0.007)*** | -0.008(0.007)*** | -0.021(0.006)*** |
| year2015age3 | 0.001(0.005) | 0(0.006) | 0.007(0.007) | 0.007(0.007) | -0.009(0.006) |
| year2016age3 | 0.002(0.005) | -0.012(0.007) | -0.013(0.007) | -0.003(0.007) | -0.017(0.006) |
| year2017age3 | -0.008(0.005) | -0.016(0.006) | -0.005(0.007) | 0.001(0.007) | -0.01(0.006) |
| year2018age3 | -0.007(0.005) | -0.009(0.006) | 0.007(0.007) | 0.005(0.007) | -0.01(0.006) |
| year2019age3 | -0.01(0.005)* | -0.009(0.006)* | -0.007(0.007)* | 0.002(0.007)* | -0.007(0.006)* |

Supplementary Table 3.7: Coefficient estimates (standard errors) for different quantiles (tau) for SBJ, boys; **p<*0.05, ***p<*0.01,****p<*0.001, age2=10-12 years, age3=13-15 years.

| Covarite | tau=0.1 | tau=0.25 | tau=0.5 | tau=0.75 | tau=0.9 |
| --- | --- | --- | --- | --- | --- |
| (Intercept) | 0.12(0.002)*** | 0.276(0.003)*** | 0.511(0.003)*** | 0.74(0.002)*** | 0.886(0.002)*** |
| year1990 | -0.006(0.003) | -0.016(0.004) | -0.018(0.004) | -0.012(0.003) | -0.007(0.002) |
| year1991 | -0.02(0.003)*** | -0.029(0.004)*** | -0.029(0.004)*** | -0.014(0.003)*** | -0.007(0.002)*** |
| year1992 | -0.017(0.003)*** | -0.022(0.004)*** | -0.018(0.004)*** | -0.011(0.003)*** | -0.003(0.002)*** |
| year1993 | -0.032(0.003)*** | -0.059(0.004)*** | -0.053(0.004)*** | -0.036(0.003)*** | -0.018(0.002)*** |
| year1994 | -0.026(0.003)*** | -0.05(0.004)*** | -0.046(0.004)*** | -0.026(0.003)*** | -0.01(0.002)*** |
| year1995 | -0.024(0.003)*** | -0.037(0.004)*** | -0.033(0.004)*** | -0.017(0.003)*** | -0.007(0.002)*** |
| year1996 | -0.029(0.003)*** | -0.043(0.004)*** | -0.041(0.004)*** | -0.022(0.003)*** | -0.009(0.002)*** |
| year1997 | -0.024(0.003)*** | -0.034(0.004)*** | -0.026(0.004)*** | -0.012(0.003)*** | -0.004(0.002)*** |
| year1998 | -0.016(0.003)*** | -0.023(0.004)*** | -0.013(0.004)*** | -0.001(0.003)*** | 0.002(0.002)*** |
| year1999 | -0.016(0.003)*** | -0.024(0.004)*** | -0.016(0.004)*** | -0.005(0.003)*** | 0(0.002)*** |
| year2000 | -0.024(0.003)*** | -0.039(0.004)*** | -0.035(0.004)*** | -0.014(0.003)*** | -0.006(0.002)*** |
| year2001 | -0.032(0.003)*** | -0.055(0.004)*** | -0.056(0.004)*** | -0.033(0.003)*** | -0.015(0.002)*** |
| year2002 | -0.036(0.003)*** | -0.059(0.004)*** | -0.054(0.004)*** | -0.032(0.003)*** | -0.015(0.002)*** |
| year2003 | -0.041(0.003)*** | -0.07(0.004)*** | -0.065(0.004)*** | -0.041(0.003)*** | -0.021(0.002)*** |
| year2004 | -0.043(0.003)*** | -0.066(0.004)*** | -0.063(0.004)*** | -0.038(0.003)*** | -0.016(0.002)*** |
| year2005 | -0.043(0.003)*** | -0.076(0.004)*** | -0.08(0.004)*** | -0.049(0.003)*** | -0.021(0.002)*** |
| year2006 | -0.052(0.003)*** | -0.089(0.004)*** | -0.089(0.004)*** | -0.053(0.004)*** | -0.022(0.002)*** |
| year2007 | -0.066(0.003)*** | -0.113(0.004)*** | -0.116(0.005)*** | -0.07(0.004)*** | -0.03(0.003)*** |
| year2008 | -0.069(0.003)*** | -0.116(0.004)*** | -0.122(0.004)*** | -0.083(0.004)*** | -0.036(0.003)*** |
| year2009 | -0.069(0.003)*** | -0.118(0.004)*** | -0.127(0.004)*** | -0.081(0.004)*** | -0.036(0.003)*** |
| year2010 | -0.073(0.003)*** | -0.122(0.004)*** | -0.135(0.004)*** | -0.089(0.004)*** | -0.04(0.003)*** |
| year2011 | -0.076(0.003)*** | -0.135(0.004)*** | -0.154(0.004)*** | -0.112(0.004)*** | -0.055(0.003)*** |
| year2012 | -0.073(0.003)*** | -0.126(0.004)*** | -0.14(0.004)*** | -0.099(0.004)*** | -0.048(0.003)*** |
| year2013 | -0.078(0.003)*** | -0.14(0.004)*** | -0.159(0.004)*** | -0.116(0.004)*** | -0.059(0.003)*** |
| year2014 | -0.078(0.003)*** | -0.137(0.004)*** | -0.16(0.004)*** | -0.107(0.004)*** | -0.054(0.003)*** |
| year2015 | -0.079(0.003)*** | -0.137(0.004)*** | -0.153(0.004)*** | -0.107(0.004)*** | -0.053(0.003)*** |
| year2016 | -0.081(0.003)*** | -0.14(0.004)*** | -0.151(0.004)*** | -0.101(0.004)*** | -0.049(0.003)*** |
| year2017 | -0.08(0.003)*** | -0.136(0.003)*** | -0.156(0.004)*** | -0.105(0.004)*** | -0.054(0.003)*** |
| year2018 | -0.081(0.003)*** | -0.141(0.003)*** | -0.158(0.004)*** | -0.103(0.004)*** | -0.051(0.003)*** |
| year2019 | -0.081(0.003)*** | -0.137(0.003)*** | -0.152(0.004)*** | -0.107(0.003)*** | -0.053(0.003)*** |
| age2 | 0.016(0.003)*** | -0.001(0.004)*** | -0.018(0.004)*** | -0.014(0.003)*** | -0.008(0.002)*** |
| age3 | -0.016(0.004)*** | -0.052(0.005)*** | -0.066(0.005)*** | -0.053(0.004)*** | -0.032(0.003)*** |
| region1 | 0.016(0.001)*** | 0.029(0.001)*** | 0.035(0.002)*** | 0.029(0.002)*** | 0.02(0.001)*** |
| region2 | 0.057(0.001)*** | 0.107(0.002)*** | 0.134(0.002)*** | 0.106(0.002)*** | 0.062(0.001)*** |
| region3 | 0.025(0.001)*** | 0.047(0.001)*** | 0.063(0.002)*** | 0.053(0.002)*** | 0.031(0.001)*** |
| region4 | 0.014(0.001)*** | 0.018(0.002)*** | 0.012(0.003)*** | 0.001(0.003)*** | -0.001(0.002)*** |
| region5 | 0.02(0.001)*** | 0.039(0.002)*** | 0.052(0.002)*** | 0.045(0.002)*** | 0.026(0.002)*** |
| region6 | 0.023(0.001)*** | 0.048(0.001)*** | 0.063(0.002)*** | 0.054(0.002)*** | 0.032(0.001)*** |
| region7 | 0.039(0.001)*** | 0.072(0.001)*** | 0.087(0.001)*** | 0.068(0.001)*** | 0.039(0.001)*** |
| region8 | 0.057(0.001)*** | 0.103(0.001)*** | 0.121(0.002)*** | 0.092(0.002)*** | 0.052(0.001)*** |
| region9 | 0.03(0.001)*** | 0.057(0.002)*** | 0.076(0.002)*** | 0.059(0.002)*** | 0.035(0.002)*** |
| region10 | 0.057(0.001)*** | 0.102(0.002)*** | 0.125(0.002)*** | 0.099(0.002)*** | 0.055(0.001)*** |
| Covariate | tau=0.1 | tau=0.25 | tau=0.5 | tau=0.75 | tau=0.9 |
| region11 | 0.024(0.001)*** | 0.041(0.002)*** | 0.047(0.002)*** | 0.035(0.002)*** | 0.018(0.001)*** |
| year1990age2 | 0.007(0.005) | 0.025(0.005) | 0.026(0.005) | 0.019(0.004) | 0.011(0.003) |
| year1991age2 | 0.018(0.005)*** | 0.032(0.005)*** | 0.04(0.005)*** | 0.02(0.004)*** | 0.015(0.003)*** |
| year1992age2 | 0.02(0.005)*** | 0.036(0.005)*** | 0.038(0.005)*** | 0.032(0.004)*** | 0.017(0.003)*** |
| year1993age2 | 0.011(0.004)* | 0.04(0.005)* | 0.038(0.005)* | 0.028(0.004)* | 0.017(0.003)* |
| year1994age2 | 0.001(0.004) | 0.028(0.005) | 0.035(0.005) | 0.023(0.004) | 0.013(0.003) |
| year1995age2 | 0.004(0.004) | 0.021(0.005) | 0.024(0.005) | 0.013(0.004) | 0.008(0.003) |
| year1996age2 | -0.001(0.004) | 0.016(0.005) | 0.025(0.006) | 0.019(0.004) | 0.01(0.003) |
| year1997age2 | -0.005(0.005) | 0.01(0.005) | 0.017(0.006) | 0.009(0.004) | 0.007(0.003) |
| year1998age2 | -0.013(0.005)** | 0.005(0.006)** | 0.008(0.005)** | 0.004(0.004)** | 0.005(0.003)** |
| year1999age2 | -0.01(0.005)* | 0.002(0.005)* | 0.007(0.005)* | 0.005(0.004)* | 0.004(0.003)* |
| year2000age2 | -0.014(0.005)** | 0.002(0.005)** | 0.013(0.006)** | 0.007(0.004)** | 0.007(0.003)** |
| year2001age2 | -0.019(0.004)*** | -0.006(0.006)*** | 0.001(0.006)*** | 0.003(0.005)*** | 0.004(0.003)*** |
| year2002age2 | -0.019(0.004)*** | -0.014(0.006)*** | -0.01(0.006)*** | -0.01(0.005)*** | 0.001(0.003)*** |
| year2003age2 | -0.022(0.004)*** | -0.007(0.005)*** | -0.007(0.006)*** | -0.005(0.005)*** | 0.002(0.003)*** |
| year2004age2 | -0.019(0.004)*** | -0.008(0.005)*** | 0(0.006)*** | 0.003(0.005)*** | 0.002(0.003)*** |
| year2005age2 | -0.03(0.004)*** | -0.023(0.005)*** | -0.003(0.006)*** | 0.001(0.005)*** | 0(0.004)*** |
| year2006age2 | -0.025(0.004)*** | -0.006(0.005)*** | 0.005(0.006)*** | 0.006(0.005)*** | 0.004(0.004)*** |
| year2007age2 | -0.019(0.004)*** | 0(0.006)*** | 0.011(0.006)*** | 0.007(0.005)*** | 0.005(0.004)*** |
| year2008age2 | -0.018(0.004)*** | -0.006(0.005)*** | 0.01(0.006)*** | 0.019(0.005)*** | 0.015(0.004)*** |
| year2009age2 | -0.021(0.004)*** | -0.007(0.005)*** | 0.005(0.006)*** | 0.011(0.005)*** | 0.009(0.004)*** |
| year2010age2 | -0.022(0.004)*** | -0.014(0.005)*** | 0.002(0.006)*** | 0.009(0.006)*** | 0.01(0.004)*** |
| year2011age2 | -0.029(0.004)*** | -0.018(0.005)*** | -0.005(0.006)*** | 0.007(0.006)*** | 0.009(0.004)*** |
| year2012age2 | -0.029(0.004)*** | -0.024(0.005)*** | -0.01(0.006)*** | -0.004(0.006)*** | 0(0.004)*** |
| year2013age2 | -0.026(0.004)*** | -0.01(0.005)*** | 0.009(0.006)*** | 0.015(0.006)*** | 0.018(0.004)*** |
| year2014age2 | -0.028(0.004)*** | -0.015(0.005)*** | 0.001(0.006)*** | 0.001(0.006)*** | 0.01(0.004)*** |
| year2015age2 | -0.026(0.004)*** | -0.017(0.005)*** | -0.006(0.006)*** | 0(0.006)*** | 0.005(0.004)*** |
| year2016age2 | -0.028(0.004)*** | -0.022(0.005)*** | -0.02(0.006)*** | -0.013(0.005)*** | -0.005(0.004)*** |
| year2017age2 | -0.032(0.004)*** | -0.03(0.005)*** | -0.012(0.006)*** | -0.014(0.005)*** | -0.003(0.004)*** |
| year2018age2 | -0.03(0.004)*** | -0.02(0.005)*** | -0.002(0.006)*** | -0.003(0.005)*** | 0.006(0.004)*** |
| year2019age2 | -0.031(0.004)*** | -0.027(0.005)*** | -0.016(0.006)*** | -0.001(0.005)*** | 0.008(0.004)*** |
| year1990age3 | 0.02(0.005)*** | 0.043(0.006)*** | 0.05(0.006)*** | 0.034(0.005)*** | 0.023(0.004)*** |
| year1991age3 | 0.029(0.005)*** | 0.064(0.006)*** | 0.072(0.006)*** | 0.053(0.005)*** | 0.034(0.004)*** |
| year1992age3 | 0.035(0.005)*** | 0.073(0.006)*** | 0.077(0.006)*** | 0.063(0.005)*** | 0.039(0.004)*** |
| year1993age3 | 0.042(0.005)*** | 0.093(0.006)*** | 0.096(0.006)*** | 0.076(0.005)*** | 0.047(0.004)*** |
| year1994age3 | 0.034(0.005)*** | 0.08(0.006)*** | 0.087(0.006)*** | 0.061(0.005)*** | 0.04(0.004)*** |
| year1995age3 | 0.031(0.005)*** | 0.063(0.006)*** | 0.074(0.006)*** | 0.056(0.005)*** | 0.037(0.004)*** |
| year1996age3 | 0.025(0.005)*** | 0.067(0.006)*** | 0.078(0.006)*** | 0.06(0.005)*** | 0.038(0.004)*** |
| year1997age3 | 0.025(0.005)*** | 0.056(0.006)*** | 0.059(0.006)*** | 0.042(0.005)*** | 0.029(0.004)*** |
| year1998age3 | 0.011(0.005)* | 0.039(0.006)* | 0.039(0.006)* | 0.027(0.005)* | 0.022(0.004)* |
| year1999age3 | 0.008(0.005) | 0.035(0.006) | 0.038(0.006) | 0.03(0.005) | 0.021(0.004) |
| year2000age3 | 0.007(0.005) | 0.04(0.006) | 0.056(0.007) | 0.035(0.005) | 0.027(0.004) |
| year2001age3 | 0.009(0.005) | 0.036(0.006) | 0.049(0.007) | 0.039(0.006) | 0.026(0.004) |
| year2002age3 | 0.008(0.005) | 0.03(0.006) | 0.038(0.007) | 0.032(0.005) | 0.021(0.004) |
| year2003age3 | 0.003(0.005) | 0.026(0.006) | 0.031(0.007) | 0.026(0.006) | 0.02(0.004) |
| Covariate | tau=0.1 | tau=0.25 | tau=0.5 | tau=0.75 | tau=0.9 |
| year2004age3 | 0.002(0.005) | 0.023(0.006) | 0.038(0.007) | 0.036(0.006) | 0.023(0.004) |
| year2005age3 | -0.001(0.005) | 0.024(0.006) | 0.035(0.007) | 0.031(0.006) | 0.022(0.004) |
| year2006age3 | 0.005(0.005) | 0.036(0.006) | 0.051(0.007) | 0.043(0.006) | 0.024(0.004) |
| year2007age3 | 0.011(0.005)* | 0.047(0.006)* | 0.06(0.007)* | 0.049(0.006)* | 0.028(0.004)* |
| year2008age3 | 0.012(0.004)** | 0.046(0.006)** | 0.071(0.007)** | 0.062(0.006)** | 0.038(0.004)** |
| year2009age3 | 0.011(0.004)* | 0.055(0.006)* | 0.079(0.007)* | 0.062(0.006)* | 0.039(0.005)* |
| year2010age3 | 0.013(0.004)** | 0.05(0.006)** | 0.069(0.007)** | 0.063(0.006)** | 0.037(0.005)** |
| year2011age3 | 0.01(0.004)* | 0.041(0.006)* | 0.064(0.007)* | 0.068(0.007)* | 0.042(0.005)* |
| year2012age3 | 0.002(0.004) | 0.028(0.006) | 0.049(0.007) | 0.051(0.006) | 0.03(0.005) |
| year2013age3 | 0.005(0.004) | 0.041(0.006) | 0.076(0.007) | 0.073(0.007) | 0.048(0.005) |
| year2014age3 | 0.001(0.004) | 0.035(0.006) | 0.065(0.007) | 0.054(0.006) | 0.039(0.005) |
| year2015age3 | 0.006(0.004) | 0.04(0.006) | 0.063(0.007) | 0.05(0.006) | 0.038(0.005) |
| year2016age3 | 0.007(0.004) | 0.043(0.006) | 0.058(0.007) | 0.047(0.006) | 0.037(0.005) |
| year2017age3 | 0.006(0.004) | 0.036(0.006) | 0.065(0.007) | 0.053(0.007) | 0.04(0.005) |
| year2018age3 | 0.009(0.004)* | 0.044(0.006)* | 0.076(0.007)* | 0.059(0.006)* | 0.041(0.005)* |
| year2019age3 | 0.004(0.004) | 0.034(0.006) | 0.055(0.007) | 0.054(0.006) | 0.043(0.005) |

Supplementary Table 3.8: Coefficient estimates (standard errors) for different quantiles (tau) for SBJ, girls; **p<*0.05, ***p<*0.01,****p<*0.001, age2=10-12 years, age3=13-15 years.

| Covariate | tau=0.1 | tau=0.25 | tau=0.5 | tau=0.75 | tau=0.9 |
| --- | --- | --- | --- | --- | --- |
| (Intercept) | 0.114(0.002)*** | 0.262(0.003)*** | 0.5(0.003)*** | 0.736(0.003)*** | 0.885(0.002)*** |
| year1990 | 0.001(0.003) | 0.01(0.004) | 0.013(0.004) | 0.006(0.003) | 0.004(0.002) |
| year1991 | -0.01(0.003)** | -0.007(0.004)** | -0.004(0.004)** | -0.008(0.003)** | -0.001(0.002)** |
| year1992 | -0.001(0.003) | 0.001(0.004) | 0.006(0.004) | 0.003(0.003) | 0(0.002) |
| year1993 | -0.026(0.003)*** | -0.04(0.004)*** | -0.039(0.004)*** | -0.026(0.003)*** | -0.011(0.002)*** |
| year1994 | -0.023(0.003)*** | -0.036(0.004)*** | -0.034(0.004)*** | -0.023(0.003)*** | -0.009(0.002)*** |
| year1995 | -0.018(0.003)*** | -0.028(0.004)*** | -0.033(0.004)*** | -0.023(0.003)*** | -0.008(0.002)*** |
| year1996 | -0.03(0.003)*** | -0.046(0.004)*** | -0.042(0.004)*** | -0.028(0.003)*** | -0.009(0.002)*** |
| year1997 | -0.018(0.003)*** | -0.028(0.004)*** | -0.032(0.004)*** | -0.021(0.003)*** | -0.007(0.002)*** |
| year1998 | -0.02(0.003)*** | -0.027(0.004)*** | -0.034(0.004)*** | -0.023(0.003)*** | -0.008(0.002)*** |
| year1999 | -0.022(0.003)*** | -0.036(0.004)*** | -0.042(0.004)*** | -0.035(0.003)*** | -0.016(0.002)*** |
| year2000 | -0.031(0.003)*** | -0.048(0.004)*** | -0.053(0.004)*** | -0.037(0.003)*** | -0.017(0.002)*** |
| year2001 | -0.028(0.003)*** | -0.047(0.004)*** | -0.057(0.004)*** | -0.04(0.003)*** | -0.02(0.002)*** |
| year2002 | -0.028(0.003)*** | -0.045(0.004)*** | -0.056(0.004)*** | -0.042(0.003)*** | -0.016(0.002)*** |
| year2003 | -0.034(0.003)*** | -0.06(0.004)*** | -0.067(0.004)*** | -0.046(0.003)*** | -0.02(0.002)*** |
| year2004 | -0.037(0.003)*** | -0.058(0.004)*** | -0.071(0.004)*** | -0.044(0.004)*** | -0.019(0.002)*** |
| year2005 | -0.047(0.003)*** | -0.074(0.004)*** | -0.084(0.004)*** | -0.056(0.004)*** | -0.025(0.002)*** |
| year2006 | -0.05(0.003)*** | -0.084(0.004)*** | -0.097(0.004)*** | -0.069(0.004)*** | -0.026(0.003)*** |
| year2007 | -0.059(0.003)*** | -0.102(0.004)*** | -0.115(0.004)*** | -0.079(0.004)*** | -0.033(0.003)*** |
| year2008 | -0.065(0.003)*** | -0.111(0.004)*** | -0.128(0.004)*** | -0.086(0.004)*** | -0.034(0.003)*** |
| year2009 | -0.065(0.003)*** | -0.106(0.004)*** | -0.121(0.004)*** | -0.081(0.004)*** | -0.034(0.003)*** |
| year2010 | -0.066(0.003)*** | -0.108(0.004)*** | -0.126(0.004)*** | -0.088(0.004)*** | -0.04(0.003)*** |
| year2011 | -0.066(0.003)*** | -0.116(0.004)*** | -0.14(0.004)*** | -0.106(0.004)*** | -0.049(0.003)*** |
| year2012 | -0.061(0.003)*** | -0.102(0.004)*** | -0.12(0.004)*** | -0.081(0.004)*** | -0.034(0.003)*** |
| year2013 | -0.068(0.003)*** | -0.119(0.004)*** | -0.137(0.004)*** | -0.098(0.004)*** | -0.044(0.003)*** |
| year2014 | -0.07(0.003)*** | -0.121(0.004)*** | -0.135(0.004)*** | -0.091(0.004)*** | -0.039(0.003)*** |
| year2015 | -0.068(0.003)*** | -0.118(0.004)*** | -0.139(0.004)*** | -0.097(0.004)*** | -0.044(0.003)*** |
| year2016 | -0.075(0.003)*** | -0.129(0.004)*** | -0.148(0.004)*** | -0.107(0.004)*** | -0.048(0.003)*** |
| year2017 | -0.07(0.003)*** | -0.121(0.004)*** | -0.143(0.004)*** | -0.101(0.004)*** | -0.048(0.003)*** |
| year2018 | -0.076(0.003)*** | -0.127(0.004)*** | -0.146(0.004)*** | -0.107(0.004)*** | -0.047(0.003)*** |
| year2019 | -0.072(0.003)*** | -0.125(0.004)*** | -0.152(0.004)*** | -0.11(0.004)*** | -0.049(0.003)*** |
| age2 | 0.005(0.003) | -0.008(0.004) | -0.026(0.004) | -0.026(0.003) | -0.009(0.002) |
| age3 | -0.005(0.004) | -0.027(0.005) | -0.042(0.005) | -0.033(0.004) | -0.017(0.002) |
| region1 | 0.009(0.001)*** | 0.014(0.001)*** | 0.014(0.002)*** | 0.01(0.002)*** | 0.006(0.001)*** |
| region2 | 0.055(0.001)*** | 0.099(0.002)*** | 0.121(0.002)*** | 0.099(0.002)*** | 0.053(0.001)*** |
| region3 | 0.027(0.001)*** | 0.044(0.001)*** | 0.054(0.002)*** | 0.046(0.002)*** | 0.025(0.001)*** |
| region4 | 0.012(0.001)*** | 0.017(0.002)*** | 0.01(0.003)*** | 0(0.003)*** | -0.005(0.002)*** |
| region5 | 0.027(0.001)*** | 0.057(0.002)*** | 0.076(0.002)*** | 0.067(0.002)*** | 0.036(0.002)*** |
| region6 | 0.02(0.001)*** | 0.041(0.001)*** | 0.055(0.002)*** | 0.05(0.002)*** | 0.029(0.001)*** |
| region7 | 0.043(0.001)*** | 0.079(0.001)*** | 0.097(0.001)*** | 0.08(0.001)*** | 0.043(0.001)*** |
| region8 | 0.068(0.001)*** | 0.114(0.001)*** | 0.134(0.002)*** | 0.105(0.002)*** | 0.054(0.001)*** |
| region9 | 0.04(0.001)*** | 0.069(0.002)*** | 0.086(0.002)*** | 0.07(0.002)*** | 0.038(0.002)*** |
| Covariate | tau=0.1 | tau=0.25 | tau=0.5 | tau=0.75 | tau=0.9 |
| region10 | 0.081(0.001)*** | 0.139(0.002)*** | 0.166(0.002)*** | 0.131(0.002)*** | 0.069(0.001)*** |
| region11 | 0.036(0.001)*** | 0.068(0.002)*** | 0.085(0.002)*** | 0.072(0.002)*** | 0.04(0.001)*** |
| year1990age2 | 0.002(0.005) | -0.002(0.005) | -0.004(0.006) | 0.001(0.004) | -0.002(0.003) |
| year1991age2 | 0.016(0.005)*** | 0.017(0.006)*** | 0.018(0.006)*** | 0.02(0.004)*** | 0.003(0.003)*** |
| year1992age2 | 0.023(0.005)*** | 0.031(0.006)*** | 0.031(0.006)*** | 0.025(0.004)*** | 0.013(0.003)*** |
| year1993age2 | 0.028(0.004)*** | 0.043(0.005)*** | 0.043(0.006)*** | 0.032(0.004)*** | 0.013(0.003)*** |
| year1994age2 | 0.021(0.004)*** | 0.041(0.005)*** | 0.041(0.006)*** | 0.032(0.004)*** | 0.013(0.003)*** |
| year1995age2 | 0.015(0.004)*** | 0.031(0.006)*** | 0.041(0.006)*** | 0.032(0.005)*** | 0.014(0.003)*** |
| year1996age2 | 0.016(0.004)*** | 0.033(0.005)*** | 0.033(0.006)*** | 0.027(0.005)*** | 0.01(0.003)*** |
| year1997age2 | 0.007(0.004) | 0.016(0.005) | 0.027(0.006) | 0.024(0.005) | 0.01(0.003) |
| year1998age2 | 0.003(0.005) | 0.01(0.006) | 0.021(0.006) | 0.019(0.005) | 0.008(0.003) |
| year1999age2 | 0.003(0.004) | 0.011(0.006) | 0.021(0.006) | 0.026(0.005) | 0.012(0.003) |
| year2000age2 | 0(0.004) | 0.009(0.005) | 0.018(0.006) | 0.016(0.005) | 0.006(0.003) |
| year2001age2 | -0.011(0.004)** | -0.007(0.006)** | 0.008(0.006)** | 0.012(0.005)** | 0.007(0.003)** |
| year2002age2 | -0.012(0.004)** | -0.006(0.006)** | 0.008(0.006)** | 0.017(0.005)** | 0.004(0.003)** |
| year2003age2 | -0.012(0.004)** | 0.004(0.005)** | 0.013(0.006)** | 0.014(0.005)** | 0.003(0.003)** |
| year2004age2 | -0.009(0.004)* | 0.001(0.006)* | 0.027(0.006)* | 0.014(0.005)* | 0.005(0.004)* |
| year2005age2 | -0.007(0.004) | -0.005(0.006) | 0.011(0.006) | 0.01(0.005) | 0.004(0.004) |
| year2006age2 | -0.013(0.004)** | 0(0.006)** | 0.022(0.006)** | 0.024(0.005)** | 0.004(0.004)** |
| year2007age2 | -0.01(0.004)* | 0.006(0.005)* | 0.023(0.006)* | 0.024(0.005)* | 0.007(0.004)* |
| year2008age2 | -0.012(0.004)** | 0(0.005)** | 0.022(0.006)** | 0.025(0.006)** | 0.006(0.004)** |
| year2009age2 | -0.01(0.004)* | -0.005(0.005)* | 0.015(0.006)* | 0.017(0.006)* | 0.007(0.004)* |
| year2010age2 | -0.016(0.004)*** | -0.013(0.005)*** | 0.01(0.006)*** | 0.018(0.006)*** | 0.01(0.004)*** |
| year2011age2 | -0.02(0.004)*** | -0.018(0.005)*** | 0.006(0.006)*** | 0.021(0.006)*** | 0.008(0.004)*** |
| year2012age2 | -0.025(0.004)*** | -0.027(0.005)*** | -0.003(0.006)*** | 0.008(0.006)*** | 0.002(0.004)*** |
| year2013age2 | -0.018(0.004)*** | -0.01(0.005)*** | 0.005(0.006)*** | 0.017(0.006)*** | 0.008(0.004)*** |
| year2014age2 | -0.017(0.004)*** | -0.008(0.005)*** | 0.007(0.006)*** | 0.013(0.006)*** | 0.004(0.004)*** |
| year2015age2 | -0.017(0.004)*** | -0.006(0.005)*** | 0.018(0.006)*** | 0.023(0.006)*** | 0.014(0.004)*** |
| year2016age2 | -0.015(0.004)*** | -0.004(0.005)*** | 0.017(0.006)*** | 0.026(0.006)*** | 0.011(0.004)*** |
| year2017age2 | -0.022(0.004)*** | -0.015(0.005)*** | 0.011(0.006)*** | 0.028(0.005)*** | 0.014(0.004)*** |
| year2018age2 | -0.018(0.004)*** | -0.011(0.005)*** | 0.013(0.006)*** | 0.03(0.006)*** | 0.015(0.004)*** |
| year2019age2 | -0.023(0.004)*** | -0.016(0.005)*** | 0.014(0.006)*** | 0.033(0.006)*** | 0.017(0.004)*** |
| year1990age3 | 0.01(0.005) | 0.017(0.007) | 0.016(0.006) | 0.015(0.005) | 0.007(0.003) |
| year1991age3 | 0.027(0.005)*** | 0.038(0.007)*** | 0.032(0.006)*** | 0.032(0.005)*** | 0.014(0.003)*** |
| year1992age3 | 0.032(0.005)*** | 0.047(0.006)*** | 0.042(0.006)*** | 0.033(0.005)*** | 0.017(0.003)*** |
| year1993age3 | 0.042(0.005)*** | 0.07(0.006)*** | 0.062(0.006)*** | 0.044(0.005)*** | 0.021(0.003)*** |
| year1994age3 | 0.04(0.005)*** | 0.068(0.006)*** | 0.062(0.006)*** | 0.049(0.005)*** | 0.023(0.003)*** |
| year1995age3 | 0.039(0.005)*** | 0.072(0.006)*** | 0.077(0.006)*** | 0.058(0.005)*** | 0.028(0.003)*** |
| year1996age3 | 0.039(0.005)*** | 0.073(0.006)*** | 0.069(0.007)*** | 0.053(0.005)*** | 0.025(0.003)*** |
| year1997age3 | 0.015(0.005)** | 0.039(0.007)** | 0.045(0.007)** | 0.034(0.005)** | 0.019(0.003)** |
| year1998age3 | 0.006(0.005) | 0.022(0.007) | 0.036(0.007) | 0.024(0.006) | 0.012(0.003) |
| year1999age3 | 0.008(0.005) | 0.021(0.006) | 0.032(0.007) | 0.031(0.006) | 0.02(0.004) |
| year2000age3 | 0.014(0.005)** | 0.035(0.006)** | 0.038(0.007)** | 0.031(0.006)** | 0.019(0.004)** |
| year2001age3 | 0.003(0.005) | 0.019(0.006) | 0.03(0.007) | 0.017(0.006) | 0.016(0.004) |
| year2002age3 | -0.009(0.005) | -0.002(0.006) | 0.009(0.007) | 0.009(0.006) | 0.009(0.004) |
| Covariate | tau=0.1 | tau=0.25 | tau=0.5 | tau=0.75 | tau=0.9 |
| year2003age3 | -0.007(0.005) | 0.004(0.006) | 0.009(0.007) | 0.001(0.006) | 0.008(0.004) |
| year2004age3 | -0.012(0.005)* | -0.003(0.007)* | 0.012(0.007)* | 0.009(0.006)* | 0.011(0.004)* |
| year2005age3 | -0.009(0.005)* | 0.002(0.007)* | 0.007(0.007)* | 0.007(0.006)* | 0.008(0.004)* |
| year2006age3 | -0.008(0.005) | 0.01(0.007) | 0.022(0.007) | 0.02(0.007) | 0.011(0.004) |
| year2007age3 | -0.007(0.004) | 0.013(0.006) | 0.023(0.007) | 0.015(0.007) | 0.014(0.005) |
| year2008age3 | -0.004(0.004) | 0.017(0.006) | 0.031(0.007) | 0.022(0.007) | 0.01(0.005) |
| year2009age3 | -0.005(0.004) | 0.013(0.006) | 0.031(0.007) | 0.027(0.007) | 0.014(0.004) |
| year2010age3 | -0.011(0.004)* | -0.003(0.006)* | 0.02(0.007)* | 0.021(0.007)* | 0.014(0.005)* |
| year2011age3 | -0.022(0.004)*** | -0.014(0.006)*** | 0.009(0.007)*** | 0.017(0.007)*** | 0.014(0.005)*** |
| year2012age3 | -0.025(0.004)*** | -0.022(0.006)*** | -0.004(0.007)*** | 0.009(0.007)*** | 0.012(0.005)*** |
| year2013age3 | -0.017(0.004)*** | -0.007(0.006)*** | 0.015(0.007)*** | 0.017(0.007)*** | 0.012(0.005)*** |
| year2014age3 | -0.019(0.004)*** | -0.006(0.006)*** | 0.006(0.007)*** | 0.019(0.007)*** | 0.012(0.004)*** |
| year2015age3 | -0.017(0.004)*** | 0.001(0.006)*** | 0.02(0.007)*** | 0.031(0.007)*** | 0.026(0.004)*** |
| year2016age3 | -0.008(0.004)* | 0.016(0.006)* | 0.048(0.007)* | 0.055(0.007)* | 0.036(0.004)* |
| year2017age3 | -0.011(0.004)* | 0.014(0.006)* | 0.046(0.007)* | 0.049(0.007)* | 0.032(0.004)* |
| year2018age3 | -0.002(0.004) | 0.029(0.006) | 0.062(0.007) | 0.067(0.007) | 0.041(0.004) |
| year2019age3 | -0.008(0.004) | 0.023(0.006) | 0.066(0.007) | 0.068(0.007) | 0.043(0.004) |

Supplementary Table 3.9: Coefficient estimates (standard errors) for different quantiles (tau) for BOC, boys; **p<*0.05, ***p<*0.01,****p<*0.001, age2=10-12 years, age3=13-15 years.

| Covariate | tau=0.1 | tau=0.25 | tau=0.5 | tau=0.75 | tau=0.9 |
| --- | --- | --- | --- | --- | --- |
| (Intercept) | 0.158(0.002)*** | 0.347(0.003)*** | 0.613(0.003)*** | 0.826(0.002)*** | 0.938(0.002)*** |
| year1990 | -0.016(0.003)*** | -0.03(0.004)*** | -0.032(0.004)*** | -0.02(0.003)*** | -0.012(0.002)*** |
| year1991 | -0.019(0.003)*** | -0.032(0.004)*** | -0.032(0.004)*** | -0.02(0.003)*** | -0.012(0.002)*** |
| year1992 | -0.026(0.003)*** | -0.04(0.004)*** | -0.044(0.004)*** | -0.03(0.003)*** | -0.016(0.002)*** |
| year1993 | -0.018(0.003)*** | -0.028(0.004)*** | -0.03(0.004)*** | -0.019(0.003)*** | -0.006(0.002)*** |
| year1994 | -0.034(0.003)*** | -0.054(0.004)*** | -0.066(0.004)*** | -0.045(0.003)*** | -0.027(0.002)*** |
| year1995 | -0.029(0.003)*** | -0.047(0.004)*** | -0.044(0.004)*** | -0.028(0.003)*** | -0.013(0.002)*** |
| year1996 | -0.033(0.003)*** | -0.05(0.004)*** | -0.049(0.004)*** | -0.027(0.003)*** | -0.011(0.002)*** |
| year1997 | -0.028(0.003)*** | -0.047(0.004)*** | -0.045(0.004)*** | -0.029(0.003)*** | -0.015(0.002)*** |
| year1998 | -0.03(0.003)*** | -0.045(0.004)*** | -0.044(0.004)*** | -0.025(0.003)*** | -0.007(0.002)*** |
| year1999 | -0.038(0.003)*** | -0.058(0.004)*** | -0.059(0.004)*** | -0.034(0.003)*** | -0.015(0.002)*** |
| year2000 | -0.041(0.003)*** | -0.06(0.004)*** | -0.058(0.004)*** | -0.034(0.003)*** | -0.013(0.003)*** |
| year2001 | -0.032(0.003)*** | -0.053(0.004)*** | -0.051(0.004)*** | -0.031(0.004)*** | -0.011(0.002)*** |
| year2002 | -0.046(0.003)*** | -0.067(0.004)*** | -0.06(0.004)*** | -0.031(0.004)*** | -0.012(0.002)*** |
| year2003 | -0.044(0.003)*** | -0.067(0.004)*** | -0.069(0.004)*** | -0.046(0.004)*** | -0.018(0.003)*** |
| year2004 | -0.042(0.003)*** | -0.064(0.004)*** | -0.063(0.004)*** | -0.037(0.004)*** | -0.014(0.002)*** |
| year2005 | -0.045(0.003)*** | -0.076(0.004)*** | -0.076(0.004)*** | -0.048(0.004)*** | -0.018(0.003)*** |
| year2006 | -0.048(0.003)*** | -0.073(0.004)*** | -0.076(0.004)*** | -0.039(0.004)*** | -0.018(0.003)*** |
| year2007 | -0.043(0.003)*** | -0.058(0.004)*** | -0.059(0.004)*** | -0.03(0.004)*** | -0.01(0.003)*** |
| year2008 | -0.047(0.003)*** | -0.073(0.004)*** | -0.077(0.004)*** | -0.046(0.004)*** | -0.018(0.003)*** |
| year2009 | -0.05(0.003)*** | -0.077(0.004)*** | -0.077(0.004)*** | -0.042(0.004)*** | -0.014(0.003)*** |
| year2010 | -0.052(0.003)*** | -0.086(0.004)*** | -0.087(0.004)*** | -0.052(0.004)*** | -0.018(0.003)*** |
| year2011 | -0.047(0.003)*** | -0.077(0.004)*** | -0.073(0.004)*** | -0.043(0.004)*** | -0.015(0.003)*** |
| year2012 | -0.043(0.003)*** | -0.072(0.004)*** | -0.068(0.004)*** | -0.037(0.004)*** | -0.014(0.003)*** |
| year2013 | -0.048(0.003)*** | -0.079(0.004)*** | -0.081(0.004)*** | -0.051(0.004)*** | -0.019(0.003)*** |
| year2014 | -0.05(0.003)*** | -0.082(0.004)*** | -0.084(0.004)*** | -0.051(0.004)*** | -0.016(0.003)*** |
| year2015 | -0.059(0.003)*** | -0.095(0.004)*** | -0.104(0.004)*** | -0.069(0.004)*** | -0.028(0.003)*** |
| year2016 | -0.058(0.003)*** | -0.097(0.004)*** | -0.103(0.004)*** | -0.061(0.004)*** | -0.023(0.003)*** |
| year2017 | -0.058(0.003)*** | -0.096(0.004)*** | -0.107(0.004)*** | -0.063(0.003)*** | -0.026(0.003)*** |
| year2018 | -0.058(0.003)*** | -0.092(0.004)*** | -0.093(0.004)*** | -0.055(0.004)*** | -0.018(0.002)*** |
| year2019 | -0.057(0.003)*** | -0.094(0.004)*** | -0.103(0.004)*** | -0.059(0.003)*** | -0.02(0.003)*** |
| age2 | 0.007(0.003)* | 0(0.004)* | 0.002(0.004)* | 0(0.003)* | -0.002(0.002)* |
| age3 | 0.01(0.004)* | 0.006(0.005)* | -0.002(0.005)* | 0.001(0.004)* | -0.004(0.003)* |
| region1 | -0.013(0.001)*** | -0.02(0.001)*** | -0.033(0.002)*** | -0.026(0.001)*** | -0.015(0.001)*** |
| region2 | -0.022(0.001)*** | -0.038(0.002)*** | -0.054(0.002)*** | -0.042(0.002)*** | -0.025(0.001)*** |
| region3 | -0.011(0.001)*** | -0.02(0.002)*** | -0.035(0.002)*** | -0.029(0.001)*** | -0.018(0.001)*** |
| region4 | 0.012(0.002)*** | 0.013(0.002)*** | -0.005(0.003)*** | -0.012(0.002)*** | -0.013(0.001)*** |
| region5 | -0.035(0.001)*** | -0.069(0.002)*** | -0.1(0.002)*** | -0.075(0.002)*** | -0.041(0.001)*** |
| region6 | -0.028(0.001)*** | -0.053(0.002)*** | -0.082(0.002)*** | -0.07(0.002)*** | -0.042(0.001)*** |
| region7 | -0.025(0.001)*** | -0.047(0.001)*** | -0.071(0.002)*** | -0.059(0.001)*** | -0.035(0.001)*** |
| region8 | -0.03(0.001)*** | -0.056(0.002)*** | -0.083(0.002)*** | -0.069(0.001)*** | -0.039(0.001)*** |
| region9 | -0.009(0.002)*** | -0.028(0.002)*** | -0.057(0.002)*** | -0.053(0.002)*** | -0.031(0.002)*** |
| Covariate | tau=0.1 | tau=0.25 | tau=0.5 | tau=0.75 | tau=0.9 |
| region10 | -0.013(0.001)*** | -0.031(0.002)*** | -0.05(0.002)*** | -0.044(0.002)*** | -0.026(0.001)*** |
| region11 | 0.046(0.002)*** | 0.073(0.002)*** | 0.068(0.002)*** | 0.044(0.001)*** | 0.018(0.001)*** |
| year1990age2 | -0.003(0.004) | -0.001(0.005) | -0.007(0.005) | -0.009(0.005) | -0.005(0.003) |
| year1991age2 | -0.016(0.004)*** | -0.028(0.005)*** | -0.044(0.006)*** | -0.042(0.005)*** | -0.025(0.003)*** |
| year1992age2 | -0.026(0.004)*** | -0.049(0.005)*** | -0.07(0.005)*** | -0.058(0.005)*** | -0.039(0.003)*** |
| year1993age2 | -0.033(0.004)*** | -0.056(0.005)*** | -0.071(0.005)*** | -0.058(0.005)*** | -0.037(0.003)*** |
| year1994age2 | -0.028(0.004)*** | -0.044(0.005)*** | -0.057(0.005)*** | -0.047(0.005)*** | -0.025(0.003)*** |
| year1995age2 | -0.031(0.004)*** | -0.047(0.005)*** | -0.069(0.006)*** | -0.058(0.005)*** | -0.034(0.003)*** |
| year1996age2 | -0.028(0.004)*** | -0.047(0.005)*** | -0.062(0.006)*** | -0.054(0.005)*** | -0.032(0.004)*** |
| year1997age2 | -0.031(0.004)*** | -0.049(0.005)*** | -0.067(0.006)*** | -0.056(0.005)*** | -0.029(0.004)*** |
| year1998age2 | -0.029(0.004)*** | -0.045(0.005)*** | -0.055(0.006)*** | -0.045(0.005)*** | -0.029(0.004)*** |
| year1999age2 | -0.023(0.004)*** | -0.034(0.005)*** | -0.05(0.006)*** | -0.039(0.005)*** | -0.022(0.004)*** |
| year2000age2 | -0.014(0.004)*** | -0.021(0.005)*** | -0.036(0.006)*** | -0.022(0.005)*** | -0.013(0.004)*** |
| year2001age2 | -0.015(0.004)*** | -0.017(0.006)*** | -0.024(0.006)*** | -0.013(0.005)*** | -0.005(0.004)*** |
| year2002age2 | -0.007(0.004) | -0.01(0.006) | -0.02(0.006) | -0.012(0.005) | -0.008(0.003) |
| year2003age2 | -0.009(0.004)* | -0.012(0.006)* | -0.018(0.006)* | -0.004(0.005)* | -0.004(0.003)* |
| year2004age2 | -0.011(0.004)* | -0.01(0.006)* | -0.012(0.006)* | 0.001(0.005)* | 0(0.003)* |
| year2005age2 | -0.003(0.004) | 0.013(0.006) | 0.015(0.006) | 0.019(0.005) | 0.01(0.004) |
| year2006age2 | 0.006(0.004) | 0.02(0.006) | 0.025(0.006) | 0.023(0.005) | 0.018(0.003) |
| year2007age2 | 0.013(0.004)** | 0.021(0.006)** | 0.03(0.006)** | 0.03(0.005)** | 0.016(0.003)** |
| year2008age2 | 0.011(0.004)* | 0.034(0.006)* | 0.05(0.006)* | 0.046(0.005)* | 0.025(0.003)* |
| year2009age2 | 0.017(0.004)*** | 0.043(0.006)*** | 0.057(0.006)*** | 0.049(0.005)*** | 0.027(0.003)*** |
| year2010age2 | 0.013(0.004)** | 0.043(0.006)** | 0.058(0.006)** | 0.057(0.005)** | 0.027(0.003)** |
| year2011age2 | 0.015(0.004)*** | 0.046(0.006)*** | 0.066(0.006)*** | 0.059(0.005)*** | 0.032(0.003)*** |
| year2012age2 | 0.01(0.004)* | 0.041(0.006)* | 0.051(0.006)* | 0.052(0.005)* | 0.032(0.003)* |
| year2013age2 | 0.011(0.004)* | 0.036(0.006)* | 0.055(0.006)* | 0.057(0.005)* | 0.033(0.004)* |
| year2014age2 | 0.011(0.004)* | 0.039(0.006)* | 0.058(0.006)* | 0.055(0.005)* | 0.026(0.003)* |
| year2015age2 | 0.012(0.004)** | 0.034(0.006)** | 0.056(0.006)** | 0.057(0.005)** | 0.033(0.004)** |
| year2016age2 | 0.011(0.004)** | 0.042(0.006)** | 0.065(0.006)** | 0.061(0.005)** | 0.033(0.003)** |
| year2017age2 | 0.013(0.004)** | 0.045(0.006)** | 0.067(0.006)** | 0.062(0.005)** | 0.039(0.003)** |
| year2018age2 | 0.013(0.004)** | 0.043(0.006)** | 0.064(0.006)** | 0.06(0.005)** | 0.031(0.003)** |
| year2019age2 | 0.01(0.004)* | 0.042(0.006)* | 0.067(0.006)* | 0.062(0.005)* | 0.034(0.003)* |
| year1990age3 | -0.003(0.005) | -0.001(0.006) | -0.005(0.006) | -0.012(0.005) | -0.006(0.004) |
| year1991age3 | -0.017(0.005)*** | -0.016(0.006)*** | -0.026(0.006)*** | -0.023(0.005)*** | -0.01(0.004)*** |
| year1992age3 | -0.024(0.005)*** | -0.034(0.006)*** | -0.044(0.006)*** | -0.033(0.005)*** | -0.017(0.004)*** |
| year1993age3 | -0.037(0.005)*** | -0.061(0.006)*** | -0.075(0.006)*** | -0.065(0.005)*** | -0.035(0.004)*** |
| year1994age3 | -0.032(0.005)*** | -0.049(0.006)*** | -0.06(0.006)*** | -0.056(0.005)*** | -0.026(0.004)*** |
| year1995age3 | -0.034(0.005)*** | -0.057(0.006)*** | -0.08(0.006)*** | -0.063(0.005)*** | -0.036(0.004)*** |
| year1996age3 | -0.035(0.005)*** | -0.055(0.006)*** | -0.074(0.006)*** | -0.06(0.006)*** | -0.036(0.004)*** |
| year1997age3 | -0.039(0.005)*** | -0.059(0.006)*** | -0.078(0.006)*** | -0.067(0.005)*** | -0.036(0.004)*** |
| year1998age3 | -0.034(0.005)*** | -0.054(0.006)*** | -0.066(0.007)*** | -0.054(0.006)*** | -0.032(0.004)*** |
| year1999age3 | -0.024(0.005)*** | -0.04(0.006)*** | -0.051(0.007)*** | -0.042(0.006)*** | -0.023(0.004)*** |
| year2000age3 | -0.019(0.005)*** | -0.031(0.006)*** | -0.04(0.007)*** | -0.035(0.006)*** | -0.017(0.004)*** |
| year2001age3 | -0.02(0.005)*** | -0.026(0.006)*** | -0.033(0.007)*** | -0.025(0.006)*** | -0.01(0.004)*** |
| year2002age3 | -0.005(0.005) | -0.006(0.006) | -0.015(0.007) | -0.011(0.006) | 0(0.004) |
| Covariate | tau=0.1 | tau=0.25 | tau=0.5 | tau=0.75 | tau=0.9 |
| year2003age3 | -0.005(0.005) | -0.006(0.006) | -0.001(0.007) | -0.002(0.006) | 0.001(0.004) |
| year2004age3 | -0.004(0.005) | -0.001(0.006) | 0.006(0.007) | 0.006(0.006) | 0.003(0.004) |
| year2005age3 | -0.007(0.005) | 0.003(0.006) | 0.014(0.007) | 0.015(0.006) | 0.009(0.004) |
| year2006age3 | 0.004(0.005) | 0.019(0.006) | 0.024(0.007) | 0.014(0.006) | 0.016(0.004) |
| year2007age3 | 0.007(0.005) | 0.011(0.007) | 0.028(0.007) | 0.019(0.006) | 0.015(0.004) |
| year2008age3 | 0.012(0.005)* | 0.028(0.007)* | 0.047(0.007)* | 0.042(0.006)* | 0.027(0.004)* |
| year2009age3 | 0.016(0.005)** | 0.038(0.006)** | 0.062(0.007)** | 0.044(0.006)** | 0.028(0.004)** |
| year2010age3 | 0.017(0.005)*** | 0.041(0.006)*** | 0.07(0.007)*** | 0.055(0.006)*** | 0.034(0.004)*** |
| year2011age3 | 0.012(0.005)* | 0.044(0.007)* | 0.072(0.007)* | 0.06(0.006)* | 0.037(0.004)* |
| year2012age3 | 0.008(0.005) | 0.043(0.007) | 0.067(0.007) | 0.062(0.006) | 0.037(0.004) |
| year2013age3 | 0.011(0.005)* | 0.038(0.007)* | 0.068(0.007)* | 0.064(0.006)* | 0.038(0.004)* |
| year2014age3 | 0.009(0.005) | 0.036(0.006) | 0.066(0.007) | 0.067(0.006) | 0.037(0.004) |
| year2015age3 | 0.014(0.005)** | 0.034(0.006)** | 0.071(0.007)** | 0.072(0.006)** | 0.043(0.004)** |
| year2016age3 | 0.008(0.005) | 0.035(0.006) | 0.065(0.007) | 0.061(0.006) | 0.039(0.004) |
| year2017age3 | 0.01(0.005)* | 0.032(0.006)* | 0.069(0.007)* | 0.065(0.006)* | 0.042(0.004)* |
| year2018age3 | 0.009(0.005) | 0.035(0.007) | 0.065(0.007) | 0.056(0.006) | 0.032(0.004) |
| year2019age3 | 0.009(0.005) | 0.039(0.007) | 0.074(0.007) | 0.063(0.006) | 0.035(0.004) |

Supplementary Table 3.10: Coefficient estimates (standard errors) for different quantiles (tau) for BOC, girls; **p<*0.05, ***p<*0.01,****p<*0.001, age2=10-12 years, age3=13-15 years.

| Covariate | tau=0.1 | tau=0.25 | tau=0.5 | tau=0.75 | tau=0.9 |
| --- | --- | --- | --- | --- | --- |
| (Intercept) | 0.214(0.003)*** | 0.436(0.003)*** | 0.704(0.003)*** | 0.894(0.002)*** | 0.971(0.001)*** |
| year1990 | -0.024(0.004)*** | -0.043(0.004)*** | -0.044(0.004)*** | -0.027(0.003)*** | -0.013(0.002)*** |
| year1991 | -0.031(0.004)*** | -0.041(0.004)*** | -0.047(0.004)*** | -0.032(0.003)*** | -0.016(0.002)*** |
| year1992 | -0.049(0.004)*** | -0.075(0.004)*** | -0.077(0.004)*** | -0.051(0.003)*** | -0.026(0.002)*** |
| year1993 | -0.038(0.004)*** | -0.057(0.004)*** | -0.058(0.004)*** | -0.04(0.003)*** | -0.018(0.002)*** |
| year1994 | -0.057(0.003)*** | -0.091(0.004)*** | -0.094(0.004)*** | -0.065(0.003)*** | -0.031(0.002)*** |
| year1995 | -0.054(0.003)*** | -0.081(0.004)*** | -0.08(0.004)*** | -0.052(0.003)*** | -0.024(0.002)*** |
| year1996 | -0.054(0.003)*** | -0.078(0.004)*** | -0.08(0.004)*** | -0.053(0.003)*** | -0.021(0.002)*** |
| year1997 | -0.056(0.004)*** | -0.093(0.004)*** | -0.093(0.004)*** | -0.063(0.003)*** | -0.028(0.002)*** |
| year1998 | -0.057(0.003)*** | -0.08(0.004)*** | -0.083(0.004)*** | -0.053(0.003)*** | -0.024(0.002)*** |
| year1999 | -0.062(0.003)*** | -0.093(0.004)*** | -0.099(0.004)*** | -0.064(0.003)*** | -0.028(0.002)*** |
| year2000 | -0.067(0.003)*** | -0.104(0.004)*** | -0.101(0.004)*** | -0.065(0.003)*** | -0.03(0.002)*** |
| year2001 | -0.067(0.004)*** | -0.108(0.004)*** | -0.11(0.004)*** | -0.072(0.003)*** | -0.031(0.002)*** |
| year2002 | -0.075(0.003)*** | -0.117(0.004)*** | -0.121(0.004)*** | -0.079(0.003)*** | -0.034(0.002)*** |
| year2003 | -0.079(0.003)*** | -0.122(0.004)*** | -0.128(0.004)*** | -0.086(0.003)*** | -0.037(0.002)*** |
| year2004 | -0.08(0.003)*** | -0.126(0.004)*** | -0.128(0.004)*** | -0.086(0.004)*** | -0.035(0.002)*** |
| year2005 | -0.092(0.003)*** | -0.147(0.004)*** | -0.156(0.004)*** | -0.109(0.003)*** | -0.05(0.002)*** |
| year2006 | -0.094(0.003)*** | -0.148(0.004)*** | -0.156(0.004)*** | -0.106(0.004)*** | -0.048(0.003)*** |
| year2007 | -0.086(0.003)*** | -0.141(0.004)*** | -0.147(0.004)*** | -0.096(0.004)*** | -0.042(0.002)*** |
| year2008 | -0.095(0.003)*** | -0.153(0.004)*** | -0.162(0.004)*** | -0.112(0.004)*** | -0.05(0.003)*** |
| year2009 | -0.096(0.003)*** | -0.158(0.004)*** | -0.168(0.004)*** | -0.111(0.004)*** | -0.049(0.003)*** |
| year2010 | -0.101(0.003)*** | -0.164(0.004)*** | -0.181(0.004)*** | -0.125(0.004)*** | -0.058(0.003)*** |
| year2011 | -0.099(0.003)*** | -0.161(0.004)*** | -0.174(0.004)*** | -0.121(0.004)*** | -0.054(0.003)*** |
| year2012 | -0.099(0.003)*** | -0.165(0.004)*** | -0.178(0.004)*** | -0.123(0.004)*** | -0.056(0.003)*** |
| year2013 | -0.104(0.003)*** | -0.172(0.004)*** | -0.191(0.004)*** | -0.138(0.004)*** | -0.064(0.003)*** |
| year2014 | -0.106(0.003)*** | -0.181(0.004)*** | -0.201(0.004)*** | -0.144(0.004)*** | -0.071(0.003)*** |
| year2015 | -0.113(0.003)*** | -0.19(0.004)*** | -0.217(0.004)*** | -0.155(0.004)*** | -0.078(0.003)*** |
| year2016 | -0.114(0.003)*** | -0.187(0.004)*** | -0.214(0.004)*** | -0.161(0.004)*** | -0.077(0.003)*** |
| year2017 | -0.117(0.003)*** | -0.195(0.004)*** | -0.233(0.004)*** | -0.174(0.004)*** | -0.088(0.003)*** |
| year2018 | -0.116(0.003)*** | -0.195(0.004)*** | -0.228(0.004)*** | -0.169(0.004)*** | -0.085(0.003)*** |
| year2019 | -0.117(0.003)*** | -0.199(0.004)*** | -0.232(0.004)*** | -0.174(0.004)*** | -0.087(0.003)*** |
| age2 | 0.007(0.004) | 0.019(0.005) | 0.03(0.004) | 0.017(0.003) | 0.007(0.002) |
| age3 | 0.002(0.004) | -0.001(0.005) | 0.009(0.005) | 0.003(0.003) | -0.005(0.002) |
| region1 | -0.021(0.001)*** | -0.039(0.002)*** | -0.048(0.002)*** | -0.04(0.001)*** | -0.021(0.001)*** |
| region2 | -0.029(0.001)*** | -0.051(0.002)*** | -0.066(0.002)*** | -0.057(0.002)*** | -0.035(0.001)*** |
| region3 | -0.009(0.001)*** | -0.017(0.002)*** | -0.032(0.002)*** | -0.033(0.001)*** | -0.021(0.001)*** |
| region4 | 0.008(0.002)*** | -0.003(0.003)*** | -0.024(0.003)*** | -0.034(0.002)*** | -0.024(0.001)*** |
| region5 | -0.061(0.001)*** | -0.11(0.002)*** | -0.136(0.002)*** | -0.101(0.002)*** | -0.054(0.001)*** |
| region6 | -0.026(0.001)*** | -0.054(0.002)*** | -0.078(0.002)*** | -0.067(0.002)*** | -0.038(0.001)*** |
| region7 | -0.047(0.001)*** | -0.086(0.001)*** | -0.112(0.002)*** | -0.092(0.001)*** | -0.051(0.001)*** |
| region8 | -0.044(0.001)*** | -0.085(0.002)*** | -0.114(0.002)*** | -0.095(0.001)*** | -0.053(0.001)*** |
| region9 | -0.019(0.002)*** | -0.046(0.002)*** | -0.075(0.002)*** | -0.073(0.002)*** | -0.045(0.002)*** |
| region10 | -0.029(0.001)*** | -0.057(0.002)*** | -0.084(0.002)*** | -0.075(0.002)*** | -0.042(0.001)*** |
| Covariate | tau=0.1 | tau=0.25 | tau=0.5 | tau=0.75 | tau=0.9 |
| region11 | 0.004(0.002)* | 0.011(0.002)* | 0.013(0.002)* | 0.005(0.002)* | 0.002(0.001)* |
| year1990age2 | -0.002(0.006) | -0.001(0.006) | -0.009(0.006) | -0.007(0.004) | -0.005(0.002) |
| year1991age2 | -0.019(0.005)*** | -0.042(0.006)*** | -0.049(0.005)*** | -0.035(0.004)*** | -0.02(0.003)*** |
| year1992age2 | -0.033(0.005)*** | -0.065(0.006)*** | -0.075(0.005)*** | -0.056(0.004)*** | -0.032(0.003)*** |
| year1993age2 | -0.042(0.005)*** | -0.076(0.006)*** | -0.091(0.006)*** | -0.064(0.004)*** | -0.033(0.003)*** |
| year1994age2 | -0.038(0.005)*** | -0.073(0.006)*** | -0.096(0.005)*** | -0.068(0.004)*** | -0.038(0.003)*** |
| year1995age2 | -0.04(0.005)*** | -0.075(0.006)*** | -0.093(0.006)*** | -0.07(0.004)*** | -0.036(0.003)*** |
| year1996age2 | -0.04(0.005)*** | -0.076(0.006)*** | -0.092(0.005)*** | -0.062(0.004)*** | -0.036(0.003)*** |
| year1997age2 | -0.035(0.005)*** | -0.065(0.006)*** | -0.089(0.006)*** | -0.062(0.004)*** | -0.035(0.003)*** |
| year1998age2 | -0.031(0.005)*** | -0.067(0.006)*** | -0.082(0.006)*** | -0.063(0.004)*** | -0.033(0.003)*** |
| year1999age2 | -0.028(0.005)*** | -0.058(0.006)*** | -0.071(0.006)*** | -0.051(0.005)*** | -0.028(0.003)*** |
| year2000age2 | -0.015(0.005)** | -0.037(0.006)** | -0.06(0.006)** | -0.042(0.004)** | -0.025(0.003)** |
| year2001age2 | -0.017(0.005)** | -0.038(0.006)** | -0.055(0.006)** | -0.034(0.005)** | -0.019(0.003)** |
| year2002age2 | -0.019(0.005)*** | -0.044(0.006)*** | -0.053(0.006)*** | -0.037(0.005)*** | -0.024(0.003)*** |
| year2003age2 | -0.014(0.005)** | -0.037(0.006)** | -0.052(0.006)** | -0.037(0.005)** | -0.022(0.003)** |
| year2004age2 | -0.013(0.005)* | -0.031(0.006)* | -0.051(0.006)* | -0.034(0.005)* | -0.023(0.003)* |
| year2005age2 | 0.001(0.005) | -0.007(0.006) | -0.019(0.006) | 0.003(0.005) | -0.001(0.003) |
| year2006age2 | 0.006(0.005) | 0.001(0.006) | -0.002(0.006) | 0.006(0.005) | 0.005(0.003) |
| year2007age2 | 0.002(0.005) | 0(0.006) | -0.001(0.006) | 0.007(0.005) | 0.005(0.003) |
| year2008age2 | 0.007(0.005) | 0.014(0.006) | 0.009(0.006) | 0.02(0.005) | 0.01(0.003) |
| year2009age2 | 0.01(0.005) | 0.023(0.006) | 0.031(0.006) | 0.027(0.005) | 0.015(0.003) |
| year2010age2 | 0.014(0.005)** | 0.021(0.006)** | 0.033(0.006)** | 0.033(0.005)** | 0.019(0.003)** |
| year2011age2 | 0.006(0.005) | 0.021(0.006) | 0.029(0.006) | 0.035(0.005) | 0.018(0.003) |
| year2012age2 | 0.007(0.005) | 0.018(0.006) | 0.032(0.006) | 0.037(0.005) | 0.021(0.003) |
| year2013age2 | 0.001(0.005) | 0.005(0.006) | 0.018(0.006) | 0.034(0.005) | 0.018(0.004) |
| year2014age2 | -0.001(0.005) | 0.01(0.006) | 0.018(0.006) | 0.034(0.005) | 0.027(0.004) |
| year2015age2 | -0.001(0.005) | 0(0.006) | 0.003(0.006) | 0.021(0.005) | 0.021(0.004) |
| year2016age2 | 0(0.005) | -0.004(0.006) | 0.002(0.006) | 0.03(0.005) | 0.023(0.004) |
| year2017age2 | -0.003(0.005) | -0.008(0.006) | 0.005(0.006) | 0.03(0.005) | 0.027(0.004) |
| year2018age2 | -0.004(0.005) | -0.005(0.006) | 0.006(0.006) | 0.027(0.005) | 0.024(0.004) |
| year2019age2 | -0.004(0.005) | -0.007(0.006) | 0.003(0.006) | 0.026(0.005) | 0.023(0.004) |
| year1990age3 | -0.006(0.006) | -0.013(0.007) | -0.008(0.007) | -0.004(0.005) | 0(0.003) |
| year1991age3 | -0.007(0.006) | -0.021(0.007) | -0.024(0.007) | -0.016(0.005) | -0.003(0.003) |
| year1992age3 | -0.026(0.005)*** | -0.049(0.007)*** | -0.05(0.007)*** | -0.032(0.005)*** | -0.011(0.003)*** |
| year1993age3 | -0.035(0.005)*** | -0.068(0.007)*** | -0.08(0.006)*** | -0.055(0.005)*** | -0.03(0.003)*** |
| year1994age3 | -0.034(0.005)*** | -0.064(0.006)*** | -0.088(0.006)*** | -0.06(0.005)*** | -0.035(0.003)*** |
| year1995age3 | -0.042(0.005)*** | -0.08(0.006)*** | -0.108(0.007)*** | -0.083(0.005)*** | -0.043(0.003)*** |
| year1996age3 | -0.042(0.005)*** | -0.087(0.006)*** | -0.114(0.006)*** | -0.083(0.005)*** | -0.044(0.003)*** |
| year1997age3 | -0.039(0.005)*** | -0.065(0.007)*** | -0.094(0.007)*** | -0.069(0.005)*** | -0.034(0.003)*** |
| year1998age3 | -0.038(0.005)*** | -0.071(0.007)*** | -0.086(0.007)*** | -0.062(0.005)*** | -0.034(0.004)*** |
| year1999age3 | -0.029(0.005)*** | -0.053(0.007)*** | -0.066(0.007)*** | -0.05(0.005)*** | -0.024(0.004)*** |
| year2000age3 | -0.017(0.005)** | -0.04(0.007)** | -0.064(0.007)** | -0.05(0.005)** | -0.024(0.004)** |
| year2001age3 | -0.021(0.005)*** | -0.04(0.007)*** | -0.056(0.007)*** | -0.042(0.005)*** | -0.022(0.004)*** |
| year2002age3 | -0.009(0.005) | -0.018(0.007) | -0.036(0.007) | -0.023(0.005) | -0.014(0.004) |
| year2003age3 | -0.008(0.005) | -0.018(0.007) | -0.025(0.007) | -0.015(0.006) | -0.009(0.004) |
| Covariate | tau=0.1 | tau=0.25 | tau=0.5 | tau=0.75 | tau=0.9 |
| year2004age3 | -0.001(0.005) | -0.012(0.007) | -0.021(0.007) | -0.01(0.006) | -0.006(0.004) |
| year2005age3 | 0.007(0.005) | 0.013(0.007) | 0.01(0.007) | 0.017(0.006) | 0.013(0.004) |
| year2006age3 | 0.006(0.005) | 0.009(0.007) | 0.008(0.007) | 0.021(0.006) | 0.013(0.004) |
| year2007age3 | 0.011(0.006) | 0.023(0.007) | 0.028(0.007) | 0.025(0.006) | 0.013(0.004) |
| year2008age3 | 0.021(0.006)*** | 0.048(0.007)*** | 0.056(0.007)*** | 0.046(0.006)*** | 0.027(0.004)*** |
| year2009age3 | 0.032(0.006)*** | 0.056(0.007)*** | 0.07(0.007)*** | 0.058(0.006)*** | 0.036(0.004)*** |
| year2010age3 | 0.033(0.006)*** | 0.06(0.007)*** | 0.081(0.007)*** | 0.076(0.006)*** | 0.044(0.004)*** |
| year2011age3 | 0.022(0.006)*** | 0.066(0.007)*** | 0.086(0.007)*** | 0.081(0.006)*** | 0.049(0.004)*** |
| year2012age3 | 0.025(0.006)*** | 0.063(0.007)*** | 0.083(0.007)*** | 0.078(0.006)*** | 0.047(0.004)*** |
| year2013age3 | 0.026(0.006)*** | 0.059(0.007)*** | 0.083(0.007)*** | 0.08(0.006)*** | 0.052(0.004)*** |
| year2014age3 | 0.02(0.005)*** | 0.051(0.007)*** | 0.082(0.007)*** | 0.084(0.006)*** | 0.061(0.004)*** |
| year2015age3 | 0.013(0.005)* | 0.035(0.007)* | 0.059(0.007)* | 0.069(0.006)* | 0.053(0.004)* |
| year2016age3 | 0.006(0.005) | 0.02(0.007) | 0.05(0.008) | 0.068(0.006) | 0.047(0.004) |
| year2017age3 | 0.003(0.005) | 0.019(0.007) | 0.051(0.007) | 0.074(0.006) | 0.053(0.004) |
| year2018age3 | 0.001(0.005) | 0.012(0.007) | 0.029(0.007) | 0.054(0.006) | 0.044(0.004) |
| year2019age3 | -0.002(0.005) | 0.013(0.007) | 0.026(0.007) | 0.049(0.006) | 0.042(0.004) |

Supplementary Table 3.11: Coefficient estimates (standard errors) for different quantiles (tau) for SU60, boys; **p<*0.05, ***p<*0.01,****p<*0.001, age2=10-12 years, age3=13-15 years.

| Covariate | tau=0.1 | tau=0.25 | tau=0.5 | tau=0.75 | tau=0.9 |
| --- | --- | --- | --- | --- | --- |
| (Intercept) | 0.053(0.002)*** | 0.159(0.002)*** | 0.365(0.003)*** | 0.62(0.003)*** | 0.816(0.003)*** |
| year1990 | -0.004(0.002)* | -0.002(0.003)* | -0.003(0.004)* | 0.002(0.004)* | 0(0.004)* |
| year1991 | -0.004(0.002)* | -0.002(0.003)* | -0.002(0.004)* | -0.004(0.004)* | -0.001(0.004)* |
| year1992 | -0.003(0.002) | 0.003(0.003) | 0.006(0.004) | 0.004(0.004) | 0.003(0.004) |
| year1993 | -0.001(0.002) | 0.002(0.003) | 0.001(0.004) | 0(0.004) | 0.001(0.004) |
| year1994 | 0.004(0.002) | 0.014(0.003) | 0.018(0.004) | 0.015(0.004) | 0.011(0.004) |
| year1995 | 0.008(0.002)*** | 0.02(0.003)*** | 0.026(0.004)*** | 0.028(0.004)*** | 0.018(0.004)*** |
| year1996 | 0.01(0.002)*** | 0.023(0.003)*** | 0.028(0.004)*** | 0.029(0.004)*** | 0.02(0.004)*** |
| year1997 | 0.021(0.002)*** | 0.041(0.003)*** | 0.053(0.004)*** | 0.042(0.004)*** | 0.026(0.003)*** |
| year1998 | 0.02(0.002)*** | 0.038(0.003)*** | 0.053(0.004)*** | 0.051(0.004)*** | 0.036(0.003)*** |
| year1999 | 0.022(0.002)*** | 0.043(0.003)*** | 0.061(0.004)*** | 0.059(0.004)*** | 0.039(0.003)*** |
| year2000 | 0.022(0.002)*** | 0.05(0.003)*** | 0.075(0.004)*** | 0.072(0.004)*** | 0.047(0.004)*** |
| year2001 | 0.021(0.002)*** | 0.044(0.003)*** | 0.076(0.004)*** | 0.074(0.004)*** | 0.051(0.003)*** |
| year2002 | 0.025(0.002)*** | 0.056(0.003)*** | 0.092(0.004)*** | 0.091(0.004)*** | 0.062(0.003)*** |
| year2003 | 0.028(0.002)*** | 0.062(0.003)*** | 0.093(0.004)*** | 0.089(0.004)*** | 0.059(0.003)*** |
| year2004 | 0.034(0.002)*** | 0.074(0.003)*** | 0.108(0.004)*** | 0.102(0.004)*** | 0.065(0.003)*** |
| year2005 | 0.04(0.003)*** | 0.084(0.004)*** | 0.124(0.004)*** | 0.116(0.004)*** | 0.074(0.003)*** |
| year2006 | 0.041(0.003)*** | 0.09(0.004)*** | 0.13(0.004)*** | 0.123(0.004)*** | 0.081(0.003)*** |
| year2007 | 0.038(0.003)*** | 0.08(0.004)*** | 0.124(0.004)*** | 0.119(0.004)*** | 0.075(0.003)*** |
| year2008 | 0.033(0.002)*** | 0.072(0.004)*** | 0.115(0.004)*** | 0.116(0.004)*** | 0.08(0.003)*** |
| year2009 | 0.035(0.003)*** | 0.081(0.004)*** | 0.135(0.004)*** | 0.128(0.004)*** | 0.084(0.003)*** |
| year2010 | 0.047(0.003)*** | 0.101(0.004)*** | 0.157(0.004)*** | 0.153(0.004)*** | 0.098(0.003)*** |
| year2011 | 0.043(0.003)*** | 0.105(0.004)*** | 0.165(0.004)*** | 0.162(0.004)*** | 0.101(0.003)*** |
| year2012 | 0.054(0.003)*** | 0.12(0.004)*** | 0.183(0.004)*** | 0.167(0.004)*** | 0.102(0.003)*** |
| year2013 | 0.054(0.003)*** | 0.117(0.004)*** | 0.182(0.004)*** | 0.165(0.004)*** | 0.104(0.003)*** |
| year2014 | 0.058(0.003)*** | 0.124(0.004)*** | 0.191(0.004)*** | 0.174(0.004)*** | 0.107(0.003)*** |
| year2015 | 0.059(0.003)*** | 0.127(0.004)*** | 0.189(0.004)*** | 0.174(0.004)*** | 0.107(0.003)*** |
| year2016 | 0.056(0.003)*** | 0.13(0.004)*** | 0.195(0.004)*** | 0.175(0.004)*** | 0.108(0.003)*** |
| year2017 | 0.06(0.003)*** | 0.135(0.004)*** | 0.197(0.004)*** | 0.176(0.004)*** | 0.108(0.003)*** |
| year2018 | 0.062(0.003)*** | 0.131(0.004)*** | 0.192(0.004)*** | 0.174(0.004)*** | 0.105(0.003)*** |
| year2019 | 0.063(0.003)*** | 0.138(0.004)*** | 0.197(0.004)*** | 0.175(0.004)*** | 0.106(0.003)*** |
| age2 | -0.007(0.002)*** | -0.025(0.003)*** | -0.051(0.004)*** | -0.066(0.004)*** | -0.047(0.004)*** |
| age3 | -0.017(0.002)*** | -0.042(0.003)*** | -0.097(0.004)*** | -0.117(0.005)*** | -0.095(0.005)*** |
| region1 | 0.02(0.001)*** | 0.023(0.001)*** | 0.022(0.002)*** | 0.017(0.001)*** | 0.009(0.001)*** |
| region2 | 0.029(0.001)*** | 0.043(0.002)*** | 0.05(0.002)*** | 0.042(0.002)*** | 0.02(0.001)*** |
| region3 | 0.018(0.001)*** | 0.028(0.001)*** | 0.036(0.002)*** | 0.03(0.001)*** | 0.015(0.001)*** |
| region4 | 0.004(0.001)** | 0.007(0.002)** | 0.014(0.003)** | 0.01(0.002)** | 0.002(0.002)** |
| region5 | 0.036(0.001)*** | 0.056(0.002)*** | 0.065(0.002)*** | 0.05(0.002)*** | 0.024(0.001)*** |
| region6 | 0.017(0.001)*** | 0.024(0.001)*** | 0.031(0.002)*** | 0.025(0.002)*** | 0.014(0.001)*** |
| region7 | 0.037(0.001)*** | 0.054(0.001)*** | 0.062(0.001)*** | 0.049(0.001)*** | 0.026(0.001)*** |
| region8 | 0.037(0.001)*** | 0.052(0.001)*** | 0.057(0.002)*** | 0.043(0.001)*** | 0.021(0.001)*** |
| region9 | 0.028(0.002)*** | 0.04(0.002)*** | 0.048(0.002)*** | 0.033(0.002)*** | 0.016(0.001)*** |
| Covariate | tau=0.1 | tau=0.25 | tau=0.5 | tau=0.75 | tau=0.9 |
| region10 | 0.011(0.001)*** | 0.001(0.001)*** | -0.013(0.002)*** | -0.02(0.002)*** | -0.015(0.001)*** |
| region11 | 0.011(0.001)*** | 0.011(0.002)*** | 0.01(0.002)*** | 0.004(0.002)*** | -0.001(0.001)*** |
| year1990age2 | 0.011(0.003)*** | 0.015(0.004)*** | 0.023(0.005)*** | 0.016(0.006)*** | 0.011(0.005)*** |
| year1991age2 | 0.014(0.003)*** | 0.026(0.004)*** | 0.039(0.005)*** | 0.048(0.006)*** | 0.036(0.005)*** |
| year1992age2 | 0.02(0.003)*** | 0.034(0.004)*** | 0.052(0.005)*** | 0.061(0.006)*** | 0.043(0.005)*** |
| year1993age2 | 0.018(0.003)*** | 0.034(0.004)*** | 0.061(0.005)*** | 0.068(0.006)*** | 0.054(0.005)*** |
| year1994age2 | 0.017(0.003)*** | 0.032(0.004)*** | 0.056(0.005)*** | 0.064(0.006)*** | 0.046(0.005)*** |
| year1995age2 | 0.015(0.003)*** | 0.029(0.004)*** | 0.053(0.005)*** | 0.061(0.006)*** | 0.05(0.005)*** |
| year1996age2 | 0.02(0.003)*** | 0.038(0.004)*** | 0.07(0.005)*** | 0.08(0.006)*** | 0.061(0.005)*** |
| year1997age2 | 0.015(0.003)*** | 0.032(0.004)*** | 0.062(0.005)*** | 0.082(0.006)*** | 0.064(0.005)*** |
| year1998age2 | 0.015(0.003)*** | 0.034(0.004)*** | 0.07(0.005)*** | 0.083(0.006)*** | 0.06(0.005)*** |
| year1999age2 | 0.017(0.003)*** | 0.044(0.004)*** | 0.07(0.005)*** | 0.085(0.006)*** | 0.061(0.005)*** |
| year2000age2 | 0.017(0.003)*** | 0.04(0.004)*** | 0.064(0.006)*** | 0.082(0.006)*** | 0.063(0.005)*** |
| year2001age2 | 0.02(0.003)*** | 0.045(0.005)*** | 0.069(0.006)*** | 0.086(0.006)*** | 0.059(0.005)*** |
| year2002age2 | 0.015(0.003)*** | 0.04(0.005)*** | 0.059(0.006)*** | 0.076(0.006)*** | 0.058(0.005)*** |
| year2003age2 | 0.018(0.003)*** | 0.042(0.005)*** | 0.067(0.006)*** | 0.084(0.006)*** | 0.062(0.005)*** |
| year2004age2 | 0.019(0.004)*** | 0.043(0.005)*** | 0.074(0.006)*** | 0.093(0.006)*** | 0.069(0.005)*** |
| year2005age2 | 0.016(0.004)*** | 0.038(0.005)*** | 0.067(0.006)*** | 0.085(0.006)*** | 0.058(0.005)*** |
| year2006age2 | 0.011(0.004)** | 0.023(0.005)** | 0.056(0.006)** | 0.079(0.006)** | 0.055(0.005)** |
| year2007age2 | 0.013(0.004)*** | 0.031(0.005)*** | 0.059(0.006)*** | 0.078(0.006)*** | 0.059(0.005)*** |
| year2008age2 | 0.012(0.003)*** | 0.037(0.005)*** | 0.07(0.006)*** | 0.082(0.006)*** | 0.056(0.005)*** |
| year2009age2 | 0.006(0.004) | 0.023(0.005) | 0.051(0.006) | 0.082(0.006) | 0.055(0.005) |
| year2010age2 | 0.001(0.004) | 0.016(0.005) | 0.038(0.006) | 0.06(0.006) | 0.05(0.005) |
| year2011age2 | 0.006(0.004) | 0.019(0.005) | 0.038(0.006) | 0.059(0.006) | 0.05(0.005) |
| year2012age2 | 0.002(0.004) | 0.012(0.005) | 0.042(0.006) | 0.076(0.006) | 0.057(0.005) |
| year2013age2 | 0.011(0.004)** | 0.032(0.005)** | 0.058(0.006)** | 0.085(0.006)** | 0.058(0.004)** |
| year2014age2 | 0.012(0.004)** | 0.035(0.005)** | 0.059(0.006)** | 0.082(0.006)** | 0.059(0.004)** |
| year2015age2 | 0.007(0.004) | 0.028(0.005) | 0.055(0.006) | 0.073(0.006) | 0.053(0.004) |
| year2016age2 | 0.01(0.004)** | 0.019(0.005)** | 0.047(0.006)** | 0.073(0.005)** | 0.053(0.004)** |
| year2017age2 | 0.005(0.004) | 0.015(0.005) | 0.04(0.006) | 0.071(0.005) | 0.05(0.004) |
| year2018age2 | 0(0.004) | 0.017(0.005) | 0.047(0.006) | 0.073(0.005) | 0.054(0.004) |
| year2019age2 | -0.006(0.004) | 0.003(0.005) | 0.033(0.006) | 0.067(0.005) | 0.052(0.004) |
| year1990age3 | 0.014(0.003)*** | 0.017(0.004)*** | 0.025(0.005)*** | 0.021(0.006)*** | 0.02(0.007)*** |
| year1991age3 | 0.019(0.003)*** | 0.034(0.004)*** | 0.055(0.006)*** | 0.061(0.007)*** | 0.052(0.007)*** |
| year1992age3 | 0.021(0.003)*** | 0.035(0.004)*** | 0.064(0.006)*** | 0.083(0.007)*** | 0.071(0.007)*** |
| year1993age3 | 0.025(0.003)*** | 0.053(0.004)*** | 0.102(0.006)*** | 0.114(0.006)*** | 0.094(0.007)*** |
| year1994age3 | 0.031(0.003)*** | 0.054(0.004)*** | 0.098(0.006)*** | 0.117(0.006)*** | 0.1(0.006)*** |
| year1995age3 | 0.03(0.003)*** | 0.055(0.004)*** | 0.108(0.006)*** | 0.124(0.006)*** | 0.104(0.006)*** |
| year1996age3 | 0.033(0.003)*** | 0.06(0.004)*** | 0.125(0.006)*** | 0.142(0.006)*** | 0.115(0.006)*** |
| year1997age3 | 0.031(0.003)*** | 0.055(0.004)*** | 0.113(0.006)*** | 0.139(0.006)*** | 0.116(0.006)*** |
| year1998age3 | 0.03(0.003)*** | 0.065(0.005)*** | 0.132(0.006)*** | 0.149(0.006)*** | 0.119(0.006)*** |
| year1999age3 | 0.036(0.004)*** | 0.078(0.005)*** | 0.137(0.006)*** | 0.156(0.006)*** | 0.128(0.006)*** |
| year2000age3 | 0.033(0.004)*** | 0.068(0.005)*** | 0.141(0.006)*** | 0.162(0.006)*** | 0.13(0.006)*** |
| year2001age3 | 0.033(0.004)*** | 0.073(0.005)*** | 0.135(0.006)*** | 0.151(0.006)*** | 0.115(0.006)*** |
| year2002age3 | 0.028(0.004)*** | 0.064(0.005)*** | 0.124(0.006)*** | 0.141(0.007)*** | 0.112(0.006)*** |
| Covariate | tau=0.1 | tau=0.25 | tau=0.5 | tau=0.75 | tau=0.9 |
| year2003age3 | 0.031(0.004)*** | 0.062(0.005)*** | 0.127(0.006)*** | 0.148(0.006)*** | 0.118(0.006)*** |
| year2004age3 | 0.028(0.004)*** | 0.064(0.005)*** | 0.125(0.006)*** | 0.147(0.007)*** | 0.121(0.006)*** |
| year2005age3 | 0.023(0.004)*** | 0.056(0.005)*** | 0.116(0.006)*** | 0.14(0.006)*** | 0.113(0.006)*** |
| year2006age3 | 0.022(0.004)*** | 0.05(0.006)*** | 0.112(0.006)*** | 0.139(0.007)*** | 0.106(0.006)*** |
| year2007age3 | 0.023(0.004)*** | 0.059(0.005)*** | 0.117(0.007)*** | 0.141(0.007)*** | 0.112(0.006)*** |
| year2008age3 | 0.027(0.004)*** | 0.068(0.006)*** | 0.121(0.007)*** | 0.135(0.007)*** | 0.103(0.006)*** |
| year2009age3 | 0.025(0.004)*** | 0.061(0.006)*** | 0.122(0.006)*** | 0.14(0.006)*** | 0.107(0.006)*** |
| year2010age3 | 0.018(0.004)*** | 0.045(0.006)*** | 0.102(0.007)*** | 0.121(0.007)*** | 0.103(0.006)*** |
| year2011age3 | 0.022(0.004)*** | 0.039(0.006)*** | 0.099(0.007)*** | 0.115(0.006)*** | 0.102(0.006)*** |
| year2012age3 | 0.02(0.005)*** | 0.037(0.006)*** | 0.095(0.006)*** | 0.132(0.007)*** | 0.108(0.006)*** |
| year2013age3 | 0.015(0.005)** | 0.038(0.006)** | 0.111(0.007)** | 0.142(0.006)** | 0.107(0.006)** |
| year2014age3 | 0.017(0.005)*** | 0.044(0.006)*** | 0.1(0.007)*** | 0.136(0.006)*** | 0.107(0.006)*** |
| year2015age3 | 0.02(0.005)*** | 0.042(0.006)*** | 0.104(0.007)*** | 0.128(0.006)*** | 0.103(0.006)*** |
| year2016age3 | 0.022(0.004)*** | 0.035(0.006)*** | 0.098(0.007)*** | 0.119(0.006)*** | 0.092(0.006)*** |
| year2017age3 | 0.013(0.004)** | 0.02(0.006)** | 0.086(0.006)** | 0.12(0.006)** | 0.1(0.006)** |
| year2018age3 | 0.013(0.005)** | 0.031(0.006)** | 0.084(0.006)** | 0.11(0.006)** | 0.095(0.006)** |
| year2019age3 | -0.002(0.004) | 0.007(0.006) | 0.062(0.007) | 0.104(0.006) | 0.09(0.006) |

Supplementary Table 3.12: Coefficient estimates (standard errors) for different quantiles (tau) for SU60, girls; **p<*0.05, ***p<*0.01,****p<*0.001, age2=10-12 years, age3=13-15 years.

| Covariate | tau=0.1 | tau=0.25 | tau=0.5 | tau=0.75 | tau=0.9 |
| --- | --- | --- | --- | --- | --- |
| (Intercept) | 0.038(0.002)*** | 0.13(0.002)*** | 0.318(0.003)*** | 0.578(0.003)*** | 0.796(0.003)*** |
| year1990 | 0(0.002) | 0.005(0.003) | 0.016(0.004) | 0.018(0.004) | 0.005(0.003) |
| year1991 | 0(0.002) | 0.004(0.003) | 0.013(0.004) | 0.013(0.004) | 0.004(0.004) |
| year1992 | 0.008(0.002)*** | 0.019(0.003)*** | 0.032(0.004)*** | 0.026(0.004)*** | 0.015(0.003)*** |
| year1993 | 0.003(0.002) | 0.005(0.003) | 0.003(0.004) | 0(0.004) | -0.004(0.004) |
| year1994 | 0.01(0.002)*** | 0.019(0.003)*** | 0.026(0.004)*** | 0.02(0.004)*** | 0.011(0.004)*** |
| year1995 | 0.011(0.002)*** | 0.017(0.003)*** | 0.032(0.004)*** | 0.036(0.004)*** | 0.02(0.003)*** |
| year1996 | 0.012(0.002)*** | 0.026(0.003)*** | 0.036(0.004)*** | 0.03(0.004)*** | 0.017(0.003)*** |
| year1997 | 0.025(0.002)*** | 0.038(0.003)*** | 0.053(0.004)*** | 0.051(0.004)*** | 0.026(0.003)*** |
| year1998 | 0.024(0.002)*** | 0.038(0.003)*** | 0.061(0.004)*** | 0.055(0.004)*** | 0.027(0.004)*** |
| year1999 | 0.024(0.002)*** | 0.045(0.003)*** | 0.068(0.004)*** | 0.066(0.004)*** | 0.032(0.003)*** |
| year2000 | 0.025(0.002)*** | 0.05(0.003)*** | 0.073(0.004)*** | 0.07(0.004)*** | 0.039(0.003)*** |
| year2001 | 0.028(0.002)*** | 0.051(0.003)*** | 0.08(0.004)*** | 0.083(0.004)*** | 0.049(0.003)*** |
| year2002 | 0.029(0.002)*** | 0.067(0.003)*** | 0.098(0.004)*** | 0.101(0.004)*** | 0.056(0.003)*** |
| year2003 | 0.036(0.002)*** | 0.069(0.003)*** | 0.107(0.004)*** | 0.103(0.004)*** | 0.063(0.003)*** |
| year2004 | 0.039(0.002)*** | 0.084(0.003)*** | 0.129(0.004)*** | 0.126(0.004)*** | 0.074(0.003)*** |
| year2005 | 0.051(0.003)*** | 0.104(0.004)*** | 0.159(0.004)*** | 0.15(0.004)*** | 0.087(0.003)*** |
| year2006 | 0.054(0.003)*** | 0.111(0.004)*** | 0.17(0.004)*** | 0.157(0.004)*** | 0.091(0.003)*** |
| year2007 | 0.054(0.003)*** | 0.106(0.004)*** | 0.159(0.004)*** | 0.153(0.004)*** | 0.09(0.003)*** |
| year2008 | 0.05(0.003)*** | 0.099(0.004)*** | 0.154(0.004)*** | 0.142(0.004)*** | 0.087(0.003)*** |
| year2009 | 0.053(0.003)*** | 0.108(0.004)*** | 0.161(0.004)*** | 0.158(0.004)*** | 0.095(0.003)*** |
| year2010 | 0.065(0.003)*** | 0.131(0.004)*** | 0.194(0.004)*** | 0.174(0.004)*** | 0.101(0.003)*** |
| year2011 | 0.062(0.003)*** | 0.132(0.004)*** | 0.196(0.004)*** | 0.179(0.004)*** | 0.103(0.003)*** |
| year2012 | 0.078(0.003)*** | 0.154(0.004)*** | 0.218(0.004)*** | 0.191(0.004)*** | 0.108(0.003)*** |
| year2013 | 0.072(0.003)*** | 0.142(0.004)*** | 0.209(0.004)*** | 0.188(0.004)*** | 0.108(0.003)*** |
| year2014 | 0.079(0.003)*** | 0.155(0.004)*** | 0.227(0.004)*** | 0.201(0.004)*** | 0.113(0.003)*** |
| year2015 | 0.081(0.003)*** | 0.158(0.004)*** | 0.223(0.004)*** | 0.194(0.004)*** | 0.11(0.003)*** |
| year2016 | 0.082(0.003)*** | 0.156(0.004)*** | 0.222(0.004)*** | 0.194(0.004)*** | 0.108(0.003)*** |
| year2017 | 0.083(0.003)*** | 0.161(0.004)*** | 0.226(0.004)*** | 0.196(0.004)*** | 0.109(0.003)*** |
| year2018 | 0.084(0.003)*** | 0.159(0.004)*** | 0.222(0.004)*** | 0.19(0.004)*** | 0.105(0.003)*** |
| year2019 | 0.083(0.003)*** | 0.155(0.004)*** | 0.216(0.004)*** | 0.19(0.004)*** | 0.107(0.003)*** |
| age2 | -0.012(0.002)*** | -0.037(0.003)*** | -0.071(0.004)*** | -0.089(0.004)*** | -0.072(0.004)*** |
| age3 | -0.02(0.002)*** | -0.061(0.003)*** | -0.114(0.004)*** | -0.154(0.006)*** | -0.117(0.006)*** |
| region1 | 0.015(0.001)*** | 0.022(0.001)*** | 0.025(0.002)*** | 0.022(0.001)*** | 0.015(0.001)*** |
| region2 | 0.035(0.001)*** | 0.057(0.002)*** | 0.072(0.002)*** | 0.059(0.002)*** | 0.035(0.001)*** |
| region3 | 0.021(0.001)*** | 0.034(0.001)*** | 0.043(0.002)*** | 0.036(0.002)*** | 0.019(0.001)*** |
| region4 | 0.02(0.002)*** | 0.033(0.002)*** | 0.043(0.003)*** | 0.03(0.002)*** | 0.015(0.002)*** |
| region5 | 0.044(0.001)*** | 0.078(0.002)*** | 0.1(0.002)*** | 0.083(0.002)*** | 0.046(0.001)*** |
| region6 | 0.011(0.001)*** | 0.026(0.001)*** | 0.037(0.002)*** | 0.034(0.002)*** | 0.021(0.001)*** |
| region7 | 0.047(0.001)*** | 0.079(0.001)*** | 0.1(0.001)*** | 0.083(0.001)*** | 0.046(0.001)*** |
| region8 | 0.054(0.001)*** | 0.089(0.001)*** | 0.108(0.002)*** | 0.086(0.001)*** | 0.045(0.001)*** |
| region9 | 0.046(0.002)*** | 0.075(0.002)*** | 0.089(0.002)*** | 0.072(0.002)*** | 0.041(0.001)*** |
| Covariate | tau=0.1 | tau=0.25 | tau=0.5 | tau=0.75 | tau=0.9 |
| region10 | 0.028(0.001)*** | 0.04(0.001)*** | 0.048(0.002)*** | 0.04(0.002)*** | 0.021(0.001)*** |
| region11 | 0.03(0.001)*** | 0.049(0.002)*** | 0.061(0.002)*** | 0.051(0.002)*** | 0.03(0.001)*** |
| year1990age2 | 0.009(0.003)*** | 0.011(0.004)*** | 0.014(0.005)*** | 0.013(0.006)*** | 0.015(0.006)*** |
| year1991age2 | 0.017(0.003)*** | 0.028(0.004)*** | 0.041(0.005)*** | 0.05(0.006)*** | 0.039(0.006)*** |
| year1992age2 | 0.023(0.003)*** | 0.042(0.004)*** | 0.067(0.005)*** | 0.083(0.006)*** | 0.06(0.005)*** |
| year1993age2 | 0.027(0.003)*** | 0.055(0.004)*** | 0.088(0.005)*** | 0.099(0.006)*** | 0.082(0.005)*** |
| year1994age2 | 0.025(0.003)*** | 0.045(0.004)*** | 0.078(0.005)*** | 0.097(0.006)*** | 0.073(0.005)*** |
| year1995age2 | 0.022(0.003)*** | 0.051(0.004)*** | 0.082(0.005)*** | 0.085(0.006)*** | 0.069(0.005)*** |
| year1996age2 | 0.027(0.003)*** | 0.052(0.004)*** | 0.089(0.005)*** | 0.109(0.006)*** | 0.086(0.005)*** |
| year1997age2 | 0.026(0.003)*** | 0.057(0.004)*** | 0.1(0.005)*** | 0.113(0.006)*** | 0.094(0.005)*** |
| year1998age2 | 0.023(0.003)*** | 0.056(0.004)*** | 0.093(0.005)*** | 0.114(0.006)*** | 0.096(0.005)*** |
| year1999age2 | 0.032(0.003)*** | 0.06(0.004)*** | 0.099(0.006)*** | 0.107(0.006)*** | 0.093(0.005)*** |
| year2000age2 | 0.027(0.003)*** | 0.057(0.004)*** | 0.106(0.006)*** | 0.114(0.006)*** | 0.09(0.005)*** |
| year2001age2 | 0.028(0.003)*** | 0.063(0.004)*** | 0.105(0.006)*** | 0.113(0.006)*** | 0.091(0.005)*** |
| year2002age2 | 0.033(0.003)*** | 0.056(0.005)*** | 0.104(0.006)*** | 0.112(0.006)*** | 0.095(0.005)*** |
| year2003age2 | 0.031(0.003)*** | 0.06(0.005)*** | 0.1(0.006)*** | 0.118(0.006)*** | 0.09(0.005)*** |
| year2004age2 | 0.036(0.003)*** | 0.066(0.005)*** | 0.109(0.006)*** | 0.12(0.006)*** | 0.091(0.005)*** |
| year2005age2 | 0.027(0.004)*** | 0.054(0.005)*** | 0.082(0.006)*** | 0.101(0.006)*** | 0.08(0.005)*** |
| year2006age2 | 0.02(0.004)*** | 0.046(0.005)*** | 0.077(0.006)*** | 0.096(0.006)*** | 0.08(0.005)*** |
| year2007age2 | 0.021(0.004)*** | 0.052(0.005)*** | 0.086(0.006)*** | 0.102(0.006)*** | 0.084(0.005)*** |
| year2008age2 | 0.022(0.004)*** | 0.051(0.005)*** | 0.094(0.006)*** | 0.111(0.006)*** | 0.084(0.005)*** |
| year2009age2 | 0.019(0.004)*** | 0.045(0.005)*** | 0.088(0.006)*** | 0.101(0.006)*** | 0.08(0.005)*** |
| year2010age2 | 0.016(0.004)*** | 0.033(0.005)*** | 0.067(0.006)*** | 0.093(0.006)*** | 0.076(0.005)*** |
| year2011age2 | 0.012(0.004)** | 0.028(0.005)** | 0.065(0.006)** | 0.093(0.006)** | 0.078(0.005)** |
| year2012age2 | 0.001(0.004) | 0.015(0.005) | 0.05(0.006) | 0.081(0.006) | 0.075(0.005) |
| year2013age2 | 0.015(0.004)*** | 0.044(0.005)*** | 0.072(0.006)*** | 0.098(0.006)*** | 0.082(0.005)*** |
| year2014age2 | 0.007(0.004) | 0.031(0.005) | 0.063(0.006) | 0.088(0.005) | 0.076(0.005) |
| year2015age2 | 0.016(0.004)*** | 0.042(0.006)*** | 0.071(0.006)*** | 0.098(0.005)*** | 0.08(0.005)*** |
| year2016age2 | 0.01(0.004)* | 0.029(0.006)* | 0.064(0.006)* | 0.093(0.006)* | 0.081(0.005)* |
| year2017age2 | 0.011(0.004)** | 0.025(0.005)** | 0.066(0.006)** | 0.09(0.005)** | 0.078(0.005)** |
| year2018age2 | 0.004(0.004) | 0.021(0.005) | 0.056(0.006) | 0.09(0.006) | 0.079(0.005) |
| year2019age2 | 0.005(0.004) | 0.022(0.005) | 0.064(0.006) | 0.086(0.005) | 0.076(0.005) |
| year1990age3 | 0.01(0.003)*** | 0.015(0.004)*** | 0.016(0.005)*** | 0.03(0.008)*** | 0.024(0.007)*** |
| year1991age3 | 0.016(0.003)*** | 0.034(0.004)*** | 0.049(0.005)*** | 0.072(0.007)*** | 0.052(0.007)*** |
| year1992age3 | 0.018(0.003)*** | 0.041(0.004)*** | 0.062(0.005)*** | 0.1(0.008)*** | 0.07(0.007)*** |
| year1993age3 | 0.028(0.003)*** | 0.068(0.004)*** | 0.106(0.005)*** | 0.152(0.007)*** | 0.108(0.007)*** |
| year1994age3 | 0.03(0.003)*** | 0.067(0.004)*** | 0.11(0.006)*** | 0.156(0.007)*** | 0.105(0.007)*** |
| year1995age3 | 0.034(0.003)*** | 0.086(0.004)*** | 0.129(0.006)*** | 0.169(0.007)*** | 0.13(0.007)*** |
| year1996age3 | 0.044(0.003)*** | 0.094(0.004)*** | 0.144(0.006)*** | 0.192(0.007)*** | 0.141(0.007)*** |
| year1997age3 | 0.037(0.003)*** | 0.092(0.005)*** | 0.145(0.006)*** | 0.191(0.007)*** | 0.143(0.007)*** |
| year1998age3 | 0.043(0.003)*** | 0.106(0.005)*** | 0.165(0.006)*** | 0.214(0.007)*** | 0.16(0.007)*** |
| year1999age3 | 0.043(0.004)*** | 0.108(0.005)*** | 0.17(0.006)*** | 0.212(0.007)*** | 0.16(0.006)*** |
| year2000age3 | 0.05(0.004)*** | 0.116(0.005)*** | 0.185(0.006)*** | 0.219(0.007)*** | 0.158(0.006)*** |
| year2001age3 | 0.057(0.004)*** | 0.132(0.005)*** | 0.192(0.006)*** | 0.217(0.007)*** | 0.151(0.006)*** |
| year2002age3 | 0.052(0.004)*** | 0.111(0.005)*** | 0.176(0.006)*** | 0.201(0.007)*** | 0.151(0.006)*** |
| Covariate | tau=0.1 | tau=0.25 | tau=0.5 | tau=0.75 | tau=0.9 |
| year2003age3 | 0.048(0.004)*** | 0.116(0.005)*** | 0.182(0.006)*** | 0.211(0.007)*** | 0.153(0.006)*** |
| year2004age3 | 0.06(0.004)*** | 0.125(0.005)*** | 0.192(0.007)*** | 0.209(0.007)*** | 0.148(0.006)*** |
| year2005age3 | 0.043(0.004)*** | 0.099(0.005)*** | 0.149(0.007)*** | 0.188(0.007)*** | 0.135(0.006)*** |
| year2006age3 | 0.039(0.005)*** | 0.097(0.005)*** | 0.144(0.007)*** | 0.183(0.007)*** | 0.134(0.006)*** |
| year2007age3 | 0.03(0.005)*** | 0.087(0.006)*** | 0.142(0.007)*** | 0.175(0.007)*** | 0.129(0.006)*** |
| year2008age3 | 0.032(0.004)*** | 0.098(0.005)*** | 0.149(0.007)*** | 0.187(0.007)*** | 0.133(0.006)*** |
| year2009age3 | 0.03(0.005)*** | 0.089(0.006)*** | 0.152(0.007)*** | 0.175(0.007)*** | 0.127(0.006)*** |
| year2010age3 | 0.017(0.005)*** | 0.061(0.006)*** | 0.104(0.007)*** | 0.151(0.007)*** | 0.118(0.006)*** |
| year2011age3 | 0.017(0.004)*** | 0.05(0.006)*** | 0.101(0.007)*** | 0.148(0.007)*** | 0.118(0.006)*** |
| year2012age3 | 0.009(0.005) | 0.043(0.006) | 0.089(0.007) | 0.147(0.007) | 0.117(0.006) |
| year2013age3 | 0.014(0.005)** | 0.053(0.006)** | 0.104(0.007)** | 0.15(0.007)** | 0.116(0.006)** |
| year2014age3 | 0.013(0.005)** | 0.048(0.006)** | 0.098(0.007)** | 0.146(0.007)** | 0.118(0.006)** |
| year2015age3 | 0.015(0.005)** | 0.053(0.006)** | 0.101(0.007)** | 0.152(0.007)** | 0.121(0.006)** |
| year2016age3 | 0.005(0.005) | 0.048(0.006) | 0.093(0.007) | 0.145(0.007) | 0.12(0.006) |
| year2017age3 | -0.001(0.005) | 0.037(0.006) | 0.082(0.007) | 0.14(0.007) | 0.121(0.006) |
| year2018age3 | 0.01(0.005)* | 0.038(0.006)* | 0.083(0.006)* | 0.139(0.007)* | 0.116(0.006)* |
| year2019age3 | 0.005(0.005) | 0.041(0.006) | 0.078(0.007) | 0.132(0.007) | 0.115(0.006) |

Supplementary Table 3.13: Coefficient estimates (standard errors) for different quantiles (tau) for SAR, boys; **p<*0.05, ***p<*0.01,****p<*0.001, age2=10-12 years, age3=13-15 years.

| Covariate | tau=0.1 | tau=0.25 | tau=0.5 | tau=0.75 | tau=0.9 |
| --- | --- | --- | --- | --- | --- |
| (Intercept) | 0.1(0.002)*** | 0.234(0.004)*** | 0.446(0.004)*** | 0.686(0.003)*** | 0.873(0.002)*** |
| year1990 | 0.002(0.003) | 0.011(0.005) | 0.005(0.005) | 0.003(0.003) | 0.005(0.003) |
| year1991 | 0.012(0.003)*** | 0.019(0.005)*** | 0.033(0.004)*** | 0.025(0.004)*** | 0.018(0.002)*** |
| year1992 | 0.012(0.003)*** | 0.016(0.005)*** | 0.031(0.004)*** | 0.029(0.004)*** | 0.015(0.002)*** |
| year1993 | 0.008(0.003)** | 0.011(0.005)** | 0.026(0.004)** | 0.024(0.004)** | 0.016(0.002)** |
| year1994 | 0.012(0.003)*** | 0.02(0.005)*** | 0.041(0.004)*** | 0.033(0.003)*** | 0.021(0.002)*** |
| year1995 | 0.015(0.003)*** | 0.024(0.005)*** | 0.057(0.005)*** | 0.053(0.003)*** | 0.024(0.002)*** |
| year1996 | 0.02(0.003)*** | 0.049(0.007)*** | 0.066(0.005)*** | 0.054(0.003)*** | 0.028(0.002)*** |
| year1997 | 0.015(0.003)*** | 0.034(0.006)*** | 0.064(0.005)*** | 0.053(0.004)*** | 0.027(0.002)*** |
| year1998 | 0.014(0.003)*** | 0.022(0.005)*** | 0.047(0.005)*** | 0.045(0.004)*** | 0.028(0.002)*** |
| year1999 | 0.013(0.003)*** | 0.025(0.005)*** | 0.063(0.005)*** | 0.055(0.003)*** | 0.028(0.002)*** |
| year2000 | 0.019(0.003)*** | 0.026(0.005)*** | 0.07(0.005)*** | 0.053(0.004)*** | 0.029(0.003)*** |
| year2001 | 0.012(0.003)*** | 0.013(0.005)*** | 0.041(0.005)*** | 0.037(0.004)*** | 0.022(0.002)*** |
| year2002 | 0.012(0.003)*** | 0.012(0.005)*** | 0.036(0.004)*** | 0.034(0.004)*** | 0.021(0.002)*** |
| year2003 | 0.013(0.003)*** | 0.019(0.005)*** | 0.057(0.005)*** | 0.049(0.004)*** | 0.026(0.002)*** |
| year2004 | 0.016(0.003)*** | 0.024(0.005)*** | 0.072(0.005)*** | 0.061(0.004)*** | 0.032(0.002)*** |
| year2005 | 0.013(0.003)*** | 0.018(0.005)*** | 0.062(0.005)*** | 0.053(0.004)*** | 0.031(0.002)*** |
| year2006 | 0.01(0.003)*** | 0.015(0.005)*** | 0.057(0.005)*** | 0.053(0.004)*** | 0.031(0.002)*** |
| year2007 | 0.002(0.003) | 0(0.007) | 0.036(0.005) | 0.037(0.004) | 0.026(0.003) |
| year2008 | -0.011(0.003)*** | -0.024(0.005)*** | 0.026(0.005)*** | 0.026(0.004)*** | 0.017(0.003)*** |
| year2009 | -0.01(0.003)** | -0.02(0.005)** | 0.035(0.005)** | 0.036(0.004)** | 0.023(0.003)** |
| year2010 | -0.01(0.003)** | -0.02(0.005)** | 0.036(0.005)** | 0.039(0.004)** | 0.026(0.003)** |
| year2011 | -0.001(0.004) | -0.019(0.005) | 0.033(0.005) | 0.033(0.003) | 0.021(0.003) |
| year2012 | -0.012(0.003)*** | -0.025(0.005)*** | 0.023(0.005)*** | 0.024(0.004)*** | 0.016(0.003)*** |
| year2013 | -0.001(0.004) | -0.018(0.005) | 0.034(0.004) | 0.033(0.003) | 0.021(0.003) |
| year2014 | -0.009(0.003)** | -0.008(0.007)** | 0.036(0.004)** | 0.036(0.003)** | 0.023(0.003)** |
| year2015 | -0.011(0.003)*** | -0.024(0.005)*** | 0.029(0.005)*** | 0.029(0.003)*** | 0.021(0.003)*** |
| year2016 | -0.012(0.003)*** | -0.024(0.005)*** | 0.026(0.005)*** | 0.032(0.003)*** | 0.021(0.002)*** |
| year2017 | -0.011(0.003)*** | -0.021(0.005)*** | 0.034(0.004)*** | 0.035(0.003)*** | 0.023(0.002)*** |
| year2018 | -0.012(0.003)*** | -0.025(0.005)*** | 0.025(0.004)*** | 0.027(0.003)*** | 0.018(0.003)*** |
| year2019 | -0.014(0.003)*** | -0.028(0.005)*** | 0.022(0.004)*** | 0.026(0.003)*** | 0.018(0.002)*** |
| age2 | -0.001(0.003) | -0.004(0.005) | 0.018(0.004) | 0.015(0.004) | -0.003(0.003) |
| age3 | 0.013(0.003)*** | 0.023(0.006)*** | 0.035(0.005)*** | 0.028(0.004)*** | -0.007(0.004)*** |
| region1 | 0.016(0.001)*** | 0.042(0.001)*** | 0.049(0.002)*** | 0.04(0.001)*** | 0.018(0.001)*** |
| region2 | 0.043(0.001)*** | 0.096(0.002)*** | 0.106(0.002)*** | 0.074(0.002)*** | 0.034(0.001)*** |
| region3 | -0.005(0.001)*** | 0(0.001)*** | -0.002(0.002)*** | -0.002(0.001)*** | 0(0.001)*** |
| region4 | -0.019(0.002)*** | -0.023(0.003)*** | -0.026(0.003)*** | -0.019(0.002)*** | -0.01(0.002)*** |
| region5 | 0.016(0.001)*** | 0.044(0.002)*** | 0.049(0.002)*** | 0.043(0.002)*** | 0.019(0.001)*** |
| region6 | -0.009(0.001)*** | -0.005(0.002)*** | -0.011(0.002)*** | -0.009(0.002)*** | -0.006(0.001)*** |
| region7 | -0.009(0.001)*** | 0(0.001)*** | 0.013(0.002)*** | 0.016(0.001)*** | 0.009(0.001)*** |
| region8 | 0.001(0.001) | 0.011(0.002) | 0.022(0.002) | 0.018(0.001) | 0.008(0.001) |
| region9 | -0.011(0.002)*** | -0.001(0.002)*** | -0.001(0.002)*** | 0.003(0.002)*** | 0.001(0.001)*** |
| region10 | -0.01(0.001)*** | -0.004(0.002)*** | -0.008(0.002)*** | -0.006(0.002)*** | -0.005(0.001)*** |
| region11 | -0.02(0.001)*** | -0.025(0.002)*** | -0.015(0.002)*** | -0.006(0.002)*** | -0.002(0.001)*** |
| year1990age2 | 0.001(0.004) | 0.006(0.007) | 0.003(0.006) | 0(0.005) | -0.001(0.003) |
| year1991age2 | 0.006(0.004) | 0.023(0.006) | 0.013(0.006) | 0.004(0.005) | -0.007(0.003) |
| year1992age2 | 0.02(0.004)*** | 0.057(0.006)*** | 0.035(0.005)*** | 0.023(0.005)*** | 0.013(0.003)*** |
| year1993age2 | 0.016(0.004)*** | 0.052(0.007)*** | 0.034(0.006)*** | 0.022(0.005)*** | 0.006(0.003)*** |
| year1994age2 | 0.022(0.004)*** | 0.059(0.006)*** | 0.04(0.006)*** | 0.025(0.005)*** | 0.01(0.003)*** |
| year1995age2 | 0.022(0.004)*** | 0.056(0.006)*** | 0.021(0.006)*** | 0.011(0.005)*** | 0.01(0.003)*** |
| Covariate | tau=0.1 | tau=0.25 | tau=0.5 | tau=0.75 | tau=0.9 |
| year1996age2 | 0.021(0.004)*** | 0.043(0.008)*** | 0.043(0.006)*** | 0.027(0.005)*** | 0.014(0.003)*** |
| year1997age2 | 0.025(0.004)*** | 0.053(0.007)*** | 0.04(0.007)*** | 0.016(0.005)*** | 0.009(0.003)*** |
| year1998age2 | 0.023(0.004)*** | 0.058(0.006)*** | 0.034(0.006)*** | 0.018(0.005)*** | 0.006(0.003)*** |
| year1999age2 | 0.024(0.004)*** | 0.061(0.007)*** | 0.036(0.007)*** | 0.012(0.005)*** | 0.01(0.003)*** |
| year2000age2 | 0.007(0.004) | 0.039(0.007) | -0.002(0.006) | -0.001(0.005) | 0(0.003) |
| year2001age2 | 0.003(0.004) | 0.022(0.007) | 0.008(0.006) | -0.001(0.005) | -0.003(0.003) |
| year2002age2 | 0.001(0.004) | 0.015(0.007) | 0.007(0.007) | -0.003(0.005) | -0.004(0.003) |
| year2003age2 | 0(0.004) | 0.012(0.007) | -0.005(0.007) | -0.011(0.005) | -0.004(0.003) |
| year2004age2 | -0.004(0.004) | 0.011(0.007) | -0.016(0.007) | -0.023(0.005) | -0.01(0.003) |
| year2005age2 | -0.021(0.004)*** | -0.022(0.007)*** | -0.045(0.006)*** | -0.039(0.005)*** | -0.02(0.003)*** |
| year2006age2 | -0.018(0.004)*** | -0.022(0.007)*** | -0.049(0.007)*** | -0.04(0.006)*** | -0.02(0.003)*** |
| year2007age2 | -0.018(0.004)*** | -0.025(0.008)*** | -0.042(0.006)*** | -0.039(0.005)*** | -0.02(0.003)*** |
| year2008age2 | -0.009(0.004)* | -0.018(0.006)* | -0.061(0.006)* | -0.042(0.005)* | -0.016(0.003)* |
| year2009age2 | -0.015(0.004)*** | -0.019(0.007)*** | -0.069(0.007)*** | -0.055(0.005)*** | -0.023(0.003)*** |
| year2010age2 | -0.017(0.004)*** | -0.022(0.007)*** | -0.061(0.007)*** | -0.049(0.006)*** | -0.022(0.003)*** |
| year2011age2 | -0.024(0.004)*** | -0.021(0.007)*** | -0.054(0.007)*** | -0.044(0.005)*** | -0.017(0.003)*** |
| year2012age2 | -0.019(0.004)*** | -0.02(0.006)*** | -0.059(0.007)*** | -0.043(0.005)*** | -0.014(0.003)*** |
| year2013age2 | -0.024(0.004)*** | -0.022(0.007)*** | -0.054(0.007)*** | -0.046(0.005)*** | -0.017(0.003)*** |
| year2014age2 | -0.018(0.004)*** | -0.034(0.008)*** | -0.066(0.007)*** | -0.05(0.005)*** | -0.022(0.003)*** |
| year2015age2 | -0.015(0.004)*** | -0.018(0.006)*** | -0.063(0.007)*** | -0.045(0.005)*** | -0.018(0.003)*** |
| year2016age2 | -0.016(0.004)*** | -0.017(0.006)*** | -0.06(0.007)*** | -0.049(0.005)*** | -0.019(0.003)*** |
| year2017age2 | -0.021(0.004)*** | -0.023(0.006)*** | -0.069(0.006)*** | -0.053(0.005)*** | -0.023(0.003)*** |
| year2018age2 | -0.02(0.004)*** | -0.024(0.006)*** | -0.063(0.006)*** | -0.044(0.005)*** | -0.016(0.003)*** |
| year2019age2 | -0.025(0.004)*** | -0.034(0.006)*** | -0.077(0.006)*** | -0.052(0.005)*** | -0.022(0.003)*** |
| year1990age3 | 0.003(0.005) | 0.003(0.007) | 0.013(0.007) | 0.018(0.006) | 0.02(0.005) |
| year1991age3 | 0.001(0.005) | 0.007(0.007) | 0.006(0.007) | 0.013(0.006) | 0.014(0.005) |
| year1992age3 | 0.008(0.005) | 0.028(0.007) | 0.036(0.006) | 0.03(0.006) | 0.032(0.004) |
| year1993age3 | 0.011(0.004)* | 0.034(0.007)* | 0.038(0.006)* | 0.036(0.006)* | 0.027(0.004)* |
| year1994age3 | 0.013(0.004)** | 0.039(0.007)** | 0.032(0.006)** | 0.029(0.005)** | 0.025(0.004)** |
| year1995age3 | 0.014(0.004)** | 0.041(0.007)** | 0.022(0.007)** | 0.015(0.006)** | 0.023(0.004)** |
| year1996age3 | 0.014(0.005)** | 0.021(0.008)** | 0.02(0.007)** | 0.016(0.006)** | 0.02(0.004)** |
| year1997age3 | 0.014(0.004)** | 0.03(0.008)** | 0.019(0.007)** | 0.019(0.006)** | 0.021(0.004)** |
| year1998age3 | 0.01(0.004)* | 0.034(0.007)* | 0.028(0.007)* | 0.021(0.006)* | 0.019(0.004)* |
| year1999age3 | 0.007(0.005) | 0.021(0.007) | 0.004(0.007) | 0.004(0.006) | 0.015(0.004) |
| year2000age3 | -0.004(0.004) | 0.015(0.007) | -0.009(0.007) | 0.002(0.006) | 0.014(0.005) |
| year2001age3 | -0.009(0.005) | 0.004(0.007) | -0.003(0.007) | 0.002(0.006) | 0.012(0.005) |
| year2002age3 | -0.017(0.005)*** | -0.016(0.007)*** | -0.021(0.007)*** | -0.012(0.006)*** | 0.004(0.005)*** |
| year2003age3 | -0.022(0.005)*** | -0.029(0.007)*** | -0.049(0.007)*** | -0.032(0.006)*** | -0.002(0.005)*** |
| year2004age3 | -0.023(0.005)*** | -0.029(0.007)*** | -0.056(0.007)*** | -0.04(0.006)*** | -0.003(0.005)*** |
| year2005age3 | -0.033(0.005)*** | -0.045(0.007)*** | -0.07(0.007)*** | -0.052(0.006)*** | -0.013(0.005)*** |
| year2006age3 | -0.034(0.005)*** | -0.053(0.008)*** | -0.072(0.007)*** | -0.058(0.006)*** | -0.022(0.005)*** |
| year2007age3 | -0.037(0.005)*** | -0.049(0.009)*** | -0.068(0.007)*** | -0.052(0.006)*** | -0.017(0.005)*** |
| year2008age3 | -0.03(0.005)*** | -0.044(0.007)*** | -0.079(0.008)*** | -0.06(0.007)*** | -0.021(0.005)*** |
| year2009age3 | -0.037(0.005)*** | -0.054(0.007)*** | -0.099(0.008)*** | -0.078(0.007)*** | -0.036(0.005)*** |
| year2010age3 | -0.042(0.004)*** | -0.061(0.007)*** | -0.101(0.008)*** | -0.082(0.007)*** | -0.035(0.005)*** |
| year2011age3 | -0.05(0.005)*** | -0.069(0.007)*** | -0.116(0.007)*** | -0.087(0.007)*** | -0.04(0.006)*** |
| year2012age3 | -0.041(0.004)*** | -0.068(0.007)*** | -0.104(0.008)*** | -0.08(0.007)*** | -0.037(0.005)*** |
| year2013age3 | -0.049(0.005)*** | -0.066(0.007)*** | -0.1(0.007)*** | -0.077(0.007)*** | -0.03(0.006)*** |
| year2014age3 | -0.039(0.005)*** | -0.072(0.009)*** | -0.101(0.007)*** | -0.079(0.007)*** | -0.034(0.005)*** |
| year2015age3 | -0.035(0.005)*** | -0.052(0.007)*** | -0.086(0.008)*** | -0.065(0.007)*** | -0.03(0.006)*** |
| year2016age3 | -0.035(0.004)*** | -0.055(0.007)*** | -0.086(0.008)*** | -0.077(0.006)*** | -0.033(0.006)*** |
| year2017age3 | -0.038(0.004)*** | -0.062(0.007)*** | -0.093(0.007)*** | -0.077(0.007)*** | -0.037(0.006)*** |
| year2018age3 | -0.037(0.004)*** | -0.053(0.007)*** | -0.087(0.007)*** | -0.067(0.007)*** | -0.024(0.005)*** |
| year2019age3 | -0.04(0.004)*** | -0.066(0.007)*** | -0.103(0.007)*** | -0.078(0.007)*** | -0.028(0.005)*** |

Supplementary Table 3.14: Coefficient estimates (standard errors) for different quantiles (tau) for SAR, girls; **p<*0.05, ***p<*0.01,****p<*0.001, age2=10-12 years, age3=13-15 years.

| Covariate | tau=0.1 | tau=0.25 | tau=0.5 | tau=0.75 | tau=0.9 |
| --- | --- | --- | --- | --- | --- |
| (Intercept) | 0.071(0.002)*** | 0.187(0.002)*** | 0.406(0.003)*** | 0.642(0.004)*** | 0.829(0.002)*** |
| year1990 | 0.002(0.002) | 0.007(0.002) | 0.008(0.003) | 0.012(0.005) | 0.003(0.003) |
| year1991 | 0.004(0.003) | 0.013(0.003) | 0.025(0.004) | 0.035(0.005) | 0.016(0.003) |
| year1992 | 0.017(0.003)*** | 0.036(0.003)*** | 0.058(0.003)*** | 0.071(0.004)*** | 0.035(0.003)*** |
| year1993 | 0.007(0.002)** | 0.019(0.003)** | 0.039(0.004)** | 0.053(0.005)** | 0.028(0.003)** |
| year1994 | 0.008(0.002)*** | 0.018(0.003)*** | 0.034(0.004)*** | 0.047(0.005)*** | 0.023(0.003)*** |
| year1995 | 0.007(0.003)** | 0.019(0.003)** | 0.046(0.003)** | 0.051(0.005)** | 0.03(0.003)** |
| year1996 | 0.01(0.003)*** | 0.025(0.003)*** | 0.048(0.003)*** | 0.058(0.005)*** | 0.028(0.003)*** |
| year1997 | 0.005(0.002)* | 0.02(0.003)* | 0.043(0.004)* | 0.057(0.005)* | 0.032(0.003)* |
| year1998 | 0.003(0.002) | 0.013(0.003) | 0.032(0.004) | 0.047(0.005) | 0.025(0.003) |
| year1999 | 0.006(0.003)* | 0.021(0.003)* | 0.05(0.004)* | 0.062(0.005)* | 0.035(0.003)* |
| year2000 | 0.007(0.003)** | 0.025(0.003)** | 0.052(0.003)** | 0.07(0.005)** | 0.038(0.003)** |
| year2001 | 0.005(0.003) | 0.021(0.003) | 0.051(0.003) | 0.066(0.005) | 0.037(0.003) |
| year2002 | 0.004(0.003) | 0.023(0.003) | 0.052(0.004) | 0.069(0.005) | 0.044(0.003) |
| year2003 | 0.005(0.003) | 0.03(0.003) | 0.058(0.004) | 0.075(0.005) | 0.047(0.003) |
| year2004 | 0.007(0.003)** | 0.036(0.003)** | 0.07(0.003)** | 0.085(0.005)** | 0.051(0.003)** |
| year2005 | 0.003(0.003) | 0.028(0.003) | 0.062(0.004) | 0.071(0.005) | 0.042(0.003) |
| year2006 | 0.006(0.003)* | 0.023(0.003)* | 0.057(0.004)* | 0.072(0.005)* | 0.044(0.003)* |
| year2007 | 0.001(0.002) | 0.021(0.003) | 0.058(0.004) | 0.073(0.005) | 0.045(0.003) |
| year2008 | -0.006(0.002)* | 0.013(0.003)* | 0.046(0.004)* | 0.059(0.005)* | 0.033(0.003)* |
| year2009 | -0.002(0.002) | 0.022(0.003) | 0.057(0.004) | 0.069(0.005) | 0.041(0.003) |
| year2010 | 0.006(0.003)* | 0.038(0.003)* | 0.07(0.003)* | 0.088(0.005)* | 0.05(0.003)* |
| year2011 | 0.01(0.003)*** | 0.043(0.003)*** | 0.074(0.003)*** | 0.086(0.005)*** | 0.051(0.003)*** |
| year2012 | 0.008(0.003)** | 0.041(0.003)** | 0.073(0.003)** | 0.094(0.005)** | 0.052(0.003)** |
| year2013 | 0.009(0.003)*** | 0.047(0.003)*** | 0.078(0.004)*** | 0.099(0.005)*** | 0.056(0.003)*** |
| year2014 | 0.009(0.003)*** | 0.046(0.003)*** | 0.08(0.004)*** | 0.105(0.004)*** | 0.062(0.003)*** |
| year2015 | 0.007(0.003)* | 0.047(0.003)* | 0.08(0.004)* | 0.103(0.004)* | 0.063(0.003)* |
| year2016 | 0.01(0.003)*** | 0.051(0.003)*** | 0.083(0.003)*** | 0.107(0.004)*** | 0.068(0.003)*** |
| year2017 | 0.014(0.003)*** | 0.06(0.003)*** | 0.098(0.004)*** | 0.12(0.004)*** | 0.073(0.003)*** |
| year2018 | 0.02(0.003)*** | 0.079(0.004)*** | 0.117(0.004)*** | 0.131(0.004)*** | 0.078(0.003)*** |
| year2019 | 0.021(0.003)*** | 0.075(0.004)*** | 0.122(0.004)*** | 0.136(0.004)*** | 0.083(0.003)*** |
| age2 | 0.01(0.003)*** | 0.014(0.003)*** | 0.006(0.004)*** | 0.018(0.005)*** | 0.011(0.003)*** |
| age3 | 0.01(0.003)*** | 0.012(0.004)*** | 0.021(0.004)*** | 0.031(0.005)*** | 0.02(0.004)*** |
| region1 | 0.015(0.001)*** | 0.028(0.001)*** | 0.04(0.001)*** | 0.039(0.002)*** | 0.023(0.001)*** |
| region2 | 0.04(0.001)*** | 0.065(0.002)*** | 0.072(0.002)*** | 0.06(0.002)*** | 0.031(0.001)*** |
| region3 | 0(0.001) | 0(0.001) | -0.004(0.001) | -0.009(0.002) | -0.01(0.001) |
| region4 | 0.02(0.002)*** | 0.032(0.002)*** | 0.036(0.003)*** | 0.029(0.002)*** | 0.016(0.002)*** |
| region5 | 0.028(0.001)*** | 0.06(0.002)*** | 0.075(0.002)*** | 0.066(0.002)*** | 0.037(0.001)*** |
| region6 | 0.002(0.001) | 0.004(0.001) | 0.005(0.002) | 0(0.002) | -0.003(0.001) |
| region7 | 0.023(0.001)*** | 0.048(0.001)*** | 0.069(0.001)*** | 0.068(0.001)*** | 0.041(0.001)*** |
| region8 | 0.023(0.001)*** | 0.04(0.001)*** | 0.051(0.001)*** | 0.045(0.002)*** | 0.024(0.001)*** |
| region9 | 0.002(0.001) | 0.013(0.002) | 0.019(0.002) | 0.019(0.002) | 0.01(0.002) |
| region10 | 0.016(0.001)*** | 0.029(0.001)*** | 0.04(0.002)*** | 0.037(0.002)*** | 0.022(0.001)*** |
| region11 | 0.015(0.001)*** | 0.048(0.002)*** | 0.081(0.002)*** | 0.084(0.002)*** | 0.053(0.001)*** |
| year1990age2 | -0.001(0.004) | -0.008(0.005) | -0.005(0.006) | -0.008(0.006) | 0.001(0.004) |
| year1991age2 | 0.011(0.004)** | 0.005(0.004)** | 0(0.006)** | -0.015(0.006)** | -0.006(0.004)** |
| year1992age2 | 0.016(0.004)*** | 0.025(0.005)*** | 0.021(0.005)*** | -0.012(0.006)*** | 0.003(0.004)*** |
| year1993age2 | 0.017(0.004)*** | 0.027(0.005)*** | 0.023(0.006)*** | -0.004(0.006)*** | 0.003(0.004)*** |
| year1994age2 | 0.018(0.004)*** | 0.034(0.005)*** | 0.03(0.006)*** | 0.008(0.006)*** | 0.012(0.004)*** |
| year1995age2 | 0.015(0.004)*** | 0.028(0.005)*** | 0.02(0.006)*** | 0.004(0.006)*** | 0.006(0.004)*** |
| year1996age2 | 0.021(0.004)*** | 0.032(0.005)*** | 0.026(0.005)*** | 0.002(0.006)*** | 0.01(0.004)*** |
| Covariate | tau=0.1 | tau=0.25 | tau=0.5 | tau=0.75 | tau=0.9 |
| year1997age2 | 0.021(0.004)*** | 0.03(0.005)*** | 0.025(0.006)*** | -0.001(0.006)*** | 0.005(0.004)*** |
| year1998age2 | 0.011(0.004)** | 0.023(0.005)** | 0.024(0.006)** | 0.004(0.006)** | 0.007(0.004)** |
| year1999age2 | 0.009(0.004)* | 0.018(0.005)* | 0.008(0.006)* | -0.009(0.006)* | -0.002(0.004)* |
| year2000age2 | 0.004(0.004) | 0.006(0.005) | 0(0.006) | -0.026(0.006) | -0.01(0.004) |
| year2001age2 | 0.003(0.004) | 0.001(0.005) | -0.014(0.006) | -0.023(0.006) | -0.004(0.004) |
| year2002age2 | 0.004(0.004) | 0.007(0.005) | -0.004(0.006) | -0.019(0.006) | -0.008(0.004) |
| year2003age2 | -0.003(0.004) | -0.004(0.005) | -0.016(0.006) | -0.029(0.006) | -0.009(0.004) |
| year2004age2 | 0(0.004) | -0.014(0.005) | -0.027(0.006) | -0.036(0.006) | -0.013(0.004) |
| year2005age2 | -0.016(0.004)*** | -0.028(0.005)*** | -0.046(0.006)*** | -0.044(0.006)*** | -0.016(0.004)*** |
| year2006age2 | -0.017(0.004)*** | -0.025(0.005)*** | -0.041(0.006)*** | -0.041(0.006)*** | -0.011(0.004)*** |
| year2007age2 | -0.02(0.004)*** | -0.036(0.005)*** | -0.053(0.006)*** | -0.05(0.007)*** | -0.019(0.005)*** |
| year2008age2 | -0.022(0.003)*** | -0.039(0.005)*** | -0.059(0.006)*** | -0.05(0.007)*** | -0.018(0.005)*** |
| year2009age2 | -0.024(0.004)*** | -0.044(0.005)*** | -0.066(0.006)*** | -0.061(0.007)*** | -0.023(0.005)*** |
| year2010age2 | -0.023(0.004)*** | -0.041(0.005)*** | -0.062(0.006)*** | -0.062(0.006)*** | -0.028(0.004)*** |
| year2011age2 | -0.024(0.004)*** | -0.047(0.005)*** | -0.055(0.006)*** | -0.046(0.006)*** | -0.013(0.004)*** |
| year2012age2 | -0.027(0.004)*** | -0.043(0.005)*** | -0.049(0.006)*** | -0.05(0.006)*** | -0.014(0.004)*** |
| year2013age2 | -0.018(0.004)*** | -0.033(0.005)*** | -0.033(0.006)*** | -0.041(0.006)*** | -0.01(0.004)*** |
| year2014age2 | -0.019(0.004)*** | -0.028(0.005)*** | -0.027(0.006)*** | -0.039(0.006)*** | -0.011(0.004)*** |
| year2015age2 | -0.012(0.004)** | -0.024(0.005)** | -0.02(0.006)** | -0.029(0.006)** | -0.009(0.004)** |
| year2016age2 | -0.017(0.004)*** | -0.026(0.005)*** | -0.019(0.006)*** | -0.025(0.006)*** | -0.011(0.004)*** |
| year2017age2 | -0.019(0.004)*** | -0.031(0.006)*** | -0.026(0.006)*** | -0.028(0.006)*** | -0.007(0.004)*** |
| year2018age2 | -0.017(0.004)*** | -0.035(0.006)*** | -0.028(0.006)*** | -0.031(0.006)*** | -0.009(0.004)*** |
| year2019age2 | -0.025(0.004)*** | -0.032(0.006)*** | -0.031(0.006)*** | -0.036(0.006)*** | -0.011(0.004)*** |
| year1990age3 | 0.009(0.004)* | 0.02(0.005)* | 0.018(0.006)* | 0.011(0.007)* | 0.014(0.005)* |
| year1991age3 | 0.019(0.004)*** | 0.028(0.005)*** | 0.017(0.006)*** | 0(0.007)*** | 0.005(0.005)*** |
| year1992age3 | 0.018(0.005)*** | 0.027(0.006)*** | 0.012(0.006)*** | -0.016(0.006)*** | -0.005(0.005)*** |
| year1993age3 | 0.034(0.004)*** | 0.05(0.005)*** | 0.032(0.006)*** | 0.002(0.006)*** | 0.005(0.005)*** |
| year1994age3 | 0.035(0.004)*** | 0.052(0.005)*** | 0.029(0.006)*** | 0.004(0.006)*** | 0.009(0.005)*** |
| year1995age3 | 0.032(0.004)*** | 0.046(0.005)*** | 0.021(0.006)*** | 0.001(0.007)*** | 0.003(0.005)*** |
| year1996age3 | 0.038(0.004)*** | 0.054(0.006)*** | 0.038(0.006)*** | 0.007(0.007)*** | 0.013(0.005)*** |
| year1997age3 | 0.038(0.004)*** | 0.048(0.005)*** | 0.031(0.006)*** | -0.001(0.007)*** | 0.002(0.005)*** |
| year1998age3 | 0.03(0.004)*** | 0.048(0.006)*** | 0.035(0.006)*** | 0.008(0.007)*** | 0.008(0.005)*** |
| year1999age3 | 0.028(0.004)*** | 0.039(0.006)*** | 0.014(0.006)*** | -0.012(0.007)*** | -0.003(0.005)*** |
| year2000age3 | 0.019(0.004)*** | 0.02(0.006)*** | 0.003(0.006)*** | -0.024(0.007)*** | -0.009(0.005)*** |
| year2001age3 | 0.008(0.004) | 0.01(0.005) | -0.012(0.006) | -0.026(0.007) | -0.006(0.005) |
| year2002age3 | 0(0.004) | -0.007(0.005) | -0.029(0.006) | -0.037(0.007) | -0.022(0.005) |
| year2003age3 | -0.011(0.004)** | -0.023(0.005)** | -0.042(0.006)** | -0.047(0.007)** | -0.025(0.005)** |
| year2004age3 | -0.012(0.004)** | -0.032(0.006)** | -0.058(0.006)** | -0.058(0.007)** | -0.028(0.005)** |
| year2005age3 | -0.017(0.004)*** | -0.04(0.005)*** | -0.069(0.006)*** | -0.06(0.007)*** | -0.025(0.005)*** |
| year2006age3 | -0.021(0.004)*** | -0.038(0.005)*** | -0.066(0.006)*** | -0.065(0.007)*** | -0.029(0.006)*** |
| year2007age3 | -0.018(0.004)*** | -0.047(0.005)*** | -0.089(0.006)*** | -0.078(0.007)*** | -0.038(0.006)*** |
| year2008age3 | -0.019(0.004)*** | -0.048(0.005)*** | -0.088(0.006)*** | -0.073(0.008)*** | -0.034(0.006)*** |
| year2009age3 | -0.023(0.004)*** | -0.061(0.005)*** | -0.102(0.006)*** | -0.087(0.007)*** | -0.041(0.006)*** |
| year2010age3 | -0.031(0.004)*** | -0.072(0.005)*** | -0.108(0.006)*** | -0.097(0.007)*** | -0.042(0.006)*** |
| year2011age3 | -0.036(0.004)*** | -0.078(0.005)*** | -0.109(0.006)*** | -0.09(0.007)*** | -0.041(0.006)*** |
| year2012age3 | -0.033(0.004)*** | -0.071(0.005)*** | -0.1(0.006)*** | -0.09(0.007)*** | -0.041(0.006)*** |
| year2013age3 | -0.032(0.004)*** | -0.068(0.006)*** | -0.096(0.007)*** | -0.083(0.007)*** | -0.027(0.005)*** |
| year2014age3 | -0.027(0.004)*** | -0.058(0.006)*** | -0.078(0.006)*** | -0.078(0.007)*** | -0.03(0.005)*** |
| year2015age3 | -0.022(0.004)*** | -0.047(0.006)*** | -0.061(0.007)*** | -0.058(0.007)*** | -0.022(0.005)*** |
| year2016age3 | -0.022(0.004)*** | -0.044(0.006)*** | -0.052(0.007)*** | -0.051(0.007)*** | -0.016(0.005)*** |
| year2017age3 | -0.021(0.004)*** | -0.041(0.006)*** | -0.049(0.007)*** | -0.054(0.007)*** | -0.016(0.005)*** |
| year2018age3 | -0.018(0.005)*** | -0.044(0.007)*** | -0.048(0.007)*** | -0.05(0.007)*** | -0.016(0.005)*** |
| year2019age3 | -0.029(0.004)*** | -0.054(0.007)*** | -0.058(0.007)*** | -0.049(0.007)*** | -0.019(0.005)*** |

Supplementary Table 3.15: Coefficient estimates (standard errors) for different quantiles (tau) for BAH, boys; **p<*0.05, ***p<*0.01,****p<*0.001, age2=10-12 years, age3=13-15 years.

| Covarate | tau=0.1 | tau=0.25 | tau=0.5 | tau=0.75 | tau=0.9 |
| --- | --- | --- | --- | --- | --- |
| (Intercept) | 0.154(0.002)*** | 0.285(0.003)*** | 0.5(0.003)*** | 0.753(0.003)*** | 0.897(0.002)*** |
| year1990 | 0(0.003) | 0.004(0.003) | 0.008(0.004) | 0.006(0.003) | 0.002(0.002) |
| year1991 | -0.009(0.003)** | -0.008(0.003)** | -0.001(0.004)** | 0.004(0.003)** | 0.001(0.002)** |
| year1992 | -0.001(0.003) | 0.004(0.003) | 0.016(0.004) | 0.015(0.003) | 0.002(0.002) |
| year1993 | -0.019(0.003)*** | -0.018(0.003)*** | -0.014(0.004)*** | -0.002(0.003)*** | -0.001(0.002)*** |
| year1994 | -0.018(0.003)*** | -0.017(0.003)*** | -0.005(0.004)*** | 0.01(0.003)*** | 0.007(0.002)*** |
| year1995 | -0.022(0.003)*** | -0.024(0.003)*** | -0.014(0.004)*** | -0.002(0.003)*** | -0.001(0.002)*** |
| year1996 | -0.028(0.003)*** | -0.029(0.003)*** | -0.021(0.004)*** | -0.002(0.004)*** | 0(0.002)*** |
| year1997 | -0.03(0.003)*** | -0.028(0.003)*** | -0.013(0.004)*** | 0.007(0.003)*** | 0.003(0.002)*** |
| year1998 | -0.038(0.003)*** | -0.044(0.003)*** | -0.034(0.004)*** | -0.005(0.004)*** | 0.002(0.002)*** |
| year1999 | -0.043(0.003)*** | -0.051(0.003)*** | -0.042(0.004)*** | -0.012(0.004)*** | -0.001(0.002)*** |
| year2000 | -0.053(0.003)*** | -0.06(0.004)*** | -0.049(0.004)*** | -0.014(0.004)*** | -0.003(0.002)*** |
| year2001 | -0.057(0.003)*** | -0.07(0.003)*** | -0.072(0.004)*** | -0.035(0.004)*** | -0.012(0.002)*** |
| year2002 | -0.064(0.003)*** | -0.079(0.003)*** | -0.07(0.004)*** | -0.032(0.004)*** | -0.011(0.002)*** |
| year2003 | -0.066(0.003)*** | -0.081(0.003)*** | -0.076(0.004)*** | -0.04(0.004)*** | -0.014(0.002)*** |
| year2004 | -0.072(0.003)*** | -0.095(0.003)*** | -0.095(0.004)*** | -0.052(0.004)*** | -0.017(0.002)*** |
| year2005 | -0.076(0.003)*** | -0.099(0.003)*** | -0.104(0.004)*** | -0.061(0.004)*** | -0.02(0.002)*** |
| year2006 | -0.085(0.003)*** | -0.114(0.003)*** | -0.129(0.004)*** | -0.076(0.004)*** | -0.027(0.002)*** |
| year2007 | -0.088(0.003)*** | -0.123(0.003)*** | -0.144(0.004)*** | -0.092(0.004)*** | -0.032(0.003)*** |
| year2008 | -0.094(0.003)*** | -0.13(0.003)*** | -0.152(0.004)*** | -0.107(0.004)*** | -0.037(0.002)*** |
| year2009 | -0.09(0.003)*** | -0.121(0.003)*** | -0.142(0.004)*** | -0.096(0.004)*** | -0.035(0.003)*** |
| year2010 | -0.098(0.003)*** | -0.126(0.003)*** | -0.14(0.004)*** | -0.092(0.004)*** | -0.03(0.002)*** |
| year2011 | -0.098(0.003)*** | -0.126(0.003)*** | -0.144(0.004)*** | -0.089(0.004)*** | -0.03(0.002)*** |
| year2012 | -0.099(0.003)*** | -0.126(0.003)*** | -0.137(0.004)*** | -0.077(0.004)*** | -0.023(0.002)*** |
| year2013 | -0.101(0.003)*** | -0.134(0.003)*** | -0.148(0.004)*** | -0.095(0.004)*** | -0.031(0.002)*** |
| year2014 | -0.101(0.003)*** | -0.125(0.003)*** | -0.121(0.004)*** | -0.062(0.004)*** | -0.019(0.002)*** |
| year2015 | -0.099(0.003)*** | -0.12(0.003)*** | -0.12(0.004)*** | -0.061(0.004)*** | -0.016(0.002)*** |
| year2016 | -0.098(0.003)*** | -0.119(0.003)*** | -0.123(0.004)*** | -0.063(0.004)*** | -0.018(0.002)*** |
| year2017 | -0.093(0.003)*** | -0.113(0.003)*** | -0.11(0.004)*** | -0.051(0.004)*** | -0.013(0.002)*** |
| year2018 | -0.101(0.003)*** | -0.122(0.003)*** | -0.123(0.004)*** | -0.066(0.004)*** | -0.021(0.002)*** |
| year2019 | -0.101(0.003)*** | -0.122(0.003)*** | -0.12(0.004)*** | -0.065(0.004)*** | -0.021(0.002)*** |
| age2 | -0.002(0.003) | 0.008(0.004) | 0.015(0.004) | 0.005(0.003) | -0.004(0.002) |
| age3 | -0.03(0.004)*** | -0.018(0.005)*** | -0.003(0.005)*** | -0.024(0.004)*** | -0.006(0.003)*** |
| region1 | 0.029(0.001)*** | 0.047(0.001)*** | 0.05(0.002)*** | 0.026(0.002)*** | 0.008(0.001)*** |
| region2 | 0.028(0.001)*** | 0.049(0.002)*** | 0.059(0.002)*** | 0.036(0.002)*** | 0.017(0.001)*** |
| region3 | 0.029(0.001)*** | 0.05(0.001)*** | 0.064(0.002)*** | 0.044(0.002)*** | 0.021(0.001)*** |
| region4 | -0.006(0.001)*** | -0.007(0.002)*** | -0.005(0.003)*** | 0.001(0.003)*** | -0.004(0.002)*** |
| region5 | 0.02(0.001)*** | 0.049(0.002)*** | 0.077(0.002)*** | 0.059(0.002)*** | 0.029(0.001)*** |
| region6 | 0.025(0.001)*** | 0.052(0.001)*** | 0.081(0.002)*** | 0.063(0.002)*** | 0.032(0.001)*** |
| region7 | 0.026(0.001)*** | 0.048(0.001)*** | 0.061(0.002)*** | 0.04(0.001)*** | 0.014(0.001)*** |
| region8 | 0.05(0.001)*** | 0.089(0.001)*** | 0.116(0.002)*** | 0.079(0.002)*** | 0.041(0.001)*** |
| region9 | 0.027(0.001)*** | 0.057(0.002)*** | 0.081(0.003)*** | 0.055(0.002)*** | 0.023(0.001)*** |
| region10 | 0.029(0.001)*** | 0.053(0.002)*** | 0.073(0.002)*** | 0.052(0.002)*** | 0.025(0.001)*** |
| Covariate | tau=0.1 | tau=0.25 | tau=0.5 | tau=0.75 | tau=0.9 |
| region11 | 0.01(0.001)*** | 0.019(0.002)*** | 0.025(0.002)*** | 0.013(0.002)*** | 0.004(0.001)*** |
| year1990age2 | 0.002(0.004) | -0.002(0.005) | -0.012(0.006) | -0.007(0.004) | -0.005(0.003) |
| year1991age2 | -0.004(0.004) | -0.004(0.005) | -0.015(0.006) | -0.011(0.004) | -0.005(0.003) |
| year1992age2 | 0.001(0.005) | 0.002(0.005) | -0.008(0.006) | -0.004(0.004) | 0.008(0.003) |
| year1993age2 | -0.001(0.004) | -0.003(0.005) | -0.01(0.006) | -0.001(0.004) | 0(0.003) |
| year1994age2 | -0.008(0.004)* | -0.007(0.005)* | -0.012(0.006)* | -0.006(0.004)* | -0.005(0.003)* |
| year1995age2 | -0.003(0.004) | -0.001(0.005) | -0.012(0.006) | 0.002(0.004) | 0.001(0.003) |
| year1996age2 | -0.004(0.004) | -0.001(0.005) | -0.004(0.006) | 0.001(0.004) | 0.001(0.003) |
| year1997age2 | 0(0.004) | 0.002(0.005) | -0.008(0.006) | -0.002(0.004) | 0.001(0.003) |
| year1998age2 | 0.001(0.004) | 0.005(0.005) | 0.004(0.006) | 0.004(0.004) | -0.002(0.003) |
| year1999age2 | 0.001(0.004) | -0.002(0.005) | 0(0.006) | 0.009(0.004) | -0.001(0.003) |
| year2000age2 | -0.003(0.004) | -0.001(0.005) | -0.004(0.006) | 0.004(0.005) | -0.004(0.003) |
| year2001age2 | -0.01(0.004)* | -0.013(0.005)* | -0.008(0.006)* | 0.01(0.005)* | -0.006(0.003)* |
| year2002age2 | -0.002(0.004) | -0.003(0.005) | -0.01(0.006) | 0.009(0.005) | -0.006(0.003) |
| year2003age2 | 0(0.004) | 0.001(0.005) | -0.007(0.006) | 0.012(0.005) | -0.005(0.003) |
| year2004age2 | -0.004(0.004) | -0.002(0.005) | 0.004(0.006) | 0.017(0.005) | -0.006(0.003) |
| year2005age2 | -0.009(0.004)* | -0.01(0.005)* | -0.001(0.006)* | 0.008(0.006)* | -0.007(0.003)* |
| year2006age2 | -0.003(0.004) | -0.003(0.005) | 0.007(0.006) | 0.006(0.006) | -0.004(0.004) |
| year2007age2 | -0.008(0.004) | -0.007(0.005) | 0.006(0.006) | 0.006(0.006) | -0.012(0.004) |
| year2008age2 | -0.01(0.004)* | -0.006(0.005)* | 0.003(0.006)* | 0.011(0.006)* | -0.013(0.004)* |
| year2009age2 | -0.014(0.004)*** | -0.022(0.005)*** | -0.011(0.006)*** | -0.007(0.006)*** | -0.014(0.004)*** |
| year2010age2 | -0.011(0.004)** | -0.023(0.005)** | -0.024(0.006)** | -0.012(0.006)** | -0.02(0.004)** |
| year2011age2 | -0.014(0.004)*** | -0.028(0.005)*** | -0.019(0.006)*** | -0.016(0.006)*** | -0.02(0.004)*** |
| year2012age2 | -0.01(0.004)* | -0.018(0.005)* | -0.014(0.006)* | -0.011(0.006)* | -0.021(0.004)* |
| year2013age2 | -0.009(0.004)* | -0.012(0.005)* | -0.004(0.006)* | 0.007(0.006)* | -0.011(0.004)* |
| year2014age2 | -0.006(0.004) | -0.01(0.005) | -0.009(0.006) | -0.005(0.006) | -0.02(0.003) |
| year2015age2 | -0.007(0.004) | -0.009(0.005) | -0.008(0.006) | -0.005(0.006) | -0.019(0.004) |
| year2016age2 | -0.013(0.004)*** | -0.024(0.005)*** | -0.014(0.006)*** | -0.013(0.006)*** | -0.025(0.003)*** |
| year2017age2 | -0.016(0.004)*** | -0.027(0.005)*** | -0.023(0.006)*** | -0.017(0.006)*** | -0.026(0.003)*** |
| year2018age2 | -0.014(0.004)*** | -0.024(0.005)*** | -0.016(0.006)*** | -0.009(0.006)*** | -0.022(0.003)*** |
| year2019age2 | -0.017(0.004)*** | -0.027(0.005)*** | -0.025(0.006)*** | -0.021(0.006)*** | -0.025(0.003)*** |
| year1990age3 | 0.002(0.005) | 0.01(0.006) | 0.007(0.007) | 0(0.005) | -0.001(0.004) |
| year1991age3 | 0.005(0.005) | 0.016(0.006) | 0.019(0.007) | 0.01(0.005) | 0.007(0.004) |
| year1992age3 | 0.016(0.005)** | 0.023(0.006)** | 0.022(0.007)** | 0.01(0.005)** | 0.014(0.004)** |
| year1993age3 | 0.008(0.005) | 0.017(0.006) | 0.022(0.007) | 0.008(0.005) | 0.004(0.004) |
| year1994age3 | 0.002(0.005) | 0.012(0.006) | 0.009(0.007) | -0.001(0.005) | -0.003(0.004) |
| year1995age3 | 0(0.005) | 0.015(0.006) | 0.014(0.007) | 0.004(0.005) | -0.002(0.004) |
| year1996age3 | 0.001(0.005) | 0.008(0.006) | 0.017(0.007) | 0.008(0.005) | 0.003(0.004) |
| year1997age3 | 0.008(0.005) | 0.018(0.006) | 0.024(0.007) | 0.002(0.005) | 0.001(0.004) |
| year1998age3 | 0.003(0.005) | 0.014(0.006) | 0.025(0.007) | 0.009(0.005) | 0.003(0.004) |
| year1999age3 | 0.004(0.005) | 0.012(0.006) | 0.021(0.007) | 0.008(0.005) | -0.005(0.004) |
| year2000age3 | 0.007(0.005) | 0.014(0.006) | 0.018(0.007) | 0.003(0.005) | -0.01(0.004) |
| year2001age3 | 0.007(0.005) | 0.01(0.006) | 0.021(0.007) | 0.012(0.005) | -0.012(0.004) |
| year2002age3 | 0.015(0.005)** | 0.016(0.006)** | 0.015(0.007)** | 0.007(0.005)** | -0.01(0.004)** |
| year2003age3 | 0.009(0.005) | 0.01(0.006) | 0.018(0.007) | 0.015(0.005) | -0.01(0.004) |
| Covariate | tau=0.1 | tau=0.25 | tau=0.5 | tau=0.75 | tau=0.9 |
| year2004age3 | 0.014(0.005)** | 0.018(0.006)** | 0.022(0.007)** | 0.017(0.005)** | -0.015(0.005)** |
| year2005age3 | 0.012(0.005)* | 0.022(0.006)* | 0.029(0.007)* | 0.028(0.006)* | -0.009(0.005)* |
| year2006age3 | 0.017(0.005)*** | 0.033(0.006)*** | 0.06(0.007)*** | 0.039(0.006)*** | -0.007(0.005)*** |
| year2007age3 | 0.011(0.005)* | 0.024(0.006)* | 0.053(0.007)* | 0.04(0.006)* | -0.012(0.005)* |
| year2008age3 | 0.016(0.005)*** | 0.025(0.006)*** | 0.053(0.007)*** | 0.056(0.006)*** | -0.011(0.005)*** |
| year2009age3 | 0.015(0.005)** | 0.016(0.006)** | 0.045(0.007)** | 0.038(0.006)** | -0.016(0.005)** |
| year2010age3 | 0.019(0.005)*** | 0.021(0.006)*** | 0.042(0.007)*** | 0.041(0.006)*** | -0.014(0.005)*** |
| year2011age3 | 0.015(0.005)** | 0.015(0.006)** | 0.037(0.007)** | 0.039(0.006)** | -0.019(0.005)** |
| year2012age3 | 0.016(0.005)*** | 0.015(0.007)*** | 0.045(0.007)*** | 0.035(0.006)*** | -0.018(0.005)*** |
| year2013age3 | 0.018(0.005)*** | 0.028(0.006)*** | 0.067(0.007)*** | 0.058(0.006)*** | -0.003(0.005)*** |
| year2014age3 | 0.021(0.005)*** | 0.032(0.006)*** | 0.063(0.007)*** | 0.038(0.005)*** | -0.015(0.005)*** |
| year2015age3 | 0.023(0.005)*** | 0.043(0.007)*** | 0.066(0.007)*** | 0.033(0.006)*** | -0.016(0.005)*** |
| year2016age3 | 0.022(0.005)*** | 0.038(0.007)*** | 0.073(0.007)*** | 0.036(0.005)*** | -0.016(0.005)*** |
| year2017age3 | 0.014(0.005)** | 0.027(0.007)** | 0.055(0.007)** | 0.024(0.005)** | -0.024(0.005)** |
| year2018age3 | 0.024(0.005)*** | 0.037(0.007)*** | 0.057(0.007)*** | 0.037(0.005)*** | -0.014(0.005)*** |
| year2019age3 | 0.014(0.005)** | 0.02(0.006)** | 0.034(0.007)** | 0.026(0.006)** | -0.022(0.005)** |

Supplementary Table 3.16: Coefficient estimates (standard errors) for different quantiles (tau) for BAH, girls; **p<*0.05, ***p<*0.01,****p<*0.001, age2=10-12 years, age3=13-15 years.

| Covariate | tau=0.1 | tau=0.25 | tau=0.5 | tau=0.75 | tau=0.9 |
| --- | --- | --- | --- | --- | --- |
| (Intercept) | 0.116(0.002)*** | 0.24(0.003)*** | 0.44(0.003)*** | 0.691(0.003)*** | 0.877(0.002)*** |
| year1990 | 0.004(0.003) | 0.007(0.003) | 0.015(0.004) | 0.011(0.003) | 0.008(0.002) |
| year1991 | 0.002(0.003) | 0.005(0.003) | 0.006(0.004) | 0.008(0.004) | 0.006(0.002) |
| year1992 | 0.015(0.003)*** | 0.016(0.003)*** | 0.019(0.004)*** | 0.016(0.004)*** | 0.007(0.002)*** |
| year1993 | -0.008(0.003)** | -0.011(0.003)** | -0.006(0.004)** | -0.001(0.004)** | 0.002(0.002)** |
| year1994 | -0.009(0.003)** | -0.013(0.004)** | -0.003(0.004)** | 0.009(0.004)** | 0.011(0.002)** |
| year1995 | -0.009(0.003)** | -0.012(0.003)** | -0.005(0.004)** | 0.005(0.004)** | 0.008(0.002)** |
| year1996 | -0.023(0.003)*** | -0.023(0.004)*** | -0.003(0.004)*** | 0.011(0.004)*** | 0.009(0.002)*** |
| year1997 | -0.019(0.003)*** | -0.014(0.004)*** | -0.004(0.004)*** | 0.013(0.004)*** | 0.011(0.002)*** |
| year1998 | -0.03(0.003)*** | -0.035(0.004)*** | -0.025(0.004)*** | -0.006(0.004)*** | 0.005(0.002)*** |
| year1999 | -0.03(0.003)*** | -0.044(0.004)*** | -0.032(0.004)*** | -0.008(0.004)*** | 0.007(0.002)*** |
| year2000 | -0.043(0.003)*** | -0.053(0.003)*** | -0.039(0.004)*** | -0.009(0.004)*** | 0.003(0.002)*** |
| year2001 | -0.04(0.003)*** | -0.052(0.003)*** | -0.039(0.004)*** | -0.005(0.004)*** | 0.006(0.002)*** |
| year2002 | -0.043(0.003)*** | -0.05(0.004)*** | -0.032(0.004)*** | -0.002(0.004)*** | 0.009(0.002)*** |
| year2003 | -0.044(0.003)*** | -0.052(0.003)*** | -0.043(0.004)*** | -0.007(0.004)*** | 0.008(0.002)*** |
| year2004 | -0.051(0.003)*** | -0.067(0.003)*** | -0.054(0.004)*** | -0.018(0.004)*** | 0.004(0.002)*** |
| year2005 | -0.053(0.003)*** | -0.069(0.004)*** | -0.055(0.004)*** | -0.025(0.004)*** | -0.003(0.003)*** |
| year2006 | -0.056(0.003)*** | -0.077(0.003)*** | -0.068(0.004)*** | -0.03(0.004)*** | -0.001(0.003)*** |
| year2007 | -0.063(0.003)*** | -0.086(0.003)*** | -0.081(0.004)*** | -0.045(0.004)*** | -0.011(0.003)*** |
| year2008 | -0.066(0.003)*** | -0.091(0.003)*** | -0.084(0.004)*** | -0.044(0.005)*** | -0.006(0.003)*** |
| year2009 | -0.064(0.003)*** | -0.077(0.004)*** | -0.068(0.004)*** | -0.035(0.004)*** | -0.003(0.002)*** |
| year2010 | -0.067(0.003)*** | -0.082(0.003)*** | -0.071(0.005)*** | -0.037(0.004)*** | -0.003(0.002)*** |
| year2011 | -0.067(0.003)*** | -0.083(0.004)*** | -0.071(0.004)*** | -0.028(0.004)*** | -0.001(0.002)*** |
| year2012 | -0.069(0.003)*** | -0.081(0.004)*** | -0.065(0.005)*** | -0.021(0.004)*** | 0.003(0.002)*** |
| year2013 | -0.071(0.003)*** | -0.095(0.003)*** | -0.082(0.005)*** | -0.033(0.004)*** | 0.001(0.002)*** |
| year2014 | -0.069(0.003)*** | -0.077(0.004)*** | -0.057(0.004)*** | -0.011(0.004)*** | 0.008(0.002)*** |
| year2015 | -0.068(0.003)*** | -0.082(0.004)*** | -0.055(0.005)*** | -0.009(0.004)*** | 0.011(0.002)*** |
| year2016 | -0.07(0.003)*** | -0.082(0.003)*** | -0.063(0.004)*** | -0.023(0.004)*** | 0.006(0.002)*** |
| year2017 | -0.068(0.003)*** | -0.073(0.003)*** | -0.039(0.004)*** | 0.007(0.004)*** | 0.016(0.002)*** |
| year2018 | -0.069(0.003)*** | -0.076(0.003)*** | -0.06(0.004)*** | -0.009(0.004)*** | 0.008(0.002)*** |
| year2019 | -0.067(0.003)*** | -0.074(0.003)*** | -0.048(0.004)*** | -0.008(0.004)*** | 0.011(0.002)*** |
| age2 | -0.013(0.003)*** | -0.031(0.003)*** | -0.032(0.004)*** | -0.025(0.004)*** | -0.016(0.002)*** |
| age3 | -0.033(0.003)*** | -0.062(0.004)*** | -0.058(0.005)*** | -0.056(0.005)*** | -0.03(0.003)*** |
| region1 | 0.032(0.001)*** | 0.048(0.001)*** | 0.059(0.002)*** | 0.037(0.002)*** | 0.011(0.001)*** |
| region2 | 0.032(0.001)*** | 0.045(0.002)*** | 0.055(0.002)*** | 0.038(0.002)*** | 0.014(0.001)*** |
| region3 | 0.032(0.001)*** | 0.053(0.001)*** | 0.069(0.002)*** | 0.055(0.002)*** | 0.019(0.001)*** |
| region4 | 0(0.001) | 0.008(0.002) | 0.025(0.003) | 0.03(0.003) | 0.007(0.001) |
| region5 | 0.026(0.001)*** | 0.064(0.002)*** | 0.105(0.002)*** | 0.094(0.002)*** | 0.033(0.001)*** |
| region6 | 0.024(0.001)*** | 0.053(0.001)*** | 0.088(0.002)*** | 0.081(0.002)*** | 0.029(0.001)*** |
| region7 | 0.034(0.001)*** | 0.065(0.001)*** | 0.093(0.002)*** | 0.079(0.002)*** | 0.028(0.001)*** |
| region8 | 0.068(0.001)*** | 0.121(0.001)*** | 0.16(0.002)*** | 0.13(0.002)*** | 0.045(0.001)*** |
| region9 | 0.034(0.001)*** | 0.074(0.002)*** | 0.113(0.003)*** | 0.096(0.002)*** | 0.033(0.001)*** |
| Covariate | tau=0.1 | tau=0.25 | tau=0.5 | tau=0.75 | tau=0.9 |
| region10 | 0.048(0.001)*** | 0.093(0.002)*** | 0.135(0.002)*** | 0.11(0.002)*** | 0.037(0.001)*** |
| region11 | 0.021(0.001)*** | 0.04(0.002)*** | 0.059(0.002)*** | 0.048(0.002)*** | 0.017(0.001)*** |
| year1990age2 | 0(0.004) | -0.001(0.005) | -0.01(0.006) | -0.01(0.005) | -0.005(0.003) |
| year1991age2 | -0.004(0.004) | -0.005(0.005) | -0.003(0.006) | -0.003(0.005) | 0.001(0.003) |
| year1992age2 | 0.003(0.004) | 0.014(0.005) | 0.021(0.006) | 0.021(0.005) | 0.009(0.003) |
| year1993age2 | 0.011(0.004)** | 0.019(0.005)** | 0.018(0.006)** | 0.019(0.005)** | 0.01(0.003)** |
| year1994age2 | 0.011(0.004)** | 0.023(0.005)** | 0.03(0.006)** | 0.026(0.005)** | 0.006(0.003)** |
| year1995age2 | 0.006(0.004) | 0.021(0.005) | 0.023(0.006) | 0.019(0.005) | 0.005(0.003) |
| year1996age2 | 0.015(0.004)*** | 0.029(0.005)*** | 0.026(0.006)*** | 0.016(0.005)*** | 0.008(0.003)*** |
| year1997age2 | 0.018(0.004)*** | 0.029(0.005)*** | 0.038(0.006)*** | 0.029(0.006)*** | 0.009(0.003)*** |
| year1998age2 | 0.023(0.004)*** | 0.041(0.005)*** | 0.045(0.006)*** | 0.042(0.006)*** | 0.015(0.003)*** |
| year1999age2 | 0.016(0.004)*** | 0.042(0.005)*** | 0.047(0.006)*** | 0.039(0.005)*** | 0.009(0.003)*** |
| year2000age2 | 0.02(0.004)*** | 0.038(0.005)*** | 0.042(0.006)*** | 0.035(0.006)*** | 0.012(0.003)*** |
| year2001age2 | 0.015(0.004)*** | 0.037(0.005)*** | 0.042(0.006)*** | 0.027(0.006)*** | 0.008(0.003)*** |
| year2002age2 | 0.022(0.004)*** | 0.048(0.005)*** | 0.057(0.006)*** | 0.043(0.006)*** | 0.01(0.003)*** |
| year2003age2 | 0.022(0.004)*** | 0.044(0.005)*** | 0.057(0.006)*** | 0.042(0.006)*** | 0.01(0.003)*** |
| year2004age2 | 0.023(0.004)*** | 0.047(0.005)*** | 0.056(0.006)*** | 0.047(0.006)*** | 0.012(0.003)*** |
| year2005age2 | 0.019(0.004)*** | 0.039(0.005)*** | 0.047(0.006)*** | 0.048(0.006)*** | 0.021(0.003)*** |
| year2006age2 | 0.009(0.004)* | 0.031(0.005)* | 0.041(0.007)* | 0.038(0.006)* | 0.015(0.003)* |
| year2007age2 | 0.014(0.004)*** | 0.037(0.005)*** | 0.043(0.007)*** | 0.039(0.006)*** | 0.017(0.003)*** |
| year2008age2 | 0.012(0.004)** | 0.037(0.005)** | 0.045(0.006)** | 0.042(0.006)** | 0.011(0.003)** |
| year2009age2 | 0.01(0.004)** | 0.024(0.005)** | 0.031(0.006)** | 0.03(0.006)** | 0.009(0.003)** |
| year2010age2 | 0.011(0.004)** | 0.022(0.005)** | 0.035(0.007)** | 0.038(0.006)** | 0.013(0.003)** |
| year2011age2 | 0.008(0.004)* | 0.021(0.005)* | 0.029(0.007)* | 0.031(0.006)* | 0.012(0.003)* |
| year2012age2 | 0.013(0.004)*** | 0.028(0.005)*** | 0.035(0.007)*** | 0.034(0.006)*** | 0.01(0.003)*** |
| year2013age2 | 0.017(0.004)*** | 0.045(0.005)*** | 0.061(0.007)*** | 0.056(0.006)*** | 0.016(0.003)*** |
| year2014age2 | 0.014(0.004)*** | 0.035(0.005)*** | 0.05(0.007)*** | 0.042(0.006)*** | 0.014(0.003)*** |
| year2015age2 | 0.014(0.004)*** | 0.04(0.005)*** | 0.052(0.007)*** | 0.046(0.006)*** | 0.011(0.003)*** |
| year2016age2 | 0.012(0.004)** | 0.032(0.005)** | 0.045(0.006)** | 0.044(0.006)** | 0.01(0.003)** |
| year2017age2 | 0.014(0.004)*** | 0.036(0.005)*** | 0.044(0.006)*** | 0.034(0.006)*** | 0.004(0.003)*** |
| year2018age2 | 0.013(0.004)** | 0.036(0.005)** | 0.064(0.006)** | 0.047(0.006)** | 0.01(0.003)** |
| year2019age2 | 0.01(0.004)* | 0.032(0.005)* | 0.051(0.006)* | 0.049(0.006)* | 0.009(0.003)* |
| year1990age3 | -0.002(0.004) | 0.002(0.005) | 0.001(0.007) | 0.008(0.007) | -0.001(0.004) |
| year1991age3 | 0.001(0.004) | 0.009(0.006) | 0.014(0.007) | 0.018(0.007) | 0.003(0.004) |
| year1992age3 | 0.003(0.004) | 0.03(0.005) | 0.035(0.007) | 0.042(0.006) | 0.015(0.003) |
| year1993age3 | 0.016(0.004)*** | 0.038(0.006)*** | 0.04(0.007)*** | 0.041(0.006)*** | 0.011(0.003)*** |
| year1994age3 | 0.011(0.004)** | 0.038(0.006)** | 0.044(0.007)** | 0.042(0.006)** | 0.01(0.003)** |
| year1995age3 | 0.013(0.004)*** | 0.041(0.006)*** | 0.051(0.007)*** | 0.049(0.006)*** | 0.013(0.003)*** |
| year1996age3 | 0.027(0.004)*** | 0.059(0.006)*** | 0.054(0.007)*** | 0.053(0.006)*** | 0.016(0.003)*** |
| year1997age3 | 0.027(0.004)*** | 0.042(0.006)*** | 0.054(0.007)*** | 0.05(0.007)*** | 0.011(0.003)*** |
| year1998age3 | 0.03(0.004)*** | 0.053(0.006)*** | 0.073(0.007)*** | 0.07(0.007)*** | 0.019(0.004)*** |
| year1999age3 | 0.031(0.004)*** | 0.066(0.006)*** | 0.079(0.007)*** | 0.072(0.007)*** | 0.019(0.004)*** |
| year2000age3 | 0.042(0.004)*** | 0.078(0.006)*** | 0.089(0.007)*** | 0.08(0.007)*** | 0.027(0.004)*** |
| year2001age3 | 0.036(0.004)*** | 0.074(0.006)*** | 0.093(0.007)*** | 0.082(0.007)*** | 0.025(0.003)*** |
| year2002age3 | 0.035(0.004)*** | 0.068(0.006)*** | 0.079(0.007)*** | 0.079(0.007)*** | 0.021(0.003)*** |
| Covariate | tau=0.1 | tau=0.25 | tau=0.5 | tau=0.75 | tau=0.9 |
| year2003age3 | 0.034(0.004)*** | 0.06(0.006)*** | 0.086(0.007)*** | 0.077(0.007)*** | 0.021(0.004)*** |
| year2004age3 | 0.04(0.004)*** | 0.076(0.006)*** | 0.091(0.007)*** | 0.085(0.007)*** | 0.022(0.004)*** |
| year2005age3 | 0.039(0.004)*** | 0.07(0.006)*** | 0.091(0.007)*** | 0.088(0.007)*** | 0.026(0.004)*** |
| year2006age3 | 0.036(0.004)*** | 0.081(0.006)*** | 0.102(0.007)*** | 0.089(0.007)*** | 0.026(0.004)*** |
| year2007age3 | 0.039(0.004)*** | 0.072(0.006)*** | 0.092(0.007)*** | 0.08(0.008)*** | 0.027(0.004)*** |
| year2008age3 | 0.036(0.004)*** | 0.075(0.006)*** | 0.095(0.008)*** | 0.089(0.007)*** | 0.023(0.004)*** |
| year2009age3 | 0.034(0.004)*** | 0.059(0.006)*** | 0.071(0.008)*** | 0.069(0.007)*** | 0.019(0.004)*** |
| year2010age3 | 0.037(0.004)*** | 0.064(0.006)*** | 0.085(0.008)*** | 0.073(0.007)*** | 0.019(0.004)*** |
| year2011age3 | 0.023(0.004)*** | 0.054(0.006)*** | 0.081(0.008)*** | 0.072(0.007)*** | 0.019(0.004)*** |
| year2012age3 | 0.03(0.004)*** | 0.059(0.006)*** | 0.093(0.008)*** | 0.079(0.007)*** | 0.018(0.004)*** |
| year2013age3 | 0.037(0.004)*** | 0.078(0.006)*** | 0.11(0.008)*** | 0.092(0.008)*** | 0.023(0.004)*** |
| year2014age3 | 0.035(0.004)*** | 0.07(0.006)*** | 0.104(0.008)*** | 0.091(0.007)*** | 0.023(0.004)*** |
| year2015age3 | 0.038(0.004)*** | 0.088(0.006)*** | 0.113(0.008)*** | 0.096(0.007)*** | 0.023(0.004)*** |
| year2016age3 | 0.04(0.004)*** | 0.087(0.006)*** | 0.123(0.008)*** | 0.112(0.007)*** | 0.031(0.004)*** |
| year2017age3 | 0.039(0.004)*** | 0.086(0.006)*** | 0.096(0.007)*** | 0.08(0.007)*** | 0.019(0.003)*** |
| year2018age3 | 0.039(0.005)*** | 0.091(0.007)*** | 0.112(0.008)*** | 0.095(0.007)*** | 0.023(0.004)*** |
| year2019age3 | 0.033(0.005)*** | 0.079(0.006)*** | 0.112(0.008)*** | 0.103(0.007)*** | 0.024(0.004)*** |

Supplementary Table 3.17: Coefficient estimates (standard errors) for different quantiles (tau) for D60, boys; **p<*0.05, ***p<*0.01,****p<*0.001, age2=10-12 years, age3=13-15 years.

| Covariate | tau=0.1 | tau=0.25 | tau=0.5 | tau=0.75 | tau=0.9 |
| --- | --- | --- | --- | --- | --- |
| (Intercept) | 0.103(0.002)*** | 0.249(0.003)*** | 0.488(0.003)*** | 0.737(0.003)*** | 0.888(0.002)*** |
| year1990 | 0.003(0.002) | 0.005(0.003) | 0.007(0.004) | 0.007(0.004) | -0.001(0.003) |
| year1991 | 0.003(0.002) | 0.011(0.003) | 0.016(0.004) | 0.016(0.004) | 0.008(0.003) |
| year1992 | 0.009(0.002)*** | 0.018(0.003)*** | 0.028(0.004)*** | 0.02(0.004)*** | 0.012(0.003)*** |
| year1993 | 0.023(0.002)*** | 0.04(0.003)*** | 0.051(0.004)*** | 0.034(0.004)*** | 0.015(0.003)*** |
| year1994 | 0.019(0.002)*** | 0.031(0.003)*** | 0.039(0.004)*** | 0.028(0.004)*** | 0.012(0.003)*** |
| year1995 | 0.029(0.003)*** | 0.045(0.003)*** | 0.061(0.004)*** | 0.047(0.004)*** | 0.025(0.003)*** |
| year1996 | 0.018(0.002)*** | 0.031(0.003)*** | 0.049(0.004)*** | 0.039(0.004)*** | 0.022(0.003)*** |
| year1997 | 0.026(0.003)*** | 0.044(0.003)*** | 0.057(0.004)*** | 0.047(0.004)*** | 0.025(0.003)*** |
| year1998 | 0.022(0.003)*** | 0.036(0.004)*** | 0.053(0.004)*** | 0.043(0.004)*** | 0.024(0.003)*** |
| year1999 | 0.021(0.003)*** | 0.038(0.004)*** | 0.059(0.004)*** | 0.047(0.004)*** | 0.028(0.003)*** |
| year2000 | 0.021(0.003)*** | 0.038(0.004)*** | 0.061(0.004)*** | 0.051(0.004)*** | 0.031(0.003)*** |
| year2001 | 0.02(0.003)*** | 0.037(0.004)*** | 0.058(0.004)*** | 0.056(0.004)*** | 0.034(0.003)*** |
| year2002 | 0.01(0.003)*** | 0.02(0.004)*** | 0.043(0.004)*** | 0.045(0.004)*** | 0.028(0.003)*** |
| year2003 | 0.023(0.003)*** | 0.041(0.004)*** | 0.067(0.004)*** | 0.06(0.004)*** | 0.036(0.003)*** |
| year2004 | 0.016(0.003)*** | 0.035(0.004)*** | 0.066(0.004)*** | 0.059(0.004)*** | 0.04(0.003)*** |
| year2005 | 0.008(0.003)** | 0.023(0.004)** | 0.056(0.004)** | 0.062(0.004)** | 0.042(0.003)** |
| year2006 | 0.004(0.003) | 0.016(0.004) | 0.054(0.005) | 0.062(0.004) | 0.042(0.003) |
| year2007 | -0.001(0.003) | 0.013(0.004) | 0.047(0.004) | 0.056(0.004) | 0.04(0.003) |
| year2008 | -0.003(0.003) | 0.007(0.004) | 0.046(0.005) | 0.062(0.004) | 0.042(0.003) |
| year2009 | -0.005(0.003) | 0.013(0.004) | 0.045(0.004) | 0.056(0.004) | 0.042(0.003) |
| year2010 | -0.003(0.003) | 0.011(0.004) | 0.044(0.004) | 0.056(0.004) | 0.043(0.003) |
| year2011 | 0.001(0.003) | 0.016(0.004) | 0.053(0.005) | 0.068(0.004) | 0.047(0.003) |
| year2012 | -0.011(0.002)*** | -0.009(0.004)*** | 0.024(0.004)*** | 0.045(0.004)*** | 0.036(0.003)*** |
| year2013 | -0.013(0.003)*** | -0.003(0.004)*** | 0.037(0.004)*** | 0.057(0.004)*** | 0.043(0.003)*** |
| year2014 | -0.012(0.002)*** | -0.008(0.004)*** | 0.025(0.004)*** | 0.047(0.004)*** | 0.043(0.003)*** |
| year2015 | -0.021(0.002)*** | -0.026(0.003)*** | 0.008(0.004)*** | 0.037(0.004)*** | 0.036(0.003)*** |
| year2016 | -0.024(0.002)*** | -0.032(0.003)*** | -0.001(0.004)*** | 0.027(0.004)*** | 0.033(0.003)*** |
| year2017 | -0.031(0.002)*** | -0.043(0.003)*** | -0.017(0.004)*** | 0.021(0.004)*** | 0.032(0.003)*** |
| year2018 | -0.033(0.002)*** | -0.047(0.003)*** | -0.022(0.004)*** | 0.017(0.004)*** | 0.031(0.003)*** |
| year2019 | -0.032(0.002)*** | -0.047(0.003)*** | -0.021(0.004)*** | 0.015(0.004)*** | 0.028(0.003)*** |
| age2 | 0.024(0.003)*** | 0.033(0.003)*** | 0.03(0.004)*** | 0.012(0.004)*** | 0(0.003)*** |
| age3 | 0.051(0.004)*** | 0.082(0.005)*** | 0.097(0.005)*** | 0.063(0.004)*** | 0.025(0.003)*** |
| region1 | -0.014(0.001)*** | -0.024(0.001)*** | -0.032(0.002)*** | -0.028(0.001)*** | -0.017(0.001)*** |
| region2 | -0.005(0.001)*** | -0.011(0.002)*** | -0.025(0.002)*** | -0.029(0.002)*** | -0.02(0.001)*** |
| region3 | 0.004(0.001)*** | 0.006(0.001)*** | -0.002(0.002)*** | -0.01(0.001)*** | -0.009(0.001)*** |
| region4 | 0.016(0.002)*** | 0.026(0.002)*** | 0.023(0.003)*** | 0.012(0.002)*** | 0.005(0.001)*** |
| region5 | 0.005(0.001)*** | 0.003(0.002)*** | -0.01(0.002)*** | -0.021(0.002)*** | -0.017(0.001)*** |
| region6 | -0.009(0.001)*** | -0.019(0.002)*** | -0.034(0.002)*** | -0.039(0.002)*** | -0.025(0.001)*** |
| region7 | 0.002(0.001) | -0.003(0.001) | -0.019(0.002) | -0.027(0.001) | -0.018(0.001) |
| Covariate | tau=0.1 | tau=0.25 | tau=0.5 | tau=0.75 | tau=0.9 |
| region8 | 0.001(0.001) | -0.006(0.001) | -0.029(0.002) | -0.038(0.001) | -0.026(0.001) |
| region9 | -0.002(0.001) | -0.013(0.002) | -0.037(0.003) | -0.04(0.002) | -0.025(0.002) |
| region10 | -0.022(0.001)*** | -0.051(0.002)*** | -0.081(0.002)*** | -0.076(0.002)*** | -0.045(0.001)*** |
| region11 | -0.002(0.001)* | -0.013(0.002)* | -0.029(0.002)* | -0.033(0.002)* | -0.022(0.001)* |
| year1990age2 | -0.012(0.004)*** | -0.02(0.005)*** | -0.009(0.005)*** | -0.008(0.005)*** | 0.002(0.004)*** |
| year1991age2 | -0.011(0.004)** | -0.021(0.005)** | -0.016(0.005)** | -0.011(0.005)** | -0.003(0.004)** |
| year1992age2 | -0.018(0.004)*** | -0.029(0.005)*** | -0.03(0.005)*** | -0.021(0.005)*** | -0.012(0.004)*** |
| year1993age2 | -0.018(0.004)*** | -0.026(0.005)*** | -0.03(0.005)*** | -0.012(0.005)*** | -0.003(0.004)*** |
| year1994age2 | -0.017(0.004)*** | -0.026(0.005)*** | -0.021(0.005)*** | -0.007(0.005)*** | -0.002(0.004)*** |
| year1995age2 | -0.031(0.004)*** | -0.041(0.005)*** | -0.044(0.005)*** | -0.024(0.005)*** | -0.01(0.004)*** |
| year1996age2 | -0.023(0.004)*** | -0.033(0.005)*** | -0.036(0.006)*** | -0.015(0.005)*** | -0.004(0.004)*** |
| year1997age2 | -0.03(0.004)*** | -0.043(0.005)*** | -0.04(0.006)*** | -0.021(0.005)*** | -0.004(0.004)*** |
| year1998age2 | -0.024(0.004)*** | -0.034(0.005)*** | -0.035(0.006)*** | -0.014(0.005)*** | -0.001(0.004)*** |
| year1999age2 | -0.027(0.004)*** | -0.038(0.005)*** | -0.042(0.006)*** | -0.019(0.005)*** | -0.003(0.004)*** |
| year2000age2 | -0.029(0.004)*** | -0.041(0.005)*** | -0.038(0.006)*** | -0.015(0.005)*** | -0.001(0.004)*** |
| year2001age2 | -0.026(0.004)*** | -0.037(0.005)*** | -0.038(0.006)*** | -0.018(0.005)*** | -0.004(0.004)*** |
| year2002age2 | -0.022(0.004)*** | -0.028(0.005)*** | -0.026(0.006)*** | -0.011(0.005)*** | 0(0.004)*** |
| year2003age2 | -0.041(0.004)*** | -0.051(0.005)*** | -0.048(0.006)*** | -0.018(0.005)*** | -0.004(0.004)*** |
| year2004age2 | -0.04(0.004)*** | -0.059(0.005)*** | -0.056(0.006)*** | -0.025(0.005)*** | -0.009(0.004)*** |
| year2005age2 | -0.038(0.004)*** | -0.056(0.005)*** | -0.059(0.006)*** | -0.029(0.005)*** | -0.009(0.004)*** |
| year2006age2 | -0.037(0.004)*** | -0.052(0.005)*** | -0.055(0.006)*** | -0.022(0.005)*** | -0.006(0.004)*** |
| year2007age2 | -0.032(0.004)*** | -0.047(0.005)*** | -0.044(0.006)*** | -0.019(0.006)*** | -0.002(0.004)*** |
| year2008age2 | -0.033(0.004)*** | -0.043(0.005)*** | -0.047(0.007)*** | -0.021(0.006)*** | -0.004(0.004)*** |
| year2009age2 | -0.032(0.004)*** | -0.049(0.005)*** | -0.041(0.006)*** | -0.011(0.006)*** | 0(0.004)*** |
| year2010age2 | -0.031(0.004)*** | -0.036(0.005)*** | -0.025(0.006)*** | -0.002(0.005)*** | 0.006(0.004)*** |
| year2011age2 | -0.035(0.004)*** | -0.041(0.005)*** | -0.034(0.007)*** | -0.011(0.005)*** | 0.002(0.004)*** |
| year2012age2 | -0.03(0.004)*** | -0.034(0.005)*** | -0.022(0.006)*** | 0.002(0.006)*** | 0.01(0.004)*** |
| year2013age2 | -0.036(0.004)*** | -0.047(0.005)*** | -0.045(0.006)*** | -0.015(0.006)*** | 0.003(0.004)*** |
| year2014age2 | -0.034(0.004)*** | -0.046(0.005)*** | -0.037(0.006)*** | -0.004(0.006)*** | 0(0.004)*** |
| year2015age2 | -0.029(0.003)*** | -0.035(0.005)*** | -0.024(0.006)*** | -0.003(0.006)*** | 0.007(0.004)*** |
| year2016age2 | -0.025(0.003)*** | -0.03(0.005)*** | -0.017(0.006)*** | 0.014(0.006)*** | 0.009(0.004)*** |
| year2017age2 | -0.023(0.003)*** | -0.025(0.005)*** | -0.009(0.006)*** | 0.014(0.006)*** | 0.011(0.004)*** |
| year2018age2 | -0.021(0.003)*** | -0.026(0.005)*** | -0.015(0.006)*** | 0.01(0.006)*** | 0.014(0.004)*** |
| year2019age2 | -0.025(0.003)*** | -0.024(0.005)*** | -0.014(0.006)*** | 0.015(0.006)*** | 0.017(0.004)*** |
| year1990age3 | -0.013(0.005)** | -0.015(0.006)** | -0.021(0.006)** | -0.015(0.005)** | -0.003(0.004)** |
| year1991age3 | -0.025(0.005)*** | -0.043(0.006)*** | -0.045(0.006)*** | -0.034(0.006)*** | -0.014(0.004)*** |
| year1992age3 | -0.033(0.005)*** | -0.053(0.006)*** | -0.062(0.006)*** | -0.043(0.005)*** | -0.024(0.004)*** |
| year1993age3 | -0.04(0.005)*** | -0.066(0.006)*** | -0.071(0.006)*** | -0.046(0.005)*** | -0.018(0.004)*** |
| year1994age3 | -0.043(0.005)*** | -0.063(0.006)*** | -0.069(0.006)*** | -0.047(0.005)*** | -0.019(0.004)*** |
| year1995age3 | -0.051(0.005)*** | -0.08(0.006)*** | -0.093(0.006)*** | -0.064(0.005)*** | -0.025(0.004)*** |
| year1996age3 | -0.047(0.005)*** | -0.072(0.006)*** | -0.093(0.006)*** | -0.058(0.005)*** | -0.026(0.004)*** |
| year1997age3 | -0.057(0.005)*** | -0.081(0.006)*** | -0.096(0.006)*** | -0.07(0.006)*** | -0.026(0.004)*** |
| year1998age3 | -0.054(0.005)*** | -0.079(0.006)*** | -0.09(0.006)*** | -0.062(0.005)*** | -0.023(0.004)*** |
| year1999age3 | -0.044(0.005)*** | -0.076(0.006)*** | -0.089(0.006)*** | -0.056(0.006)*** | -0.023(0.004)*** |
| year2000age3 | -0.057(0.005)*** | -0.088(0.006)*** | -0.103(0.007)*** | -0.063(0.006)*** | -0.022(0.004)*** |
| Covariate | tau=0.1 | tau=0.25 | tau=0.5 | tau=0.75 | tau=0.9 |
| year2001age3 | -0.059(0.005)*** | -0.088(0.006)*** | -0.103(0.006)*** | -0.065(0.006)*** | -0.026(0.004)*** |
| year2002age3 | -0.051(0.005)*** | -0.078(0.006)*** | -0.096(0.007)*** | -0.062(0.006)*** | -0.022(0.004)*** |
| year2003age3 | -0.065(0.005)*** | -0.101(0.006)*** | -0.115(0.007)*** | -0.077(0.006)*** | -0.025(0.004)*** |
| year2004age3 | -0.076(0.005)*** | -0.119(0.006)*** | -0.139(0.007)*** | -0.082(0.006)*** | -0.033(0.004)*** |
| year2005age3 | -0.068(0.005)*** | -0.107(0.006)*** | -0.13(0.007)*** | -0.087(0.006)*** | -0.037(0.004)*** |
| year2006age3 | -0.068(0.005)*** | -0.106(0.006)*** | -0.137(0.007)*** | -0.09(0.006)*** | -0.04(0.004)*** |
| year2007age3 | -0.063(0.005)*** | -0.108(0.007)*** | -0.129(0.007)*** | -0.084(0.006)*** | -0.036(0.005)*** |
| year2008age3 | -0.065(0.005)*** | -0.109(0.006)*** | -0.133(0.007)*** | -0.095(0.006)*** | -0.033(0.004)*** |
| year2009age3 | -0.067(0.005)*** | -0.126(0.006)*** | -0.141(0.007)*** | -0.09(0.006)*** | -0.034(0.005)*** |
| year2010age3 | -0.066(0.005)*** | -0.113(0.006)*** | -0.134(0.007)*** | -0.083(0.006)*** | -0.033(0.004)*** |
| year2011age3 | -0.069(0.005)*** | -0.118(0.007)*** | -0.134(0.007)*** | -0.087(0.006)*** | -0.036(0.005)*** |
| year2012age3 | -0.064(0.004)*** | -0.098(0.007)*** | -0.117(0.007)*** | -0.068(0.006)*** | -0.022(0.004)*** |
| year2013age3 | -0.069(0.005)*** | -0.116(0.007)*** | -0.142(0.007)*** | -0.085(0.007)*** | -0.03(0.004)*** |
| year2014age3 | -0.069(0.004)*** | -0.112(0.006)*** | -0.127(0.007)*** | -0.075(0.006)*** | -0.03(0.004)*** |
| year2015age3 | -0.062(0.004)*** | -0.105(0.006)*** | -0.132(0.007)*** | -0.09(0.007)*** | -0.031(0.005)*** |
| year2016age3 | -0.063(0.004)*** | -0.107(0.006)*** | -0.134(0.007)*** | -0.076(0.007)*** | -0.03(0.005)*** |
| year2017age3 | -0.054(0.004)*** | -0.093(0.006)*** | -0.108(0.007)*** | -0.067(0.007)*** | -0.025(0.005)*** |
| year2018age3 | -0.053(0.004)*** | -0.084(0.006)*** | -0.105(0.007)*** | -0.069(0.007)*** | -0.026(0.005)*** |
| year2019age3 | -0.054(0.004)*** | -0.085(0.006)*** | -0.1(0.007)*** | -0.056(0.007)*** | -0.016(0.005)*** |

Supplementary Table 3.18: Coefficient estimates (standard errors) for different quantiles (tau) for D60, girls; **p<*0.05, ***p<*0.01,****p<*0.001, age2=10-12 years, age3=13-15 years.

| Covariate | tau=0.1 | tau=0.25 | tau=0.5 | tau=0.75 | tau=0.9 |
| --- | --- | --- | --- | --- | --- |
| (Intercept) | 0.127(0.002)*** | 0.293(0.003)*** | 0.552(0.003)*** | 0.782(0.003)*** | 0.918(0.002)*** |
| year1990 | 0.001(0.003) | -0.001(0.004) | -0.008(0.004) | -0.005(0.004) | -0.004(0.003) |
| year1991 | 0.008(0.003)** | 0.01(0.004)** | 0.01(0.004)** | 0.007(0.004)** | 0.003(0.003)** |
| year1992 | 0.011(0.003)*** | 0.018(0.004)*** | 0.01(0.004)*** | 0.007(0.004)*** | 0.002(0.003)*** |
| year1993 | 0.027(0.003)*** | 0.041(0.004)*** | 0.034(0.004)*** | 0.023(0.004)*** | 0.005(0.002)*** |
| year1994 | 0.015(0.003)*** | 0.021(0.004)*** | 0.013(0.004)*** | 0.006(0.004)*** | 0(0.003)*** |
| year1995 | 0.025(0.003)*** | 0.041(0.004)*** | 0.044(0.004)*** | 0.031(0.004)*** | 0.015(0.002)*** |
| year1996 | 0.017(0.003)*** | 0.022(0.004)*** | 0.022(0.004)*** | 0.018(0.004)*** | 0.007(0.002)*** |
| year1997 | 0.023(0.003)*** | 0.035(0.004)*** | 0.036(0.004)*** | 0.024(0.004)*** | 0.01(0.002)*** |
| year1998 | 0.023(0.003)*** | 0.04(0.004)*** | 0.041(0.004)*** | 0.027(0.004)*** | 0.013(0.003)*** |
| year1999 | 0.032(0.003)*** | 0.049(0.004)*** | 0.047(0.004)*** | 0.036(0.004)*** | 0.016(0.002)*** |
| year2000 | 0.027(0.003)*** | 0.054(0.004)*** | 0.058(0.004)*** | 0.046(0.004)*** | 0.023(0.002)*** |
| year2001 | 0.028(0.003)*** | 0.05(0.004)*** | 0.054(0.004)*** | 0.046(0.004)*** | 0.023(0.003)*** |
| year2002 | 0.015(0.003)*** | 0.033(0.004)*** | 0.031(0.004)*** | 0.027(0.004)*** | 0.015(0.003)*** |
| year2003 | 0.027(0.003)*** | 0.048(0.004)*** | 0.058(0.004)*** | 0.047(0.004)*** | 0.025(0.003)*** |
| year2004 | 0.017(0.003)*** | 0.039(0.004)*** | 0.047(0.004)*** | 0.041(0.004)*** | 0.023(0.002)*** |
| year2005 | 0(0.003) | 0.013(0.004) | 0.021(0.004) | 0.03(0.004) | 0.019(0.003) |
| year2006 | -0.005(0.003) | 0.004(0.004) | 0.012(0.005) | 0.016(0.004) | 0.01(0.003) |
| year2007 | -0.009(0.003)*** | -0.004(0.004)*** | 0.006(0.005)*** | 0.018(0.004)*** | 0.01(0.003)*** |
| year2008 | -0.011(0.003)*** | -0.01(0.004)*** | 0.005(0.005)*** | 0.013(0.004)*** | 0.012(0.003)*** |
| year2009 | -0.012(0.003)*** | -0.008(0.004)*** | 0.005(0.005)*** | 0.015(0.004)*** | 0.012(0.003)*** |
| year2010 | -0.014(0.003)*** | -0.008(0.004)*** | -0.001(0.005)*** | 0.012(0.004)*** | 0.013(0.003)*** |
| year2011 | -0.007(0.003)** | -0.001(0.004)** | 0.006(0.005)** | 0.023(0.004)** | 0.015(0.003)** |
| year2012 | -0.02(0.003)*** | -0.025(0.004)*** | -0.021(0.005)*** | -0.002(0.004)*** | 0.005(0.003)*** |
| year2013 | -0.017(0.003)*** | -0.021(0.004)*** | -0.008(0.005)*** | 0.015(0.004)*** | 0.014(0.003)*** |
| year2014 | -0.022(0.002)*** | -0.03(0.004)*** | -0.024(0.004)*** | -0.002(0.004)*** | 0.008(0.003)*** |
| year2015 | -0.028(0.002)*** | -0.037(0.004)*** | -0.03(0.004)*** | -0.006(0.004)*** | 0.002(0.003)*** |
| year2016 | -0.028(0.002)*** | -0.039(0.004)*** | -0.039(0.005)*** | -0.009(0.004)*** | 0.003(0.003)*** |
| year2017 | -0.035(0.002)*** | -0.053(0.003)*** | -0.058(0.004)*** | -0.024(0.004)*** | -0.005(0.003)*** |
| year2018 | -0.038(0.002)*** | -0.06(0.004)*** | -0.058(0.004)*** | -0.025(0.004)*** | -0.005(0.003)*** |
| year2019 | -0.037(0.002)*** | -0.056(0.003)*** | -0.059(0.004)*** | -0.026(0.004)*** | -0.003(0.003)*** |
| age2 | 0.015(0.003)*** | 0.019(0.004)*** | 0.008(0.004)*** | 0.001(0.004)*** | -0.005(0.003)*** |
| age3 | 0.031(0.004)*** | 0.055(0.005)*** | 0.04(0.005)*** | 0.026(0.004)*** | 0(0.003)*** |
| region1 | -0.021(0.001)*** | -0.04(0.002)*** | -0.048(0.002)*** | -0.036(0.001)*** | -0.02(0.001)*** |
| region2 | -0.025(0.001)*** | -0.049(0.002)*** | -0.073(0.002)*** | -0.059(0.002)*** | -0.035(0.001)*** |
| region3 | -0.014(0.001)*** | -0.024(0.002)*** | -0.036(0.002)*** | -0.031(0.001)*** | -0.02(0.001)*** |
| region4 | 0.012(0.002)*** | 0.017(0.003)*** | 0.014(0.003)*** | 0.004(0.002)*** | -0.001(0.001)*** |
| region5 | -0.02(0.001)*** | -0.041(0.002)*** | -0.056(0.002)*** | -0.046(0.002)*** | -0.028(0.001)*** |
| region6 | -0.029(0.001)*** | -0.055(0.002)*** | -0.072(0.002)*** | -0.057(0.002)*** | -0.031(0.001)*** |
| region7 | -0.026(0.001)*** | -0.049(0.001)*** | -0.065(0.002)*** | -0.052(0.001)*** | -0.028(0.001)*** |
| region8 | -0.027(0.001)*** | -0.056(0.002)*** | -0.082(0.002)*** | -0.071(0.001)*** | -0.041(0.001)*** |
| region9 | -0.027(0.002)*** | -0.053(0.002)*** | -0.07(0.003)*** | -0.062(0.002)*** | -0.038(0.002)*** |
| Covariate | tau=0.1 | tau=0.25 | tau=0.5 | tau=0.75 | tau=0.9 |
| region10 | -0.052(0.001)*** | -0.106(0.002)*** | -0.145(0.002)*** | -0.124(0.002)*** | -0.068(0.001)*** |
| region11 | -0.024(0.001)*** | -0.049(0.002)*** | -0.068(0.002)*** | -0.058(0.002)*** | -0.034(0.001)*** |
| year1990age2 | -0.002(0.004) | 0.002(0.005) | 0.014(0.006) | 0.012(0.005) | 0.007(0.004) |
| year1991age2 | -0.01(0.004)* | -0.009(0.005)* | -0.005(0.006)* | 0.002(0.005)* | 0.001(0.004)* |
| year1992age2 | -0.019(0.004)*** | -0.025(0.005)*** | -0.016(0.006)*** | -0.011(0.005)*** | -0.005(0.004)*** |
| year1993age2 | -0.02(0.004)*** | -0.027(0.005)*** | -0.014(0.006)*** | -0.009(0.005)*** | 0.001(0.004)*** |
| year1994age2 | -0.017(0.004)*** | -0.024(0.005)*** | -0.016(0.006)*** | -0.007(0.005)*** | -0.001(0.004)*** |
| year1995age2 | -0.023(0.004)*** | -0.033(0.005)*** | -0.027(0.006)*** | -0.015(0.005)*** | -0.004(0.004)*** |
| year1996age2 | -0.02(0.004)*** | -0.022(0.005)*** | -0.02(0.006)*** | -0.009(0.005)*** | 0(0.004)*** |
| year1997age2 | -0.025(0.004)*** | -0.031(0.005)*** | -0.025(0.006)*** | -0.008(0.005)*** | -0.002(0.004)*** |
| year1998age2 | -0.024(0.004)*** | -0.033(0.005)*** | -0.021(0.006)*** | -0.006(0.005)*** | 0(0.004)*** |
| year1999age2 | -0.027(0.004)*** | -0.038(0.005)*** | -0.021(0.006)*** | -0.008(0.005)*** | 0.001(0.004)*** |
| year2000age2 | -0.024(0.004)*** | -0.034(0.005)*** | -0.017(0.006)*** | -0.004(0.005)*** | 0.002(0.004)*** |
| year2001age2 | -0.03(0.004)*** | -0.034(0.006)*** | -0.017(0.006)*** | -0.007(0.005)*** | 0.003(0.004)*** |
| year2002age2 | -0.021(0.004)*** | -0.029(0.005)*** | -0.007(0.006)*** | 0.004(0.005)*** | 0.005(0.004)*** |
| year2003age2 | -0.031(0.004)*** | -0.039(0.006)*** | -0.03(0.006)*** | -0.014(0.005)*** | -0.001(0.004)*** |
| year2004age2 | -0.029(0.004)*** | -0.044(0.006)*** | -0.031(0.006)*** | -0.014(0.005)*** | -0.003(0.004)*** |
| year2005age2 | -0.021(0.004)*** | -0.031(0.005)*** | -0.022(0.006)*** | -0.01(0.005)*** | -0.002(0.004)*** |
| year2006age2 | -0.018(0.004)*** | -0.027(0.005)*** | -0.012(0.007)*** | 0.007(0.005)*** | 0.008(0.004)*** |
| year2007age2 | -0.015(0.004)*** | -0.019(0.006)*** | -0.006(0.007)*** | 0.006(0.005)*** | 0.011(0.004)*** |
| year2008age2 | -0.017(0.004)*** | -0.018(0.006)*** | -0.006(0.007)*** | 0.009(0.006)*** | 0.007(0.004)*** |
| year2009age2 | -0.013(0.004)** | -0.022(0.006)** | -0.009(0.007)** | 0.003(0.006)** | 0.009(0.004)** |
| year2010age2 | -0.011(0.004)** | -0.014(0.006)** | 0.004(0.007)** | 0.017(0.006)** | 0.014(0.004)** |
| year2011age2 | -0.019(0.004)*** | -0.022(0.006)*** | 0.001(0.007)*** | 0.006(0.006)*** | 0.014(0.004)*** |
| year2012age2 | -0.015(0.004)*** | -0.01(0.006)*** | 0.02(0.007)*** | 0.029(0.006)*** | 0.022(0.004)*** |
| year2013age2 | -0.02(0.004)*** | -0.023(0.005)*** | -0.01(0.007)*** | -0.002(0.006)*** | 0.005(0.004)*** |
| year2014age2 | -0.019(0.004)*** | -0.018(0.005)*** | 0.004(0.006)*** | 0.015(0.006)*** | 0.008(0.004)*** |
| year2015age2 | -0.021(0.004)*** | -0.027(0.005)*** | -0.008(0.007)*** | 0.007(0.006)*** | 0.012(0.004)*** |
| year2016age2 | -0.02(0.004)*** | -0.022(0.005)*** | -0.002(0.007)*** | 0.005(0.006)*** | 0.011(0.004)*** |
| year2017age2 | -0.017(0.003)*** | -0.021(0.005)*** | 0(0.006)*** | 0.01(0.006)*** | 0.018(0.004)*** |
| year2018age2 | -0.016(0.003)*** | -0.023(0.005)*** | -0.004(0.006)*** | 0.013(0.006)*** | 0.012(0.004)*** |
| year2019age2 | -0.017(0.003)*** | -0.021(0.005)*** | -0.001(0.006)*** | 0.02(0.006)*** | 0.014(0.004)*** |
| year1990age3 | -0.012(0.005)** | -0.017(0.006)** | -0.011(0.007)** | -0.004(0.006)** | 0.004(0.004)** |
| year1991age3 | -0.024(0.005)*** | -0.033(0.006)*** | -0.039(0.007)*** | -0.022(0.006)*** | -0.008(0.004)*** |
| year1992age3 | -0.029(0.005)*** | -0.054(0.006)*** | -0.047(0.007)*** | -0.036(0.006)*** | -0.012(0.004)*** |
| year1993age3 | -0.034(0.005)*** | -0.056(0.006)*** | -0.049(0.007)*** | -0.034(0.006)*** | -0.006(0.004)*** |
| year1994age3 | -0.028(0.005)*** | -0.051(0.006)*** | -0.045(0.007)*** | -0.031(0.006)*** | -0.007(0.004)*** |
| year1995age3 | -0.042(0.005)*** | -0.07(0.006)*** | -0.065(0.007)*** | -0.046(0.006)*** | -0.014(0.004)*** |
| year1996age3 | -0.041(0.005)*** | -0.068(0.006)*** | -0.061(0.007)*** | -0.047(0.006)*** | -0.013(0.004)*** |
| year1997age3 | -0.034(0.005)*** | -0.056(0.006)*** | -0.05(0.007)*** | -0.033(0.006)*** | -0.01(0.004)*** |
| year1998age3 | -0.038(0.005)*** | -0.063(0.006)*** | -0.051(0.007)*** | -0.034(0.006)*** | -0.005(0.004)*** |
| year1999age3 | -0.04(0.005)*** | -0.06(0.006)*** | -0.039(0.007)*** | -0.027(0.006)*** | -0.003(0.004)*** |
| year2000age3 | -0.039(0.005)*** | -0.075(0.006)*** | -0.06(0.007)*** | -0.036(0.006)*** | -0.004(0.004)*** |
| year2001age3 | -0.05(0.005)*** | -0.074(0.007)*** | -0.065(0.007)*** | -0.045(0.006)*** | -0.006(0.004)*** |
| year2002age3 | -0.037(0.005)*** | -0.062(0.007)*** | -0.04(0.007)*** | -0.022(0.006)*** | 0.001(0.004)*** |
| Covariate | tau=0.1 | tau=0.25 | tau=0.5 | tau=0.75 | tau=0.9 |
| year2003age3 | -0.045(0.005)*** | -0.071(0.007)*** | -0.064(0.007)*** | -0.041(0.006)*** | -0.009(0.004)*** |
| year2004age3 | -0.05(0.005)*** | -0.079(0.007)*** | -0.061(0.007)*** | -0.035(0.006)*** | -0.004(0.004)*** |
| year2005age3 | -0.035(0.005)*** | -0.066(0.006)*** | -0.047(0.007)*** | -0.031(0.006)*** | -0.006(0.004)*** |
| year2006age3 | -0.033(0.005)*** | -0.062(0.006)*** | -0.047(0.007)*** | -0.021(0.006)*** | 0.002(0.005)*** |
| year2007age3 | -0.027(0.005)*** | -0.049(0.006)*** | -0.032(0.008)*** | -0.02(0.006)*** | 0.009(0.005)*** |
| year2008age3 | -0.029(0.005)*** | -0.045(0.006)*** | -0.028(0.007)*** | -0.012(0.007)*** | 0.009(0.004)*** |
| year2009age3 | -0.033(0.005)*** | -0.06(0.007)*** | -0.034(0.008)*** | -0.017(0.007)*** | 0.006(0.005)*** |
| year2010age3 | -0.027(0.005)*** | -0.052(0.006)*** | -0.036(0.007)*** | -0.011(0.007)*** | 0.006(0.004)*** |
| year2011age3 | -0.034(0.005)*** | -0.06(0.007)*** | -0.029(0.007)*** | -0.02(0.007)*** | 0.007(0.004)*** |
| year2012age3 | -0.028(0.005)*** | -0.044(0.007)*** | -0.014(0.007)*** | 0.004(0.007)*** | 0.015(0.005)*** |
| year2013age3 | -0.032(0.005)*** | -0.056(0.007)*** | -0.045(0.008)*** | -0.02(0.007)*** | 0.008(0.005)*** |
| year2014age3 | -0.038(0.005)*** | -0.07(0.006)*** | -0.044(0.007)*** | -0.013(0.007)*** | 0.006(0.005)*** |
| year2015age3 | -0.04(0.004)*** | -0.068(0.006)*** | -0.055(0.007)*** | -0.024(0.007)*** | 0.006(0.005)*** |
| year2016age3 | -0.047(0.004)*** | -0.085(0.006)*** | -0.068(0.008)*** | -0.034(0.007)*** | 0.002(0.005)*** |
| year2017age3 | -0.043(0.004)*** | -0.073(0.006)*** | -0.042(0.007)*** | -0.024(0.007)*** | 0.007(0.005)*** |
| year2018age3 | -0.041(0.004)*** | -0.079(0.006)*** | -0.059(0.008)*** | -0.035(0.007)*** | 0.004(0.005)*** |
| year2019age3 | -0.041(0.004)*** | -0.075(0.006)*** | -0.055(0.008)*** | -0.028(0.007)*** | -0.001(0.005)*** |

Supplementary Table 3.19: Coefficient estimates (standard errors) for different quantiles (tau) for R600, boys; **p<*0.05, ***p<*0.01,****p<*0.001, age2=10-12 years, age3=13-15 years.

| Covariate | tau=0.1 | tau=0.25 | tau=0.5 | tau=0.75 | tau=0.9 |
| --- | --- | --- | --- | --- | --- |
| (Intercept) | 0.096(0.002)*** | 0.229(0.002)*** | 0.453(0.003)*** | 0.695(0.003)*** | 0.864(0.003)*** |
| year1990 | 0.003(0.002) | 0.003(0.003) | 0.003(0.004) | 0.001(0.004) | -0.006(0.004) |
| year1991 | 0.014(0.002)*** | 0.024(0.003)*** | 0.03(0.004)*** | 0.034(0.004)*** | 0.023(0.004)*** |
| year1992 | 0.014(0.002)*** | 0.025(0.003)*** | 0.033(0.004)*** | 0.033(0.004)*** | 0.019(0.004)*** |
| year1993 | 0.022(0.002)*** | 0.044(0.003)*** | 0.071(0.004)*** | 0.073(0.004)*** | 0.044(0.003)*** |
| year1994 | 0.022(0.002)*** | 0.043(0.003)*** | 0.067(0.004)*** | 0.068(0.004)*** | 0.041(0.004)*** |
| year1995 | 0.041(0.002)*** | 0.071(0.003)*** | 0.107(0.004)*** | 0.101(0.004)*** | 0.059(0.003)*** |
| year1996 | 0.035(0.002)*** | 0.065(0.003)*** | 0.098(0.004)*** | 0.096(0.004)*** | 0.059(0.003)*** |
| year1997 | 0.048(0.002)*** | 0.087(0.003)*** | 0.124(0.004)*** | 0.118(0.004)*** | 0.067(0.003)*** |
| year1998 | 0.046(0.002)*** | 0.086(0.003)*** | 0.121(0.004)*** | 0.117(0.004)*** | 0.068(0.003)*** |
| year1999 | 0.059(0.002)*** | 0.1(0.003)*** | 0.142(0.004)*** | 0.136(0.004)*** | 0.081(0.003)*** |
| year2000 | 0.044(0.002)*** | 0.081(0.003)*** | 0.127(0.004)*** | 0.126(0.004)*** | 0.075(0.003)*** |
| year2001 | 0.049(0.002)*** | 0.092(0.003)*** | 0.136(0.004)*** | 0.136(0.004)*** | 0.082(0.003)*** |
| year2002 | 0.047(0.002)*** | 0.089(0.003)*** | 0.138(0.004)*** | 0.141(0.004)*** | 0.083(0.003)*** |
| year2003 | 0.05(0.002)*** | 0.095(0.003)*** | 0.139(0.004)*** | 0.137(0.004)*** | 0.083(0.003)*** |
| year2004 | 0.05(0.003)*** | 0.102(0.003)*** | 0.149(0.004)*** | 0.145(0.004)*** | 0.086(0.003)*** |
| year2005 | 0.044(0.002)*** | 0.092(0.003)*** | 0.145(0.004)*** | 0.142(0.004)*** | 0.08(0.003)*** |
| year2006 | 0.037(0.003)*** | 0.084(0.004)*** | 0.137(0.004)*** | 0.142(0.004)*** | 0.088(0.003)*** |
| year2007 | 0.034(0.002)*** | 0.076(0.003)*** | 0.132(0.004)*** | 0.137(0.004)*** | 0.082(0.003)*** |
| year2008 | 0.046(0.003)*** | 0.099(0.004)*** | 0.155(0.004)*** | 0.154(0.004)*** | 0.091(0.003)*** |
| year2009 | 0.036(0.002)*** | 0.079(0.003)*** | 0.138(0.004)*** | 0.146(0.004)*** | 0.087(0.003)*** |
| year2010 | 0.036(0.002)*** | 0.083(0.004)*** | 0.14(0.004)*** | 0.144(0.004)*** | 0.088(0.003)*** |
| year2011 | 0.037(0.002)*** | 0.077(0.003)*** | 0.132(0.004)*** | 0.142(0.004)*** | 0.085(0.003)*** |
| year2012 | 0.028(0.002)*** | 0.069(0.003)*** | 0.125(0.004)*** | 0.137(0.004)*** | 0.085(0.003)*** |
| year2013 | 0.029(0.002)*** | 0.07(0.003)*** | 0.131(0.004)*** | 0.137(0.004)*** | 0.085(0.003)*** |
| year2014 | 0.022(0.002)*** | 0.056(0.003)*** | 0.118(0.004)*** | 0.132(0.004)*** | 0.082(0.003)*** |
| year2015 | 0.012(0.002)*** | 0.039(0.003)*** | 0.091(0.004)*** | 0.112(0.004)*** | 0.074(0.003)*** |
| year2016 | 0.014(0.002)*** | 0.041(0.003)*** | 0.093(0.004)*** | 0.116(0.004)*** | 0.076(0.003)*** |
| year2017 | 0.005(0.002)* | 0.029(0.003)* | 0.08(0.004)* | 0.107(0.004)* | 0.073(0.003)* |
| year2018 | 0(0.002) | 0.022(0.003) | 0.061(0.004) | 0.093(0.004) | 0.064(0.003) |
| year2019 | 0.004(0.002)* | 0.027(0.003)* | 0.075(0.004)* | 0.102(0.004)* | 0.072(0.003)* |
| age2 | 0.003(0.002) | 0.009(0.003) | 0.006(0.004) | -0.006(0.004) | -0.014(0.004) |
| age3 | 0.023(0.003)*** | 0.037(0.003)*** | 0.039(0.004)*** | 0.014(0.005)*** | -0.006(0.005)*** |
| region1 | -0.008(0.001)*** | -0.022(0.001)*** | -0.036(0.002)*** | -0.035(0.001)*** | -0.017(0.001)*** |
| region2 | -0.009(0.001)*** | -0.023(0.002)*** | -0.035(0.002)*** | -0.035(0.002)*** | -0.018(0.001)*** |
| region3 | -0.021(0.001)*** | -0.045(0.001)*** | -0.067(0.002)*** | -0.059(0.001)*** | -0.031(0.001)*** |
| region4 | 0.022(0.002)*** | 0.019(0.002)*** | 0.011(0.003)*** | 0.006(0.002)*** | 0(0.001)*** |
| region5 | -0.019(0.001)*** | -0.059(0.002)*** | -0.106(0.002)*** | -0.107(0.002)*** | -0.062(0.001)*** |
| region6 | -0.018(0.001)*** | -0.049(0.002)*** | -0.083(0.002)*** | -0.077(0.002)*** | -0.037(0.001)*** |
| region7 | -0.021(0.001)*** | -0.045(0.001)*** | -0.066(0.002)*** | -0.056(0.001)*** | -0.025(0.001)*** |
| Covariate | tau=0.1 | tau=0.25 | tau=0.5 | tau=0.75 | tau=0.9 |
| region8 | -0.016(0.001)*** | -0.052(0.001)*** | -0.09(0.002)*** | -0.088(0.001)*** | -0.046(0.001)*** |
| region9 | 0.002(0.002) | -0.007(0.002) | -0.023(0.002) | -0.031(0.002) | -0.02(0.001) |
| region10 | -0.046(0.001)*** | -0.094(0.002)*** | -0.138(0.002)*** | -0.137(0.002)*** | -0.08(0.001)*** |
| region11 | -0.008(0.001)*** | -0.026(0.002)*** | -0.048(0.002)*** | -0.051(0.002)*** | -0.027(0.001)*** |
| year1990age2 | 0(0.003) | 0.001(0.004) | 0.002(0.005) | 0.002(0.006) | 0.005(0.005) |
| year1991age2 | -0.003(0.003) | -0.004(0.004) | 0(0.005) | 0.004(0.006) | 0.003(0.005) |
| year1992age2 | -0.005(0.003) | -0.009(0.004) | -0.01(0.005) | -0.011(0.006) | -0.005(0.005) |
| year1993age2 | -0.013(0.003)*** | -0.017(0.004)*** | -0.026(0.005)*** | -0.017(0.006)*** | -0.003(0.005)*** |
| year1994age2 | -0.014(0.003)*** | -0.021(0.004)*** | -0.02(0.005)*** | -0.004(0.006)*** | 0.01(0.005)*** |
| year1995age2 | -0.026(0.003)*** | -0.037(0.004)*** | -0.048(0.005)*** | -0.028(0.005)*** | -0.007(0.005)*** |
| year1996age2 | -0.016(0.003)*** | -0.023(0.004)*** | -0.027(0.005)*** | -0.009(0.006)*** | 0.005(0.005)*** |
| year1997age2 | -0.024(0.003)*** | -0.039(0.004)*** | -0.045(0.005)*** | -0.027(0.005)*** | -0.003(0.005)*** |
| year1998age2 | -0.017(0.003)*** | -0.028(0.004)*** | -0.028(0.005)*** | -0.012(0.006)*** | 0.006(0.005)*** |
| year1999age2 | -0.023(0.003)*** | -0.029(0.005)*** | -0.032(0.006)*** | -0.017(0.006)*** | 0.001(0.005)*** |
| year2000age2 | -0.012(0.003)*** | -0.015(0.005)*** | -0.016(0.005)*** | -0.002(0.006)*** | 0.009(0.005)*** |
| year2001age2 | -0.017(0.003)*** | -0.019(0.005)*** | -0.013(0.006)*** | -0.004(0.006)*** | 0.008(0.005)*** |
| year2002age2 | -0.017(0.003)*** | -0.019(0.005)*** | -0.025(0.006)*** | -0.016(0.006)*** | 0.003(0.005)*** |
| year2003age2 | -0.019(0.003)*** | -0.027(0.005)*** | -0.027(0.006)*** | -0.011(0.006)*** | 0.004(0.005)*** |
| year2004age2 | -0.02(0.004)*** | -0.029(0.005)*** | -0.021(0.006)*** | -0.003(0.006)*** | 0.007(0.005)*** |
| year2005age2 | -0.011(0.004)** | -0.015(0.005)** | -0.011(0.006)** | 0.005(0.006)** | 0.017(0.005)** |
| year2006age2 | -0.007(0.004) | -0.01(0.005) | -0.002(0.006) | 0.011(0.006) | 0.013(0.005) |
| year2007age2 | -0.001(0.004) | 0.008(0.005) | 0.008(0.006) | 0.014(0.006) | 0.018(0.005) |
| year2008age2 | -0.001(0.004) | -0.003(0.005) | 0.008(0.006) | 0.011(0.006) | 0.016(0.005) |
| year2009age2 | 0.002(0.004) | 0.016(0.005) | 0.027(0.006) | 0.028(0.006) | 0.024(0.005) |
| year2010age2 | 0.006(0.004) | 0.02(0.005) | 0.028(0.006) | 0.033(0.006) | 0.025(0.005) |
| year2011age2 | -0.002(0.004) | 0.016(0.005) | 0.028(0.006) | 0.033(0.006) | 0.028(0.005) |
| year2012age2 | 0.004(0.003) | 0.012(0.005) | 0.025(0.006) | 0.032(0.006) | 0.022(0.005) |
| year2013age2 | -0.006(0.004) | 0.004(0.005) | 0.013(0.006) | 0.024(0.006) | 0.023(0.005) |
| year2014age2 | -0.002(0.003) | 0.01(0.005) | 0.015(0.006) | 0.021(0.006) | 0.018(0.005) |
| year2015age2 | 0.006(0.003) | 0.017(0.005) | 0.032(0.006) | 0.035(0.006) | 0.026(0.005) |
| year2016age2 | 0.003(0.003) | 0.016(0.005) | 0.038(0.006) | 0.04(0.006) | 0.028(0.005) |
| year2017age2 | 0.003(0.003) | 0.017(0.005) | 0.038(0.006) | 0.046(0.006) | 0.034(0.005) |
| year2018age2 | 0.009(0.003)** | 0.02(0.005)** | 0.052(0.006)** | 0.054(0.006)** | 0.044(0.005)** |
| year2019age2 | 0.01(0.003)*** | 0.032(0.005)*** | 0.054(0.006)*** | 0.056(0.006)*** | 0.039(0.005)*** |
| year1990age3 | -0.008(0.003)* | -0.008(0.005)* | -0.01(0.006)* | 0(0.006)* | 0.012(0.006)* |
| year1991age3 | -0.01(0.004)** | -0.017(0.005)** | -0.023(0.006)** | -0.017(0.006)** | 0.001(0.006)** |
| year1992age3 | -0.015(0.003)*** | -0.02(0.005)*** | -0.023(0.006)*** | -0.012(0.006)*** | 0.004(0.006)*** |
| year1993age3 | -0.027(0.004)*** | -0.04(0.005)*** | -0.058(0.006)*** | -0.043(0.006)*** | -0.017(0.005)*** |
| year1994age3 | -0.029(0.004)*** | -0.045(0.005)*** | -0.055(0.006)*** | -0.033(0.006)*** | -0.008(0.006)*** |
| year1995age3 | -0.049(0.004)*** | -0.068(0.005)*** | -0.084(0.006)*** | -0.054(0.006)*** | -0.016(0.005)*** |
| year1996age3 | -0.035(0.004)*** | -0.054(0.005)*** | -0.062(0.006)*** | -0.036(0.006)*** | -0.009(0.005)*** |
| year1997age3 | -0.042(0.004)*** | -0.064(0.005)*** | -0.079(0.006)*** | -0.047(0.006)*** | -0.008(0.005)*** |
| year1998age3 | -0.034(0.004)*** | -0.049(0.005)*** | -0.049(0.006)*** | -0.028(0.006)*** | 0.002(0.005)*** |
| year1999age3 | -0.037(0.004)*** | -0.049(0.005)*** | -0.055(0.006)*** | -0.03(0.006)*** | -0.003(0.005)*** |
| year2000age3 | -0.028(0.004)*** | -0.035(0.005)*** | -0.043(0.006)*** | -0.016(0.006)*** | 0.007(0.005)*** |
| Covariate | tau=0.1 | tau=0.25 | tau=0.5 | tau=0.75 | tau=0.9 |
| year2001age3 | -0.029(0.004)*** | -0.04(0.005)*** | -0.043(0.006)*** | -0.017(0.006)*** | 0.001(0.005)*** |
| year2002age3 | -0.034(0.004)*** | -0.05(0.006)*** | -0.05(0.007)*** | -0.028(0.006)*** | 0(0.005)*** |
| year2003age3 | -0.036(0.004)*** | -0.048(0.006)*** | -0.038(0.006)*** | -0.019(0.006)*** | 0.002(0.005)*** |
| year2004age3 | -0.041(0.004)*** | -0.045(0.006)*** | -0.035(0.007)*** | -0.007(0.006)*** | 0.008(0.005)*** |
| year2005age3 | -0.032(0.004)*** | -0.041(0.006)*** | -0.039(0.007)*** | -0.009(0.006)*** | 0.014(0.005)*** |
| year2006age3 | -0.031(0.004)*** | -0.041(0.006)*** | -0.038(0.007)*** | -0.017(0.006)*** | 0.004(0.005)*** |
| year2007age3 | -0.028(0.004)*** | -0.042(0.006)*** | -0.041(0.007)*** | -0.008(0.006)*** | 0.009(0.005)*** |
| year2008age3 | -0.03(0.004)*** | -0.039(0.006)*** | -0.038(0.007)*** | -0.009(0.006)*** | 0.009(0.005)*** |
| year2009age3 | -0.023(0.004)*** | -0.019(0.006)*** | -0.016(0.007)*** | 0.009(0.006)*** | 0.015(0.005)*** |
| year2010age3 | -0.027(0.004)*** | -0.024(0.006)*** | -0.021(0.007)*** | 0.001(0.006)*** | 0.013(0.005)*** |
| year2011age3 | -0.032(0.004)*** | -0.03(0.006)*** | -0.011(0.007)*** | 0.001(0.007)*** | 0.014(0.005)*** |
| year2012age3 | -0.021(0.004)*** | -0.024(0.006)*** | -0.004(0.007)*** | 0.006(0.006)*** | 0.013(0.005)*** |
| year2013age3 | -0.035(0.004)*** | -0.04(0.006)*** | -0.025(0.007)*** | 0.008(0.007)*** | 0.016(0.005)*** |
| year2014age3 | -0.035(0.004)*** | -0.04(0.006)*** | -0.033(0.007)*** | -0.005(0.007)*** | 0.014(0.005)*** |
| year2015age3 | -0.036(0.004)*** | -0.038(0.006)*** | -0.025(0.007)*** | 0.005(0.007)*** | 0.017(0.005)*** |
| year2016age3 | -0.038(0.004)*** | -0.041(0.006)*** | -0.026(0.007)*** | 0.003(0.007)*** | 0.017(0.005)*** |
| year2017age3 | -0.028(0.004)*** | -0.032(0.006)*** | -0.02(0.007)*** | 0.007(0.007)*** | 0.016(0.005)*** |
| year2018age3 | -0.015(0.004)*** | -0.013(0.006)*** | 0.015(0.007)*** | 0.031(0.006)*** | 0.028(0.005)*** |
| year2019age3 | -0.013(0.004)** | -0.002(0.006)** | 0.033(0.007)** | 0.051(0.007)** | 0.035(0.005)** |

Supplementary Table 3.20: Coefficient estimates (standard errors) for different quantiles (tau) for R600, girls; **p<*0.05, ***p<*0.01,****p<*0.001, age2=10-12 years, age3=13-15 years.

| Covariate | tau=0.1 | tau=0.25 | tau=0.5 | tau=0.75 | tau=0.9 |
| --- | --- | --- | --- | --- | --- |
| (Intercept) | 0.105(0.002)*** | 0.249(0.002)*** | 0.481(0.003)*** | 0.715(0.003)*** | 0.875(0.003)*** |
| year1990 | 0.003(0.002) | 0.003(0.003) | -0.001(0.004) | -0.005(0.004) | -0.008(0.004) |
| year1991 | 0.012(0.002)*** | 0.023(0.003)*** | 0.032(0.004)*** | 0.03(0.004)*** | 0.022(0.004)*** |
| year1992 | 0.009(0.002)*** | 0.018(0.003)*** | 0.025(0.004)*** | 0.023(0.004)*** | 0.01(0.004)*** |
| year1993 | 0.02(0.002)*** | 0.042(0.003)*** | 0.066(0.004)*** | 0.067(0.004)*** | 0.041(0.004)*** |
| year1994 | 0.02(0.002)*** | 0.045(0.003)*** | 0.066(0.004)*** | 0.065(0.004)*** | 0.033(0.004)*** |
| year1995 | 0.041(0.002)*** | 0.078(0.003)*** | 0.11(0.004)*** | 0.099(0.004)*** | 0.056(0.004)*** |
| year1996 | 0.033(0.002)*** | 0.076(0.003)*** | 0.108(0.004)*** | 0.099(0.004)*** | 0.053(0.003)*** |
| year1997 | 0.049(0.002)*** | 0.096(0.003)*** | 0.13(0.004)*** | 0.115(0.004)*** | 0.063(0.003)*** |
| year1998 | 0.056(0.003)*** | 0.106(0.003)*** | 0.147(0.004)*** | 0.13(0.004)*** | 0.065(0.003)*** |
| year1999 | 0.074(0.003)*** | 0.13(0.003)*** | 0.171(0.004)*** | 0.148(0.004)*** | 0.081(0.003)*** |
| year2000 | 0.058(0.002)*** | 0.111(0.003)*** | 0.157(0.004)*** | 0.144(0.004)*** | 0.076(0.003)*** |
| year2001 | 0.07(0.003)*** | 0.125(0.004)*** | 0.173(0.004)*** | 0.151(0.004)*** | 0.083(0.003)*** |
| year2002 | 0.06(0.003)*** | 0.119(0.004)*** | 0.165(0.004)*** | 0.146(0.004)*** | 0.085(0.003)*** |
| year2003 | 0.063(0.003)*** | 0.122(0.004)*** | 0.173(0.004)*** | 0.153(0.004)*** | 0.085(0.003)*** |
| year2004 | 0.058(0.003)*** | 0.119(0.004)*** | 0.167(0.004)*** | 0.151(0.004)*** | 0.085(0.003)*** |
| year2005 | 0.052(0.003)*** | 0.106(0.003)*** | 0.152(0.004)*** | 0.14(0.004)*** | 0.078(0.003)*** |
| year2006 | 0.038(0.002)*** | 0.09(0.004)*** | 0.135(0.004)*** | 0.124(0.004)*** | 0.072(0.004)*** |
| year2007 | 0.036(0.002)*** | 0.083(0.004)*** | 0.125(0.004)*** | 0.121(0.004)*** | 0.07(0.004)*** |
| year2008 | 0.047(0.003)*** | 0.1(0.004)*** | 0.149(0.004)*** | 0.138(0.004)*** | 0.079(0.003)*** |
| year2009 | 0.036(0.002)*** | 0.085(0.003)*** | 0.135(0.004)*** | 0.131(0.004)*** | 0.075(0.003)*** |
| year2010 | 0.034(0.002)*** | 0.086(0.004)*** | 0.142(0.005)*** | 0.133(0.004)*** | 0.076(0.004)*** |
| year2011 | 0.036(0.002)*** | 0.079(0.004)*** | 0.127(0.004)*** | 0.119(0.004)*** | 0.068(0.003)*** |
| year2012 | 0.03(0.002)*** | 0.076(0.004)*** | 0.127(0.004)*** | 0.119(0.004)*** | 0.066(0.004)*** |
| year2013 | 0.032(0.002)*** | 0.076(0.004)*** | 0.128(0.004)*** | 0.117(0.004)*** | 0.068(0.003)*** |
| year2014 | 0.025(0.002)*** | 0.07(0.004)*** | 0.116(0.004)*** | 0.109(0.004)*** | 0.065(0.004)*** |
| year2015 | 0.017(0.002)*** | 0.052(0.003)*** | 0.097(0.004)*** | 0.098(0.004)*** | 0.057(0.004)*** |
| year2016 | 0.022(0.002)*** | 0.059(0.003)*** | 0.101(0.004)*** | 0.108(0.004)*** | 0.063(0.004)*** |
| year2017 | 0.015(0.002)*** | 0.045(0.003)*** | 0.082(0.004)*** | 0.095(0.004)*** | 0.058(0.004)*** |
| year2018 | 0.01(0.002)*** | 0.035(0.003)*** | 0.069(0.004)*** | 0.077(0.004)*** | 0.046(0.004)*** |
| year2019 | 0.015(0.002)*** | 0.042(0.003)*** | 0.082(0.004)*** | 0.088(0.004)*** | 0.053(0.004)*** |
| age2 | 0.009(0.002)*** | 0.015(0.003)*** | 0.016(0.004)*** | 0.003(0.005)*** | -0.01(0.004)*** |
| age3 | 0.017(0.002)*** | 0.024(0.004)*** | 0.024(0.004)*** | 0.006(0.005)*** | -0.012(0.005)*** |
| region1 | -0.011(0.001)*** | -0.028(0.002)*** | -0.039(0.002)*** | -0.032(0.001)*** | -0.018(0.001)*** |
| region2 | -0.021(0.002)*** | -0.047(0.002)*** | -0.06(0.002)*** | -0.044(0.002)*** | -0.022(0.001)*** |
| region3 | -0.04(0.001)*** | -0.08(0.002)*** | -0.107(0.002)*** | -0.084(0.001)*** | -0.045(0.001)*** |
| region4 | 0.018(0.002)*** | 0.016(0.003)*** | 0.011(0.003)*** | -0.001(0.002)*** | -0.005(0.001)*** |
| region5 | -0.039(0.001)*** | -0.095(0.002)*** | -0.147(0.002)*** | -0.134(0.002)*** | -0.081(0.001)*** |
| region6 | -0.029(0.001)*** | -0.075(0.002)*** | -0.111(0.002)*** | -0.092(0.001)*** | -0.044(0.001)*** |
| region7 | -0.041(0.001)*** | -0.08(0.001)*** | -0.099(0.002)*** | -0.07(0.001)*** | -0.029(0.001)*** |
| region8 | -0.04(0.001)*** | -0.093(0.002)*** | -0.139(0.002)*** | -0.118(0.001)*** | -0.062(0.001)*** |
| region9 | -0.011(0.002)*** | -0.03(0.002)*** | -0.054(0.002)*** | -0.056(0.002)*** | -0.035(0.001)*** |
| Covariate | tau=0.1 | tau=0.25 | tau=0.5 | tau=0.75 | tau=0.9 |
| region10 | -0.067(0.001)*** | -0.136(0.002)*** | -0.198(0.002)*** | -0.182(0.002)*** | -0.11(0.001)*** |
| region11 | -0.014(0.001)*** | -0.042(0.002)*** | -0.074(0.002)*** | -0.067(0.002)*** | -0.039(0.001)*** |
| year1990age2 | 0.002(0.003) | 0.005(0.004) | 0.007(0.005) | 0.011(0.006) | 0.014(0.005) |
| year1991age2 | -0.001(0.003) | -0.004(0.004) | -0.005(0.006) | 0.007(0.006) | 0.005(0.005) |
| year1992age2 | -0.003(0.003) | -0.005(0.004) | -0.009(0.005) | 0.003(0.006) | 0.009(0.005) |
| year1993age2 | -0.01(0.003)*** | -0.018(0.004)*** | -0.02(0.006)*** | -0.008(0.006)*** | 0.001(0.005)*** |
| year1994age2 | -0.011(0.003)*** | -0.021(0.004)*** | -0.026(0.006)*** | -0.012(0.006)*** | 0.005(0.005)*** |
| year1995age2 | -0.023(0.003)*** | -0.043(0.004)*** | -0.05(0.006)*** | -0.028(0.006)*** | -0.008(0.005)*** |
| year1996age2 | -0.016(0.003)*** | -0.039(0.004)*** | -0.047(0.006)*** | -0.026(0.006)*** | -0.001(0.005)*** |
| year1997age2 | -0.023(0.003)*** | -0.043(0.004)*** | -0.04(0.006)*** | -0.019(0.006)*** | 0.002(0.005)*** |
| year1998age2 | -0.019(0.003)*** | -0.031(0.005)*** | -0.039(0.006)*** | -0.018(0.006)*** | 0.007(0.005)*** |
| year1999age2 | -0.027(0.004)*** | -0.036(0.005)*** | -0.034(0.006)*** | -0.015(0.006)*** | 0(0.005)*** |
| year2000age2 | -0.013(0.003)*** | -0.023(0.005)*** | -0.025(0.006)*** | -0.016(0.006)*** | 0.005(0.005)*** |
| year2001age2 | -0.028(0.004)*** | -0.035(0.005)*** | -0.036(0.006)*** | -0.016(0.006)*** | 0.002(0.005)*** |
| year2002age2 | -0.021(0.004)*** | -0.039(0.005)*** | -0.042(0.006)*** | -0.022(0.006)*** | -0.005(0.005)*** |
| year2003age2 | -0.022(0.004)*** | -0.034(0.005)*** | -0.038(0.006)*** | -0.022(0.006)*** | 0(0.005)*** |
| year2004age2 | -0.022(0.004)*** | -0.033(0.005)*** | -0.026(0.006)*** | -0.017(0.006)*** | 0.005(0.005)*** |
| year2005age2 | -0.022(0.004)*** | -0.026(0.005)*** | -0.026(0.006)*** | -0.011(0.006)*** | 0.006(0.005)*** |
| year2006age2 | -0.009(0.004)* | -0.017(0.005)* | -0.011(0.006)* | 0.004(0.006)* | 0.013(0.005)* |
| year2007age2 | -0.008(0.004)* | -0.009(0.005)* | 0(0.006)* | 0.013(0.006)* | 0.016(0.005)* |
| year2008age2 | -0.006(0.004) | -0.008(0.005) | -0.004(0.006) | 0.004(0.006) | 0.013(0.005) |
| year2009age2 | 0(0.004) | 0.009(0.005) | 0.009(0.006) | 0.013(0.006) | 0.021(0.005) |
| year2010age2 | -0.001(0.004) | 0.002(0.005) | 0.004(0.006) | 0.019(0.006) | 0.02(0.005) |
| year2011age2 | -0.006(0.004) | 0.001(0.005) | 0.015(0.006) | 0.031(0.006) | 0.029(0.005) |
| year2012age2 | -0.003(0.003) | 0.001(0.005) | 0.008(0.006) | 0.022(0.006) | 0.027(0.005) |
| year2013age2 | -0.009(0.004)** | -0.002(0.005)** | 0.001(0.006)** | 0.017(0.006)** | 0.023(0.005)** |
| year2014age2 | -0.006(0.003) | -0.01(0.005) | -0.008(0.006) | 0.013(0.006) | 0.018(0.005) |
| year2015age2 | -0.005(0.003) | -0.01(0.005) | 0.001(0.006) | 0.014(0.006) | 0.019(0.005) |
| year2016age2 | -0.008(0.003)** | -0.008(0.005)** | 0(0.006)** | 0.006(0.006)** | 0.018(0.005)** |
| year2017age2 | -0.011(0.003)*** | -0.01(0.005)*** | 0.002(0.006)*** | 0.014(0.006)*** | 0.019(0.005)*** |
| year2018age2 | -0.003(0.003) | -0.002(0.005) | 0.014(0.006) | 0.024(0.006) | 0.032(0.005) |
| year2019age2 | -0.005(0.003) | 0.003(0.005) | 0.017(0.006) | 0.029(0.006) | 0.029(0.005) |
| year1990age3 | 0.001(0.003) | 0.005(0.005) | 0.009(0.006) | 0.02(0.007) | 0.023(0.006) |
| year1991age3 | -0.007(0.003)* | -0.011(0.005)* | -0.019(0.006)* | -0.005(0.007)* | 0.002(0.006)* |
| year1992age3 | -0.005(0.003) | 0(0.005) | -0.006(0.006) | 0.003(0.007) | 0.01(0.006) |
| year1993age3 | -0.012(0.003)*** | -0.017(0.005)*** | -0.027(0.006)*** | -0.016(0.007)*** | -0.004(0.006)*** |
| year1994age3 | -0.017(0.003)*** | -0.025(0.005)*** | -0.027(0.006)*** | -0.012(0.007)*** | 0.005(0.006)*** |
| year1995age3 | -0.035(0.004)*** | -0.056(0.005)*** | -0.063(0.006)*** | -0.043(0.007)*** | -0.013(0.006)*** |
| year1996age3 | -0.024(0.004)*** | -0.047(0.005)*** | -0.048(0.006)*** | -0.029(0.007)*** | -0.001(0.006)*** |
| year1997age3 | -0.028(0.004)*** | -0.048(0.005)*** | -0.055(0.006)*** | -0.03(0.006)*** | -0.004(0.006)*** |
| year1998age3 | -0.026(0.004)*** | -0.039(0.006)*** | -0.041(0.006)*** | -0.018(0.006)*** | 0.012(0.005)*** |
| year1999age3 | -0.033(0.004)*** | -0.044(0.006)*** | -0.044(0.006)*** | -0.019(0.006)*** | 0.002(0.005)*** |
| year2000age3 | -0.028(0.004)*** | -0.039(0.006)*** | -0.039(0.007)*** | -0.024(0.007)*** | 0.006(0.005)*** |
| year2001age3 | -0.042(0.004)*** | -0.055(0.006)*** | -0.057(0.007)*** | -0.026(0.006)*** | 0.002(0.005)*** |
| year2002age3 | -0.036(0.004)*** | -0.051(0.006)*** | -0.05(0.007)*** | -0.026(0.007)*** | -0.004(0.005)*** |
| Covariate | tau=0.1 | tau=0.25 | tau=0.5 | tau=0.75 | tau=0.9 |
| year2003age3 | -0.034(0.004)*** | -0.048(0.006)*** | -0.051(0.007)*** | -0.024(0.006)*** | 0.004(0.005)*** |
| year2004age3 | -0.029(0.004)*** | -0.036(0.006)*** | -0.034(0.007)*** | -0.011(0.007)*** | 0.009(0.005)*** |
| year2005age3 | -0.027(0.004)*** | -0.037(0.006)*** | -0.032(0.007)*** | -0.005(0.007)*** | 0.014(0.005)*** |
| year2006age3 | -0.02(0.004)*** | -0.029(0.006)*** | -0.016(0.007)*** | 0.008(0.007)*** | 0.021(0.006)*** |
| year2007age3 | -0.014(0.004)** | -0.014(0.006)** | -0.003(0.007)** | 0.01(0.007)** | 0.023(0.006)** |
| year2008age3 | -0.012(0.005)** | -0.003(0.006)** | 0.001(0.007)** | 0.013(0.007)** | 0.024(0.005)** |
| year2009age3 | -0.002(0.004) | 0.012(0.006) | 0.011(0.007) | 0.02(0.007) | 0.028(0.006) |
| year2010age3 | -0.004(0.004) | -0.002(0.006) | 0(0.007) | 0.01(0.007) | 0.017(0.006) |
| year2011age3 | -0.011(0.004)** | -0.004(0.006)** | 0.002(0.007)** | 0.022(0.007)** | 0.033(0.006)** |
| year2012age3 | -0.019(0.004)*** | -0.015(0.006)*** | -0.002(0.007)*** | 0.02(0.007)*** | 0.03(0.006)*** |
| year2013age3 | -0.02(0.004)*** | -0.019(0.006)*** | -0.002(0.007)*** | 0.023(0.007)*** | 0.029(0.006)*** |
| year2014age3 | -0.019(0.004)*** | -0.022(0.006)*** | -0.014(0.007)*** | 0.014(0.007)*** | 0.019(0.006)*** |
| year2015age3 | -0.018(0.004)*** | -0.02(0.006)*** | -0.007(0.007)*** | 0.017(0.007)*** | 0.026(0.006)*** |
| year2016age3 | -0.029(0.004)*** | -0.039(0.006)*** | -0.021(0.007)*** | 0.005(0.007)*** | 0.023(0.006)*** |
| year2017age3 | -0.028(0.004)*** | -0.036(0.006)*** | -0.007(0.007)*** | 0.012(0.007)*** | 0.022(0.006)*** |
| year2018age3 | -0.021(0.004)*** | -0.017(0.006)*** | 0.002(0.007)*** | 0.025(0.007)*** | 0.03(0.006)*** |
| year2019age3 | -0.023(0.004)*** | -0.019(0.006)*** | 0(0.007)*** | 0.019(0.007)*** | 0.028(0.006)*** |

# 4. Results of joinpoint (segmented) regression analysis

Supplementary Table 4.1: Results of joinpoint regression analysis for BMI: estimates of the joinpoints (standard errors), ACP/AACP with 95% CI by gender and age groups.

| Gender | Age group | Segment | Estimate | 95% CI |
| --- | --- | --- | --- | --- |
| Boys | 7-9 | 1989- | 0.27 | (0.23 to 0.3) |
| Boys | 7-9 | 2003(0.39) | 1 | (0.83 to 1.17) |
| Boys | 7-9 | 2008(0.22) | -0.6 | (-0.66 to -0.54) |
| Boys | 7-9 | 1989-2019 | 0.08 | (0.07 to 0.09) |
| Boys | 10-12 | 1989- | 0.55 | (0.52 to 0.58) |
| Boys | 10-12 | 2003(0.63) | 1.09 | (0.93 to 1.25) |
| Boys | 10-12 | 2010(0.23) | -0.57 | (-0.64 to -0.49) |
| Boys | 10-12 | 1989-2019 | 0.33 | (0.32 to 0.35) |
| Boys | 13-15 | 1989- | 0.7 | (0.59 to 0.82) |
| Boys | 13-15 | 1995(0.63) | 0.08 | (-0.2 to 0.36) |
| Boys | 13-15 | 1999(0.48) | 0.84 | (0.76 to 0.91) |
| Boys | 13-15 | 2010(0.28) | -0.52 | (-0.63 to -0.41) |
| boys | 13-15 | 1989-2019 | 0.32 | (0.3 to 0.35) |
| Girls | 7-9 | 1989- | 0.43 | (0.41 to 0.45) |
| Girls | 7-9 | 2009(0.31) | -0.46 | (-0.53 to -0.38) |
| Girls | 7-9 | 1989-2019 | 0.14 | (0.13 to 0.16) |
| Girls | 10-12 | 1989- | 0.39 | (0.36 to 0.42) |
| Girls | 10-12 | 2004(0.76) | 0.88 | (0.67 to 1.09) |
| Girls | 10-12 | 2010(0.31) | -0.47 | (-0.55 to -0.4) |
| Girls | 10-12 | 1989-2019 | 0.23 | (0.22 to 0.25) |
| Girls | 13-15 | 1989- | 0.19 | (0.06 to 0.32) |
| Girls | 13-15 | 1996(0.85) | -0.24 | (-0.4 to -0.08) |
| Girls | 13-15 | 2002(0.35) | 0.87 | (0.76 to 0.97) |
| Girls | 13-15 | 2011(0.44) | -0.1 | (-0.22 to 0.02) |
| Girls | 13-15 | 1989-2019 | 0.24 | (0.21 to 0.26) |

Supplementary Table 4.2: Results of joinpoint regression analysis for TSF: estimates of the joinpoints (standard errors), ACP/AACP with 95% CI by gender and age groups.

| Gender | Age group | Segment | Estimate | 95% CI |
| --- | --- | --- | --- | --- |
| Boys | 7-9 | 1989- | 0.36 | (0.3 to 0.41) |
| Boys | 7-9 | 1999(0.26) | 1.43 | (1.33 to 1.53) |
| Boys | 7-9 | 2008(0.2) | -0.28 | (-0.34 to -0.22) |
| Boys | 7-9 | 1989-2019 | 0.42 | (0.4 to 0.43) |
| Boys | 10-12 | 1989- | 0.19 | (0.14 to 0.24) |
| Boys | 10-12 | 1997(0.21) | 1.29 | (1.24 to 1.35) |
| Boys | 10-12 | 2009(0.22) | -0.1 | (-0.18 to -0.01) |
| Boys | 10-12 | 1989-2019 | 0.55 | (0.54 to 0.57) |
| Boys | 13-15 | 1989- | 0 | (-0.06 to 0.06) |
| Boys | 13-15 | 1999(0.23) | 1.2 | (1.13 to 1.26) |
| Boys | 13-15 | 2009(0.28) | -0.09 | (-0.18 to 0) |
| Boys | 13-15 | 1989-2019 | 0.4 | (0.38 to 0.42) |
| Girls | 7-9 | 1989- | 0.4 | (0.34 to 0.45) |
| Girls | 7-9 | 1998(0.32) | 1.47 | (1.34 to 1.6) |
| Girls | 7-9 | 2006(0.2) | -0.06 | (-0.1 to -0.02) |
| Girls | 7-9 | 1989-2019 | 0.46 | (0.45 to 0.47) |
| Girls | 10-12 | 1989- | -0.16 | (-0.33 to 0.02) |
| Girls | 10-12 | 1992(0.38) | 0.62 | (0.37 to 0.87) |
| Girls | 10-12 | 1997(0.91) | 1.09 | (1.04 to 1.14) |
| Girls | 10-12 | 2010(0.2) | -0.18 | (-0.24 to -0.12) |
| Girls | 10-12 | 1989-2019 | 0.48 | (0.46 to 0.49) |
| Girls | 13-15 | 1989- | -0.56 | (-0.86 to -0.25) |
| Girls | 13-15 | 1993(0.55) | 0.23 | (0.1 to 0.35) |
| Girls | 13-15 | 2000(0.33) | 1.27 | (1.2 to 1.34) |
| Girls | 13-15 | 2011(0.24) | -0.35 | (-0.47 to -0.24) |
| Girls | 13-15 | 1989-2019 | 0.35 | (0.33 to 0.38) |

Supplementary Table 4.3: Results of joinpoint regression analysis for APT: estimates of the joinpoints (standard errors), ACP/AACP with 95% CI by gender and age groups.

| Gender | Age group | Segment | Estimate | 95% CI |
| --- | --- | --- | --- | --- |
| Boys | 7-9 | 1989- | 1.59 | (1.32 to 1.86) |
| Boys | 7-9 | 1992(0.16) | -0.85 | (-0.96 to -0.73) |
| Boys | 7-9 | 1999(0.14) | 1.16 | (1.12 to 1.19) |
| Boys | 7-9 | 2014(0.3) | 0.1 | (-0.07 to 0.27) |
| Boys | 7-9 | 1989-2019 | 0.57 | (0.55 to 0.59) |
| Boys | 10-12 | 1989- | 5.57 | (5.3 to 5.84) |
| Boys | 10-12 | 1992(0.05) | -1.13 | (-1.2 to -1.05) |
| Boys | 10-12 | 2001(0.15) | 0.52 | (0.48 to 0.55) |
| Boys | 10-12 | 2016(0.76) | -0.07 | (-0.47 to 0.33) |
| Boys | 10-12 | 1989-2019 | 0.45 | (0.43 to 0.48) |
| Boys | 13-15 | 1989- | 2.97 | (2.8 to 3.15) |
| Boys | 13-15 | 1994(0.09) | -2.59 | (-3.05 to -2.13) |
| Boys | 13-15 | 1998(0.21) | -0.33 | (-0.4 to -0.26) |
| Boys | 13-15 | 2008(0.56) | 0.29 | (0.21 to 0.36) |
| Boys | 13-15 | 1989-2019 | 0.24 | (0.22 to 0.26) |
| Girls | 7-9 | 1989- | 6.36 | (5.49 to 7.24) |
| Girls | 7-9 | 1990(0.06) | -0.92 | (-1 to -0.84) |
| Girls | 7-9 | 1999(0.12) | 1.16 | (1.12 to 1.19) |
| Girls | 7-9 | 2016(0.14) | -0.86 | (-1.1 to -0.62) |
| Girls | 7-9 | 1989-2019 | 0.57 | (0.54 to 0.59) |
| Girls | 10-12 | 1989- | 6.12 | (5.84 to 6.4) |
| Girls | 10-12 | 1992(0.05) | -1.21 | (-1.31 to -1.11) |
| Girls | 10-12 | 1999(0.14) | 0.62 | (0.6 to 0.64) |
| Girls | 10-12 | 1989-2019 | 0.76 | (0.74 to 0.78) |
| Girls | 13-15 | 1989- | 3.31 | (3.13 to 3.49) |
| Girls | 13-15 | 1995(0.09) | -2.66 | (-3.13 to -2.18) |
| Girls | 13-15 | 1997(0.16) | -0.07 | (-0.13 to 0) |
| Girls | 13-15 | 2009(0.38) | 0.9 | (0.81 to 0.99) |
| Girls | 13-15 | 1989-2019 | 0.62 | (0.59 to 0.64) |

Supplementary Table 4.4: Results of joinpoint regression analysis for SBJ: estimates of the joinpoints (standard errors), ACP/AACP with 95% CI by gender and age groups.

| Gender | Age group | Segment | Estimate | 95% CI |
| --- | --- | --- | --- | --- |
| Boys | 7-9 | 1989- | -1 | (-1.16 to -0.83) |
| Boys | 7-9 | 1994(0.2) | 0.76 | (0.58 to 0.94) |
| Boys | 7-9 | 1998(0.17) | -1.07 | (-1.12 to -1.01) |
| Boys | 7-9 | 2011(0.28) | -0.07 | (-0.15 to 0.01) |
| Boys | 7-9 | 1989-2019 | -0.51 | (-0.52 to -0.49) |
| Boys | 10-12 | 1989- | -0.25 | (-0.3 to -0.19) |
| Boys | 10-12 | 1999(0.27) | -1.08 | (-1.12 to -1.04) |
| Boys | 10-12 | 2011(0.36) | -0.24 | (-0.34 to -0.14) |
| Boys | 10-12 | 1989-2019 | -0.59 | (-0.61 to -0.57) |
| Boys | 13-15 | 1989- | 2.17 | (1.68 to 2.67) |
| Boys | 13-15 | 1991(0.17) | -0.46 | (-0.61 to -0.32) |
| Boys | 13-15 | 1997(0.87) | -0.81 | (-0.85 to -0.76) |
| Boys | 13-15 | 2012(0.55) | -0.04 | (-0.2 to 0.13) |
| Boys | 13-15 | 1989-2019 | -0.32 | (-0.35 to -0.29) |
| Girls | 7-9 | 1989- | -0.84 | (-1.01 to -0.66) |
| Girls | 7-9 | 1994(0.65) | -0.3 | (-0.37 to -0.22) |
| Girls | 7-9 | 2002(0.4) | -1.21 | (-1.41 to -1) |
| Girls | 7-9 | 2008(0.4) | -0.24 | (-0.3 to -0.19) |
| Girls | 7-9 | 1989-2019 | -0.54 | (-0.55 to -0.52) |
| Girls | 10-12 | 1989- | 0.05 | (-0.07 to 0.17) |
| Girls | 10-12 | 1994(0.28) | -0.82 | (-0.86 to -0.79) |
| Girls | 10-12 | 2011(0.39) | -0.12 | (-0.21 to -0.03) |
| Girls | 10-12 | 1989-2019 | -0.48 | (-0.5 to -0.46) |
| Girls | 13-15 | 1989- | 0.24 | (0.09 to 0.39) |
| Girls | 13-15 | 1995(0.25) | -1.01 | (-1.05 to -0.98) |
| Girls | 13-15 | 2012(0.21) | 0.71 | (0.57 to 0.84) |
| Girls | 13-15 | 1989-2019 | -0.35 | (-0.38 to -0.33) |

Supplementary Table 4.5: Results of joinpoint regression analysis for BOC: estimates of the joinpoints (standard errors), ACP/AACP with 95% CI by gender and age groups.

| Gender | Age group | Segment | Estimate | 95% CI |
| --- | --- | --- | --- | --- |
| Boys | 7-9 | 1989- | -3.16 | (-3.89 to -2.42) |
| Boys | 7-9 | 1990(0.1) | -0.23 | (-0.24 to -0.22) |
| Boys | 7-9 | 1989-2019 | -0.33 | (-0.35 to -0.32) |
| Boys | 10-12 | 1989- | -2.69 | (-2.86 to -2.53) |
| Boys | 10-12 | 1993(0.09) | 0.48 | (0.42 to 0.54) |
| Boys | 10-12 | 2004(0.39) | 1.31 | (1.1 to 1.51) |
| Boys | 10-12 | 2008(0.24) | -0.21 | (-0.28 to -0.15) |
| Boys | 10-12 | 1989-2019 | -0.09 | (-0.11 to -0.07) |
| Boys | 13-15 | 1989- | -2.45 | (-2.65 to -2.25) |
| Boys | 13-15 | 1994(0.17) | 0.16 | (-0.26 to 0.58) |
| Boys | 13-15 | 1997(0.73) | 0.83 | (0.78 to 0.88) |
| Boys | 13-15 | 2011(0.28) | -0.43 | (-0.54 to -0.32) |
| Boys | 13-15 | 1989-2019 | -0.1 | (-0.13 to -0.08) |
| Girls | 7-9 | 1989- | -4.43 | (-5.17 to -3.69) |
| Girls | 7-9 | 1990(0.08) | -0.64 | (-0.65 to -0.63) |
| Girls | 7-9 | 1989-2019 | -0.77 | (-0.79 to -0.75) |
| Girls | 10-12 | 1989- | -4.85 | (-5.08 to -4.63) |
| Girls | 10-12 | 1992(0.06) | -0.05 | (-0.1 to -0.01) |
| Girls | 10-12 | 2004(0.4) | 0.83 | (0.62 to 1.04) |
| Girls | 10-12 | 2009(0.19) | -1.08 | (-1.15 to -1.01) |
| Girls | 10-12 | 1989-2019 | -0.79 | (-0.81 to -0.77) |
| Girls | 13-15 | 1989- | -3.39 | (-3.54 to -3.23) |
| Girls | 13-15 | 1994(0.09) | 0.47 | (0.4 to 0.53) |
| Girls | 13-15 | 2005(0.7) | 1.07 | (0.86 to 1.27) |
| Girls | 13-15 | 2011(0.18) | -1.66 | (-1.8 to -1.52) |
| Girls | 13-15 | 1989-2019 | -0.68 | (-0.7 to -0.65) |

Supplementary Table 4.6: Results of joinpoint regression analysis for SU60: estimates of the joinpoints (standard errors), ACP/AACP with 95% CI by gender and age groups.

| Gender | Age group | Segment | Estimate | 95% CI |
| --- | --- | --- | --- | --- |
| Boys | 7-9 | 1989- | 0.21 | (-0.03 to 0.44) |
| Boys | 7-9 | 1993(0.47) | 0.88 | (0.86 to 0.9) |
| Boys | 7-9 | 2014(0.46) | 0.13 | (-0.06 to 0.31) |
| Boys | 7-9 | 1989-2019 | 0.67 | (0.65 to 0.69) |
| Boys | 10-12 | 1989- | 1.91 | (1.57 to 2.25) |
| Boys | 10-12 | 1991(0.4) | 1.13 | (1.05 to 1.21) |
| Boys | 10-12 | 2000(0.53) | 0.68 | (0.65 to 0.71) |
| Boys | 10-12 | 2015(0.39) | -0.32 | (-0.6 to -0.04) |
| Boys | 10-12 | 1989-2019 | 0.78 | (0.75 to 0.8) |
| Boys | 13-15 | 1989- | 2.56 | (2.38 to 2.74) |
| Boys | 13-15 | 1993(0.45) | 1.68 | (1.49 to 1.88) |
| Boys | 13-15 | 1999(0.35) | 0.58 | (0.54 to 0.62) |
| Boys | 13-15 | 2016(0.26) | -1.11 | (-1.48 to -0.75) |
| Boys | 13-15 | 1989-2019 | 0.88 | (0.85 to 0.9) |
| Girls | 7-9 | 1989- | 0.38 | (0.28 to 0.48) |
| Girls | 7-9 | 1995(0.35) | 1.11 | (1.08 to 1.14) |
| Girls | 7-9 | 2013(0.26) | 0.04 | (-0.07 to 0.15) |
| Girls | 7-9 | 1989-2019 | 0.73 | (0.71 to 0.75) |
| Girls | 10-12 | 1989- | 2.9 | (2.55 to 3.25) |
| Girls | 10-12 | 1992(0.23) | 1.17 | (1.13 to 1.21) |
| Girls | 10-12 | 2005(0.41) | 0.55 | (0.48 to 0.61) |
| Girls | 10-12 | 2015(0.49) | -0.31 | (-0.58 to -0.03) |
| Girls | 10-12 | 1989-2019 | 0.93 | (0.91 to 0.96) |
| Girls | 13-15 | 1989- | 3.23 | (2.84 to 3.62) |
| Girls | 13-15 | 1992(0.41) | 2.2 | (2.08 to 2.31) |
| Girls | 13-15 | 1998(0.51) | 1.35 | (1.12 to 1.58) |
| Girls | 13-15 | 2004(0.32) | 0 | (-0.05 to 0.05) |
| Girls | 13-15 | 1989-2019 | 1.04 | (1.01 to 1.06) |

Supplementary Table 4.7: Results of joinpoint regression analysis for SAR: estimates of the joinpoints (standard errors), ACP/AACP with 95% CI by gender and age groups.

| Gender | Age group | Segment | Estimate | 95% CI |
| --- | --- | --- | --- | --- |
| Boys | 7-9 | 1989- | 0.88 | (0.79 to 0.98) |
| Boys | 7-9 | 1996(0.25) | -0.19 | (-0.21 to -0.17) |
| Boys | 7-9 | 1989-2019 | 0.06 | (0.05 to 0.08) |
| Boys | 10-12 | 1989- | 1.5 | (1.4 to 1.59) |
| Boys | 10-12 | 1996(0.11) | -1.08 | (-1.13 to -1.03) |
| Boys | 10-12 | 2009(0.32) | -0.18 | (-0.25 to -0.12) |
| Boys | 10-12 | 1989-2019 | -0.16 | (-0.18 to -0.14) |
| Boys | 13-15 | 1989- | 1.33 | (1.21 to 1.45) |
| Boys | 13-15 | 1996(0.11) | -1.16 | (-1.2 to -1.11) |
| Boys | 13-15 | 2011(0.27) | 0.16 | (0.04 to 0.27) |
| Boys | 13-15 | 1989-2019 | -0.23 | (-0.26 to -0.21) |
| Girls | 7-9 | 1989- | 0.22 | (0.21 to 0.23) |
| Girls | 7-9 | 2016(0.23) | 1.31 | (1.08 to 1.54) |
| Girls | 7-9 | 1989-2019 | 0.34 | (0.32 to 0.35) |
| Girls | 10-12 | 1989- | 2.67 | (2.41 to 2.93) |
| Girls | 10-12 | 1992(0.11) | -0.27 | (-0.33 to -0.22) |
| Girls | 10-12 | 2004(0.35) | -1.19 | (-1.39 to -1) |
| Girls | 10-12 | 2008(0.15) | 0.96 | (0.9 to 1.03) |
| Girls | 10-12 | 1989-2019 | 0.36 | (0.34 to 0.38) |
| Girls | 13-15 | 1989- | 1 | (0.9 to 1.1) |
| Girls | 13-15 | 1996(0.14) | -1.05 | (-1.1 to -1) |
| Girls | 13-15 | 2010(0.14) | 1.31 | (1.21 to 1.41) |
| Girls | 13-15 | 1989-2019 | 0.17 | (0.15 to 0.2) |

Supplementary Table 4.8: Results of joinpoint regression analysis for BAH: estimates of the joinpoints (standard errors), ACP/AACP with 95% CI by gender and age groups.

| Gender | Age group | Segment | Estimate | 95% CI |
| --- | --- | --- | --- | --- |
| Boys | 7-9 | 1989- | -0.33 | (-0.4 to -0.25) |
| Boys | 7-9 | 1997(0.46) | -1.04 | (-1.16 to -0.92) |
| Boys | 7-9 | 2005(0.44) | -1.99 | (-2.43 to -1.55) |
| Boys | 7-9 | 2007(0.17) | 0.32 | (0.27 to 0.37) |
| Boys | 7-9 | 1989-2019 | -0.41 | (-0.42 to -0.39) |
| Boys | 10-12 | 1989- | -0.32 | (-0.39 to -0.25) |
| Boys | 10-12 | 1998(0.31) | -1.1 | (-1.15 to -1.06) |
| Boys | 10-12 | 2010(0.19) | 1.01 | (0.71 to 1.3) |
| Boys | 10-12 | 2015(0.34) | -0.37 | (-0.57 to -0.17) |
| Boys | 10-12 | 1989-2019 | -0.47 | (-0.49 to -0.46) |
| Boys | 13-15 | 1989- | 0.9 | (0.38 to 1.42) |
| Boys | 13-15 | 1992(0.32) | -0.77 | (-0.8 to -0.73) |
| Boys | 13-15 | 2010(0.2) | 1.1 | (0.9 to 1.31) |
| Boys | 13-15 | 2016(0.24) | -1.16 | (-1.55 to -0.78) |
| Boys | 13-15 | 1989-2019 | -0.29 | (-0.32 to -0.25) |
| Girls | 7-9 | 1989- | -0.49 | (-0.51 to -0.47) |
| Girls | 7-9 | 2008(0.34) | 0.26 | (0.19 to 0.32) |
| Girls | 7-9 | 1989-2019 | -0.23 | (-0.24 to -0.21) |
| Girls | 10-12 | 1989- | 0.61 | (0.44 to 0.78) |
| Girls | 10-12 | 1993(0.38) | -0.17 | (-0.24 to -0.11) |
| Girls | 10-12 | 2004(0.31) | -1.42 | (-1.72 to -1.11) |
| Girls | 10-12 | 2008(0.22) | 0.47 | (0.41 to 0.53) |
| Girls | 10-12 | 1989-2019 | 0.02 | (0 to 0.04) |
| Girls | 13-15 | 1989- | 1.4 | (0.87 to 1.92) |
| Girls | 13-15 | 1992(0.43) | 0.12 | (0.05 to 0.19) |
| Girls | 13-15 | 2001(0.48) | -0.61 | (-0.73 to -0.49) |
| Girls | 13-15 | 2009(0.33) | 0.65 | (0.55 to 0.75) |
| Girls | 13-15 | 1989-2019 | 0.24 | (0.21 to 0.27) |

Supplementary Table 4.9: Results of joinpoint regression analysis for D60: estimates of the joinpoints (standard errors), ACP/AACP with 95% CI by gender and age groups.

| Gender | Age group | Segment | Estimate | 95% CI |
| --- | --- | --- | --- | --- |
| Boys | 7-9 | 1989- | 0.95 | (0.82 to 1.08) |
| Boys | 7-9 | 1995(0.34) | 0.06 | (-0.01 to 0.14) |
| Boys | 7-9 | 2004(0.82) | -0.3 | (-0.38 to -0.23) |
| Boys | 7-9 | 2013(0.52) | -1.01 | (-1.16 to -0.86) |
| Boys | 7-9 | 1989-2019 | -0.1 | (-0.12 to -0.08) |
| Boys | 10-12 | 1989- | 0.51 | (0.39 to 0.63) |
| Boys | 10-12 | 1994(0.41) | -0.07 | (-0.1 to -0.04) |
| Boys | 10-12 | 2011(0.64) | -0.57 | (-0.68 to -0.46) |
| Boys | 10-12 | 1989-2019 | -0.1 | (-0.12 to -0.08) |
| Boys | 13-15 | 1989- | -0.25 | (-0.29 to -0.21) |
| Boys | 13-15 | 2002(0.98) | -1.05 | (-2.08 to -0.01) |
| Boys | 13-15 | 2004(0.84) | -0.4 | (-0.45 to -0.35) |
| Boys | 13-15 | 1989-2019 | -0.38 | (-0.4 to -0.36) |
| Girls | 7-9 | 1989- | 0.51 | (0.46 to 0.56) |
| Girls | 7-9 | 2001(0.2) | -0.63 | (-0.65 to -0.6) |
| Girls | 7-9 | 1989-2019 | -0.19 | (-0.2 to -0.18) |
| Girls | 10-12 | 1989- | 0.07 | (-0.01 to 0.15) |
| Girls | 10-12 | 1996(0.61) | 0.71 | (0.32 to 1.09) |
| Girls | 10-12 | 2000(0.43) | -0.36 | (-0.41 to -0.3) |
| Girls | 10-12 | 2012(0.68) | -0.81 | (-0.92 to -0.71) |
| Girls | 10-12 | 1989-2019 | -0.23 | (-0.25 to -0.21) |
| Girls | 13-15 | 1989- | -0.25 | (-0.37 to -0.12) |
| Girls | 13-15 | 1996(0.27) | 1.32 | (0.83 to 1.8) |
| Girls | 13-15 | 1999(0.3) | -0.27 | (-0.33 to -0.21) |
| Girls | 13-15 | 2011(0.47) | -1.16 | (-1.3 to -1.02) |
| Girls | 13-15 | 1989-2019 | -0.37 | (-0.4 to -0.35) |

Supplementary Table 4.10: Results of joinpoint regression analysis for R600: estimates of the joinpoints (standard errors), ACP/AACP with 95% CI by gender and age groups.

| Gender | Age group | Segment | Estimate | 95% CI |
| --- | --- | --- | --- | --- |
| Boys | 7-9 | 1989- | 1.61 | (1.54 to 1.68) |
| Boys | 7-9 | 1997(0.17) | 0.14 | (0.09 to 0.19) |
| Boys | 7-9 | 2010(0.27) | -0.88 | (-0.95 to -0.81) |
| Boys | 7-9 | 1989-2019 | 0.23 | (0.21 to 0.25) |
| Boys | 10-12 | 1989- | 1.03 | (0.98 to 1.07) |
| Boys | 10-12 | 1999(0.52) | 0.56 | (0.5 to 0.62) |
| Boys | 10-12 | 2010(0.28) | -0.93 | (-1.22 to -0.63) |
| Boys | 10-12 | 2015(0.55) | -0.01 | (-0.22 to 0.2) |
| Boys | 10-12 | 1989-2019 | 0.41 | (0.39 to 0.43) |
| Boys | 13-15 | 1989- | 0.42 | (0.31 to 0.54) |
| Boys | 13-15 | 1995(0.36) | 1.74 | (1.3 to 2.19) |
| Boys | 13-15 | 1999(0.37) | 0.31 | (0.24 to 0.38) |
| Boys | 13-15 | 2009(0.45) | -0.5 | (-0.6 to -0.41) |
| Boys | 13-15 | 1989-2019 | 0.25 | (0.23 to 0.27) |
| Girls | 7-9 | 1989- | 1.81 | (1.75 to 1.86) |
| Girls | 7-9 | 1999(0.12) | -0.37 | (-0.41 to -0.32) |
| Girls | 7-9 | 2013(0.73) | -0.84 | (-0.99 to -0.7) |
| Girls | 7-9 | 1989-2019 | 0.28 | (0.26 to 0.3) |
| Girls | 10-12 | 1989- | 0.93 | (0.85 to 1.01) |
| Girls | 10-12 | 1996(0.52) | 2.23 | (1.42 to 3.05) |
| Girls | 10-12 | 1999(0.31) | 0.07 | (0.02 to 0.13) |
| Girls | 10-12 | 2010(0.4) | -0.68 | (-0.77 to -0.59) |
| Girls | 10-12 | 1989-2019 | 0.27 | (0.25 to 0.29) |
| Girls | 13-15 | 1989- | 0.85 | (0.75 to 0.95) |
| Girls | 13-15 | 1997(0.29) | 2.92 | (1.89 to 3.96) |
| Girls | 13-15 | 1998(0.21) | 0.2 | (0.14 to 0.27) |
| Girls | 13-15 | 2010(0.39) | -0.8 | (-0.92 to -0.69) |
| Girls | 13-15 | 1989-2019 | 0.22 | (0.2 to 0.25) |
